# The Tsetlin Machine: A “Third Way” in QSAR Modeling

**DOI:** 10.1021/acs.jcim.5c03109

**Published:** 2026-05-28

**Authors:** Paul F. A. Clarke, Ivan Cmelo, Runar Helin, Mayur Kishor Shende, Ole-Christoffer Granmo, Darren Fayne

**Affiliations:** † Department of Information and Communication Technology, Faculty of Engineering and Science, University of Agder, 4879 Grimstad, Norway; ‡ Molecular Design Group, School of Chemical Sciences, 8818 Dublin City University, D09 V209 Dublin, Ireland; § DCU Life Sciences Institute, Dublin City University, D09 V209 Dublin, Ireland; ∥ Department of Informatics and Chemistry & CZ-OPENSCREEN: National Infrastructure for Chemical Biology, Faculty of Chemical Technology, 52735 University of Chemistry and Technology, 166 28 Prague 6, Czech Republic

## Abstract

Advances in Quantitative Structure Activity Relationship (QSAR) are led by two core paradigms, (1) descriptor engineering, where complex fixed-length vectors of compounds are generated and conventional ML methods are applied to those representations and (2) graphical chemical inputs (e.g., Simplified Molecular Input Line Entry System (SMILES), 2D-graph) being provided to deep learning neural network (NN) models, which construct their own internal representations of molecules and learn iteratively over them. Here we present the Tsetlin Machine (TM)—which combines the accuracy and easy-use of existing rule-based QSAR ML methods (e.g., RF and XGBoost), the iterative learning aspect of NN algorithms and its intrinsic interpretability. The TM uses teams of finite-state automata which capture frequent patterns as propositional logic (*clauses*) via reinforcement learning. The benchmarking pipeline presented here demonstrates that TM-QSAR coupled with ECFP4 descriptors frequently performs better than existing rule-based QSAR methods for ROC-AUC, PRC-AUC and PPV, with a high capacity for interscaffold generalization. However, due to the binary nature of TM-QSAR, performance it is currently limited when descretised continuous descriptors are used. TM-QSAR demonstrated particularly impressive classification scores for MOR (ROC-AUC = 0.87, PRC-AUC = 0.77) and CYPA4 (ROC-AUC = 0.92, PRC-AUC = 0.63), when compared to RF and XGBoost. Using TM in combination with substructural fingerprinting descriptors allows for an interpretability suite which can be extracted directly from clauses. Here we detail molecule property maps (TM-MPM) to view atom-wise TM-QSAR bioactivity contributions for single molecules and closed-form WAC scores (**W**eights × **A**ctivations × **C**lauses) for descriptor-wise contributions to regions of predicted chemical space. These methods show strong alignment of TM-QSAR interpretations to known ligand–protein interactions of the MOR target and gives nonlinear, conditional interpretations for greater predicted bioactivity. Given this combination of accuracy, computational efficiency and interpretability, we provide a basis for TM-QSAR to be explored as a standard methodology in virtual screening toolkits.

## Graphical abstract

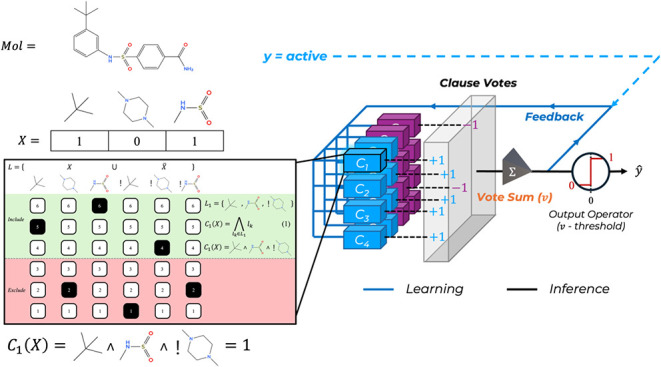

## 1 Introduction

The goal of Quantitative Structure Activity Relationship (QSAR) is to find statistically relevant patterns in data between chemical structures and their biological activity properties (active, inactive, toxic, nontoxic).^1,2^ These patterns can be used to identify and prioritise the most promising molecules likely to have desirable physicochemical properties, most often biological activity against a pharmacological target of interest. QSAR-based methodologies expedite development of compounds that would take considerable time and resources to find via a trial-and-error Edisonian approach.^3^ This is usually achieved by having QSAR-based methodologies screen vast virtual libraries of available compounds in a process termed “virtual screening”. When using machine learning (ML) to construct QSAR models, two main paradigms have emerged. The first relies on feature engineering, where either 3D or 2D graph representations of molecules are converted into binary representations (e.g., presence/absence of substructures), their calculated numerical properties (e.g., Log *P*, MW) or some combination of both. ML algorithms find patterns within these calculated representations and quantify their contribution toward a classification. These conventional methods generally rely on efforts of descriptor research, where the goal is to represent molecules in a descriptive yet condensed format. The information-rich representation of molecules is then combined with generic ML algorithms which mine for patterns in these fixed-length representations as input (e.g., SVM, GPR, RF and XGBoost).^4^

The second way relies on Neural Network (NN) algorithms to construct their own representations of molecules using their graphical form as initial input. Molecules are presented to NNs in forms usually used in communication between chemists, such as Simplified Molecular Input Line Entry System (SMILES), and with no additional knowledge provided, NNs incrementally construct their own descriptors as an internal representation within hidden layers via supervised learning. This approach has shown to be immensely flexible in terms of mapping these chemical inputs to any property output, with endless variations of architectures. For example, MolBERT (85 M parameters) considers molecules as text language with SMILES as input and utilizes the transformer architecture and self-supervised learning to predict a range of molecular activities.^5^ GROVER takes this further as a massive Graph Neural Network (GNN) of 100 M parameters combining Message Passing Networks, Transformer-style architectures, attention mechanisms, self-supervised pretraining on 10 million molecules and task fine-tuning for predicting molecular properties (activity, drug-likeness, quantum mechanical) directly from the 2D graph of a molecule.^6^ From the onset, it appears as if there is an architectural variation of NNs which can handle any graphical chemical input and achieve high performance metrics for any molecular property. Due to this inherent flexibility, the bypassing of feature engineering and their incremental learning capabilities, the use of NNs for QSAR modeling has become prolific.^7^

Despite their widespread use, NN-based QSAR often fails to generalize outside the data sets they have been trained on which makes their accuracy reliant on accessing vast amounts of data.^8^ Recent independent studies have shown that massive NNs often perform worse, the same or just marginally better than conventional ML methods when it comes to different performance metrics on more practical QSAR tasks where data is limited.^4,9–11^ These studies have revealed that having a knowledge-rich representation of molecules coupled to conventional ML works just as well, or better for QSAR modeling tasks for data sets ranging from 1000–100,000 samples. The performance comparisons are particularly striking when considering the computational efficiency and hardware requirements of basic conventional ML methods such as RF and XGBoost, relative to NNs.

While effective, most conventional ML methods lack the incremental learning capabilities inherent to NNs, and both paradigms are limited in their inherent interpretability. To bridge this gap, we introduce the Tsetlin Machine (TM) to QSAR modeling. The TM represents a novel “third way” approach to QSAR tasks as it incorporates the iterative refinement and versatility of NNs, the computational efficiency of conventional ML methods and facile interpretability without the use of wrapper functions or repeated inference. The TM uses a combination of reinforcement learning, game theory and resource allocation principles to create coordinating, but decentralised rules called *clauses* which capture patterns in chemical data as conjunctive propositions (i.e., AND-rules).^12^ Until now, the TM has been practically ignored as a potential QSAR method, despite some initial testing with other developing concepts, however the performance of baseline TM models has yet to be validated quantitatively against existing methods.^13^ There are two main goals of this study. The first is to examine how well these clauses define and utilize patterns in chemical space to predict molecular properties and to determine how many epochs of iterative learning, and localized clauses, are needed to reach state-of-the-art (SOTA) performances.

The second is to demonstrate methods of interpretability for TM-QSAR in combination with Extended Connectivity Fingerprints (ECFP4) descriptors. Since its inception, the TM was built with interpretability in mind.^12^ Clauses serve as logical conditions for *Active* or *Inactive* molecules, which are then used in conjunction with their weights to give prediction contributions of molecular fragments to activity. Here, the model is a logical explanation, the logical explanation is the model and QSAR rules can be directly interpreted. In contrast to RF and XGBoost, randomness in the TM is introduced via stochastic feedback mechanisms, single rules (clauses) are also constructed with one another in mind without an interconnected structure as in the case of NNs. Therefore, single clauses can be read as-is in isolation from the other rules, providing local interpretations for specific regions of predicted chemical space.

The information in clauses can then be further condensed to extract descriptor importances and molecular maps of predicted property values where atom-wise contributions are visualized. This is done directly from the TM model itself, without the use of additional interpretability models or wrapper functions such as *explain* = *SHAP*(*model*)^14^ or by imputing features and running repeated inference. By circumventing these operations, the TM allows for low-cost interpretability at any scale of data. These methods are detailed and conducted here as part of a case study for μ Opioid Receptor.

To evaluate raw TM-QSAR performances, we have developed a method comparison pipeline, implemented entirely in Python which expands upon the benchmarking pipeline recently proposed by Ash et al.^15^ To ensure rigorous evaluation, the pipeline provides statistical reproducibility and robustness through a repeated 5 × 5 cross validation (CV) scheme coupled with repeated measures ANOVA and Tukey’s HSD statistical tests. As proponents of TM-QSAR, we also try to mitigate our own bias by expanding on their pipeline method with classification criteria for defining when a method is “better”, “equivalent” or “worse” than the existing rule-based SOTA. This scheme increases the number of ways for the TM to be considered “equivalent” or “worse” than existing methods, providing a probabilistic handicap to offset unintentional experimenter bias. The pipeline itself utilizes Pixi for managing the Python environment, complete with pixi.toml and pixi.lock files for easy reproducibility.^16^

The method comparison pipeline is conducted using other rule-based QSAR methods, RF and XGBoost, both of which are well-established in a QSAR context. Such algorithms have been demonstrated as the SOTA for all data sets studied here, exceeding the performance of large parameter NNs.^4^

## 2 Methods

### 2.1 Data Curation

The data sets selected for this study are a subset of a broader study conducted by Deng et al.^4^ A subset was used due to the inclusion of a hyperparameter (HP) search regime (Section 2.5) which increased computational requirements and was not pursued in the original study. The data sets of small molecules chosen for this benchmarking study are summarized in Table 1, along with their number of samples and percentage of active molecules. The focus of this benchmarking study is limited to cytochrome P450 enzymes and opioid receptors. There is interest in developing opioid analgesics with reduced overdose effects through pharmacokinetic (PK) and pharmacodynamic (PD) perspectives. The PK perspective attempts to reduce overdose events by avoiding excessive amounts of opioids at the action site. Key PK-related targets include cytochrome P450 2D6 (CYP2D6) and cytochrome P450 3A4 (CYP3A4).^17^ The PD perspective attempts to prevent overdose outcomes by avoiding off-target effects. PD-related targets include μ opioid receptor (MOR), δ opioid receptor (DOR) and κ opioid receptor (KOR).^18^ We have used the exact data sets used by Deng et al.^4^ as made public on the GitHub repository for the project, which were originally derived from ChEMBL. Here, we examine the original TM architecture designed for classification. To this end, we have chosen those data sets where the pIC_50_ label is converted to binary *Active/Inactive* form using a threshold value of 6, whereby molecules with pIC_50_ greater than 6 are labeled *Active* (1) otherwise *Inactive* (0). This choice of threshold provided a known, low number of test-set edge cases of 1–5%, where an “edge case” here is defined as a weakly *Active* molecule which is regarded as *Inactive*, or an *Inactive* molecule regarded as *Active*. Furthermore, this allowed for straightfoward comparison with the results of Deng *et. al*,^4^ where the original data sets were sourced.

**Table 1:** Datasets Subject to Benchmarking Pipeline, Their Sample Size (*N*) and Their Percentage of Active Compounds (% Actives)

target data set	*N*	% actives
MOR	3534	29.7
DOR	3224	23.3
KOR	3327	27.8
CYP2D6	2294	1.4
CYP3A4	3672	2.2

#### 2.1.1 Data Set Selection Criteria

Data sets for this study are selected via the following criteria:

1. Performance of most similar existing methods: RF and XGBoost were demonstrated to achieve high metric scores compared to all other methods, including large parameter NNs such as MolBERT and GROVER, on these data sets.
2. Established performance statistics: Selected data sets are well-studied with readily available statistics on metric scores for conventional ML and representation learning methods.
3. Domain expertise: Our group has direct experience in the creation and curation of these data sets which provides good estimates for lower and upper bound ML prediction performances.^17^
4. Recognized standards: Those data sets studied here are featured on the Polaris platform and are classified as having reached a standard of data set quality.^19^ Polaris aims to be a single source of immutable, ML ready data sets and benchmarking procedures with the goal of enabling reproducible ML in drug discovery for robust comparisons of methods.

The molecules in each data set are standardized using the ChEMBL Structure Pipeline’s standardizer.^20^ In this process, the molecules are checked for validity, standardized to defined conventions set out by the FDA/IUPAC, and finally stripped of salts and solvents. If a chemical structure is determined to be invalid, i.e., it cannot be parsed or does not pass the pipelines’ inbuilt sanity checks, it is subsequently removed from the data set prior to downstream modeling.

### 2.2 Benchmarking Experimental Design

The components of the benchmarking pipeline are designed to produce a robust, statistically replicable comparison between the TM and established rule-based methods, Random Forest and XGBoost.

#### 2.2.1 The Tsetlin Machine (TM)

Proposed in 2018 by Granmo, the TM solves complex pattern recognition problems with propositional formulas (*Clauses*), composed by a collective of Tsetlin Automata (TA).^12^ These collectives of automata cooperate in a game to capture different patterns in the data. The fundamental unit of the TM is the TA (Figure 1), a finite state machine which performs one of two actions: *Include* or *Exclude* a specific feature, or mathematically, α_*z*_, *z*∈{1, 2}, depending on its current state (1 – 2N), in an environment with unknown *reward* and *penalty* probabilities.

**Figure 1:**
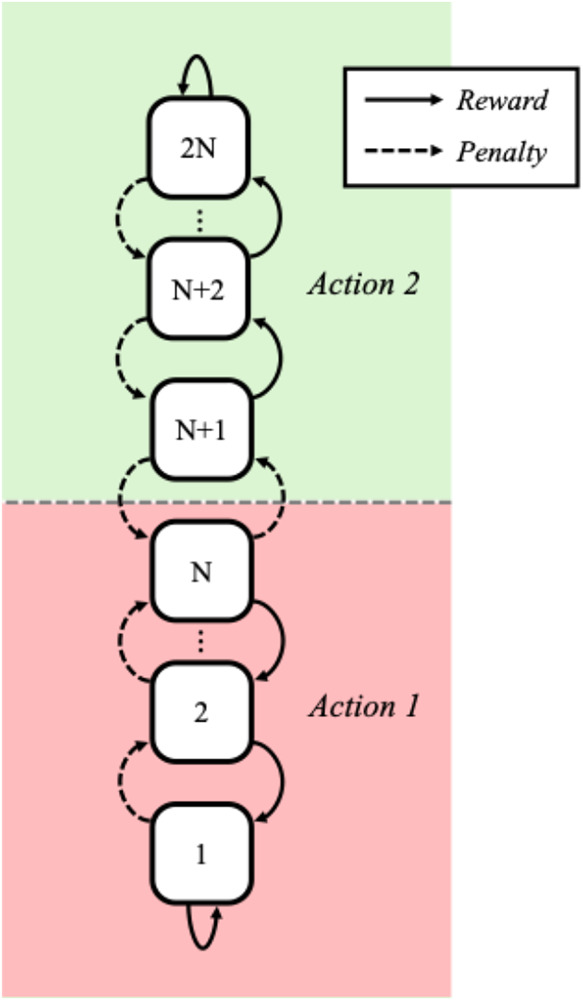
A TA in a two-action environment.

The TA in the above figure has 2*N* states. *Action 1* (α_1_) is performed in the states 1 to *N*, while *Action 2* (α_2_) is performed in the states *N* + 1 to 2*N*. State transitions of the TA allow for learning, where rewards and penalties, in response to a TA’s current action, trigger specific transitions from one state to another, with the goal of reinforcing successful actions. The states act as memory, where if *Action 1* led to a reward, the state will increase away from the transition to *Action 2*, strengthening memory for *Action 1*. If Action 1 led to a penalty, the state would decrement toward the transition to Action 2, weakening the memory of *Action 1* in favor of *Action 2*. The strength of such a finite state learning TAs in bandit problems is that they are extremely adaptive and collaborate effectively in teams within dynamic environments.

In the TM, teams of TA are tasked with constructing a propositional formula or conjunctive *clause* (e.g., SubStructure1 AND SubStructure3 AND NOT SubStructure7) where the action states are to either *Include* or *Exclude* a binary feature (e.g., SubStructure7) or its negation (e.g., NOT SubStructure 7) into said clause. The entire set of binary/boolean features (*X*), with their negated counterparts are termed *literals* where *L* = {*X*∪ *X̅*}. For example, if Extended Connectivity Fingerprints (ECFP) of length 1,024 were calculated, the entire set of literals would be the original fingerprint concatenated with its logical inverse to give a vector of length 2,048. As a condensed illustration, consider the QSAR example illustrated in Figure 2, where the feature-set of compounds is represented by the presence and absence of just three chemical fragments, resulting in six total literals including negations. Within a conjunctive clause, one TA is assigned to each literal where its objective is to decide whether to *Include* (*Action 1*) or *Exclude* (*Action 2*) the literal into the clause, based on its current state, which changes in response to reinforcements that achieve learning objectives. The subset of literals, *L*_*j*_⊆*L*, are those whose TA state is greater than or equal to *N* + 1 and TA *Action* is *Include*. A clause (*C*_*j*_) is formed by ANDing the included subset *Lj*⊆*L* of the literal set (eq 1), where *j* is the index of the literal subset and resulting clause.

1 $$C_{j}\left(X\right)=\Lambda_{l_{k}\in L_{j}}l_{k}$$

**Figure 2:**
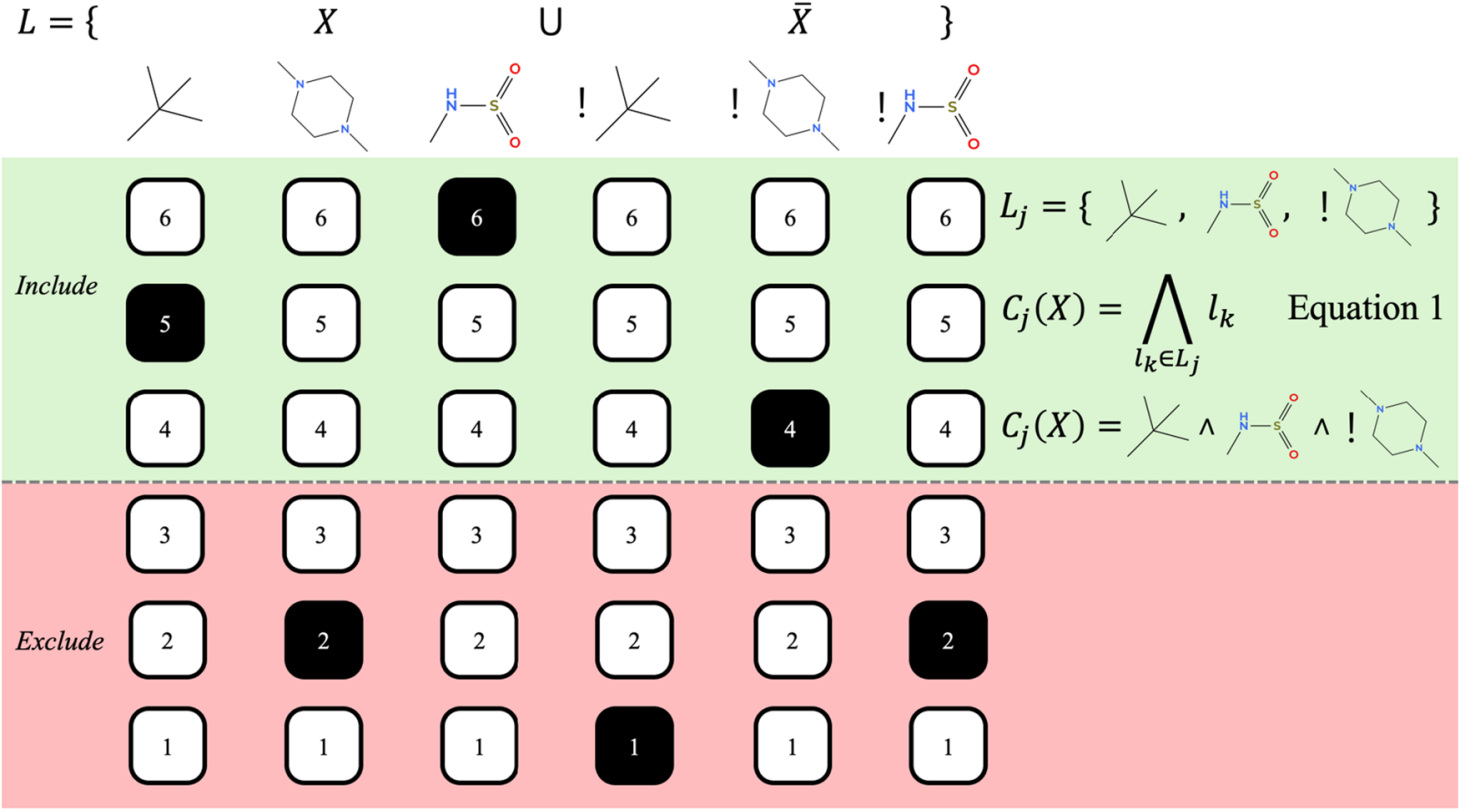
Fragment clause constructed by a team of TA. States in black are those states currently occupied by the 6-state TA.

Considering just a single clause, this team of TAs coordinate in capturing these conditional patterns for a given class (e.g., *Active* or *Inactive*). When learning, they do so by receiving continuous feedback from presented training sample molecules, and changing their states accordingly as depicted in Figure 1. *Type I* feedback produces frequent patterns present in molecules of the assigned class, which can be likened to *Active* clauses identifying specific combinations of atoms necessary for activity. *Type II* feedback increases the discrimination power of these patterns against the opposing class. This identifies molecular features that are associated with inactive molecules, such as potential steric clashes or forbidden fragments. Below, we detail examples for the *Active* TM clauses in response to *Active* and *Inactive* molecules. The same procedure is conducted for *Inactive* TM clauses but for the inverse class of samples, which have the opposing objective of classifying *Inactive* molecules.

*Type I Feedback:*

Type I feedback suppresses false negative (FN) and reinforces true positive (TP) clause output.

(a) *Type I­(a)* or “Recognize” feedback occurs when an *Active* clause is evaluated as true for an *Active sample*. Literal TA states which are true for the molecule increase in value and become memorized by the clause. Formally, *Include* is rewarded and *Exclude* is penalised with probability $\frac{s-1}{s}$ if *C*_*j*_(*X*) = 1 and *l*_*k*_ = 1. *Active* clauses begin to take on the appearance of patterns in *Active* molecules.
(b) *Type I­(b)* or “Erase” feedback occurs when an *Active* clause is evaluated as false for an *Active* sample. It essentially missed a TP *Active* molecule. Irrespective of the value of literals for the molecule, all literal TA states decrease in value with a low of probability $\frac{1}{s}$ if *C*_*j*_(*X*) = 0. Conditional substructure rules are essentially forgotten. Formally, TA states in the *Include* action for a literal are penalised, while TA states in the *Exclude* action for a literal are rewarded.

*Active* clauses are forced to learn patterns *relevant* to *Active* molecules and are penalised for clauses which produce FNs by essentially forgetting their entire structure.

*Type II Feedback:*

*Type II* or “Reject” feedback suppresses false positives (FPs) by allowing *Active* clauses to still recognize patterns of *Active* molecules while being able to reject *Inactive* molecules. This occurs when an *Active* clause is true for an *Inactive* sample. It penalises literal TAs with a probability of 1 which *Exclude* literals *not* found in the *Inactive* molecules, allowing them to be included in *Active* clauses.

*None Feedback:*

Occurs when an *Active* clause is false for an *Inactive* sample. Here, True negatives (TNs) are ignored, no feedback to the clause is provided and its TA states are retained.

Hyper-parameter *s*, *s* ≥ 1, controls how strongly we favor *Include* literals. As a generalization, small *s* tends to generate clauses which capture more frequent patterns. A higher value of *s* will give more specific clauses, which include more literals.

These teams of TA (*clauses*) coordinate by distributing themselves across different frequent patterns via a resource allocation strategy which uses their aggregated output (*clause vote sum*, *v*, eq 2) for molecule examples. *Active* clauses are given a vote of positive polarity (+1) when evaluated as true and *Inactive* clauses are given a vote of negative polarity (−1) when true. For each sample, the resulting clause vote sum (*v*) is obtained and is used to calculate the clause feedback probability (CFP, eqs 3 and 4) when presented with samples. A random value *P*, *P* ∼ *Uniform*(0, 1), is then drawn and if *P* < CFP for a clause then it will receive the appropriate feedback outlined previously (*Type Ia, Type Ib, Type II* or *None*). CFP uses an experimenter chosen hyperparameter, *T*, which in essence decides how clauses will be distributed among data set patterns. For instance, in the case of an *Active* molecule if *v* > *T*, neither rewards or penalties are provided to clauses, leaving them free to learn other subpatterns. Figure 3 illustrates this mechanism for clause coordination through clause vote sum, clause feedback probability and feedback type for assigned *Active* and *Inactive* clauses. The diversity of clauses across the descriptor space is driven by the different feedback types outlined previously in combination with this resource allocation strategy which forces clauses to distribute themselves across the range of subpatterns which exist in the descriptor set.

2 $$v=\sum_{j=1,2,...,n/2-1}C_{j}\left(X\right)-\sum_{j=n/2,...,n}C_{j}\left(X\right)$$

where the first half of a selected number of clauses (*n*) are assigned as *Active* clauses with positive voting polarity and the second half of clauses are *Inactive* clauses with a negative voting polarity.

3 $${\mathrm{CFP}}_{\mathrm{active}}=\frac{T+\mathrm{clip}\left(v\right)}{2T}$$

when *y* = 1 or *y* = *active* for a molecule sample.

4 $${\mathrm{CFP}}_{\mathrm{inactive}}=\frac{T-\mathrm{clip}\left(v\right)}{2T}$$

when *y* = 0 or *y* = *inactive* for a molecule sample.

**Figure 3:**
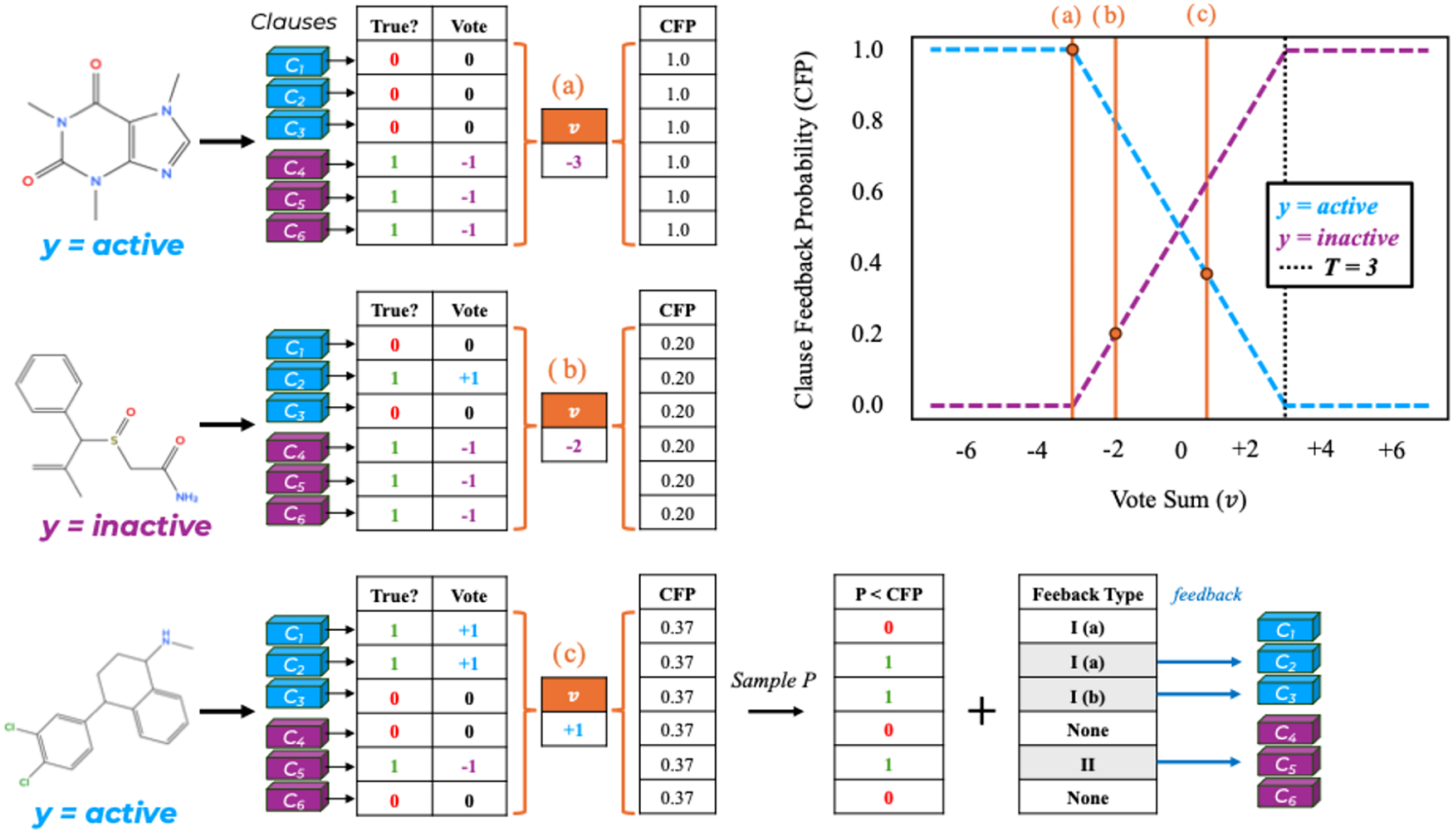
Generation of clause feedback probability (CFP) using *clause vote sum*, *v*, which allows clauses to coordinate with each other to capture different patterns in data. (a) *Active* molecule is wrongly labeled *Inactive* (*v* < 0) where *v* ≤ -*T*, forcing all clauses to receive feedback. (b) *Inactive* molecule correctly labeled *Inactive* where – *T* < *v* < + *T*, all clauses have a low probability of receiving feedback. (c) *Active* molecule is correctly labeled *Active* (*v* ≥ 0) but not by a large clause vote sum – all clauses have a modest probability of receiving feedback.

The above illustration (Figure 3) demonstrates how clause vote sum, CFP and the different feedback types of feedback work in practice.

(a) *Active* molecule is wrongly labeled *Inactive* (*v* < 0) where *v* ≤ – *T*, resulting in a CFP of 1.0 for all clauses using CFP_active_ (eq 3). Thus, all *Active* clauses will receive *Type I­(a)*, “Recognize” or *Type I­(b)*, “Erase” feedback while all *Inactive* clauses will receive *Type II*, “Reject” feedback or *None* feedback.
(b) *Inactive* molecule is correctly labeled *Inactive* (*v* < 0) where – *T* < *v* < + *T*, resulting in a low CFP (e.g., 0.20) for all clauses using CFP_inactive_ (eq 4). Thus, *Active* clauses have a low probability of receiving *Type II* or *None* feedback and *Inactive* clauses have a low probability of receiving *Type I­(a)* or *Type I­(b)* feedback.
(c) *Active* molecule is correctly labeled as *Active* (*v* > 0) where – *T* < *v* < + *T*, but with a low vote sum (*v* = +1), resulting in a *CFP* of 0.37 for all clauses. Thus, *Active* clauses have a significant probability in receiving *Type I­(a)* or *Type I­(b)* feedback. For *Inactive* clauses, where have a significant probability of receiving *Type II* or *None* feedback.

Individual TAs within each clause will receive feedback with the probabilities outlined previously. In short, when the TM is far off in its predictions for certain molecules, there is a high probability that the necessary clauses, and only the necessary clauses, will receive feedback. The TM’s clauses will receive little-to-no feedback when molecules have been labeled correctly. Thus, the TM focuses TA learning capabilities where they are needed most in chemical space. Once clauses have been trained over iterations, the clause vote sum, *v*, is used for inference by applying an output operator which is a threshold in the case of classification and the feedback mechanism is stopped (Figure 4). In its entirety, the TM operates via simple state updates via reinforcement learning, boolean operators, summation and thresholds. This allows the TM to learn and conduct inference on much simpler hardware than GPUs, even going so far as running on its own dedicated FPGA chips.^21–23^

**Figure 4:**
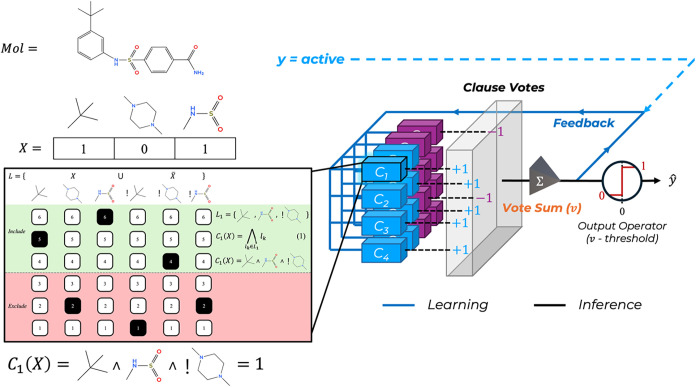
Diagram of TM learning and inference, from TA states in a single clause, to thresholding Vote Sum (*v*) for final classification.

Beyond predictions, the output of this game which combines elements of frequent pattern mining, resource allocation and decentralised teams of TA is a trained set of *clauses* which recognize subpatterns of chemical space. Their predictions are more local and decentralised when compared to RF and XGBoost, where each tree provides a prediction for every presented molecule. TM clauses are built with one another in mind but are localized to specific regions of the sampled chemical space and are only activated when evaluated as true for specific molecules (Figure 4). With the clauses built upon chemical substructures, this localization also pertains to specific parts of the chemical space. The purpose of our work is to determine if this iterative reinforcement-based, decentralised approach to QSAR modeling can achieve performance comparable to or even exceeding the current SOTA.

Many variations of the TM have been developed in recent years^24^ and in this study, we use a version of the TM which uses a single (coalesced) set^25^ of weighted clauses^26^ which is referred to here as baseline TM or just TM. Conceptually, the adjustments to the TM used here are mainly made to improve the readability of TM clauses, aiding in the interpretability of resulting TM-QSAR models. For initial testing we have used TM models from the Tsetlin Machine Unified (TMU) library and for parallelization across samples we have used the pyTsetlinMachineParallel library.

#### 2.2.2 Random Forest

Despite its simplicity Random Forest (RF) retains a high status among QSAR methods and a *de facto* gold standard in methods to beat.^4,27,28^ For RF models, a predetermined number of decision trees are built independently from one another using a random subsample-space and a random subdescriptor-space of the original data set. In the case of classification QSAR tasks, the resulting classifications of each tree for a single object is aggregated, counted and a majority vote will be the final decision of the overall “forest” model. Thus, the final model is made up of different, strongly opinionated smaller models, where errors of a single tree are mitigated by the total population of trees.^29^

#### 2.2.3 XGBoost

eXtreme Gradient Boosting or XGBoost iteratively trains decision trees to correct the errors of the previous trees. New trees are added to the model which focus on samples incorrectly predicted previously. Rules or “trees” are built in succession, one after another and classification results from each tree are bagged as before for RF. Each tree will have its own classification output which will contribute to each object’s overall classification. Despite its computational efficiency, effective usage of XGBoost in practice involves finding an optimal subset from a large set of regularisation hyperparameters.^30,31^

To achieve a robust, statistically replicable comparison of TM’s to RF and XGBoost, we follow the guidelines detailed in a study by the Polaris group.^15^ These algorithms were chosen for direct comparison to TM’s due to their similar scope in applicability to QSAR problems. Each method has a unique training scheme, yet they all mine for molecular patterns in chemical space by constructing conditional rules. Furthermore, RF and XGBoost have been shown empirically to be highly accurate in QSAR studies, consistently achieving high prediction metric scores on-par or greater than a range of methods including large, complex NN architectures.^4,9^ To examine the learning capabilities of clauses as a single unit compared to decision trees, the benchmarking procedure detailed here was repeated for N_CLAUSES = 100, 200, 400, 800, 1,600.

### 2.3 Descriptor Calculation

Here we examine the influence of descriptors on TM learning, in comparison to RF and XGBoost, by including Extended Connectivity Fingerprints (ECFP) and physicochemical descriptors calculated from the 2D molecular graph in RDKit (RDKit2D). ECFP, also known as Morgan Fingerprints, are topological fingerprints where a defined length and maximum radius are set.^32^ Circular atom environments are generated by traversing through atom neighborhoods in a circular fashion up to the maximum radius and given a unique identifier. This process is repeated for each atom of the molecule, pruning duplicate substructures which appear in the process. For the fingerprint vector, the hashing process assigns the circular substructure to a bit position in the bit vector of defined length. By increasing the maximum radius, the bit vector becomes more populated with unique substructures (i.e., fingerprint density increases). Here, a fingerprint length of 2048 and maximum radius of 2 is used, generated by the RDKit implementation of a Morgan fingerprint to yield ECFP4 features.

Physicochemical descriptors are those which represent a molecule based on point-values (1D) derived from its formula or structure (e.g., atom counts, atom types). 2D physicochemical descriptors are the continuous point-value properties calculated from the 2D structure of a compound, which have been computed from RDKit in this study. The RDKit 2D descriptor set (RDKit2D) comprises 200 molecular features, relating to structural presence (e.g., fragment frequencies) and drug-likeness (e.g., MolLogP, PSA, NumRings).

In line with previous TM modeling pipelines which use continuous numerical features as input, the RDKit2D descriptors are converted to a binary format using a quantile-based binarizer.^33^ Where, given a resolution *r* (number of bits), a set of *r* quantile thresholds are obtained from the training data. Each threshold corresponds to one bit in the binarized version. During the encoding of a descriptor value, all bits with the corresponding threshold value greater than or equal to the descriptor value is set to 1, and the rest to 0. For this study, *r* was assigned a value of 10 to provide sufficient detail on the continuous descriptor space while not increasing the number of input descriptors of the TM model dramatically past the number of training samples for the selected data sets. To examine the effects of ML method variations in isolation within the same descriptor space TM, RF and XGBoost were all trained on the same discretized set of RDKit2D descriptors.

### 2.4 Splitting Strategies

A 5 × 5 repeated cross-validation (CV) strategy is used to sample the performance distribution. Where fold patterns are decided 5 times (N_OUTER = 5) with consideration of assigned groups (random or scaffold), and 5-fold CV (N_INNER = 5) is performed for each fold pattern (Figure 5b). Repeated fold patterns are generated to account for stochasticity and compare performance distributions across populations of models the different methods produced. At these data set sizes (within 500–100,000 samples), using 5 folds reduces the dependence across CV folds, compared to 10 folds, within a split because the training sets are less likely to overlap. Furthermore, Ash et al. demonstrated empirically that 5 × 5 CV provides a more stable and accurate variance estimate than other methods for improved statistical testing.^15^

**Figure 5:**
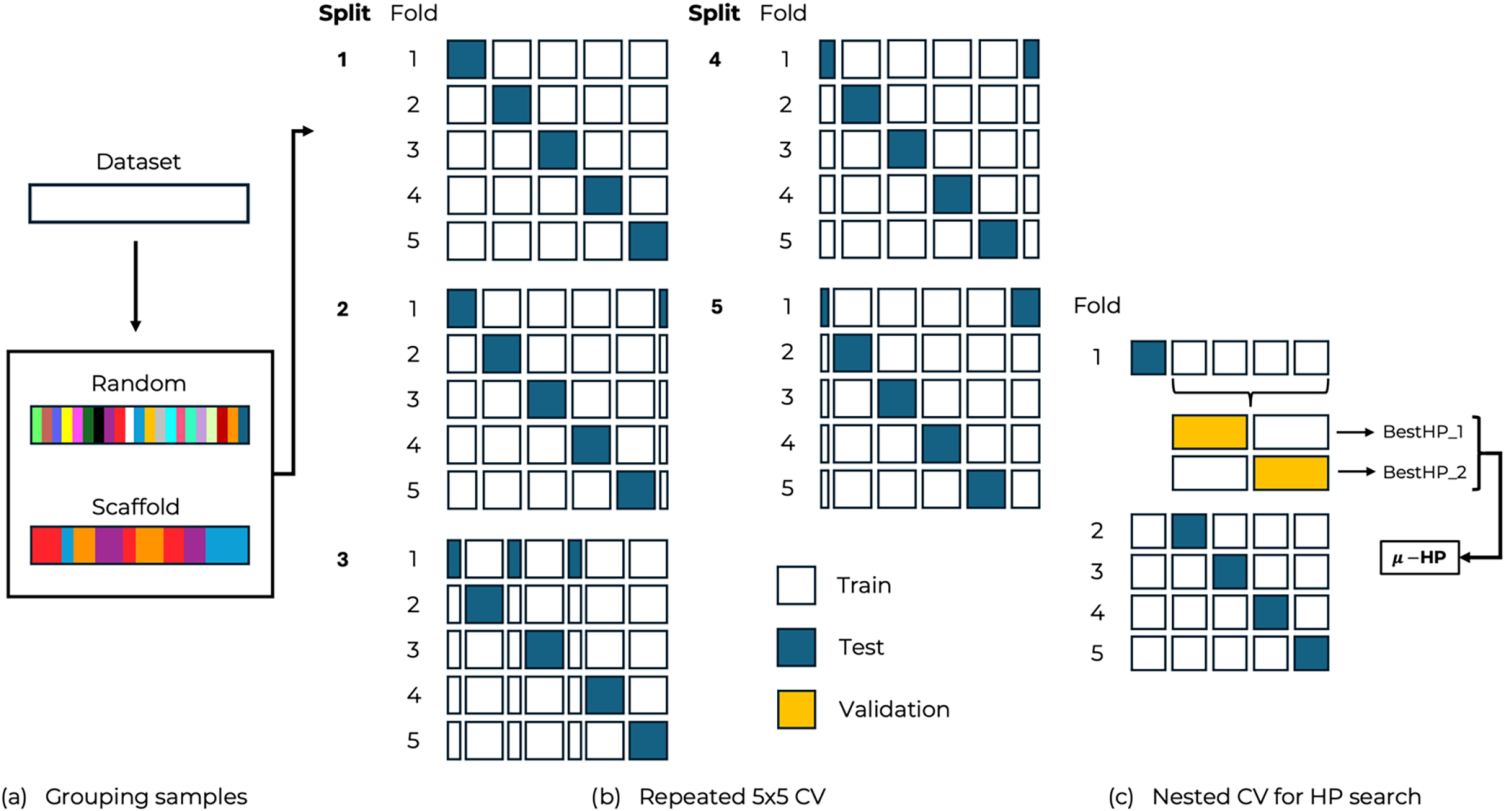
Methods combined to sample performance distributions for this benchmarking study. (a) Grouping of data set samples into random or scaffold-based clusters. (b) Repeated 5 × 5 CV used to generate train and test sets for model building and validation. (c) Nested 2-fold CV used on training sets to choose performant hyperparameters of each ML method. Adapted from Ash et al.^14^ Copyright 2025 American Chemical Society.

#### 2.4.1 Random Grouping

Groups are provided to samples before fold patterns are decided using the useful_rdkit_utils library.^34^ Random grouping of samples into 5 clusters, combined with GroupKFoldShuffle to select a folding pattern is equivalent to Random 5 × 5 repeated CV. It is also equivalent to the index of each sample being random shuffled and the same splits/fold patterns being applied to the resulting reindexed array. For each fold pattern, a different numpy.random instance is initialized.^35^

#### 2.4.2 Scaffold Grouping

The useful_rdkit_utils is also used to cluster samples into Bemis-Murcko scaffolds.^34^ In their seminal paper, Bemis and Murcko defined a framework or scaffold as the union of ring systems and linkers (atoms which connect two ring systems) in a molecule, including their heteroatoms and bond order.^36^ Bemis-Murcko scaffolds were used as a proxy for molecular shape. After molecules are clustered by their scaffold, different fold-patterns are then constructed with the aim of ensuring that samples in the test set have scaffolds unseen during training, thereby contextualising performance metrics into how well they perform when faced with entirely new scaffolds. Deng et al. postulated that this demonstrates the ability of models to consider the phenomenon known as “scaffold cliff”, where two molecules with different scaffolds have disparate properties, and achieve interscaffold generalization.^4^ Having different splitting procedures also increases the pluralism of our benchmarking study, providing model performance distributions across different and varied environments.

The result for this 5 × 5 CV workflow, combined with initial grouping, is that each sample receives 25 training set metric scores and 25 test set metric scores for all method (TM, RF, XGBoost), descriptor (ECFP4, RDKit2D) and split-group (Random, Scaffold) combinations.

### 2.5 Hyperparameter Search

The hyperparameters (HPs) used by each ML method can have significant influences on overall performance in prediction tasks. Thus, both for the TM and compared models, it is important to provide a HP search procedure which allows each method an equal opportunity to find somewhat optimal HPs within reasonable timeframes. For this purpose, we employ sequential model-based optimization (SMBO) using the open-source library Optuna.^37,38^

Within the 5 × 5 CV procedure, HP search is conducted using nested folding (Figure 5c). During training and inference, 4 folds of the target data set are provided as a training set with 1-fold as a hold-out test set (Figure 5c). Nested folding means that the resulting training set is further split into 2-folds; one acting as the “inner-training” set and the other acting as the “inner-validation” set. The inner-training set is used to build models with sampled HPs from Optuna. The performance of the model and the selected HPs are evaluated on the inner-validation set to provide validation scores which are used to select “best hyperparameters” for the first inner-fold using 25 Optuna trials. The previous inner-training set, and inner-validation set then switch roles and the process is repeated, yielding 2 sets of “best hyperparameters” found for a given training set, of which the average is taken to provide the final hyperparameters for each model achieved over 50 trials. The number of trials was chosen based on preliminary results of how many trials RF and XGBoost ROC-AUC metric scores generally took to converge (15–20) through SMBO in Optuna. This procedure is also in line with other studies which focus on statistical rigor where XGBoost and HP search are used.^9,39^

The inclusion of this hyperparameter search regime means that for each final model built for the comparison of performance distributions (8000 final models), 50 models are used to conduct HP search (400,000 total models). This limited the number of data sets which could be studied in reasonable timeframes to those selected for this study.

### 2.6 Evaluation Metrics

For a given training set, optimal HP set and test set, TM, RF and XGBoost models are then trained sequentially on the same training set and evaluated on the same hold-out test set. The metrics of evaluation are as follows:

#### 2.6.1 Collective Metrics

The receiver operating characteristic curve (ROC) is obtained by plotting the true positive rates (TPR) and false positive rates (FPR) across different probability thresholds with its area under the curve as ROC AUC. ROC AUC ranges from 0 to 1, where a classifier performing worse than random gives a ROC AUC of less than 0.5. Similarly, using precision and recall across different probability thresholds, the precision-recall curve, area under the curve (PRC AUC) can be calculated. PRC AUC is more suitable when the minor active class is of greater interest, where the baseline value is the fraction of the minor class.

#### 2.6.2 Virtual Screening Metrics

The goal of drug discovery is to rank molecules based on predicted activity, avoiding false positives or false negatives. Collective metrics give a general understanding of how ML models perform over all predictions and may not provide an indication as to how well they rank molecules. Positive predictive value (PPV) and negative predictive value (NPV) place greater emphasis on the practical ranking task desired of QSAR modeling by looking at the purity of TPs and TNs above a high predictive threshold chosen by Youden’s J statistic. Youden’s J statistic is the maximum distance between the ROC curve and a random chance line where the idea is to maximize the difference between true positive rate and false positive rate (eq 3). PPV (eq 4) and NPV (eq 5) are essentially the true positive rate and true negative rate above this threshold, where the focus is narrowed on high-ranked molecules. These metrics provide more practical relevance to TM predictive performance in the case of drug design.

5 $$J=\frac{\mathrm{TP}}{\mathrm{TP}+\mathrm{FN}}+\frac{\mathrm{TN}}{\mathrm{TN}+\mathrm{FP}}-1$$

6 $$\mathrm{PPV}=\frac{\mathrm{TP}}{\mathrm{TP}+\mathrm{FP}}$$

7 $$\mathrm{NPV}=\frac{\mathrm{TN}}{\mathrm{TN}+\mathrm{FN}}$$

### 2.7 Post-Hoc Analysis with Tukey’s HSD

This benchmarking procedure aims to establish the difference in performance of the TM to existing QSAR methods and to understand if these differences are also statistically significant. To that end, model performance distributions are compared to one another using repeated measures ANOVA followed by the Tukey HSD test. Here, the null hypothesis is that two populations of sample (train, test) metric values come from distributions having the same mean values. Tukey’s HSD determines if there is a statistically significant difference in the mean value of model performance distributions, where “performance” is defined as the metric scores outlined above. This parametric workflow provides a pairwise comparison for the means of performance distributions for each model. Aside from determining statistical significance (i.e., assess if the means of the distributions are the same), this method also determines effect size (i.e., the magnitude of the difference in mean between distributions). When conducting this pairwise comparison of means for different model performance distributions, we use Cohen’s D standardized difference of means, where the difference in means of two performance distributions is standardized by the pooled standard deviation (eq 6) which considers the variance in both distributions.

8 $$d=\frac{\mu_{1}-\mu_{2}}{\sqrt{\frac{\sigma_{1}^{2}+\sigma_{2}^{2}}{2}}}$$

Results of this pairwise comparison are summarized in a Multiple Comparison Similarity Plot (MCSPlot) proposed by Ash et al.^14^ The Cohen’s D standardized difference of means is represented numerically (difference value) and in color (positive difference → red, negative difference → blue). Asterix (*) symbols represent the degree of statistical significance (thresholded by *p*–value) to which the difference in means for two model performance distributions is considered not the same under Tukey’s HSD procedure (* = *p* < 0.05, ** = *p* < 0.01, *** = *p* < 0.001).

### 2.8 Model Performance Definitions

We propose the TM as a comparatively accurate, unexplored QSAR method, with potentially useful drug design applications. To avoid reporting overly optimiztic results in favor of the TM and to clearly define what meant by “comparatively accurate” we have implemented a classification protocol which categorizes TM performances in relation to the other methods (Figure 6). The TM is termed as “Better” than existing rule-based QSAR methods for a given metric if its mean value is greater than (positive Cohen’s D value) *both* RF and XGBoost, and this difference is statistically significant for *both* methods with a *p-value* of *at least* 0.05. The TM is termed as “Worse” than existing rule-based QSAR methods for a given metric if its mean value is less than (negative Cohen’s D value) *either* RF or XGBoost, and this difference is statistically significant for said method with a *p*-value of *at least* 0.05. In all other cases, where TM models do not have a higher metric mean value (e.g., metric means are the same) than *both* compared methods, or the difference in mean value is not statistically significant to a degree where *p* < 0.05 for either RF or XGBoost, the TM is considered “Equivalent” to existing rule-based QSAR methods. This probabilistic handicap allows for more ways that the TM can be classed as “Equivalent” or “Worse” than “Better” to established QSAR algorithms, offsetting unintentional experimenter bias and mitigating overly optimiztic reporting of the TM’s performance distributions. Furthermore, this classification method allows us to summarize our results succinctly across targets, metrics, splitting strategies and descriptors by counting their frequency for each experimental condition to give an estimated likelihood when faced with an unknown target. This is termed Performance Count.

**Figure 6:**
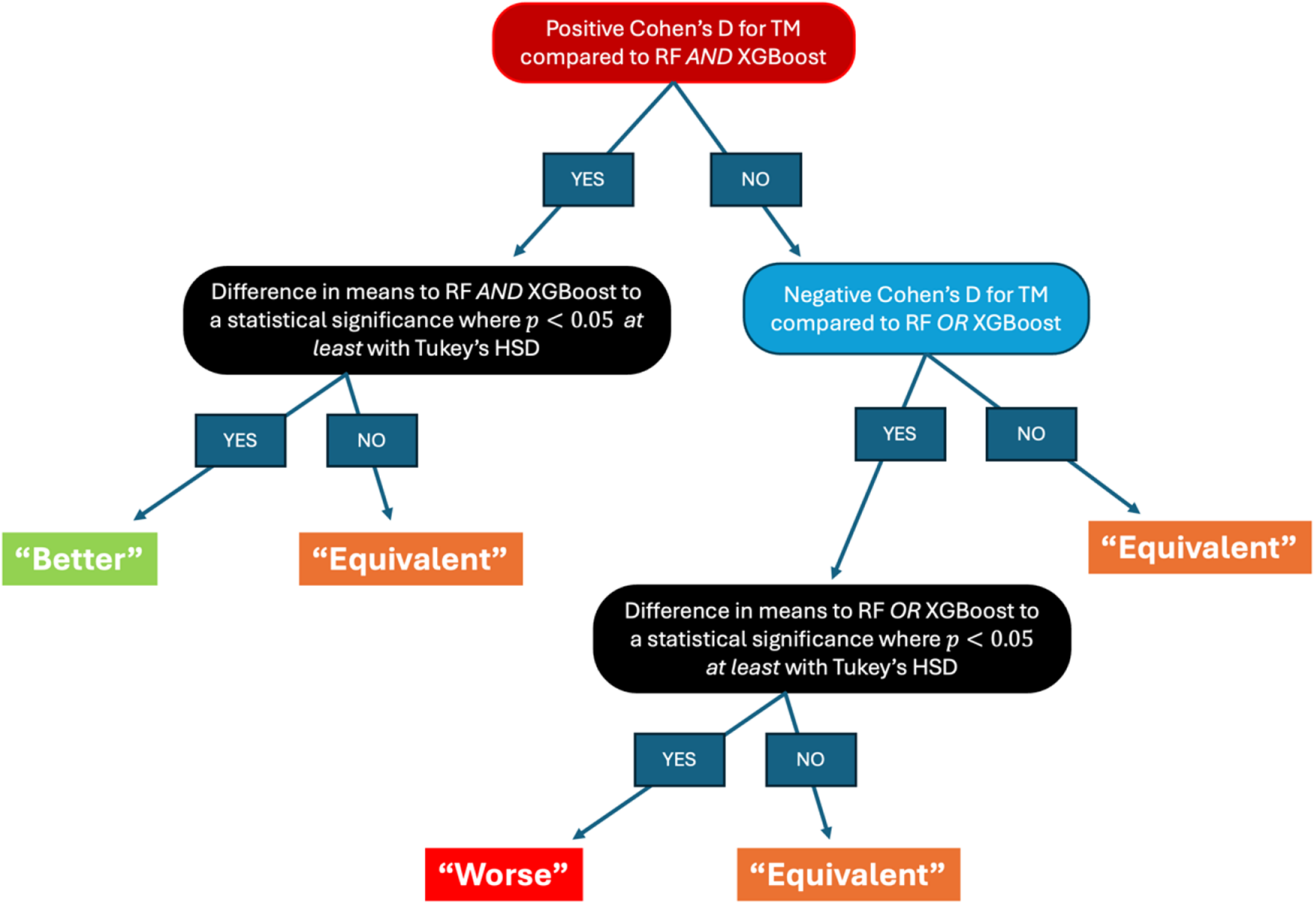
Decision tree used to label TM performance distributions for a given metric “Better”, “Equivalent” or “Worse” than established rule-based QSAR methods. This is repeated for each target, split group and descriptor set combination used.

### 2.9 Interpretability

Using the target case of MOR, ECFP4 descriptors and an 800 clause TM, we demonstrate two methods for TM-QSAR interpretability using RDKit calculated fingerprints. These TM interpretations are then compared to known established theory of ligand interactions with MOR for morphine, fentanyl, BU72 and SR17018 in order to understand if TM prediction importances of fragments are correlated to known ligand binding interactions.^40,41^

#### 2.9.1 TM Molecule Property Maps (TM-MPM)

For a given molecule, clauses are activated as conditional substructure matches considering what substructures it should (*Included* Positive Substructures) and should not (*Included* Negative Substructures) with respect to a predicted class. Each clause also has a certain polarity weight/vote (e.g., +1 *Active*, −1 *Inactive*). The net contribution of all activated clauses for a molecule can be visualized via TM molecule property maps (TM-MPM), where clause weights are accumulated onto atoms of substructure matches. The resulting atom weights for a given clause are decided via the following confusion matrix (Figure 7). Thus, fragments of the molecule contributing to a given class prediction can be visualized.

**Figure 7:**
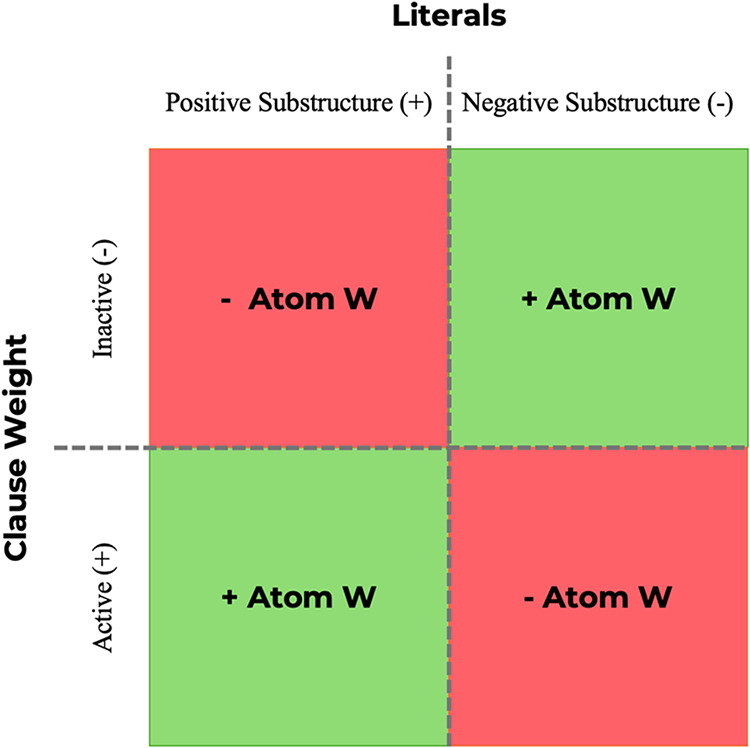
Assignment of atom weights to molecular substructures based on clause weight and whether the structure is positive or negative.

To visualize Negative Substructures of activated clauses for a molecule, training set example molecules with a high prevalence of such fragments are used as examples, *Negative Substructure Examples*.

#### 2.9.2 Closed-Form Substructure Prediction Strength

Closed-form interpretations assign a value of prediction importance to descriptor literals, in this case ECFP4 calculated substructures and their negations, across many predictions. Here the literal importance vector of activated clauses, *WAC* score is defined in eq 7 where the *Include* value of a literal (0 or 1) is multiplied by the associated clause weight and summated across activated clauses. A global interpretation of descriptor importance can be achieved using all predictions of the training or test set. Local feature importances are extracted by using the activated clauses of a chosen subset of molecules.

Here we present the global closed-form interpretation of all molecules across the test-set along with the local closed-form interpretation of the TM-QSAR top 10 ranked test-set molecules. These molecules were chosen as a region of interest (i.e., high predicted bioactivity) in chemical space, but local closed-form interpretations can be conducted for any subset of molecules.

9 $$\mathrm{WAC}={\left(w⊙a\right)}^{T}C=\sum_{i=1}^{k}w_{i}a_{i}c_{i}$$

where *a* is a binary vector indicating clauses are activated for each sample, *w* is the corresponding weight vector, *C* is a binary matrix of the learned clauses (*Included* literals) where *c*_i_ is a single clause. This is completed for *k* activated clauses of a molecule set. For simplicity the resulting score for a single substructural literal is termed the WAC score (**W**eights × **A**ctivations × **C**lauses).

## 3 Results and Discussion

Across all targets, splitting strategies, descriptor calculations, number of clauses and epochs of iterative learning (in the case of the TM), metric performances for the training and test set are collected and freely available. Raw CSV files can be found on the project GitHub^42^ and the Supporting Information contains Metric Score Vs Epoch and MCSPlot for each target, splitting strategy and descriptor combination.

### 3.1 Overall Performance Comparison

A summary table (Table 2) of metric scores across all experimental conditions outlined in the methods section, for all models is provided below. The table summary uses a TM of 800 Clauses with Random Forest and XGBoost models of 100 trees. This imbalance is due to a single Clause being a conditional rule in a rule-list model, where there is no hierarchical element to the components of the rule and are built locally for specific patterns of specific sample objects. Thereby, it is assumed that to cover the same area of chemical space as RF and XGBoost do with entropy splitting, more clauses as rules are needed. For all reported results, we demonstrate metric scores for the TM which correspond to learning at 20 epochs. This was chosen using the training set metric scores as the point where TM learning begins to plateau.

**Table 2:**
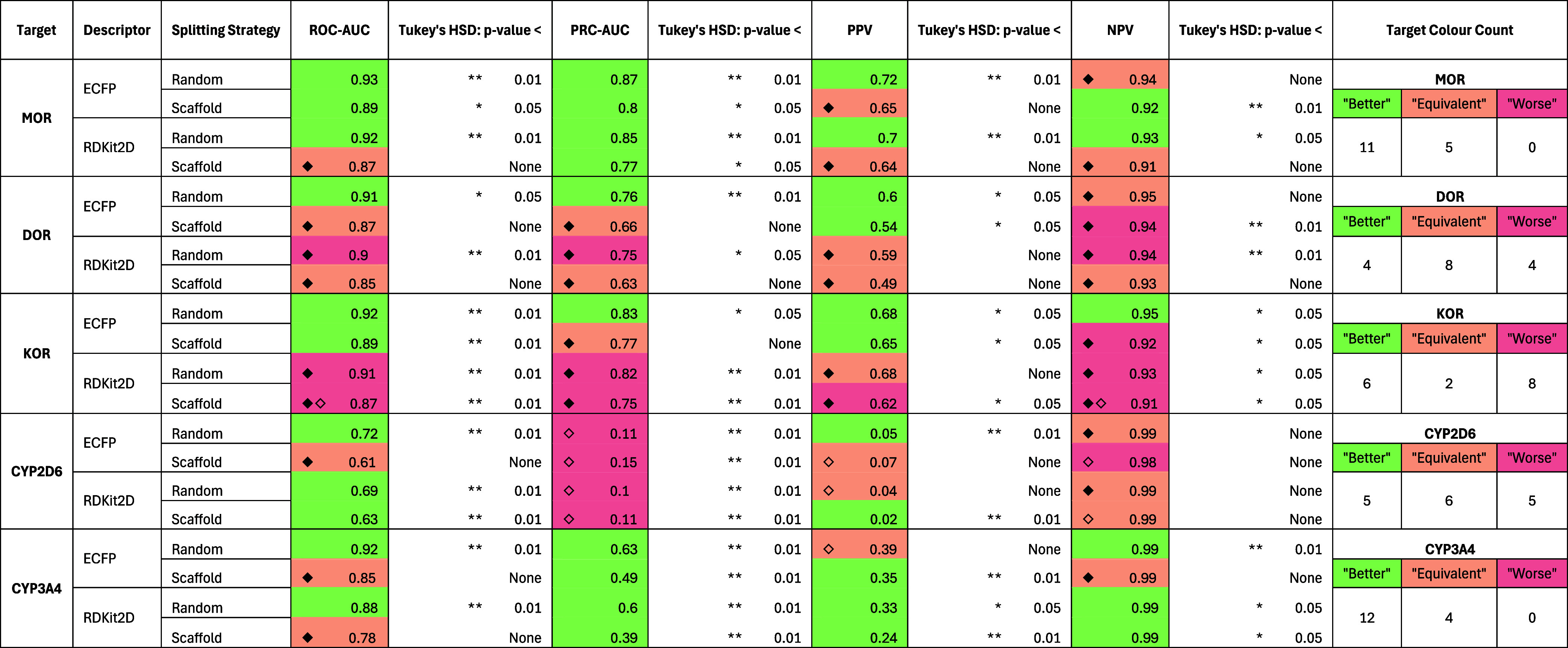
Summary of TM Test-Set Metric Scores across All Experimental Conditions of Target, Descriptor, Splitting Strategy (Group)^a^

a TM metric scores are annotated by Colour where Green = “Better”, Orange = “Equivalent” or Red = “Worse” than existing rule-based QSAR methods. Performance count of each target and metric type is provided on the margins where the frequency of these categorisations is recorded. The column “Tukey’s HSD: p-value < ” details the thresholds that *p*–value is less than with Tukey’s HSD post-hoc statistical tests to the nearest comparable model (RF symbol = ◊ or XGBoost = ⧫) as measured by the smallest Cohen’s D difference of means.

Generalizing for metric scores themselves across all targets, descriptors and splitting methods, Performance Count reveals that the TM frequently achieves better scores than existing rule-based QSAR methods for ROC-AUC (55% of the time), PRC-AUC (50%) and PPV (50% of the time). This is at the relative cost to NPV where TMs are likely to perform equivalent or worse, however mean NPV scores remain high (≥0.90). Thus, while still being able to recognize *Inactive* molecules, TM-QSAR appears capable of achieving better overall prediction performances across all test-set molecules (frequently better ROC-AUC scores), while handling the imbalanced nature of QSAR tasks (frequently better PRC-AUC scores) and keeping the ranked *Active* molecules as free from FPs as possible (frequently better PPV scores). In short, compared to RF and XGBoost, TM-learning has been shown to specialize in early enrichment, an important trait of QSAR methods when used for virtual screening (VS).

Viewing the results summary table target-wise using Performance Count, across all metrics and experimental conditions, TM-QSAR frequently performs better than RF and XGBoost for MOR (69%) and CYP3A4 (75%). Furthermore, compared to existing rule-based QSAR, TM sits at the Pareto frontier of metric scores for each descriptor and splitting strategy. TM-QSAR for MOR and CYP3A4 represent a novel potential SOTA approach to rule-based pattern recognition. This study has enabled us to flag these targets for future regression-TM^43^ and uncertainty^33,44^ studies which are outside the scope of this benchmarking study. TM-QSAR performs most frequently equivalent to existing QSAR methods for the DOR target (50%) and worse for the KOR target (50%), however TM-QSAR represents the Pareto frontier of ROC-AUC, PRC-AUC and PPV model performance for these targets when ECFP4 descriptors are used. Furthermore, in the case of KOR the frequent drop in performance of TM-QSAR was in response to using RDKit2D descriptors.CYP2D6 represents an outlier case in performance whereby RF achieves high PRC-AUC scores, at the cost of all other metric scores, for each descriptor set and splitting strategy, obscuring generalizations that can be made by Performance Count alone. Despite this, MCSPlots (SI Figures 104, 110, 116 and 122) reveal that TM-QSAR shows the highest all-round mean metric performance, above that of XGBoost. However, CYP2D6 represents the most imbalanced (1.4%) and challenging data set studied here and it is worth noting that Shende *et*. *al* have recognized TM sensitivity to class imbalance in multioutput image classification and demonstrated that TMs respond well to simple under sampling regime.^45^ Beyond under sampling, they indicate that tackling the issue of imbalance learning in TMs is underway for future implementations. Combining generalizations from metric-wise and target-wise Performance Count allows us to postulate what may happen for an unknown target in other pipelines when utilizing TM-QSAR. The results suggest that when faced with an unknown target, TM-QSAR has a high likelihood of performing better than RF and XGBoost by improving on ROC-AUC, PRC-AUC and critically PPV, providing a significant basis for TMs to be further explored as a standard method in any drug-discovery and VS toolkit.

Breaking down results by descriptor set using Performance Count indicates optimal molecular representations used to achieve SOTA TM-QSAR models. ECFP4 descriptors combined with TM learning more frequently performs better (Better -23, Equivalent -12, Worse -0) than using RDKit2D descriptors (Better -15, Equivalent -13, Worse -12) across all targets and metric scores. Furthermore, overall mean metric scores were higher for TM models trained on ECFP4 descriptors. This may be due to the presence/absence substructural nature of ECFP4 descriptors being particularly suited to the boolean, propositional-logic based learning mechanism of TMs or that the quantile-based method of binarisation of RDKit2D descriptors could be improved. What is clear, is that the chosen method of molecular representation largely influences the performance of TM-QSAR, similarly to all QSAR methods, and there is scope to improve these performances through the representation of molecules, by exploring a broader range of out-of-the-box descriptors,^46^ or developing custom TM-specific descriptors for particular tasks.

Performance Count alone would indicate that splitting strategies are an equalizer between QSAR methods across all descriptors, targets and metric score types. TM-QSAR with scaffold splitting was more frequently classified as equivalent (Better -14, Equivalent −16, Worse -8) to existing rule-based QSAR methods, with lower mean metric scores across all methods, than random splitting (Better -24, Equivalent -8, Worse -8). However, this issue for TM-QSAR specifically was compounded with the use of RDKit2D descriptors. When considering ECFP4 descriptors alone, TM-QSAR was at the pareto frontier of ROC-AUC, PRC-AUC and PPV across all targets when using a scaffold-split strategy, for all targets except CYP2D6. Therefore, ECFP4 descriptors, combined with TMs may be better at addressing “scaffold cliffs” and achieve interscaffold generalization within substructural descriptor space, compared to existing rule-based QSAR methods. A TM model which recognizes patterns of functionalization in activity, which are robust to changes in central scaffold structures, may prove useful for scaffold-hopping applications.

To update previous generalizations made of comparative TM-QSAR performances when faced with an unknown target case: TM-QSAR has a high likelihood of performing better than RF and XGBoost by improving on ROC-AUC, PRC-AUC and critically PPV, with a high capacity for interscaffold generalization, when substructural fingerprint methods are used as descriptors. This extends TM capabilities in drug-discovery to scaffold-hoping applications and provides a basis for a broader range of molecular representations (both existing and custom) to be explored with TM-QSAR.

These generalizations are made from a robust, statistically reproducible method comparison protocol with similar existing QSAR-ML methods RF and XGBoost. For added context and completeness, we indirectly compare TM results to a subsample of SOTA Neural Networks in Table 3. Our own mean metric values for 800-clause TMs of the repeated 5 × 5 CV procedure are compared to the mean metric values presented for each NN in the independent study of Deng et al.^4^ Given that metric values are not derived from the same train-test splits, we refer to this table as “NN Context” instead of a direct comparison. Despite this, for every target and metric type, 800-clause TMs using ECFP4 fingerprint descriptors, and the more challenging scaffold group-splitting, the mean metric scores are higher than that of the NN Context. Thus, notwithstanding isolated performance gaps relative to rule-based methods, TMs demonstrate robust performance when compared to that of large-scale NN QSAR architectures.

**Table 3:** Neural Network Context Provides an Estimate of TM’s Performance Relative to RNNs (MolBERT) and GNNs (GROVER, GROVER_RDKit)^a^

	ROC-AUC				PRC-AUC				PPV				NPV			
target	TM	MolBERT	GROVER	GROVER_RDKit	TM	MolBERT	GROVER	GROVER_RDKit	TM	MolBERT	GROVER	GROVER_RDKit	TM	MolBERT	GROVER	GROVER_RDKit
**MOR**	0.87	0.85	0.82	0.84	**0.77**	0.69	0.67	0.69	**0.64**	0.63	0.58	0.62	**0.91**	0.90	0.90	0.90
**DOR**	0.88	0.80	0.69	0.79	**0.67**	0.50	0.39	0.49	**0.54**	0.44	0.38	0.44	**0.95**	0.93	0.90	0.93
**KOR**	**0.89**	0.83	0.83	0.83	**0.77**	0.66	0.66	0.67	**0.65**	0.58	0.60	0.59	**0.93**	0.90	0.90	0.90
**CYP2D6**	**0.61**	0.52	0.44	0.36	**0.15**	0.03	0.02	0.02	**0.07**	0.02	0.01	0.01	**0.98**	0.98	0.98	0.97
**CYP3A4**	**0.85**	0.78	0.65	0.81	**0.49**	0.30	0.10	0.26	**0.35**	0.23	0.05	0.16	**0.99**	0.99	0.98	0.99

a ECFP4 descriptors and scaffold splitting was used to generate average TM results. This is not to be used for direct method comparison as results for the TM are generated via a different workflow.

The following section examines the iterative learning process of TMs with increasing number of clauses (N_Clauses 100, 200, 400, 800, 1,600). Here, we select the MOR and CYP3A4 data sets from the summary table (Table 1) and the same plots can be found for all targets in the Supporting Information.

### 3.2 Analysis of Tsetlin Machine Learning Dynamics

Figures 8 and 9 depict test-set metric scores vs training epochs for TMs of increasing number of clauses for MOR and CYP3A4 data sets respectively. The mean scores for RF and XGBoost are annotated across all epochs for comparison. In the case of MOR (Figure 8), the effect of doubling the number of clauses improves test-set scores with diminishing marginal returns when considering model complexity. Passing the use of 400 clauses, both general and virtual-screening specific metrics show little improvement. Moreover, it takes at least 400 clauses for TM scores to be greater than the median of RF and XGBoost. Consequently, while TMs can achieve superior results by using a higher number of rules, there is a limit to this scalability. Once the TM score reaches a performance plateau and overtakes that of alternative rule-based methods, the impact of increasing the clause number diminishes, as the information available in the descriptor space becomes covered by the rule set.

**Figure 8:**
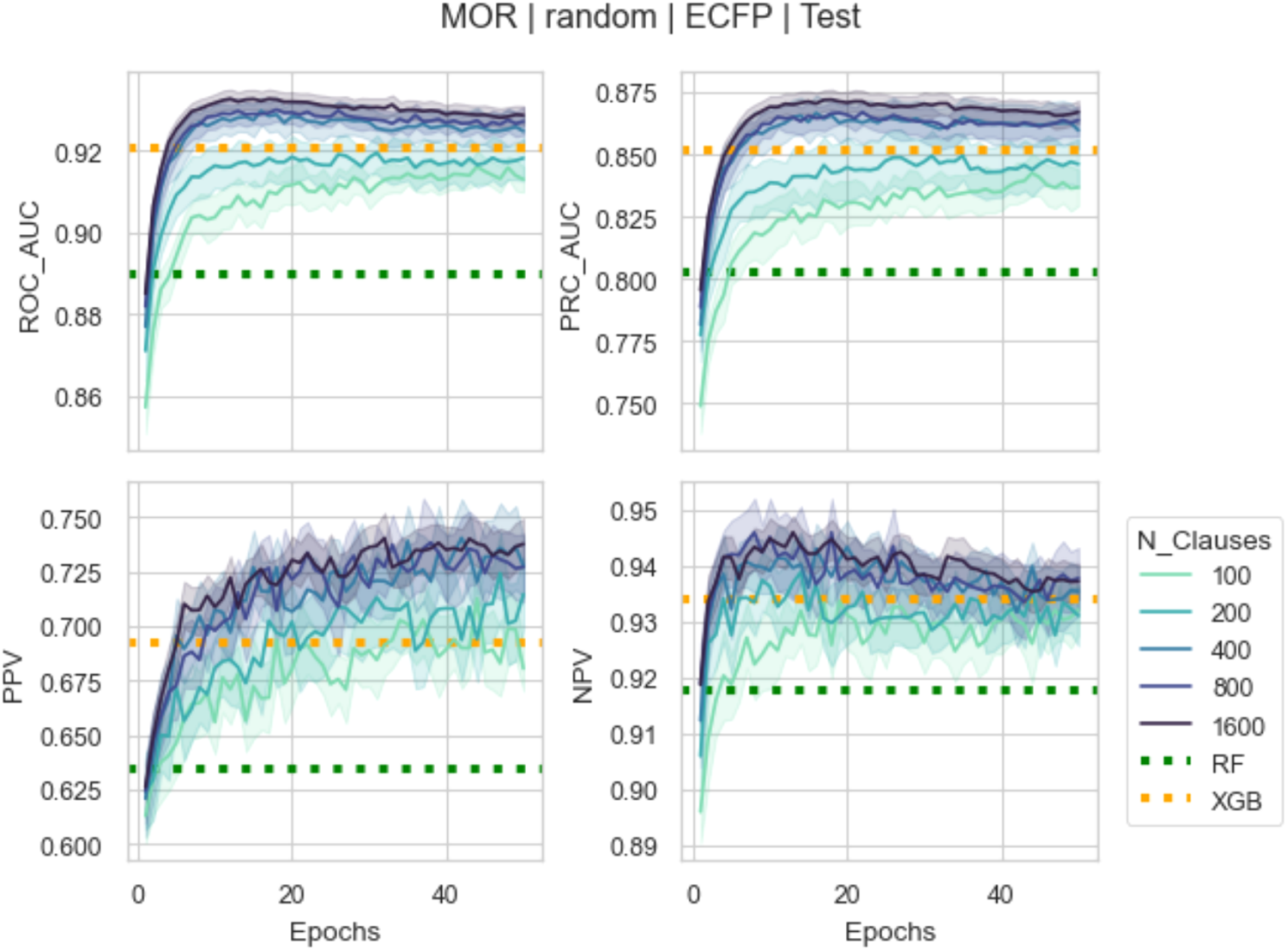
TM classification metrics for the test set samples over 50 epochs for the MOR data set. The median metric score for RF and XGBoost are annotated for comparison.

**Figure 9:**
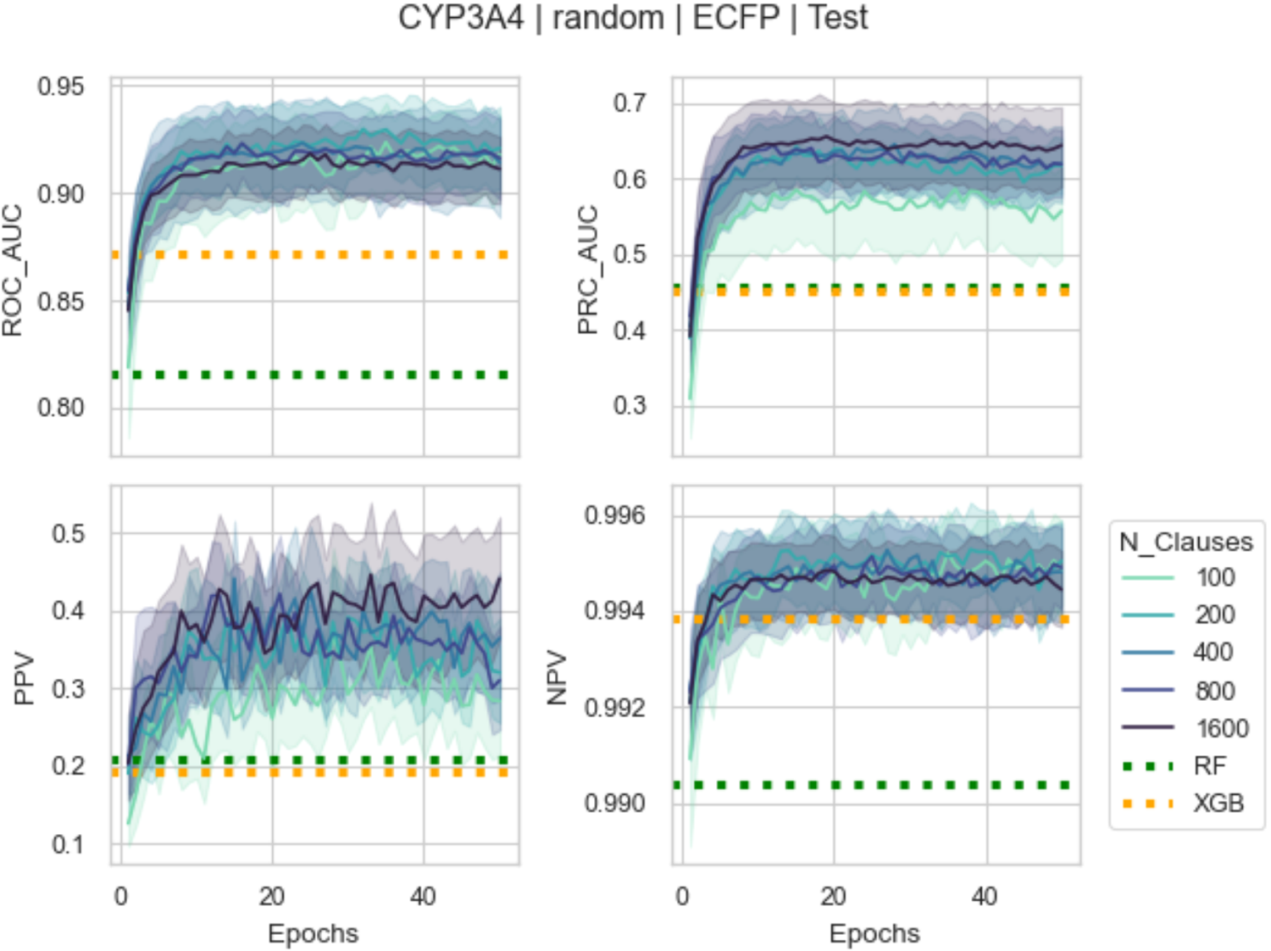
TM classification metrics for the test set samples over 50 epochs for the CYP3A4 data set. The median metric score for RF and XGBoost are annotated for comparison.

The same can be said for the CYP3A4 data set (Figure 8), where just 100 clauses are needed to achieve higher metric scores than RF and XGBoost. Doubling the number clauses then provides little benefit for test-set metric scores. For CYP3A4, the TM seems innately suited to this prediction task over RF and XGBoost. It could be argued that in such cases, small-scale TM models, where modeling is reduced to just a few rules (≤100), could be achieved, making rule-based QSAR model interpretability more facile.

For both MOR and CYP3A4, TM superiority is reached after 20 epochs of training with at least 400 clauses. For metrics of targets where TM superiority can be achieved, this is consistent across the studied set. Therefore, despite the TM’s reinforcement-based nature, SOTA QSAR models which are comparatively better than established rule-based methods and exceed the performances in the context of neural networks, can be achieved in a reasonable number of iterations over reasonable timeframes. Here, the total time for TM hyperparameter search, training and inference of 40 s per train-test split was 1.5× faster than that of XGBoost and 7.4× faster than RF.

### 3.3 Pair-Wise Comparison of Rule Based Methods

Although the above plots demonstrate that higher metric scores for TMs can be achieved, it is necessary to assess “how high” and the statistical significance of such improvements. To this purpose, pairwise comparisons of model metric scores using Tukey’s HSD are provided for MOR (Figure 10) and CYP3A4 (Figure 11) using MCSPlots. We examine the highest performing descriptor (ECFP4) and splitting strategy (random) for all models. In the case of TM models, those with 800 clauses and trained for 20 epochs were chosen for comparison here. The exact same plots for each target, descriptor and splitting strategy combination can be viewed in the Supporting Information.

**Figure 10:**
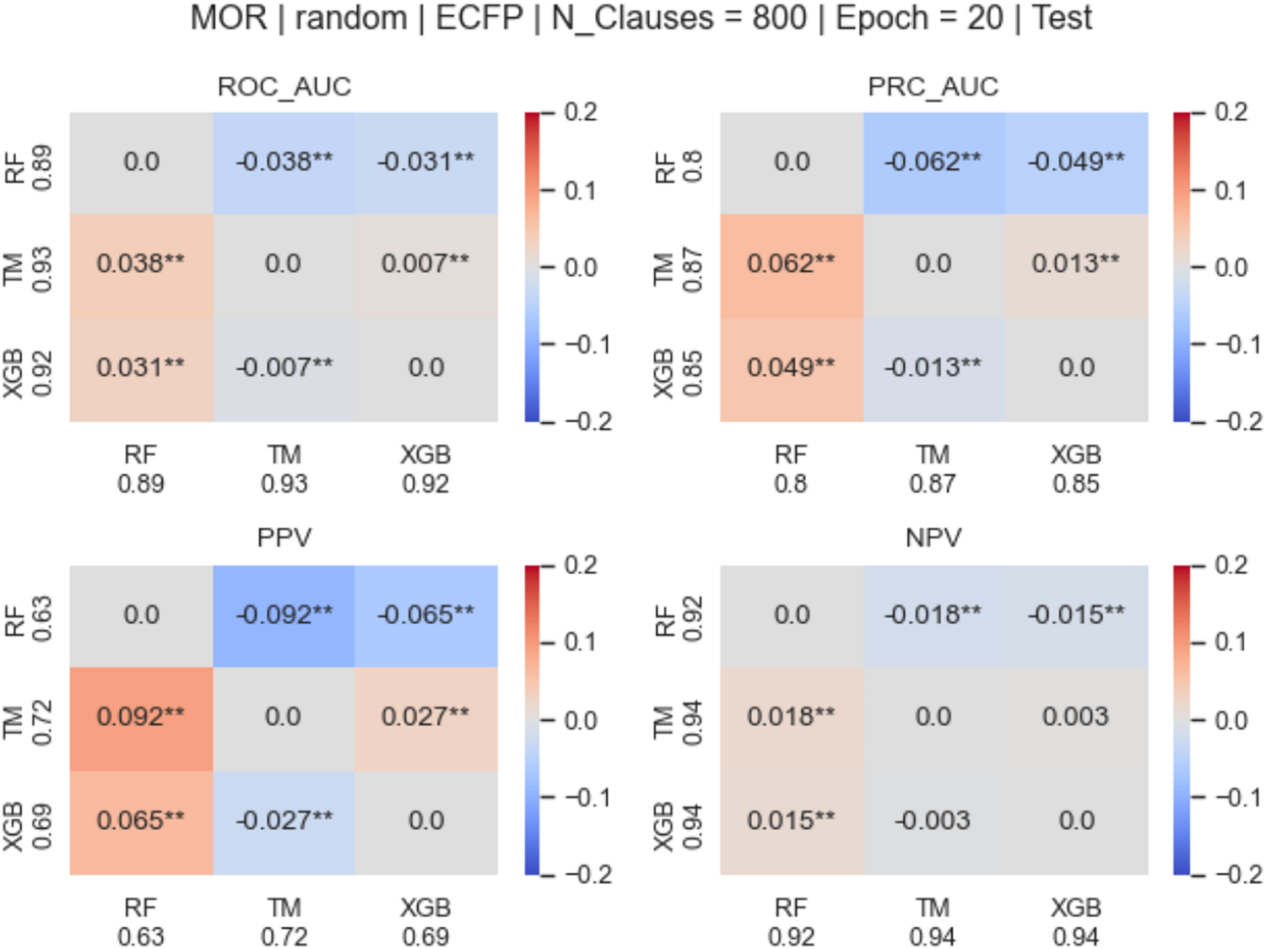
MCS plots visualizing Cohen’s D difference of means, pairwise comparison of models for the MOR data set with random group-split, ECFP4 descriptors and TM models of 800 clauses at 20 epochs. Complete with annotated statistical tests via Tukey’s HSD where the number of asterix represents a different statistical significance level.

**Figure 11:**
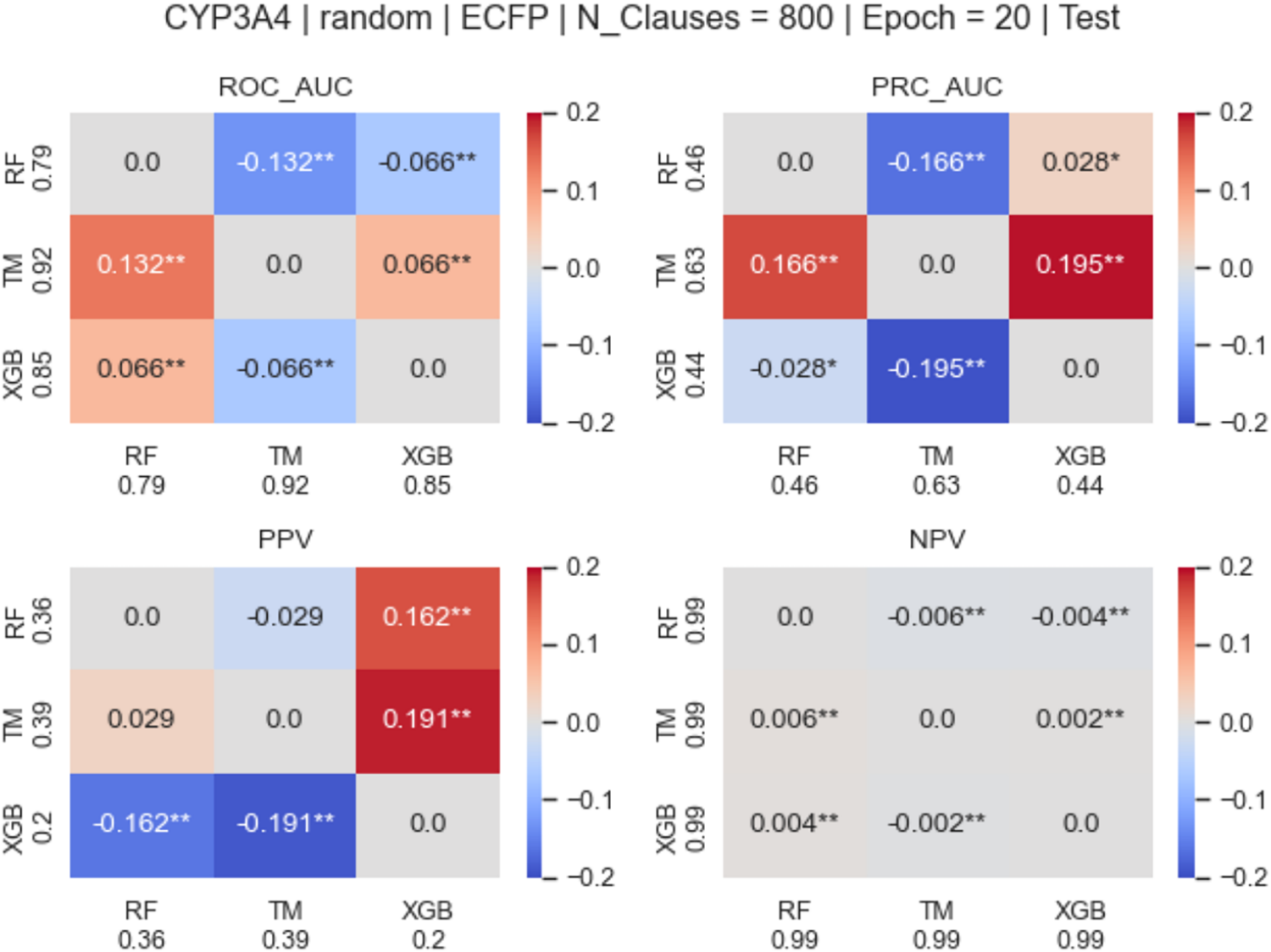
MCS plots visualizing Cohen’s D difference of means, pairwise comparison of models for the CYP3A4 data set with scaffold group-split, ECFP4 descriptors and TM models of 800 clauses at 20 epochs. Complete with annotated statistical tests via Tukey’s HSD where the number of asterix represents a different statistical significance level.

For the MOR data set (Figure 10), generic metrics of ROC-AUC and PRC-AUC are marginally better for TMs than RF and XGBoost. Despite this marginal difference, the difference is statistically significant with *p* < 0.05. There was a much higher degree of difference for PPV, a critical metric for virtual screening. Where a 0.027 difference in means is achieved at a statistical significance where *p* < 0.05, when compared to XGBoost. Although these improvements appear small, they can be impactful in the case of minority active classes. The MOR case represents potential scenarios where TMs can be used to incrementally increase precision of TPs in ranked lists, where XGBoost already performs well. Such cases, where small improvements in precision are crucial, is typically seen in drug discovery, where the cost of testing and obtaining data for potential molecules classified as “active” against any target is expensive and time-consuming.

The differences in model performance are much greater for CYP3A4 (Figure 11). With large, statistically significant differences in mean metric scores for ROC-AUC, PRC-AUC and PPV, when compared to XGBoost. CYP3A4 represents cases where TM PPV is superior to XGBoost but the same as RF, however, overall metric scores for TM are broadly better than RF. This can be generalized to drug discovery scenarios where top-ranked candidates have been tested and failed, and the overall quality of the ranked-list of molecules needs to be improved to conduct a broader exploration of drug candidates.

### 3.4 TM Hyperparameter Search Analysis

The previous sections have outlined that TMs can frequently provide an improvement over rule-based methods in terms of metric scores. Here we examine if these performance improvements are highly dependent on what hyperparameter set is chosen by SMBO, and what values for T and s are appropriate for QSAR modeling tasks. In Figures 12 and 13, the values for T and s chosen by our 2-fold validation procedure (across all N_Clauses) using SMBO in Optuna are plotted against test-set metric scores for the MOR data set.

**Figure 12:**
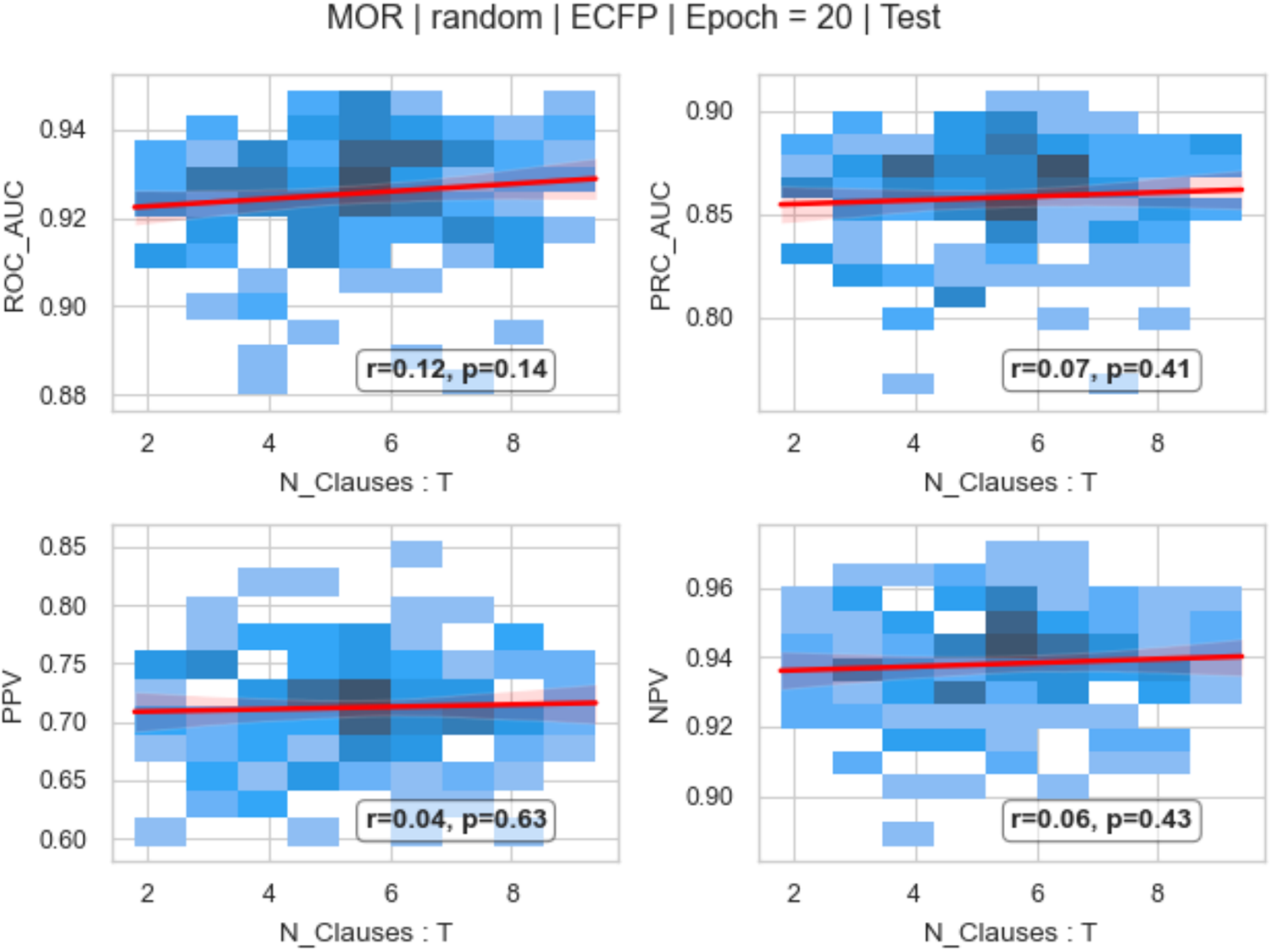
2D histograms of ***N_Clauses: T*** ratio chosen by SMBO versus TM metric scores - with an overlaid fitted regression line. Pearsons Correlation Coefficient (*r*) of the fitted line and associated *p*-value (*p*) are annotated.

**Figure 13:**
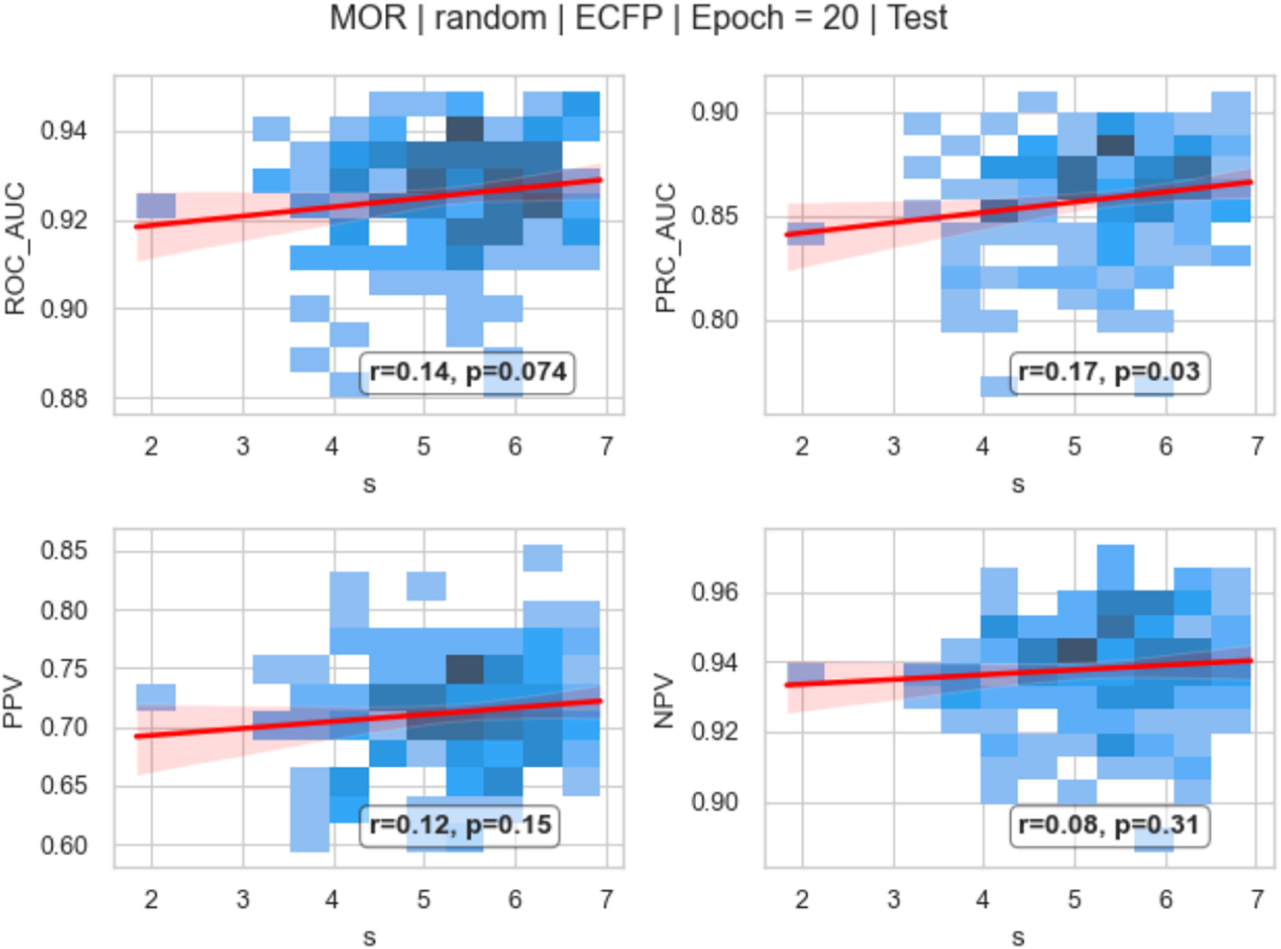
2D histograms of ***s*** chosen by SMBO versus TM metric scores, with an overlaid fitted regression line. Pearsons Correlation Coefficient (*r*) of the fitted line and associated *p*-value (*p*) are annotated.

Based on metric scores achieved on the validation set in 2-fold CV, SMBO tends to pick values of *N_Clauses:T* corresponding to the middle of the range to optimize ROC-AUC for MOR (Figure 12). For example, when using 100 clauses in a model, SMBO most-frequently, will choose a value of T around 500 (*N_Clauses:T = 5*). Despite a validation set indicating such values, there seems to be little correlation between *N_Clauses:T* and test-set metric scores, linear or otherwise (e.g., polynomial order = 2) for fitted curves. This is consistent across targets, splitting strategies and descriptor sets used for TMs in QSAR modeling tasks examined here. These results indicate that so long as reasonable HP ranges are provided and using a fixed number of training epochs, test set metric scores become insensitive to *T* when using TM-QSAR in combination with SMBO in Optuna. This contrasts with results from TMs used in image classification and the use of grid search for HP tuning, where *T* and *s* were shown to have explicit local and global optima for overall TM accuracy.^47^ Thus, future work must be conducted, implementing a similarly systematic approach for QSAR, to determine the sensitivity of metric scores for TM HPs in chemical modeling tasks. In contrast to TM-QSAR, XGBoost and RF test-set metric scores have been shown to sensitive to chosen hyperparameters.^48^

The results seen for *T* of TM-QSAR in combination with SMBO using Optuna extend to *s* also (Figure 13). When looking at *s* in isolation, there is a tendency of SMBO to select relatively high *s* values (around 5 – 7) using a validation set. For test-set metric scores, there seems to be no correlation with *s*. Indicating that when using SMBO to select an appropriate corresponding *T* value both long (low value of *s*) and short clauses (high value of *s*) suffice in chemical modeling tasks for TMs. Thus, the complexity of HP search for TMs can be lowered significantly than that conducted in this study by selecting the number of clauses (i.e., how many rules are required to model your problem) and *s* (i.e., how specific/long rules are required to be) in line with experimenter preferences and conducting SMBO to find an optimal *T* (i.e., how will these rules coordinate with each other). Once again this is more-or-less consistent across all targets, descriptors and splitting strategies. Thus, in contrast to using existing rule-based methods such as RF, the task of hyperparameter search for QSAR objectives is computationally simpler when the number of epochs is fixed. By reducing the computational complexity of such an essential step in QSAR pipelines, TMs may offer competitive SOTA models in relatively faster timeframes.

### 3.5 TM-QSAR Interpretability

As stated previously, clauses serve as logical conditions for *Active* or *Inactive* molecules, which are then used in conjunction with their weights to give prediction contributions of molecular fragments to activity. From here, we demonstrate two methods to directly extract TM-QSAR interpretations in the form of Molecule Property Maps (TM-MPM) and descriptor WAC (**W**eights × **A**ctivations × **C**lauses) scores for the MOR target without the use of external wrapper functions.

To determine if such interpretation functions for TM predicted MOR bioactivity correlate with known chemical theory of binding, we generated the 2D protein–ligand interaction diagrams in Figure 14 using the RDKit for known MOR ligands such as fentanyl, morphine (preactivation), BU72 and SR17018 for result comparison.^40,41^

**Figure 14:**
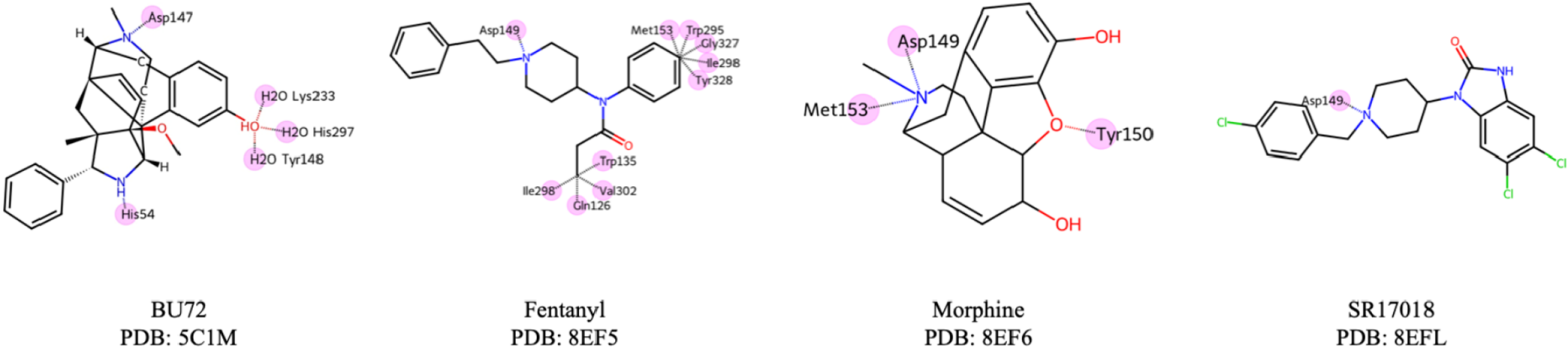
2D protein–ligand interaction diagrams of known MOR ligands as observed via X-ray crystallography.^40,41^ For hydrophobic and aromatic interactions of the n-alinine ring of fentanyl a representative atom was chosen for clear illustration.

#### 3.5.1 TM Molecule Property Maps

Clauses capture logical patterns of Positive and Negative Substructure descriptors which have been precalculated, and clauses can be read as-is with their corresponding logic (i.e., AND, NOT, AND NOT). In Figure 15, example ECFP4 clauses which were activated (i.e., evaluated as True) for all top 5 ranked test-set molecules of the MOR data set are visualized using the RDKit. All of which have a corresponding positive weight in favor of the *Active* class.

**Figure 15:**
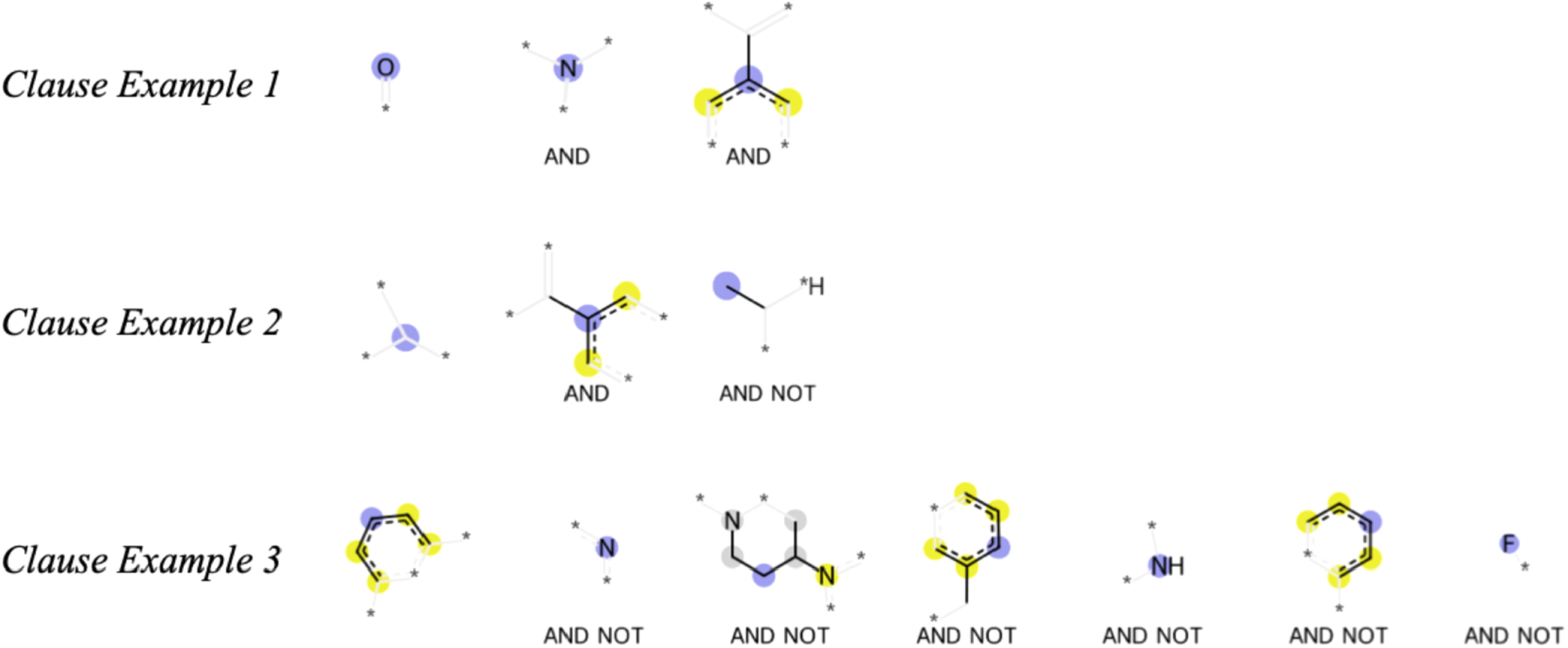
Active clause examples from MOR TM-QSAR model. Clauses were selected as a small subset from the intersection of activated clauses for the top 5 ranked test-set molecules. Atoms highlighted in blue are the central atom of the RDKit ECFP4 fragment, atoms highlighted in gray are members of aliphatic rings and atoms highlighted in yellow are members of aromatic rings.

It might be tempting to examine these clauses and see some correlations to binding interactions of MOR ligands (Figure 14). *Clause Example 1* votes in favor of a molecule being *Active* if it has a tertiary amine, which may be referencing similar ionic interactions with Asp147 of BU72′s morphinan tertiary amine or the salt bridges formed by fentanyl’s piperidine ring and morphine’smoiety with the carboxylate group of Asp149. However, it must be stated that although single clauses may be insightful when it comes to localized predictions, many clauses are activated for a single molecule. For example, the mean number of activated clauses for the top 5 ranked test set molecules was 68, with 155 unique clauses activated across those instances. The activation of many clauses and their conditionality make viewing single clauses to construct generalized rules for predicted bioactivity cumbersome without further condensing/merging of the propositional logic.

To better view the influence of many ECFP4 TM-QSAR clauses for molecules we have generated Molecule Property Maps (TM-MPM). TM-MPMs are formed by iteratively stacking clause weights on atoms matching the Positive Substructures of clauses activated for a molecule. Thus, we can visualize the aggregated contribution of atoms in a molecule to its TM-QSAR prediction. Molecule examples from the training set, exhibiting a high number of Negative Substructures from the same set of activated clauses, can then be used to visualize matching Positive and Negative Substructures (*Negative Substructure Examples*). Such a series of compound maps have been generated for the top 3 ranked test-set molecules in Figure 16.

**Figure 16:**
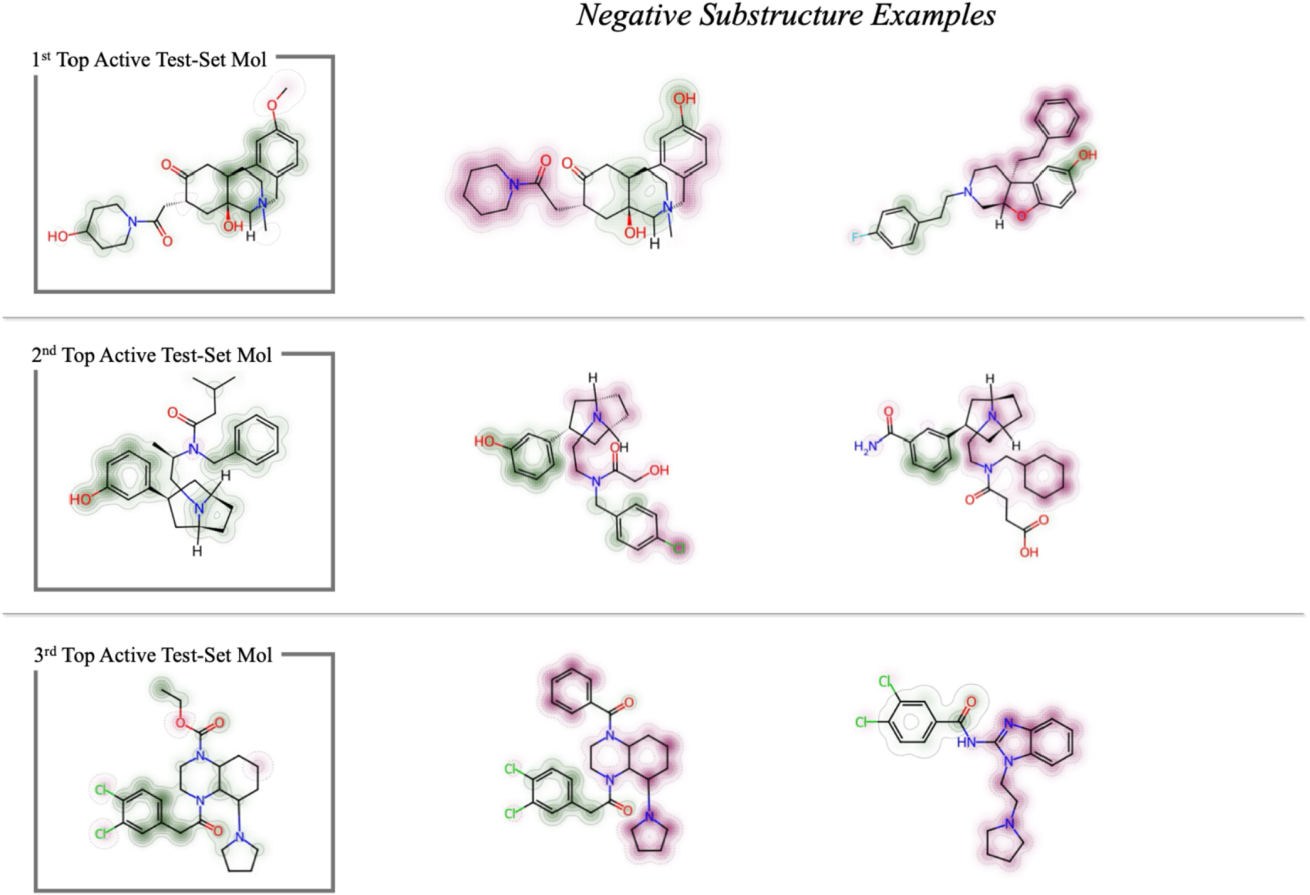
TM Molecule Property Maps of activated clauses for the top 3 ranked test set molecules for MOR. Training set examples where the same activated clauses of each test set molecule are then applied to visualize negative substructures.

Examining the test-set molecules alone, their atom-wise contributions to TM-QSAR predictions of MOR bioactivity correlate to known protein ligand binding interactions. For the first and second top ranked test-set molecule, hydroxy groups contribute positively to its *Active* classification for MOR binding affinity. This could reference the overall frequent presence of hydroxy groups seen in known MOR ligands (Figure 14) or more specifically correlate to specific interactions such as the formation of water networks with Lys233, His297, and Tyr148 seen in binding of BU72 to MOR. The tertiary amines of morphinan groups for the first and second top ranked test-set molecule also contributes positively to the *Active* classification for MOR binding affinity. This is a common moiety in the known MOR ligands depicted in Figure 14 which form salt bridges with the carboxylate group of Asp149 (**D**149). Asp149 then goes on to form polar interactions with nearby residues (**Q**126) and (**Y**328) forming the DQY motif which is crucial for MOR activation.

The ethyl ester group of the third top ranked test set molecule is also observed to have an overall positive contribution to *Active* classification. Due to its proximity to an electron withdrawing group, its importance may correlate to specific hydrophobic interactions such as those of fentanyl’s phenylethyl group with MOR’s hydrophobic minor pocket which includes residues such as Trp135 and Gln126. Furthermore, the chlorine atoms of the third top ranked test set molecule’s dichlorobenzene group have a slight negative contribution to the *Active* classification for MOR binding affinity. This corresponds to established observations by Zhuang *et. al*,^40^ where they noted that although the chloro-benzene moiety SR17018 forms favorable interactions with MOR, it is overall binding affinity is relatively lower to those without it, especially when compared to the potential water networks formed by the hydroxy-benzene group BU72.

The comparison of the test-set molecules and their *Negative Substructure Examples* demonstrate what substructural changes to a molecule can be made to yield higher predicted MOR activity for TM-QSAR. For instance, for the first top ranked test-set molecules the hydroxy-substituted piperidine functional group is seen as contributing positively to MOR TM bioactivity prediction, however without this hydroxyl group, the piperidine is seen as negatively impacting predicted MOR bioactivity in an almost identical structure. Similarly, for the second top ranked test-set molecule, functionalizing the benzene ring with a chlorine at the para position causes the resulting aromatic structure to negatively contribute to predicted MOR bioactivity, although not completely. Finally, for the third top ranked test-set molecule, replacing the ethyl ester group with a benzene ring cause that functional group to contribute negatively to predicted MOR binding affinity. Known theory suggests that having a hydrophobic group of a particular size, attached to an electron withdrawing group, is important for the previously mentioned hydrophobic interactions of ligands with MOR minor binding pocket. The substitution of this group with a larger benzene ring could result in steric clashes.

The examination of TM-MPMs combined with the Negative Substructure Examples from the training set, allow for the suggestion of potential drug design principles. However, it must be highlighted that for all ML interpretability studies using supervised models, resulting interpretations lack ground truth for accuracy validation and they specifically refer to contributions in prediction mechanisms rather than true biological or chemical associations between variables.^49,50^ Despite this, with more rigorous development of TM interpretability methods in combination with unsupervised learning and experimental validation, they may be able to provide possible SAR hypotheses for novel hit molecules in the right context.

#### 3.5.2 Closed-Form Substructure Interpretation

Further TM-QSAR interpretation insights can be found by looking at the relative contributions of ECFP4 descriptors across all clauses. WAC scores for each substructural descriptor of ECFP4 TM-QSAR have been calculated for, the entire test set of MOR to produce a *global* closed-form interpretation of descriptor importances and for the top 10 ranked test set molecules to produce a *local* closed-form interpretation of descriptor importances (Figure 17). Two cases are considered for the global and local interpretations, one where only WAC scores of Positive Substructures are considered (Figure 17a,17c) and another where WAC scores of Positive and Negative Substructure are considered (Figure 17b,d). This allows for the examination of substructures which should be included in a molecule in isolation of those which are deemed to be worth excluding. The proportion where the substructure is present as a Positive Substructure in the calculated WAC score is annotated under the substructure image. Thus, feature importances can be assigned to ECFP4 fragments and their frequent logical form (i.e., Positive or Negative) in TM models can be deduced. To allow for comparisons which are independent of TM clause number and the number of molecules used to compute the WAC values, they are given in *mol* ^–1^ *clause* ^–1^.

**Figure 17:**
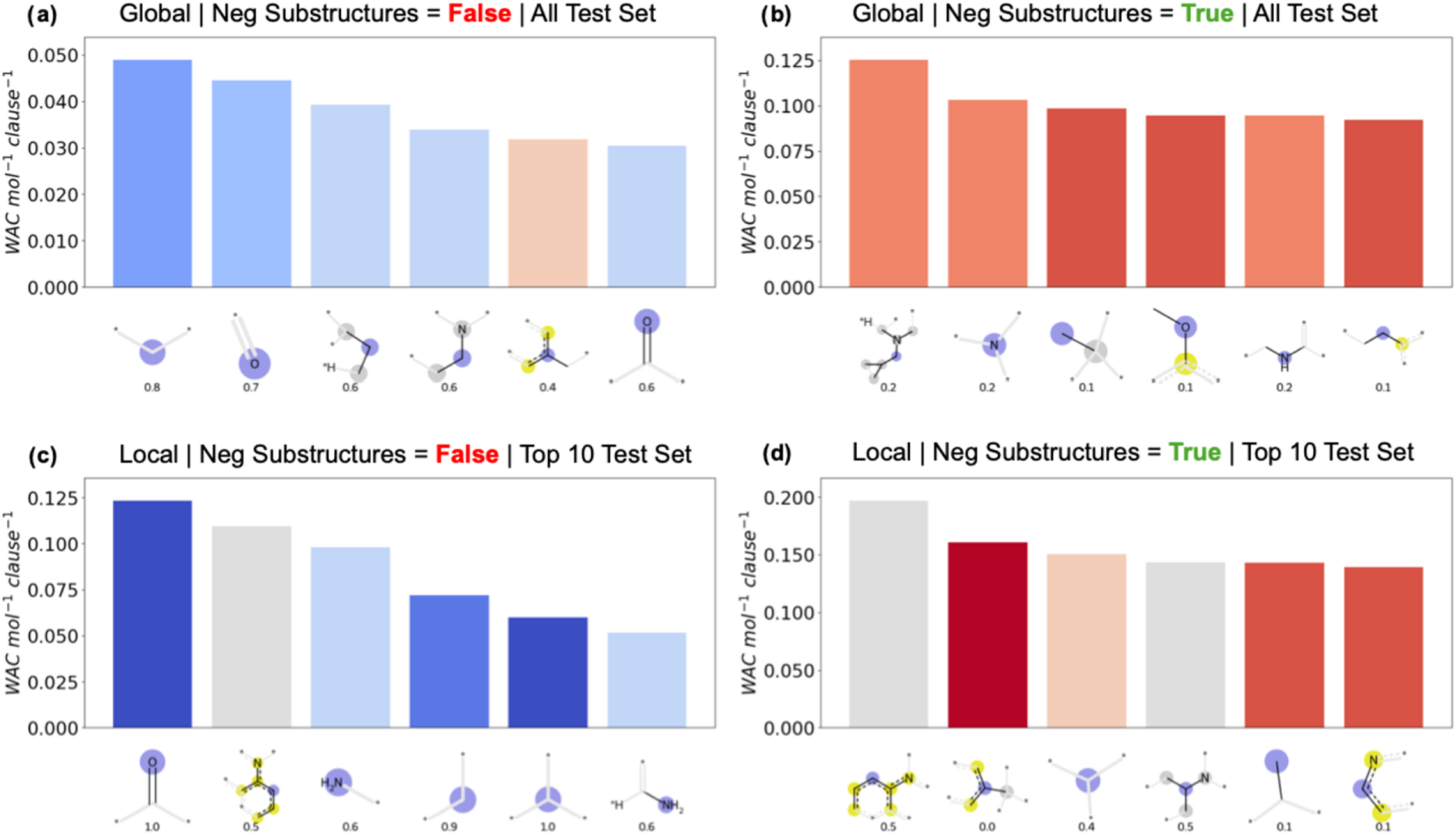
Closed-form interpretability of ECFP4 substructural fragments via WAC. The top 6 (a) Global WAC scores of substructure fragments for the entirety of the test set where negative substructures **are not** considered (Neg Substructures = False). (b) Global WAC scores of substructure fragments for entirety of the test set where negative substructures **are** considered (Neg Substructures = True). (c, d) are the same WAC scores calculated locally for the ranked top 10 test set molecules. The WAC score of each substructure was calculated and normalized by the number subset molecules (mol^–1^) and number of TM clauses (clause^–1^, N_Clauses = 800). The bar colors correspond to the proportion of clauses where the substructure was a Positive Substructure (1.0 → most blue, 0.0 → most red), which is also annotated under each ECFP4 fragment.

The global closed-form interpretation for all test set molecules reveals an emphasis on aliphatic rings, carboxylate moieties and tertiary amines (Figure 17a) for predicted MOR activity. In the case of the tertiary amines, its high WAC score correlates to known binding theory of the DQY motif discussed previously for TM-MPMs. However, there are no direct binding interactions observed via X-ray crystallography which correlate to the high WAC score of carboxylate groups. This may be in reference to a high frequency of indirect involvement of carboxylate groups for molecules in the training set, such as being an electron withdrawing group associated with hydrophobic interactions as in fentanyl. In a similar fashion, TM-QSAR may be able to reveal potential hypotheses for drug design which reflect indirect contributions to target binding or postulate currently unknown interactions.

When considering Negative Substructures (Neg Substructures = True) for the global closed-form interpretation (Figure 17b), WAC scores reveal specific conditions for tertiary amines which are deemed unfavorable to the predicted MOR *Active* class. For example, when not directly part of any ring structure or when connected to a three membered ring, tertiary amines receive high WAC scores as a Negative Substructure. Thereby, WAC scores can give fine-grained details as to what conditional elements are needed for functional groups to *have* and, maybe more critically, *not have* to increase their positive contribution to predicted bioactivity.

Local closed-form interpretations with WAC score can be conducted for any subset of molecules from the training, test or data sets not included in TM model building and evaluation. Here we examine the top 10 ranked test set compounds to view descriptor importances for regions of chemical space with high predicted bioactivity. When considering only Positive Substructures (Figure 17c), carboxylate moieties, terminal primary amines and aromatic ring networks which contain nitrogen exhibit high WAC scores. Interestingly for these aromatic networks, their WAC score increases when Negative Substructures are included in WAC score calculations (Figure 17d). This could indicate that depending on the sampled chemical space and actual clauses (i.e., other parts of the molecule), such ECFP4 fragment features can be deemed as either a Positive or Negative Substructure in chemical space where predicted bioactivity is high. For predicted highly active MOR species, the presence of this fragment is not only deemed as important, but its importance to the *Active* or *Inactive* class depends on careful functionalization and combination with other functional groups. This example illustrates how TM-QSAR can be used to not only develop highly nonlinear models but also derive nonlinear interpretation for predicted bioactivity.

Generated closed-form interpretability plots can be extended to feature any number of ranked substructural descriptors, the top 6 descriptors with highest WAC offer a condensed demonstration. The format of results are similar to feature importance scores of SHAP or LIME, however due to WAC scores being simply computed directly from the TM model itself, the amplification of model biases and the addition of proxy biases are circumvented.^51^

## 4 Conclusions and Future Work

This study establishes the TM as a novel, high-precision, QSAR method with directly accessible interpretability. Despite our classification scheme which places TM at a disadvantage when assigning which method is better for QSAR applications, the results of this study are promising regarding TM metric performances as a novel rule-based QSAR method. While still being able to recognize *Inactive* molecules within mean values of SOTA NNs, TM-QSAR in combination with ECFP4 descriptors is capable of achieving better overall prediction performances than established rule-based QSAR methods across all test-set molecules (frequently better ROC-AUC scores), while handling the imbalanced nature of QSAR tasks (frequently better PRC-AUC scores) and keeping the ranked *Active* molecules as free from FPs as possible (frequently better PPV scores). This places the TM in a promising position in, terms of raw performance to other rule-based QSAR methods for virtual screening applications. Given its computational simplicity, it offers a more environmentally friendly way of screening billions of compounds in ultra large chemical libraries.^24,52^ In essence TM-QSAR may be able to do more with less. Furthermore, when using ECFP4 descriptors, TMs were shown to be better at achieving interscaffold generalization, extending the potential use of TMs to scaffold hopping in drug discovery applications. However, despite the impressive metric scores achieved using substructural fingerprint descriptors (ECFPs), due to the Boolean logic-based learning mechanisms of current TMs, their use remains limited in the case of continuous numerical descriptors as existing rule-based QSAR methods outperformed TM-QSAR in 12 specific instances when discretized RDKit2D descriptors were used. It is worth noting that only two descriptor sets were compared in this study, so future work will involve examining more relevant or improved molecular representations within alternative TM-QSAR pipelines.

Regardless of molecular representation or splitting strategy, SOTA models were achieved via TM-QSAR for MOR and CYPA4, with a statistically significant difference in performance distributions to RF and XGBoost, where *p* < 0.01. RDKit2D descriptors generally led to mixed results across targets, where TM-QSAR performed most frequently equivalent to existing QSAR methods for the DOR target (50%) and worse for the KOR target (50%). This further indicates the importance of molecular representation for TM-QSAR and potentially the need for better binarisation methods for continuous descriptors. CYP2D6 represents the most imbalanced (1.4%) and challenging data set studied here, with the lowest metric performances of TM-QSAR to date. Efforts are being made to tackle the issue of class imbalance with TM learning within our group.

Despite conditional (e.g., RDKit2D, CYP2D6) decreases in TM performances to RF and XGBoost, all versions of 800-clause, TM-QSAR achieved higher mean metric scores than SOTA NNs via indirect comparison. Thereby, the TM has substantial room to optimize for other modeling needs such as size, training time, interpretability and still perform well within the context of SOTA NNs.

The incremental learning of TMs suggests that achieving such SOTA TM-QSAR models require 4x the number of rules than existing decision tree algorithms although some targets may be able to reach high performance results with 100 clauses or fewer (CYP3A4). TM-QSAR test-set results also appear insensitive to *T* and *s* values when using SMBO in Optuna over a fixed number of epochs, thereby in similar conditions, researchers can choose how many clauses they want to define their problem (N_Clauses), how specific they want these rules to be (*s*) and an appropriate *T* will be determined by SMBO which determines how the resulting rules will coordinate to achieve high mean metric scores.

The high performance demonstrated by the TM when ECFP4 descriptors are used may be explained through the locality of TM clauses and its reinforcement-based learning approach. The former point is in reference to the fact that clauses coordinate with each other to extract patterns from different parts of the data. Molecular patterns (active/inactive fragments) within the data were already highlighted in the Veith et al. paper describing the CYP data sets.^17^ This locality in hypothesis space allows for responsibility in test-set predictions to be distributed.^12^ If one collection of clauses, built from one area of the training set performs poorly on predictions for some test set objects, it would not affect predictions for other test-set objects where the clause is False/0. The latter point refers to the mechanism of learning, where TM models are constructed via updating procedures of clauses and TA states instead of entropy splitting.

Beyond its use as an accurate QSAR method the TM can generate additional practical significance by leveraging its incremental learning capabilities and capacity for direct interpretability. In the first case, it is possible to update the TM on the fly and so it can be potentially utilized for continuous or transfer learning.^53^ In the second case, the analysis of TM models themselves may generate potential drug design hypotheses gleamed from data.^54^ The TM is unique in that local interpretations, derived as clauses, are built with one another in mind but are not interconnected and are localized, which can, with features like ECFP4 or structural keys, map onto specific regions of chemical space. Utilizing the RDKit, we have demonstrated that these conditional propositions can be easily read to understand underpinning logical rules which are important for predicted bioactivity for regions of interest (e.g., that of highly active molecules). The hypotheses of clauses derived from data can be further condensed into molecule-wise TM-MPMs and descriptor-wise WAC scores. TM-MPM allows for the examination of atom-level contributions of TM predicted bioactivity for single molecules and WAC scores reveal the importance of single ECFP4 substructural fragments in predicting bioactivity across many molecules. Using MOR TM-QSAR models as a case study, TM-MPMs replicated key aspects of known SAR on this target and revealed potential modifications which could increase predicted bioactivity for test set molecules, while WAC scores indicated the high but nonlinear influence of ECFP4 substructures. TM-QSAR interpretations are extracted directly from the clauses of the TM model, without the use of interpretability wrapper functions such as SHAP, allowing for TM-QSAR to directly illuminate known SAR. The goal is to use clauses to deduce a list of potential candidates, outside of training, test sets and virtual screening of existing databases. Future work will focus on the comparison of TM-QSAR interpretability to other unsupervised and supervised methods to understand the degree to which TM hypotheses used for bioactivity prediction correlate with known chemical theory. Availing of its computational efficiency we will also characterize the TM’s energy efficiency on FPGA or ASIC hardware, quantifying the ‘green AI’ advantages suggested by our computational complexity analysis.

## Supplementary Material



## Data Availability

All data supporting the findings of this study are available within the article and its Supporting Information files. The benchmarking scripts, analysis notebooks, associated pixi environment (.lock and. toml files), results and figures can also be found at https://github.com/PaulC61/TM-QSAR-Benchmark.git.

## Notes

Hyperparameter search grid used for each rule-based QSAR method in SMBO via Optuna; box plots of model metric scores on test set across split group and descriptor pairs; line plots of TM metric scores over 50 epochs for each splitting strategy and descriptor set. pairwise comparison of rule-based QSAR methods using Cohen’s D difference of means for each splitting strategy and descriptor set; number of clauses and metric score histograms for random splitting strategy and ECFP4 descriptor set with annotated Pearson’s *R* and *p*-value. *N_Clauses: T* ratio and test-set metric score histograms for random splitting strategy and ECFP4 descriptor set. Hyperparameter *s* and test-set metric score histograms for random splitting strategy and ECFP4 descriptor set. Plot sequence is repeated for targets MOR (S3 – S27), DOR (S28 – S52), KOR (S53 – S77), CYP3A4 (S78 – S102) and CYP2D6 (S103 – S127) (PDF)
