# Supplementary material for: The Tsetlin Machine: A “Third Way” in QSAR Modeling
Source: J Chem Inf Model. 2026 May 28;66(11):6250–70. doi: 10.1021/acs.jcim.5c03109 (PMC13250913; doi:10.1021/acs.jcim.5c03109)
Supplement: Supplementary file 1 [file ci5c03109_si_001.pdf]

Supporting Information

The Tsetlin Machine: A “Third Way” in QSAR Modelling

Paul F. A. Clarke* [1,2,3], Ivan Cmelo[4], Runar Helin[1], Mayur Kishor Shende[1], Ole-Christoffer Granmo[1], Darren Fayne* [2,3]

[1] Department of Information and Communication Technology, Faculty of Engineering and Science, University of Agder, 4879 Grimstad, Norway
[2] Molecular Design Group, School of Chemical Sciences, Dublin City University, D09 V209 Dublin, Ireland
[3] DCU Life Sciences Institute, Dublin City University, D09 V209 Dublin Ireland
[4] Department of Informatics and Chemistry & CZ-OPENSCREEN: National Infrastructure for Chemical Biology, Faculty of Chemical Technology, University of Chemistry and Technology, 166 28 Prague 6, Czech Republic

* Email: paul.fa.clarke@proton.me, Phone: +47 401 08 537
* Email: darren.p.fayne@dcu.ie

Model	HyperParameter	LowerBound	UpperBound	log	integer
TM	T	1xN_Clauses	10xN_Clauses	False	True
	s	1	7	False	False
RF	max_depth	10	100	False	True
	ccp_alpha	0.001	1.0	True	False
XGBoost	max_depth	5	20	False	True
	learning_rate	0.01	1.0	True	False
	min_child_weight	1	10	True	False
	gamma	0.01	1.0	True	False
	subsample	0.01	1.0	True	False
	colsample_bytree	0.01	1.0	True	False
	colsample_bynode	0.01	1.0	True	False
	reg_alpha	0.001	1.0	True	False
	reg_lambda	0.001	1.0	True	False

Table S1: Hyperparameter grid for each model for SMBO via Optuna

μ - Opioid Receptor

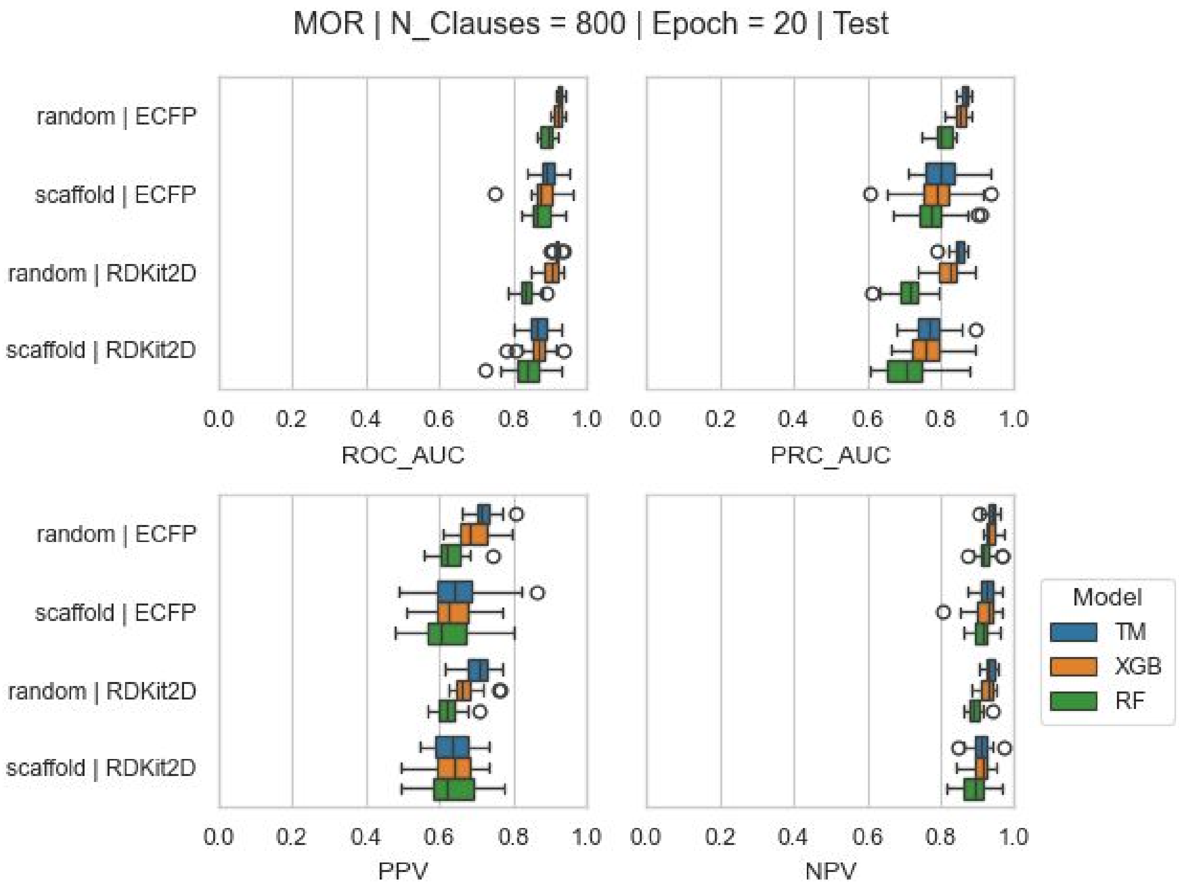

Figure S1: Box plot of model metric scores on a test set across split-group | descriptor pairs for MOR dataset. The TM uses 800 clauses and learning is stopped after 20 epochs.

random split-group | ECFP Descriptors

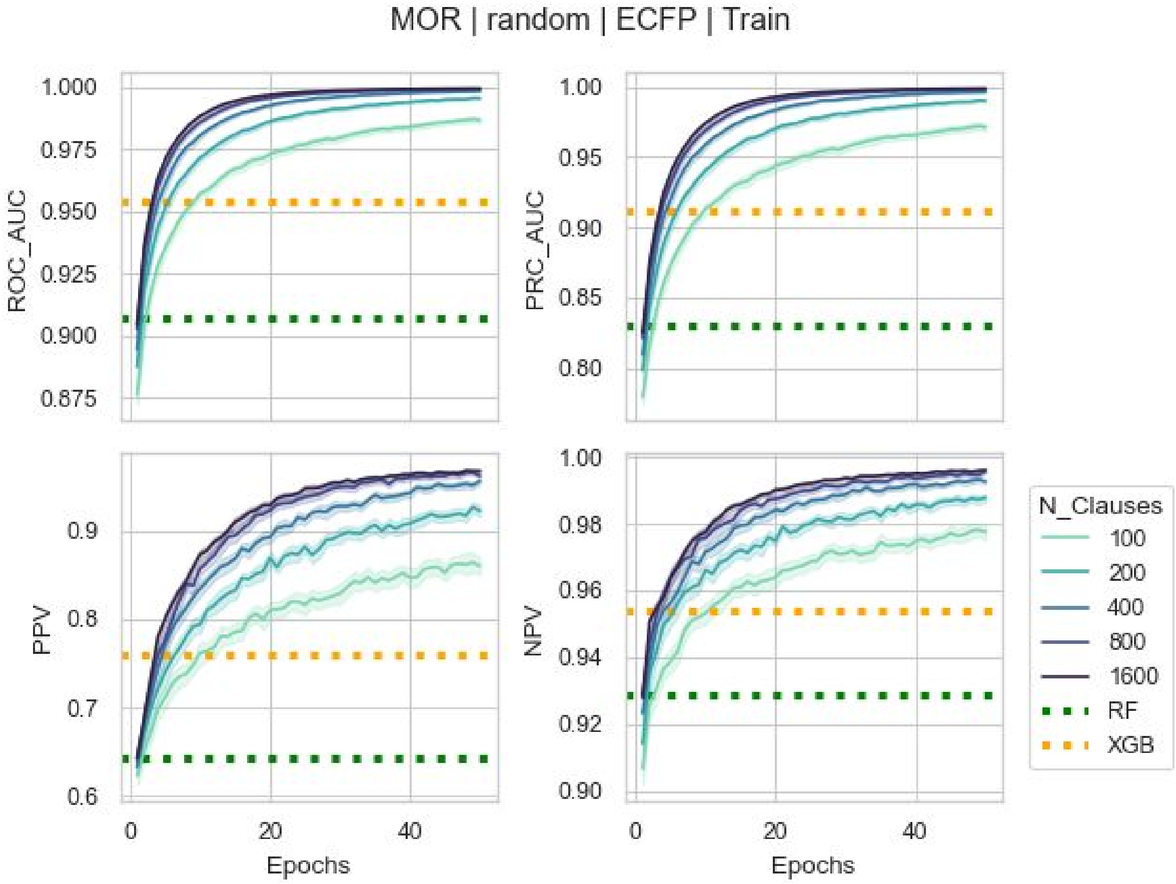

Figure S2: TM metric scores over 50 epochs on MOR training sets for random split-group and ECFP descriptors. Annotated by dotted lines are the mean training set scores of RF and XGBoost.

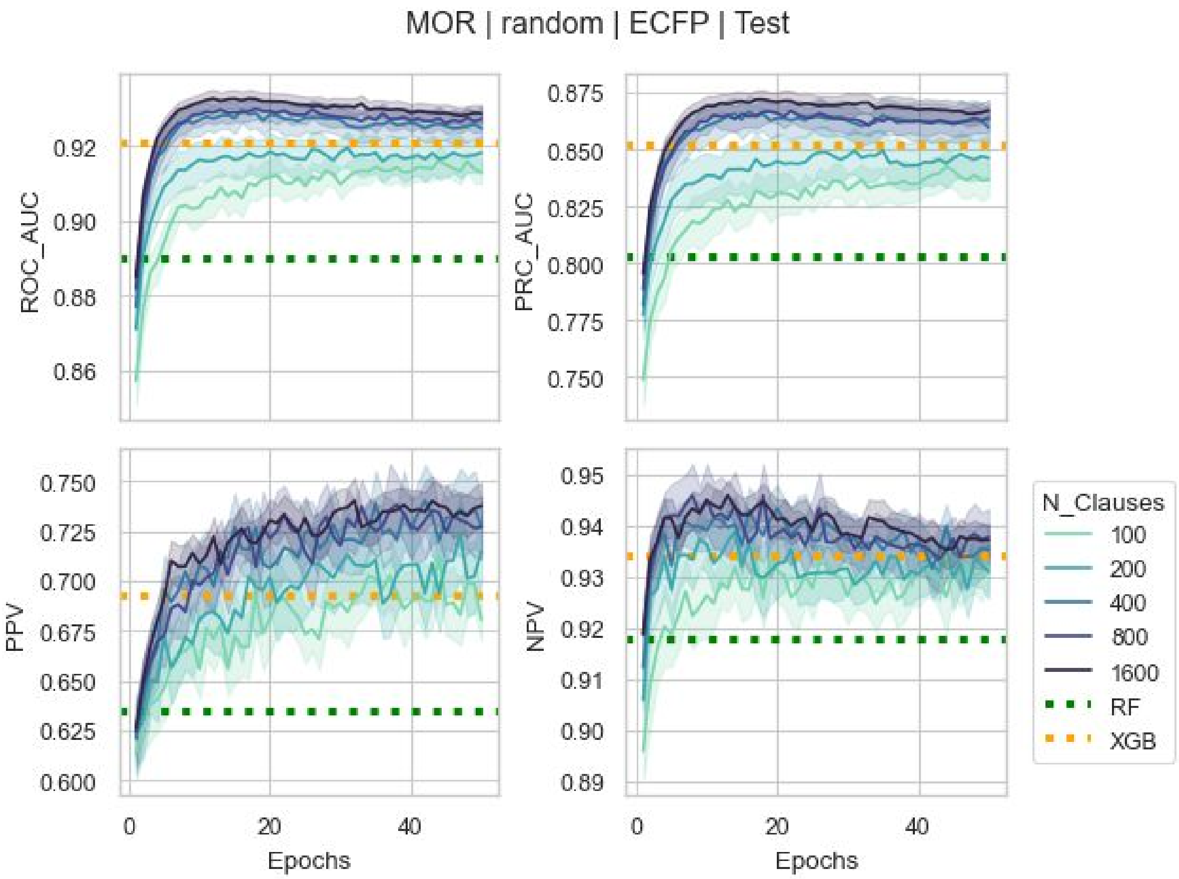

Figure S3: TM metric scores over 50 epochs on MOR test sets for random split-group and ECFP descriptors. Annotated by dotted lines are the mean test set scores of RF and XGBoost.

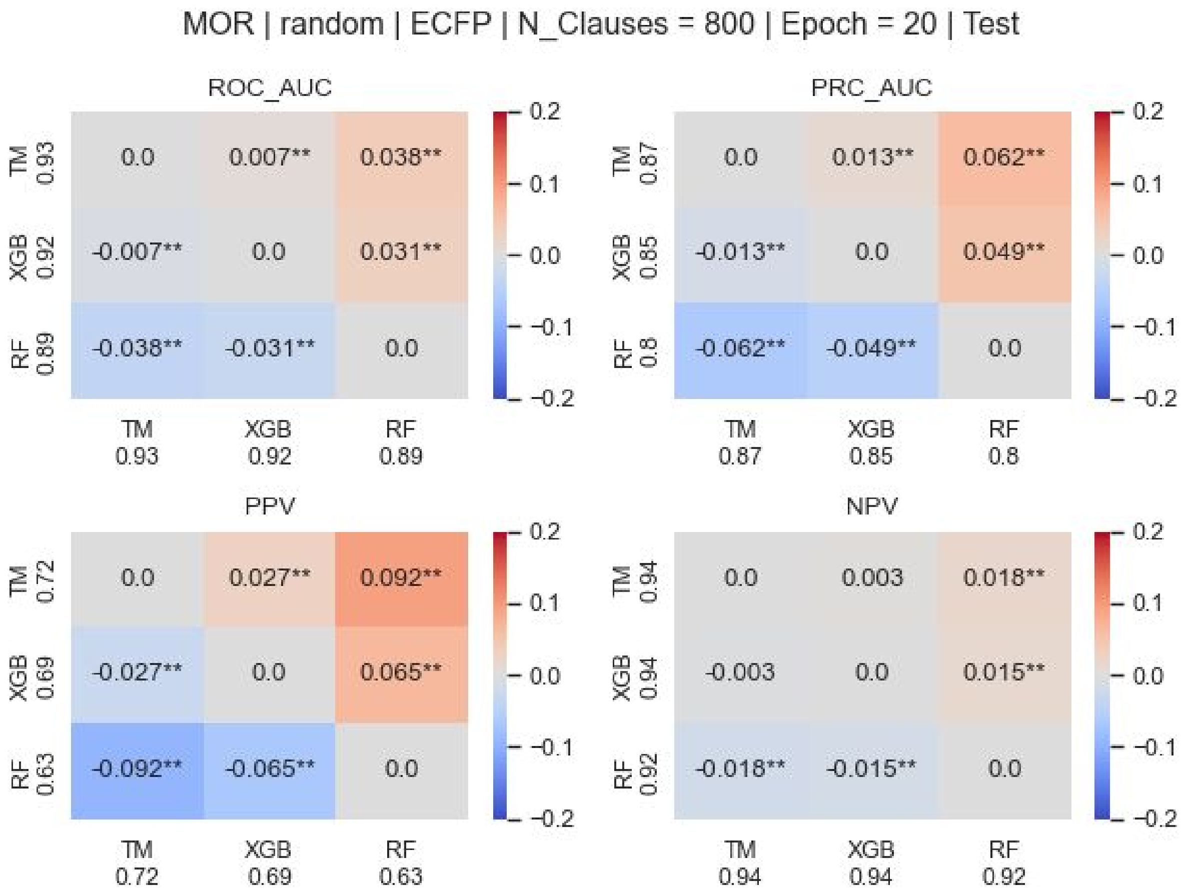

Figure S4: Cohen's D difference of means, pair-wise comparison of models for the MOR dataset with random group-split, ECFP descriptors and TM models of 800 clauses at 20 epochs. Complete with annotated statistical tests via Tukey's HSD where the number of asterix represents a different statistical significance level.

Hyper-parameter Search

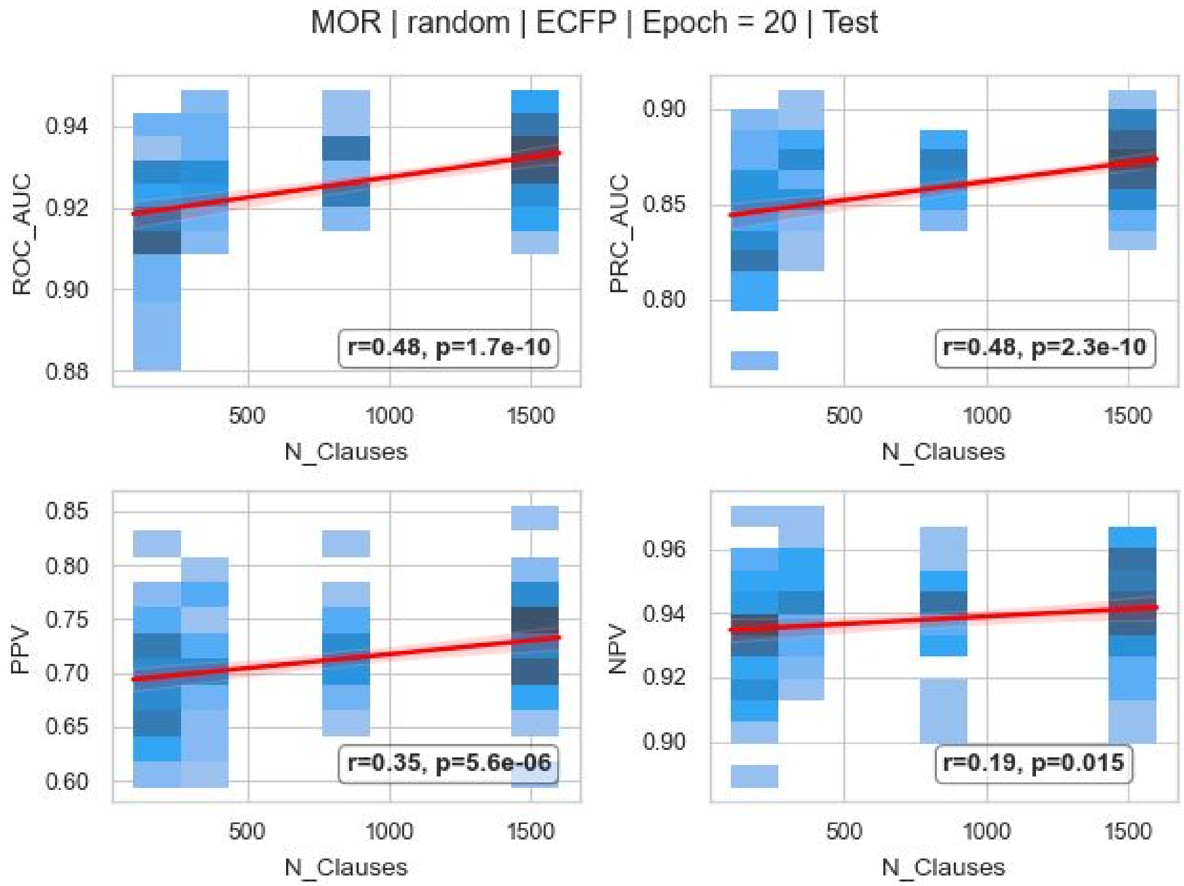

Figure S5: Number of clauses and metric-score histograms of MOR test sets with fitted line for random split-group and ECFP descriptors. Pearson's R and p-value are annotated for said line.

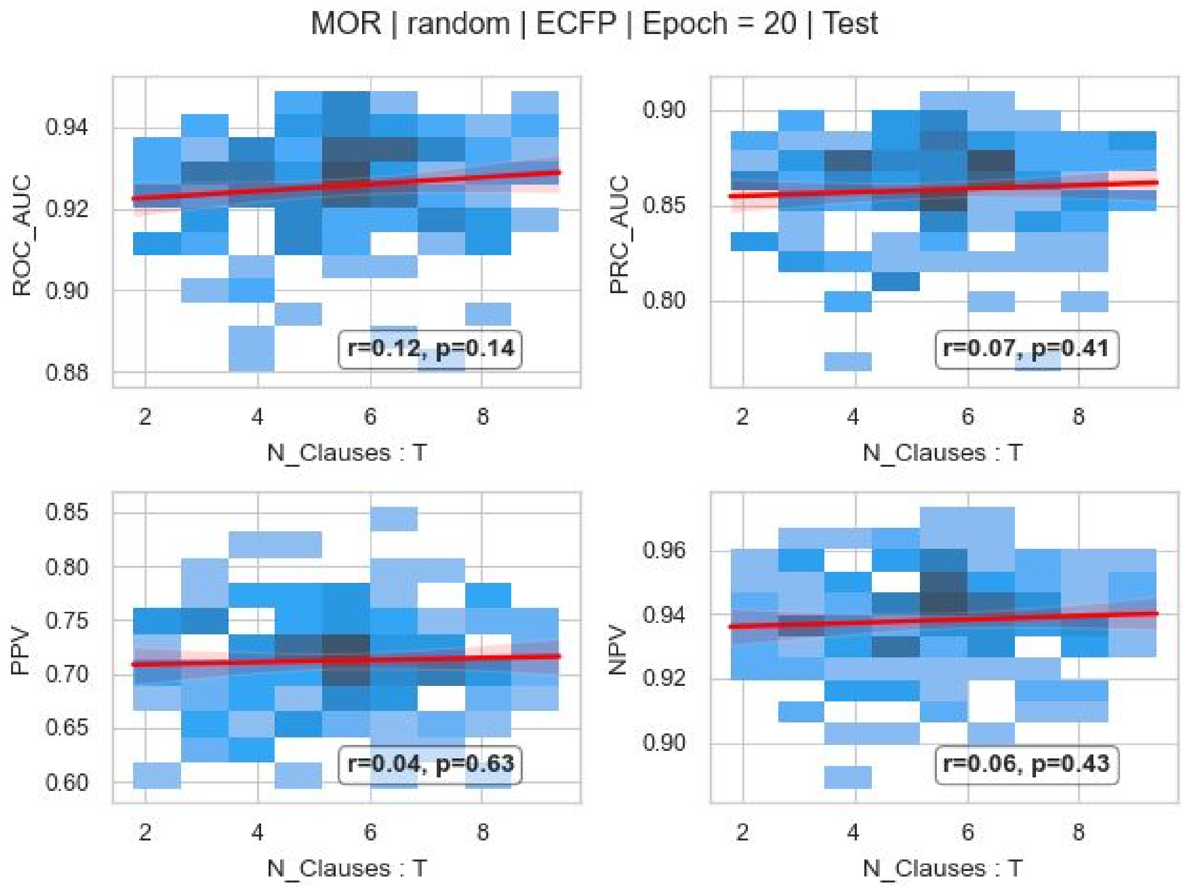

Figure S6: *N_Clauses : T* ratio and test-set metric scores histogram for MOR dataset with fitted line for random split-group and ECFP descriptors. Pearson's R and associated p-value are annotated for said line.

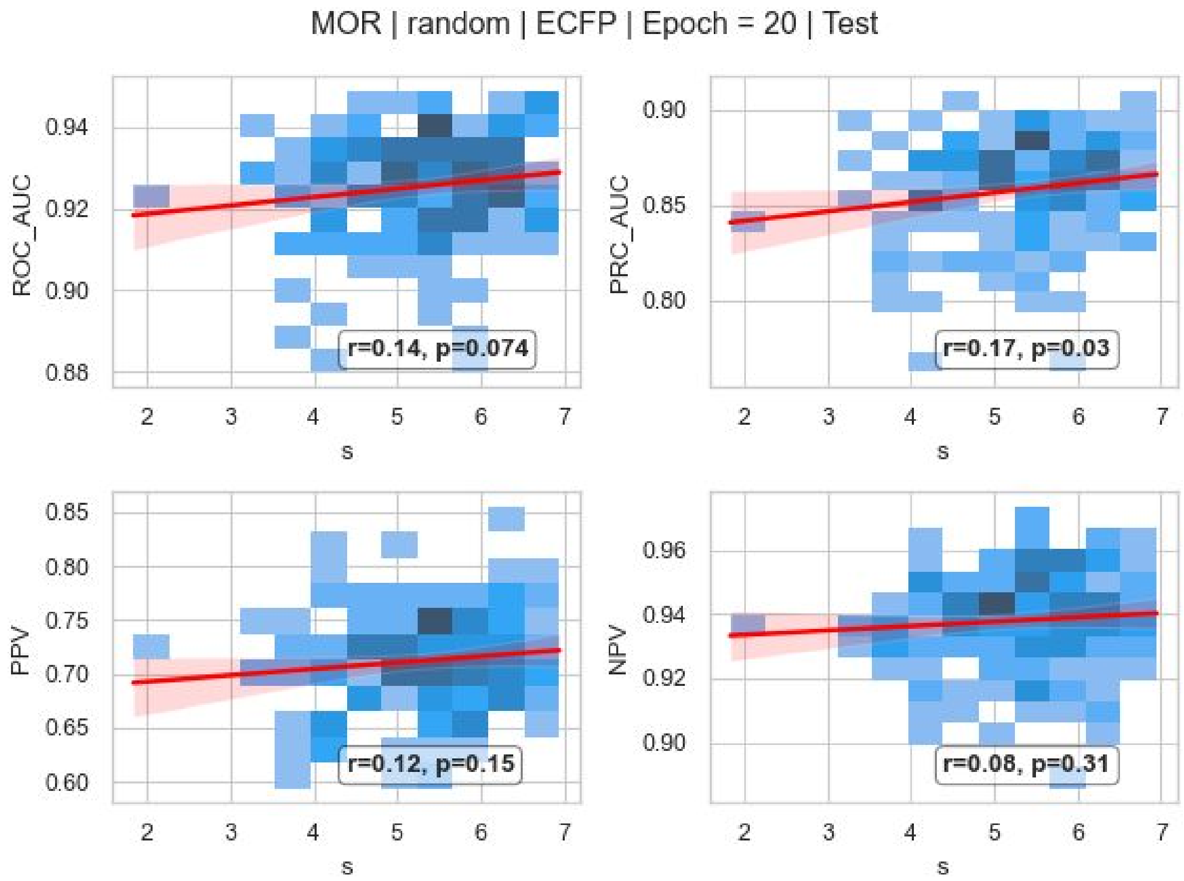

Figure S7: Hyper-parameter *s* and test-set metric scores histogram for MOR dataset with fitted line for random split-group, ECFP descriptors and TM-models of 800 clauses at 20 epochs. Pearson's R and p-value are annotated for said line.

scaffold split-group | ECFP Descriptors

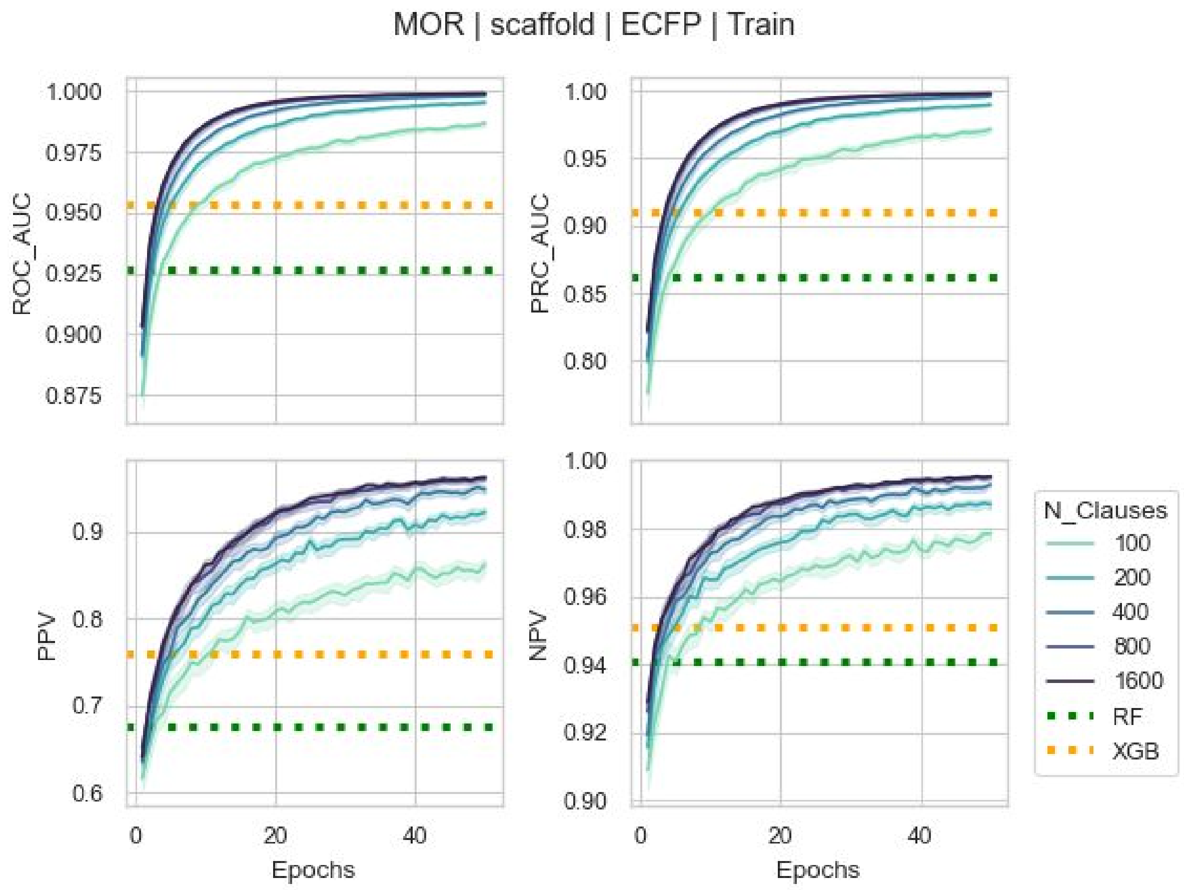

Figure S8: TM metric scores over 50 epochs on MOR training sets for scaffold split-group and ECFP descriptors. Annotated by dotted lines are the mean training set scores of RF and XGBoost.

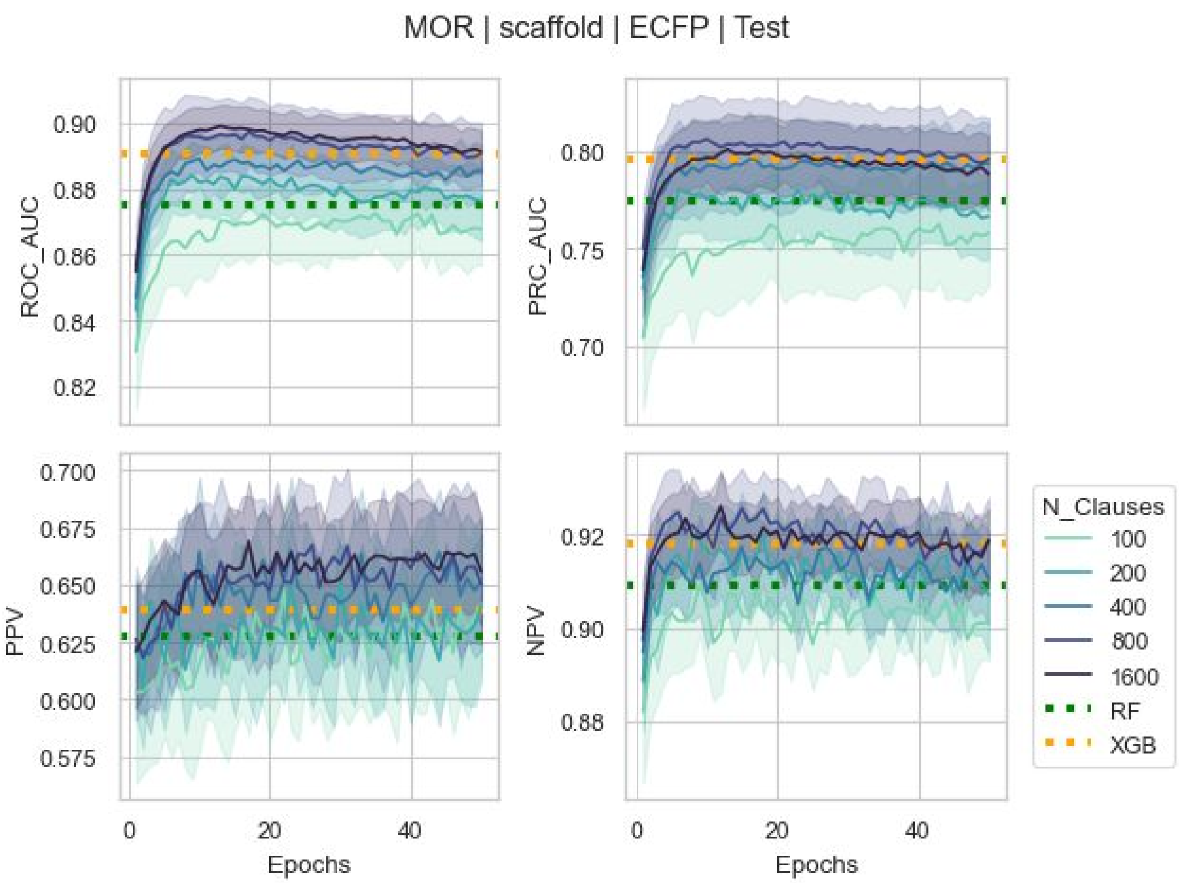

Figure S9: TM metric scores over 50 epochs on MOR test sets for scaffold split-group and ECFP descriptors. Annotated by dotted lines are the mean training set scores of RF and XGBoost.

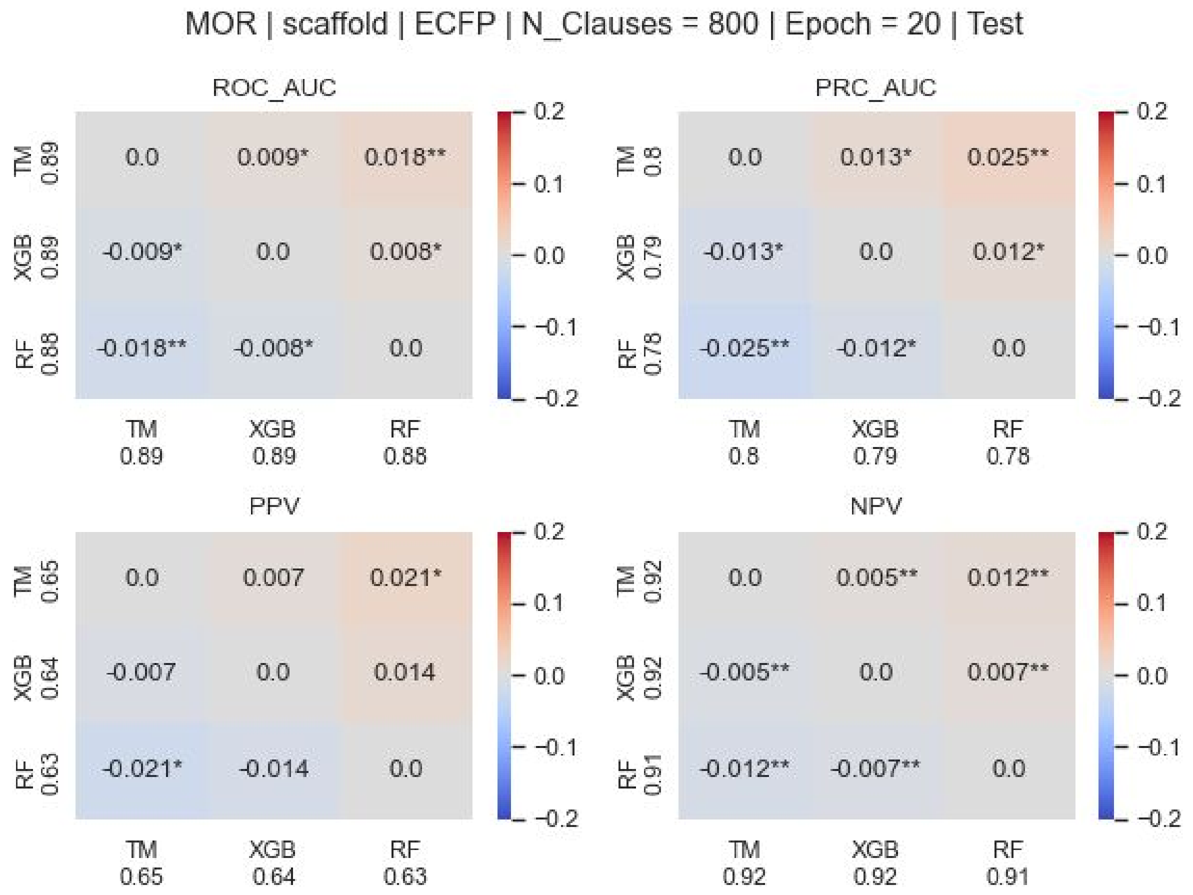

Figure S10: Cohen's D difference of means, pair-wise comparison of models for the MOR dataset with scaffold group-split, ECFP descriptors and TM models of 800 clauses at 20 epochs. Complete with annotated statistical tests via Tukey's HSD where the number of asterix represents a different statistical significance level.

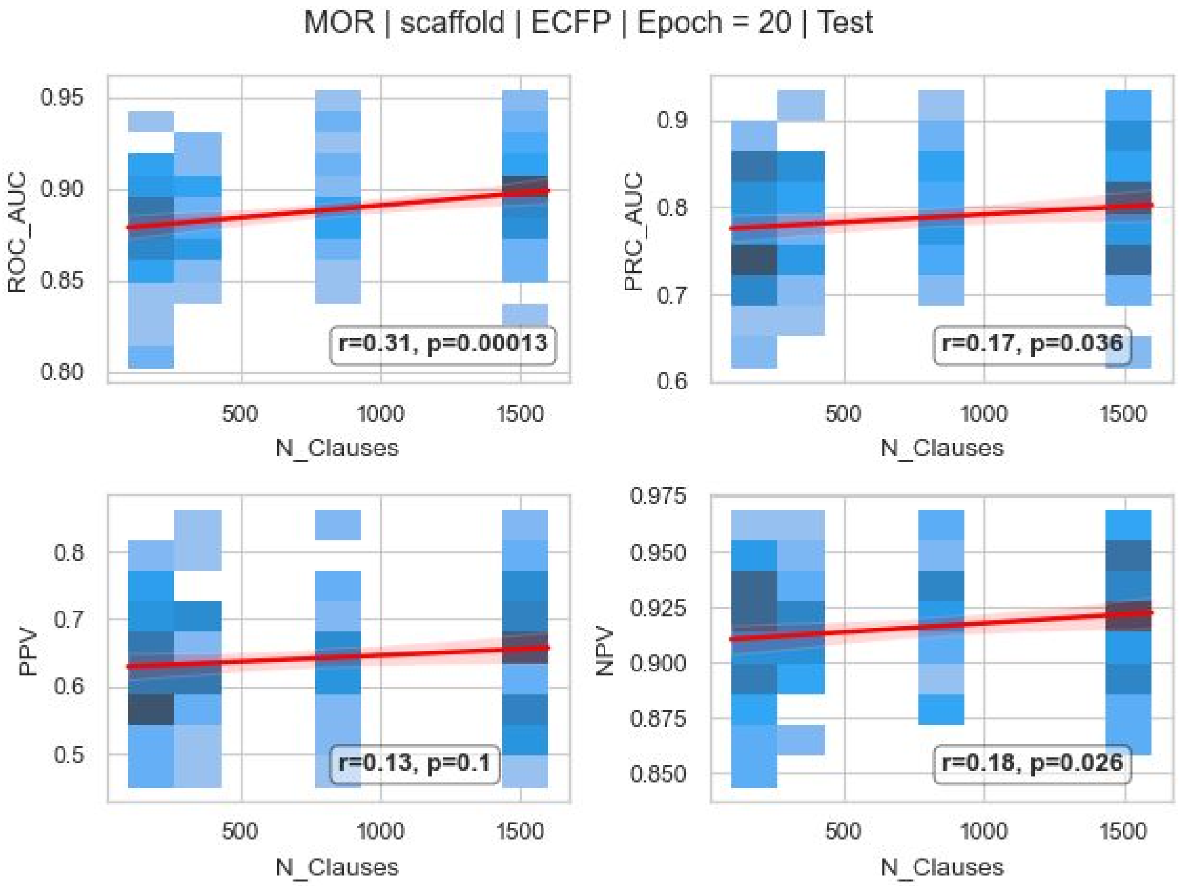

Figure S11: Number of clauses and metric-score histograms of MOR test sets with fitted line for scaffold split-group, ECFP descriptors and TM-models of 800 clauses at 20 epochs. Pearson's R and p-value are annotated for said line.

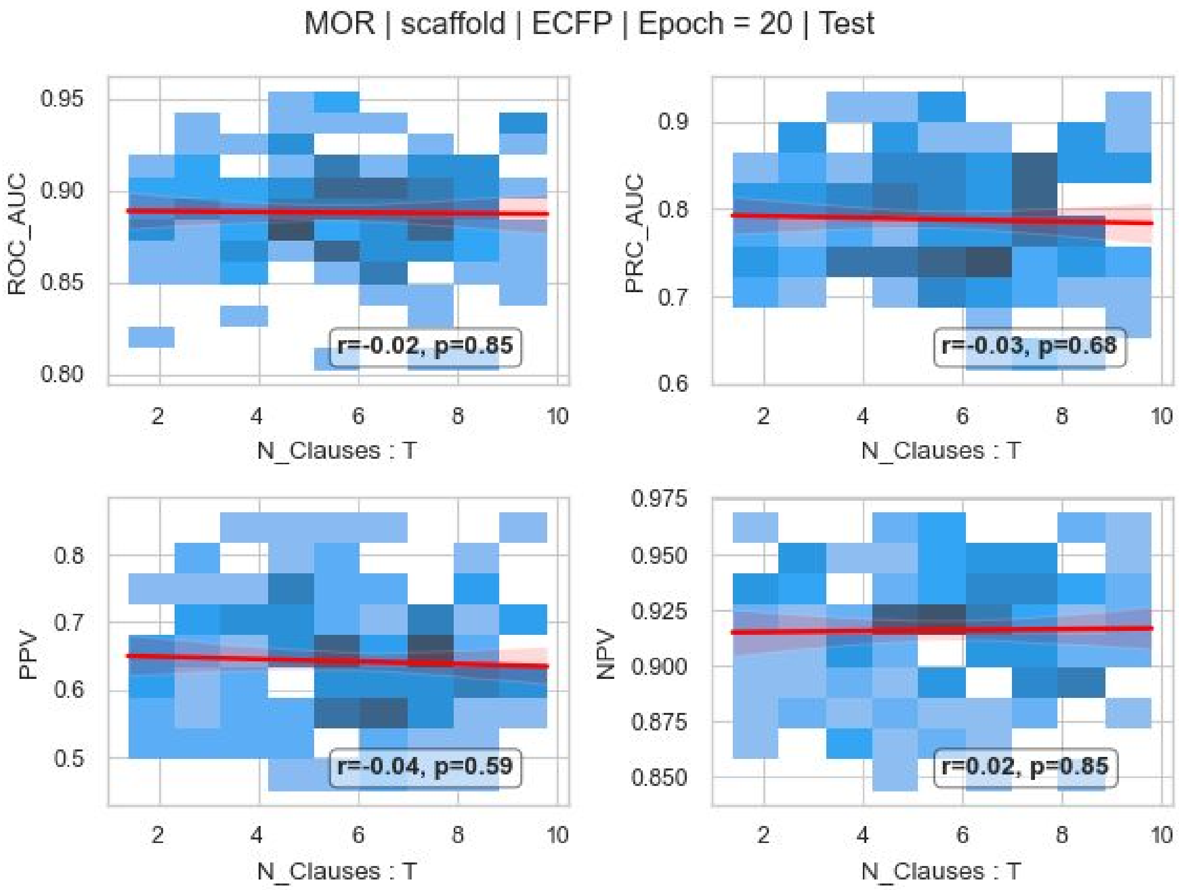

Figure S12: *N_Clauses : T* ratio and test-set metric scores histogram of MOR test sets with fitted line for scaffold split-group, ECFP descriptors and TM-models of 800 clauses at 20 epochs. Pearson's R and p-value are annotated for said line.

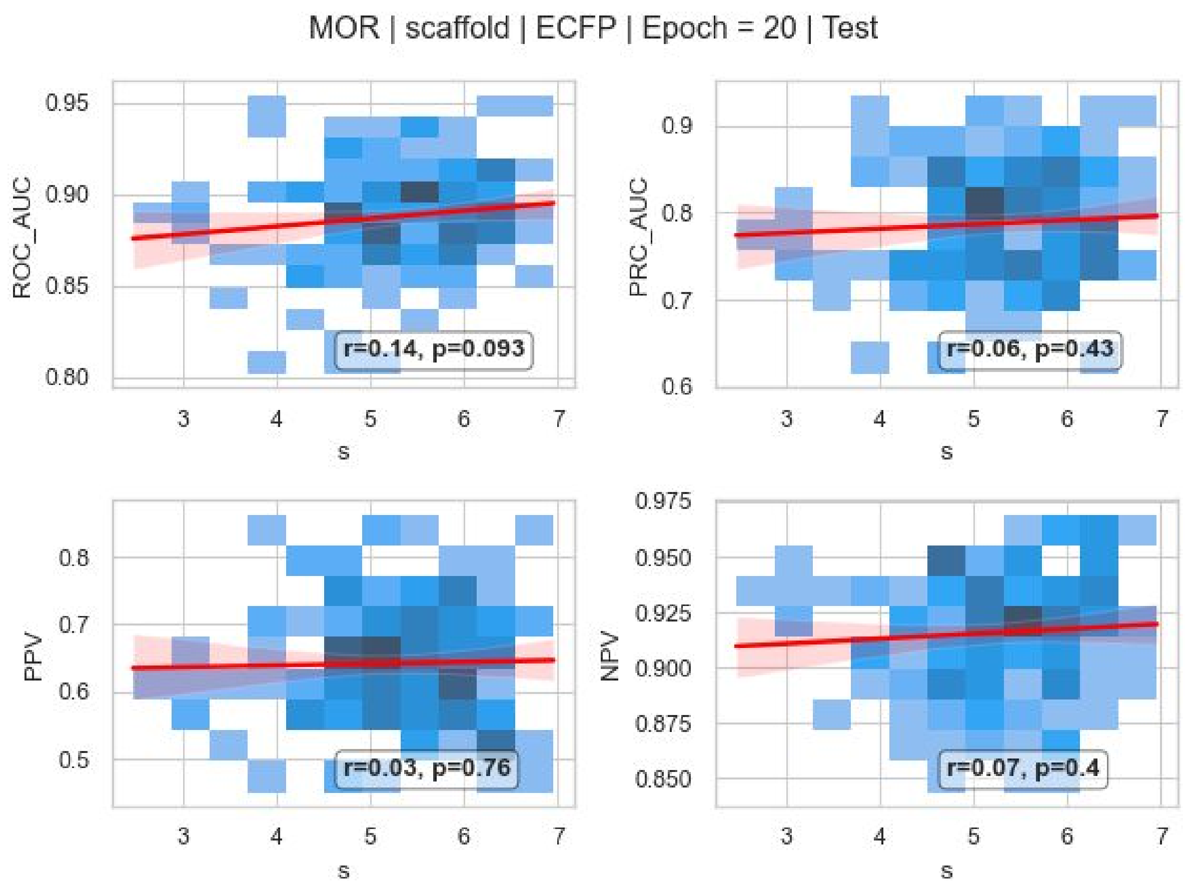

Figure S13: Hyper-parameter *s* and test-set metric scores histogram of MOR test sets with fitted line for scaffold split-group, ECFP descriptors and TM-models of 800 clauses at 20 epochs. Pearson's R and p-value are annotated for said line.

random split-group | RDKit2D Descriptors

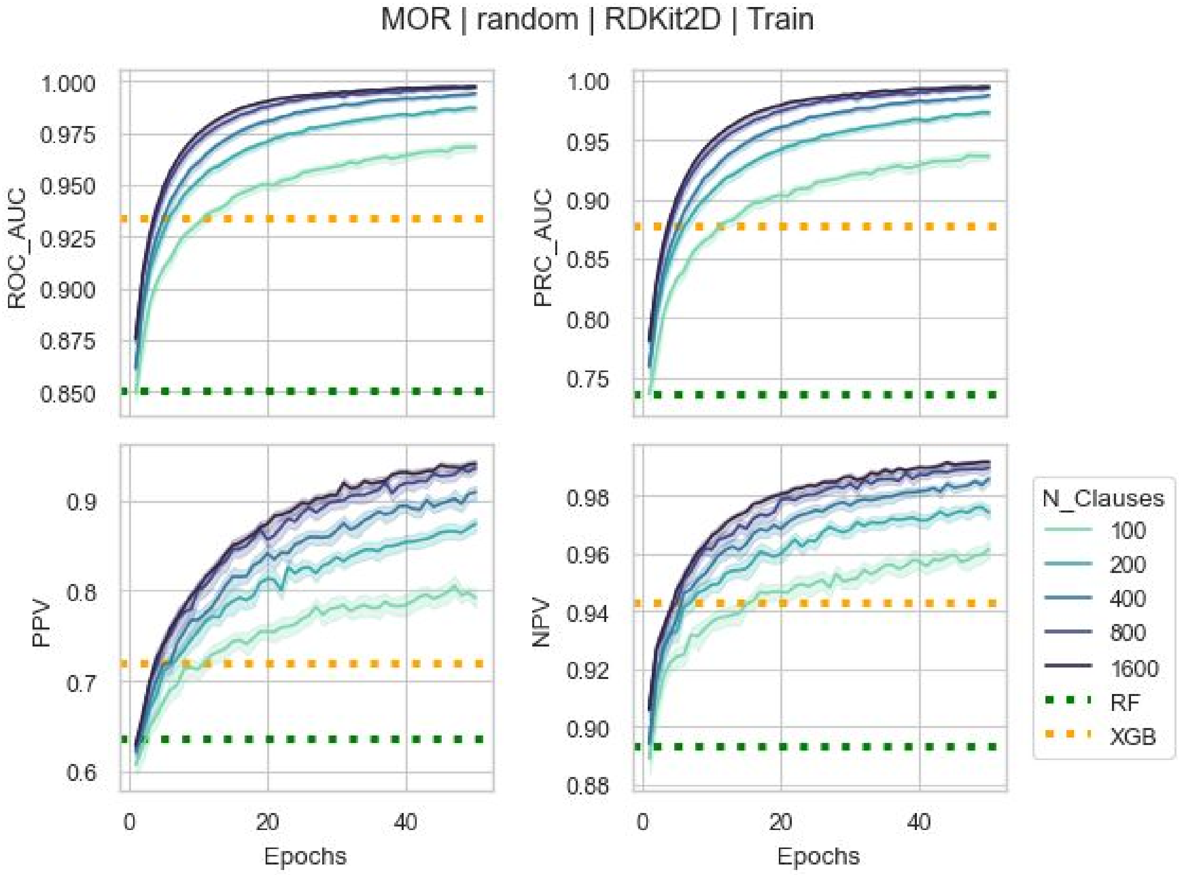

Figure S14: TM metric scores over 50 epochs on MOR training sets for random split-group and RDKit2D descriptors. Annotated by dotted lines are the mean training set scores of RF and XGBoost.

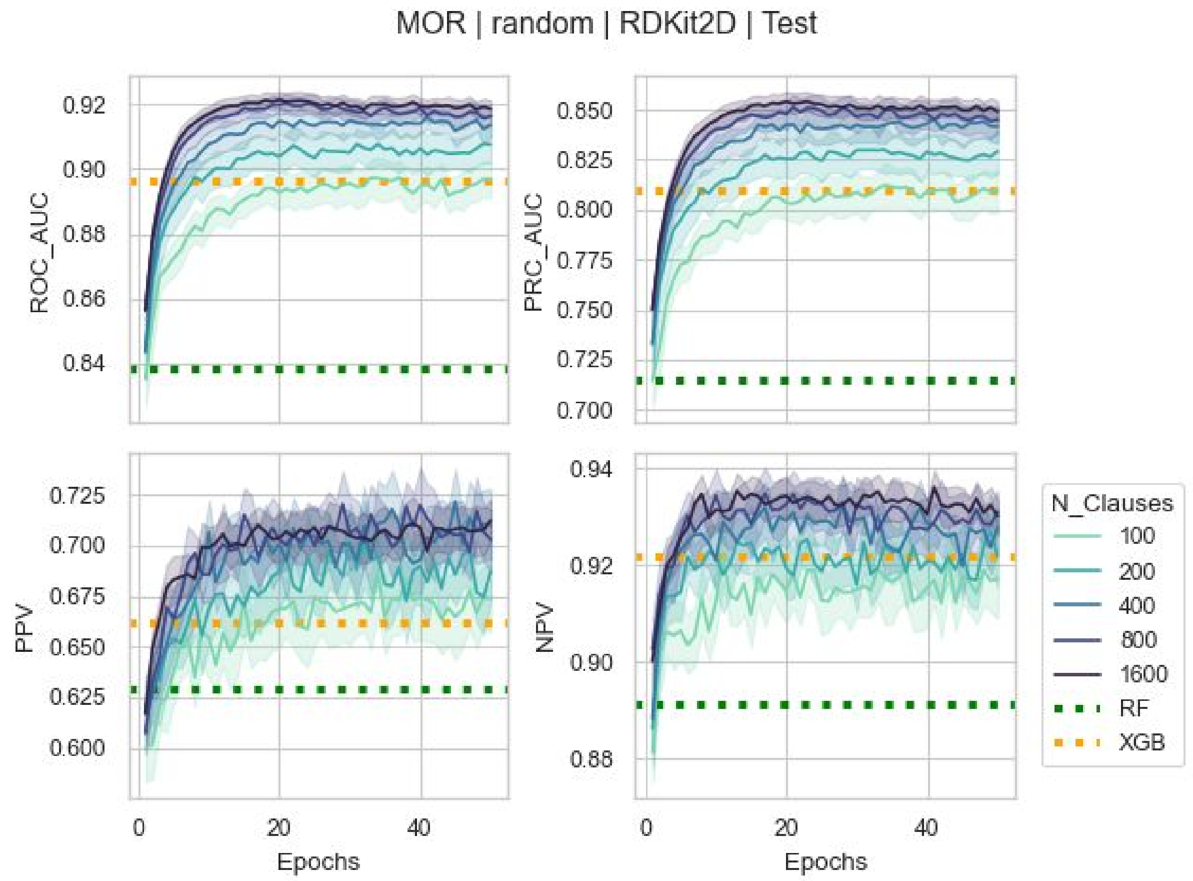

Figure S15: TM metric scores over 50 epochs on MOR test sets for random split-group and RDKit2D descriptors. Annotated by dotted lines are the mean training set scores of RF and XGBoost.

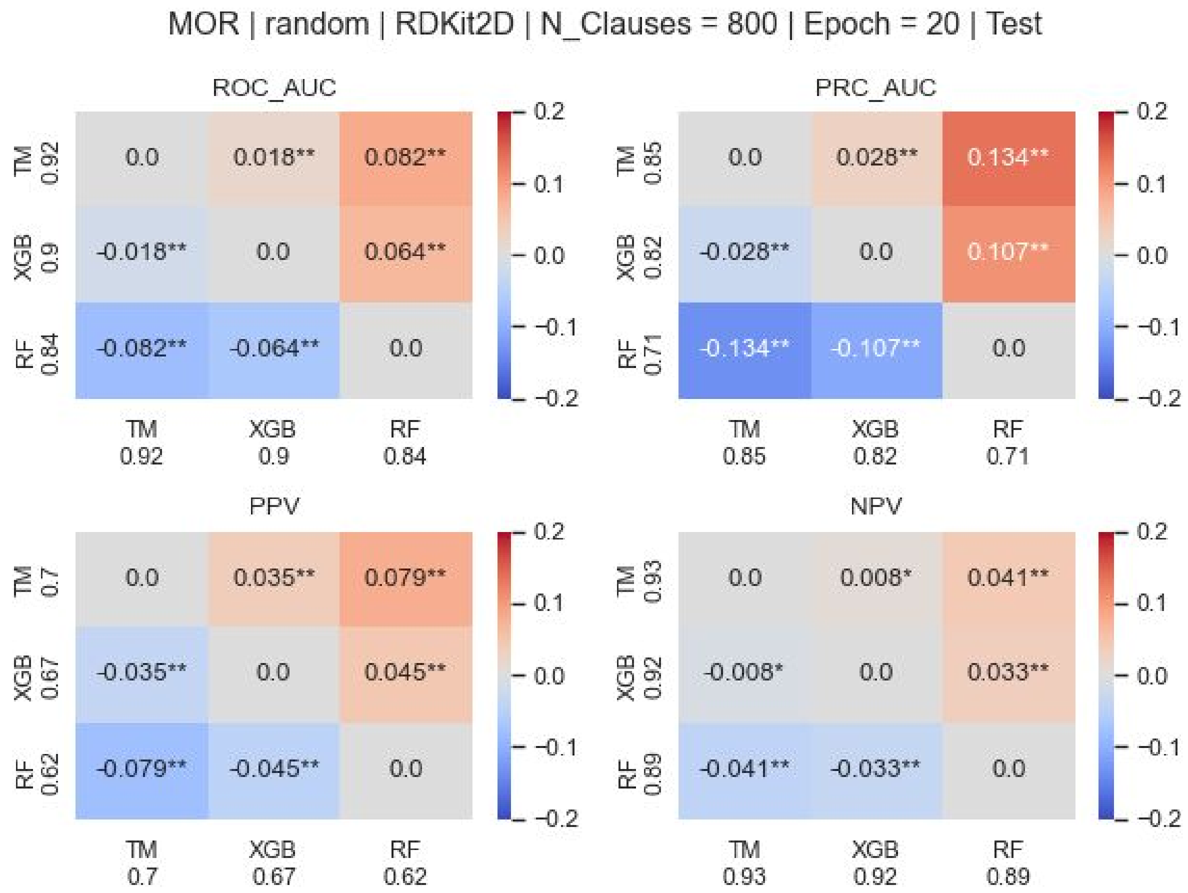

Figure S16: Cohen's D difference of means, pair-wise comparison of models for the MOR dataset with random group-split, RDKit2D descriptors and TM models of 800 clauses at 20 epochs. Complete with annotated statistical tests via Tukey's HSD where the number of asterix represents a different statistical significance level.

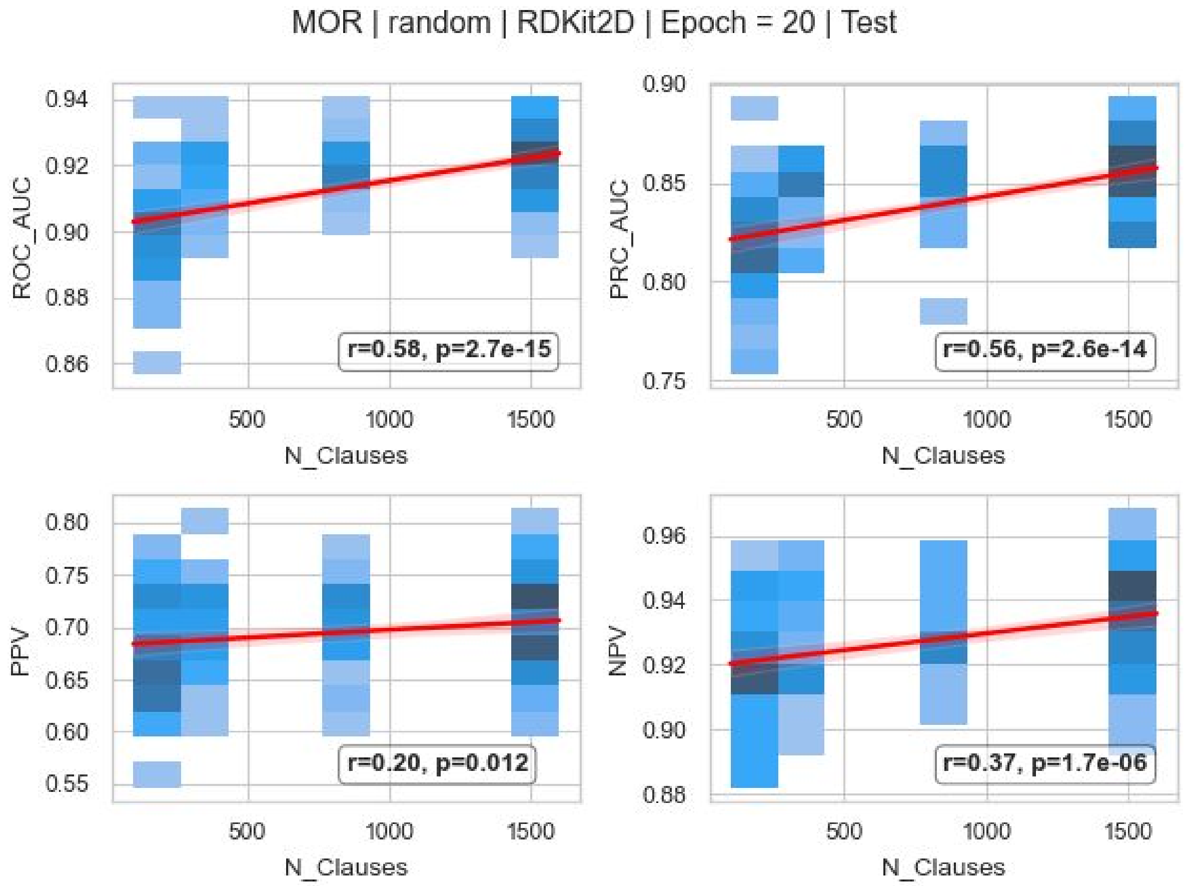

Figure S17: Number of clauses and metric-score histograms of MOR test sets with fitted line for random split-group, RDKit2D descriptors and TM-models of 800 clauses at 20 epochs. Pearson's R and p-value are annotated for said line.

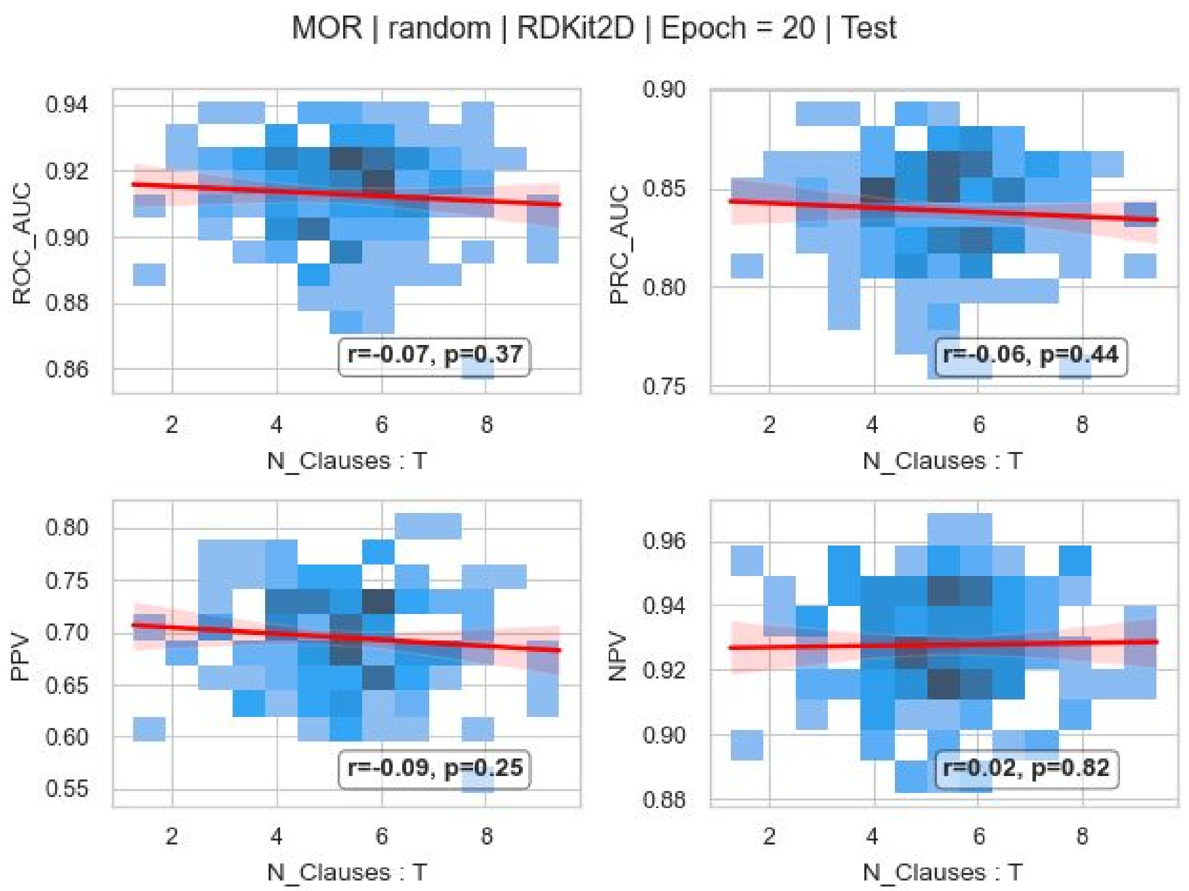

Figure S18: *N_Clauses : T* ratio and test-set metric scores histogram of MOR test sets with fitted line for random split-group, RDKit2D descriptors and TM-models of 800 clauses at 20 epochs. Pearson's R and p-value are annotated for said line.

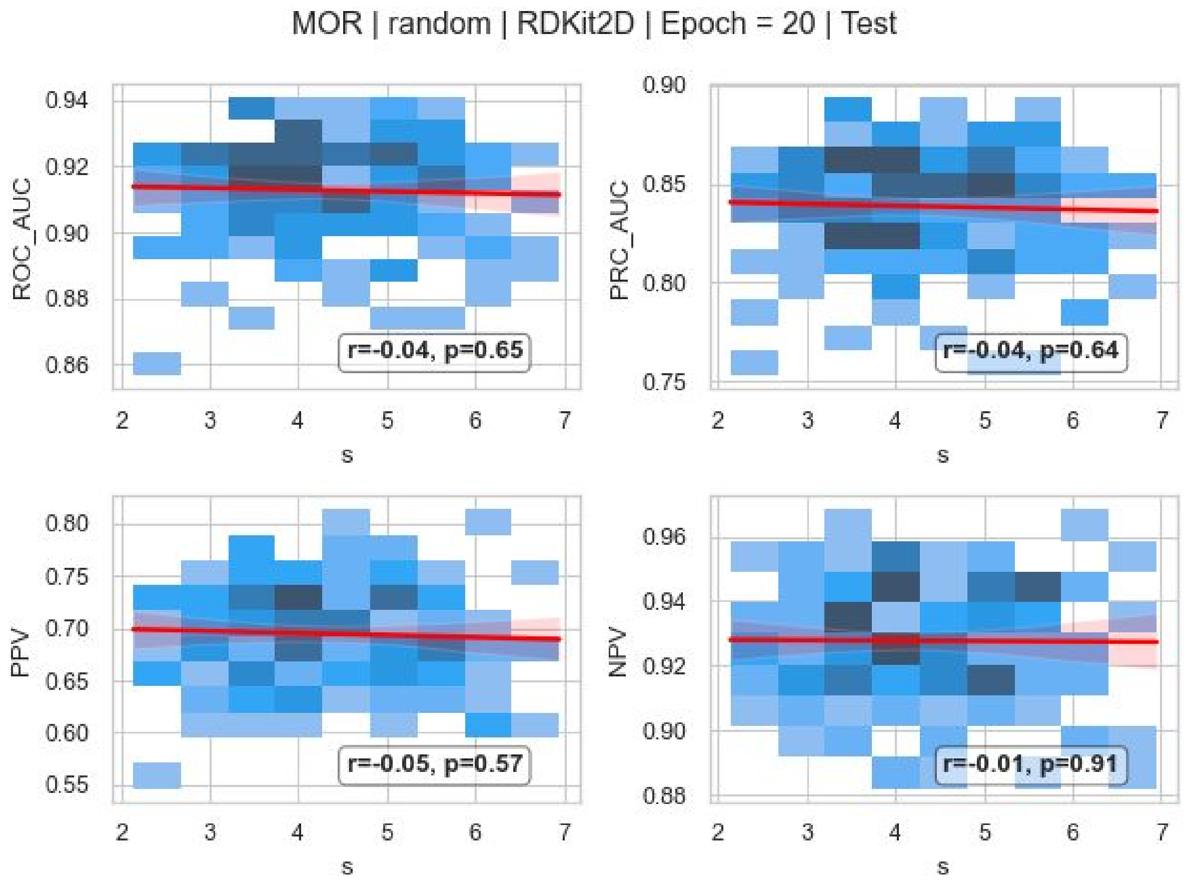

Figure S19: Hyper-parameter *s* and test-set metric scores histogram of MOR test sets with fitted line for random split-group, RDKit2D descriptors and TM-models of 800 clauses at 20 epochs. Pearson's R and p-value are annotated for said line.

scaffold group-split | RDKit2D Descriptors

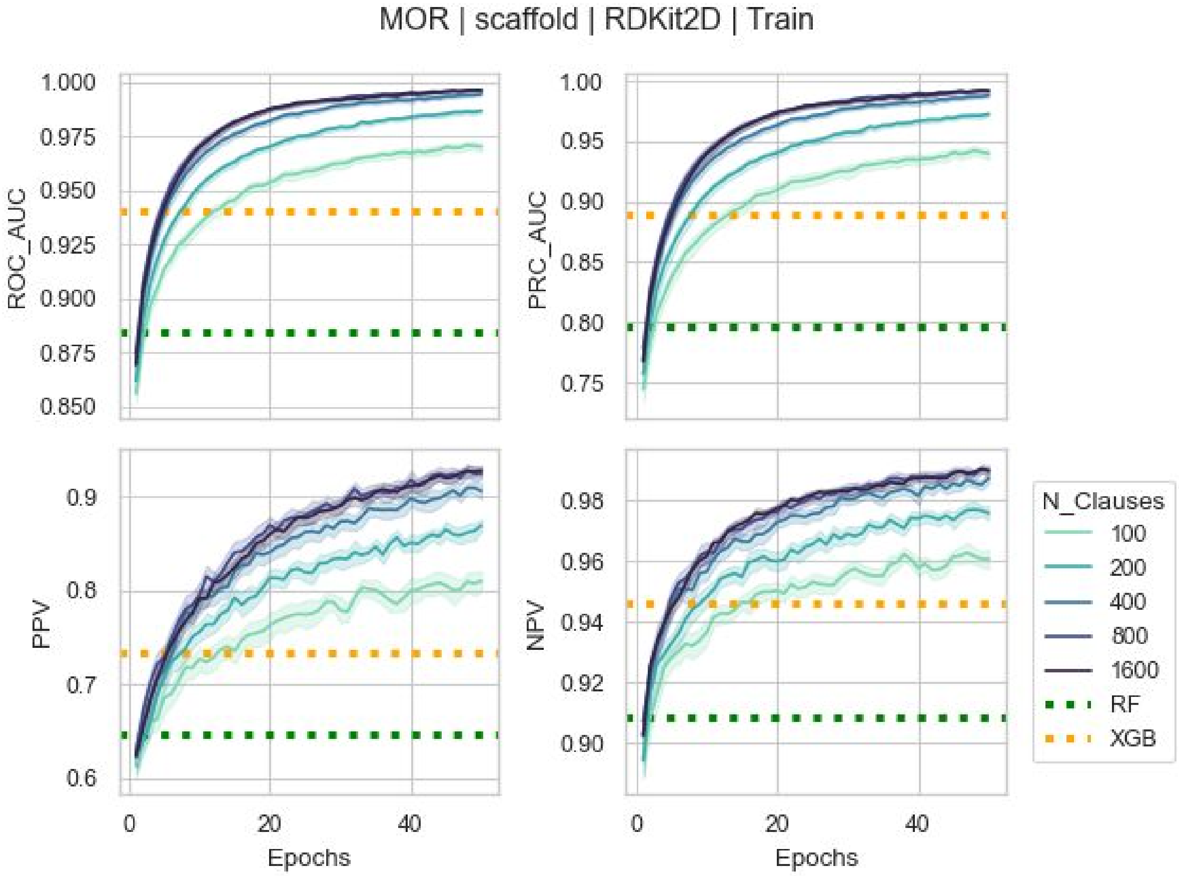

Figure S20: TM metric scores over 50 epochs on MOR training sets for scaffold split-group and RDKit2D descriptors. Annotated by dotted lines are the mean training set scores of RF and XGBoost.

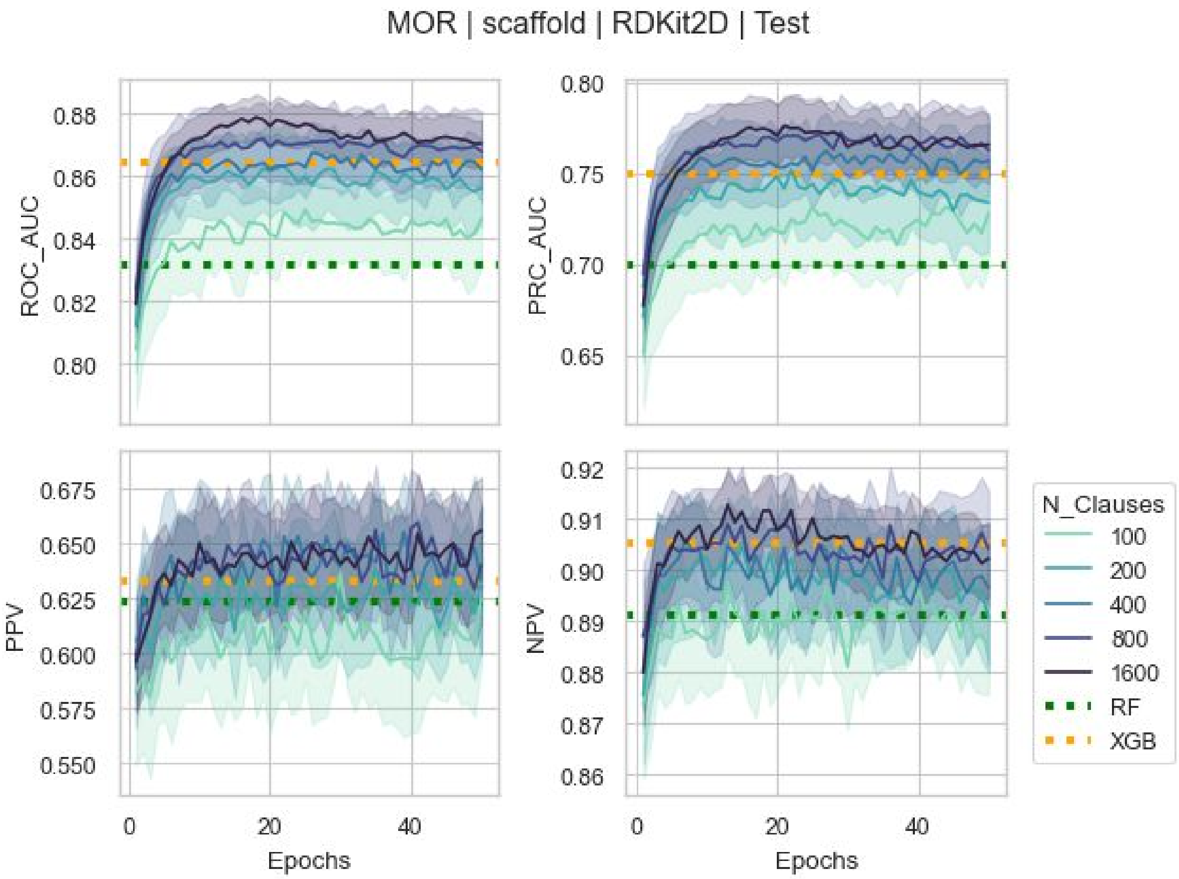

Figure S21: TM metric scores over 50 epochs on MOR test sets for scaffold split-group and RDKit2D descriptors. Annotated by dotted lines are the mean training set scores of RF and XGBoost.

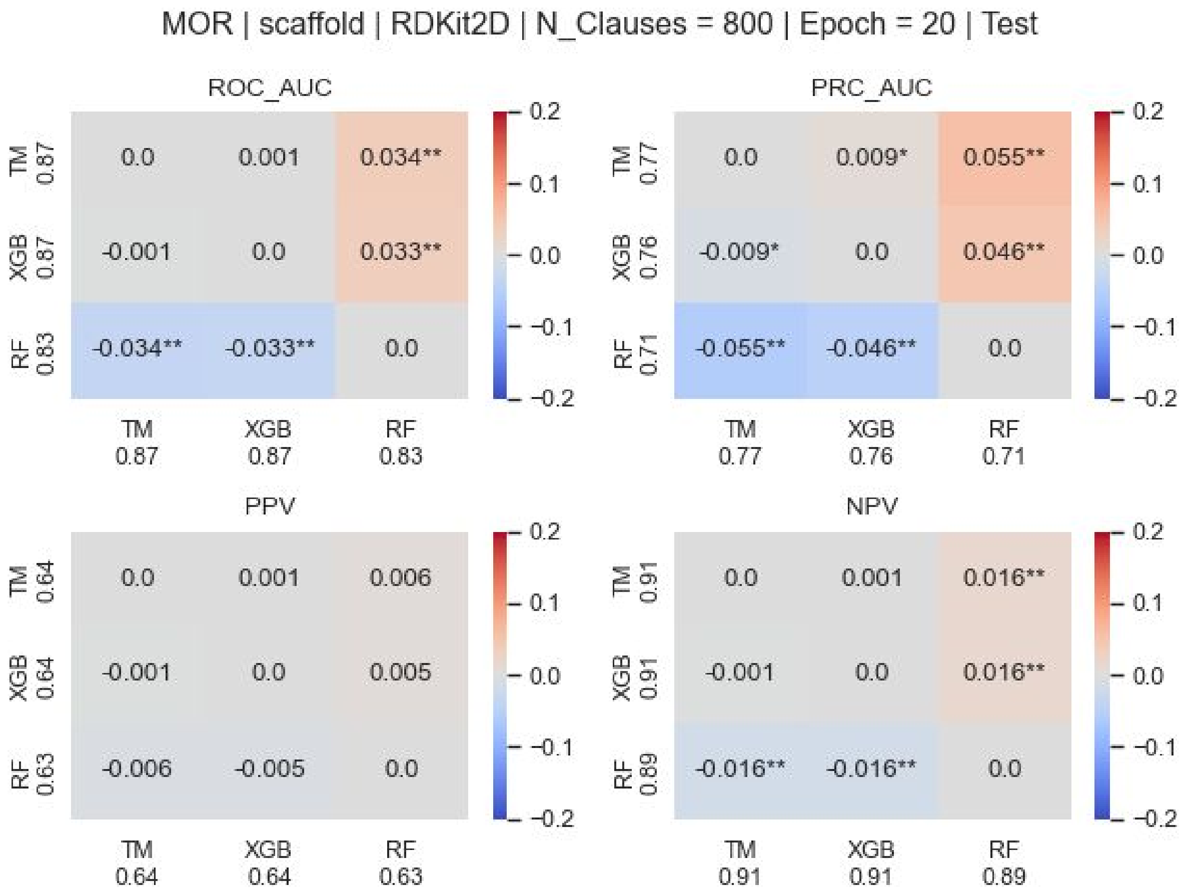

Figure S22: Cohen's D difference of means, pair-wise comparison of models for the MOR dataset with scaffold group-split, RDKit2D descriptors and TM models of 800 clauses at 20 epochs. Complete with annotated statistical tests via Tukey's HSD where the number of asterix represents a different statistical significance level.

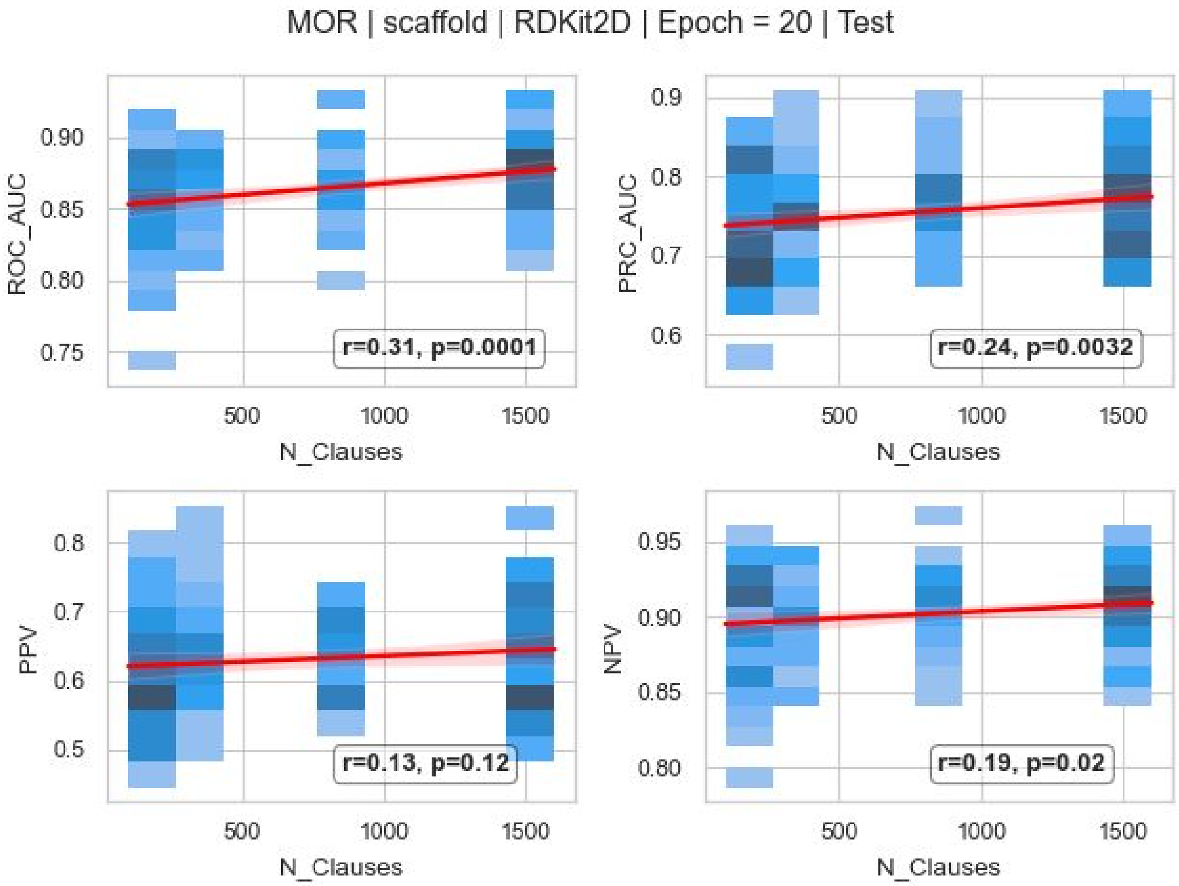

Figure S23: Number of clauses and metric-score histograms of MOR test sets with fitted line for scaffold split-group, RDKit2D descriptors and TM-models of 800 clauses at 20 epochs. Pearson's R and p-value are annotated for said line.

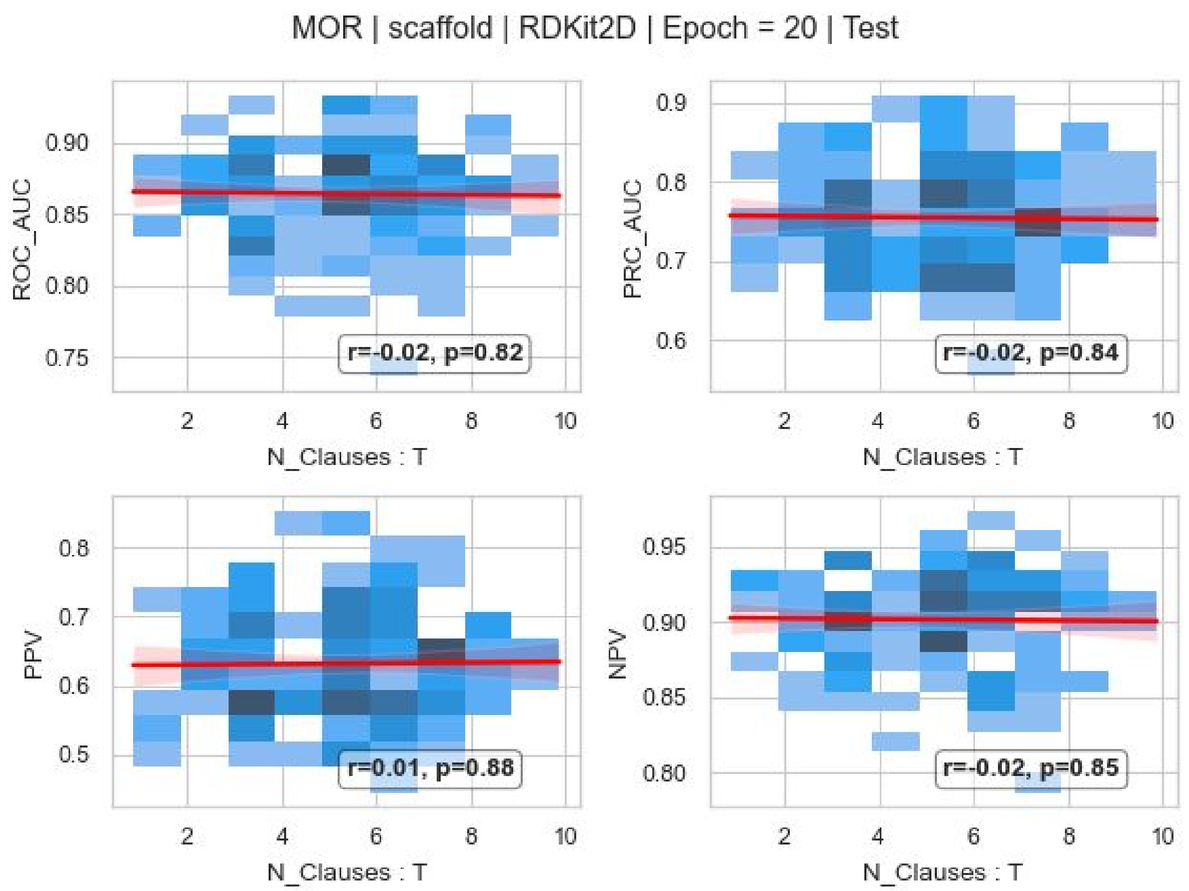

Figure S24: *N_Clauses : T* ratio and test-set metric scores histogram of MOR test sets with fitted line for scaffold split-group, RDKit2D descriptors and TM-models of 800 clauses at 20 epochs. Pearson's R and p-value are annotated for said line.

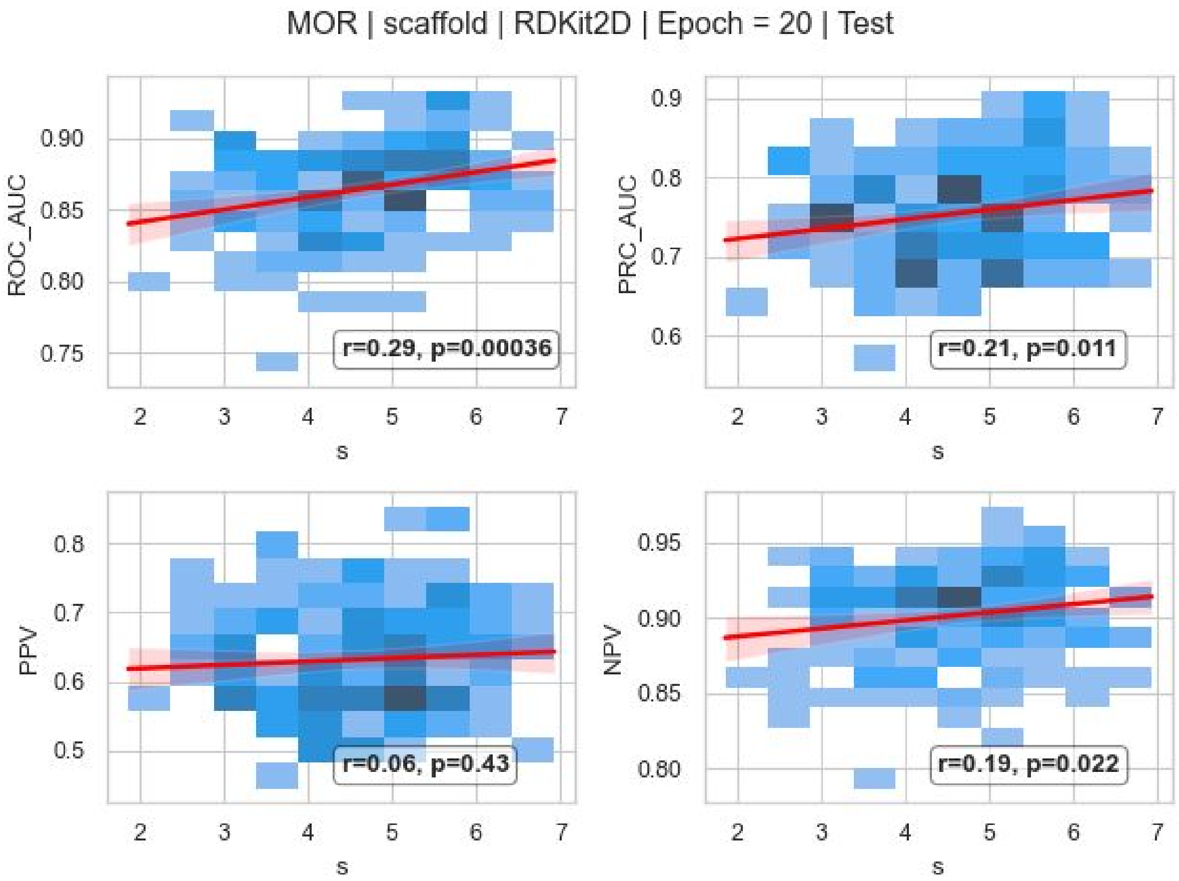

Figure S25: Hyper-parameter *s* and test-set metric scores histogram of MOR test sets with fitted line for random split-group, RDKit2D descriptors and TM-models of 800 clauses at 20 epochs. Pearson's R and p-value are annotated for said line.

δ - *Opioid Receptor*

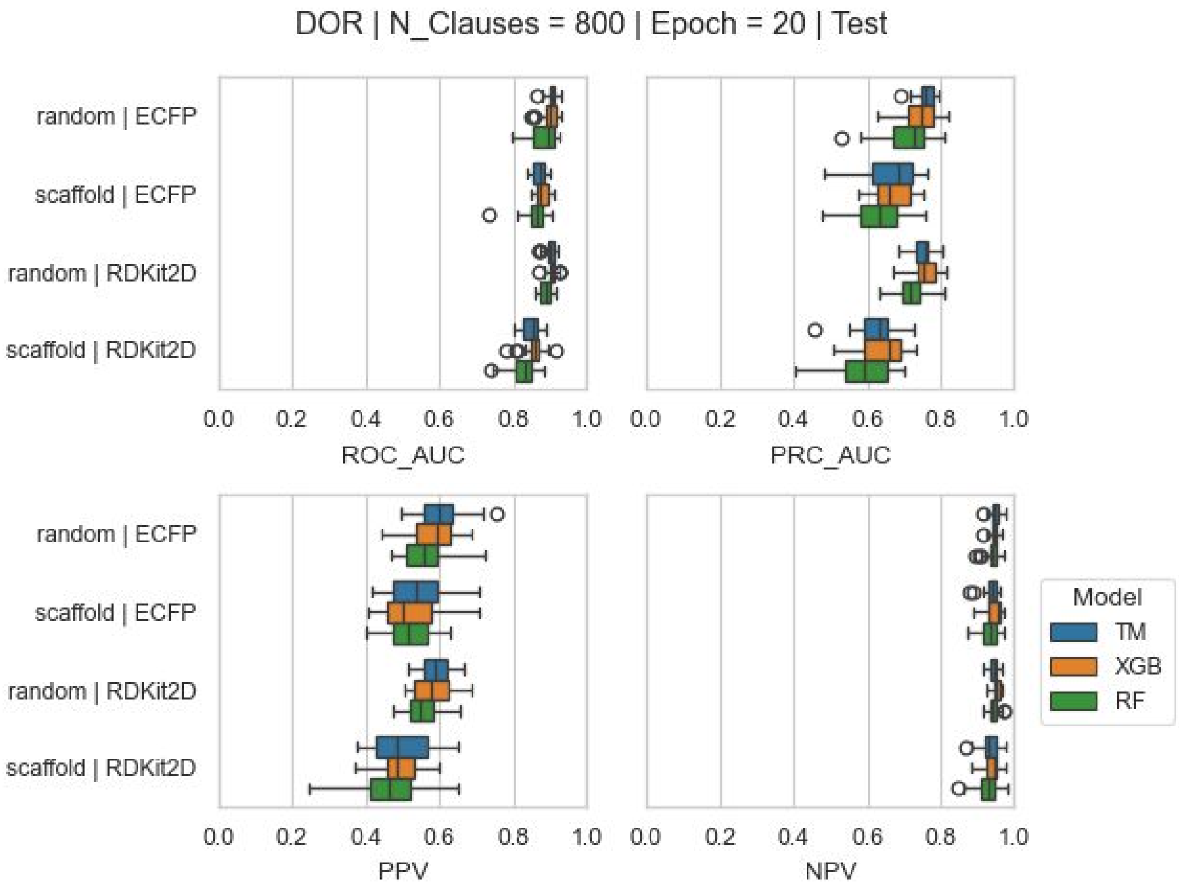

Figure S26: Box plot of model metric scores on a test set across split-group | descriptor pairs for DOR dataset. The TM uses 800 clauses and learning is stopped after 20 epochs.

random split-group I ECFP Descriptors

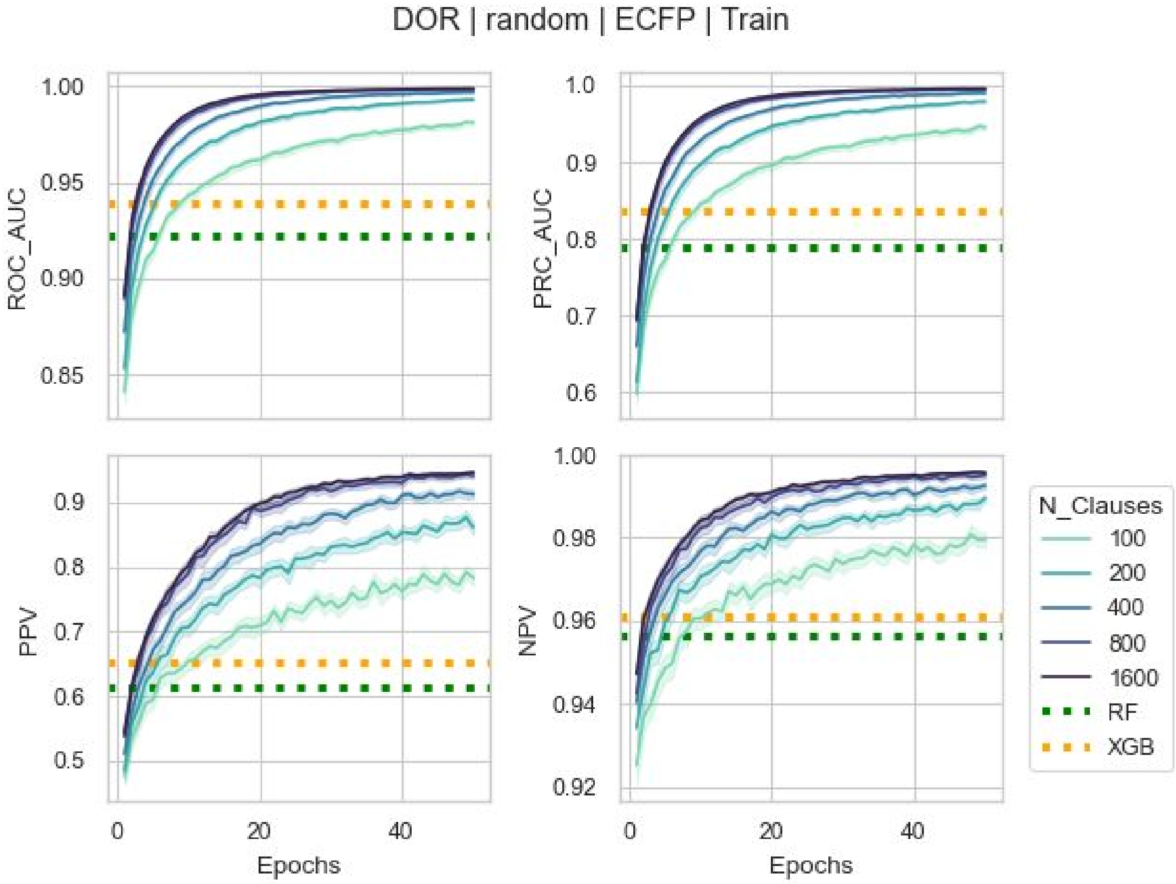

Figure S27: TM metric scores over 50 epochs on DOR training sets for random split-group and ECFP descriptors. Annotated by dotted lines are the mean training set scores of RF and XGBoost.

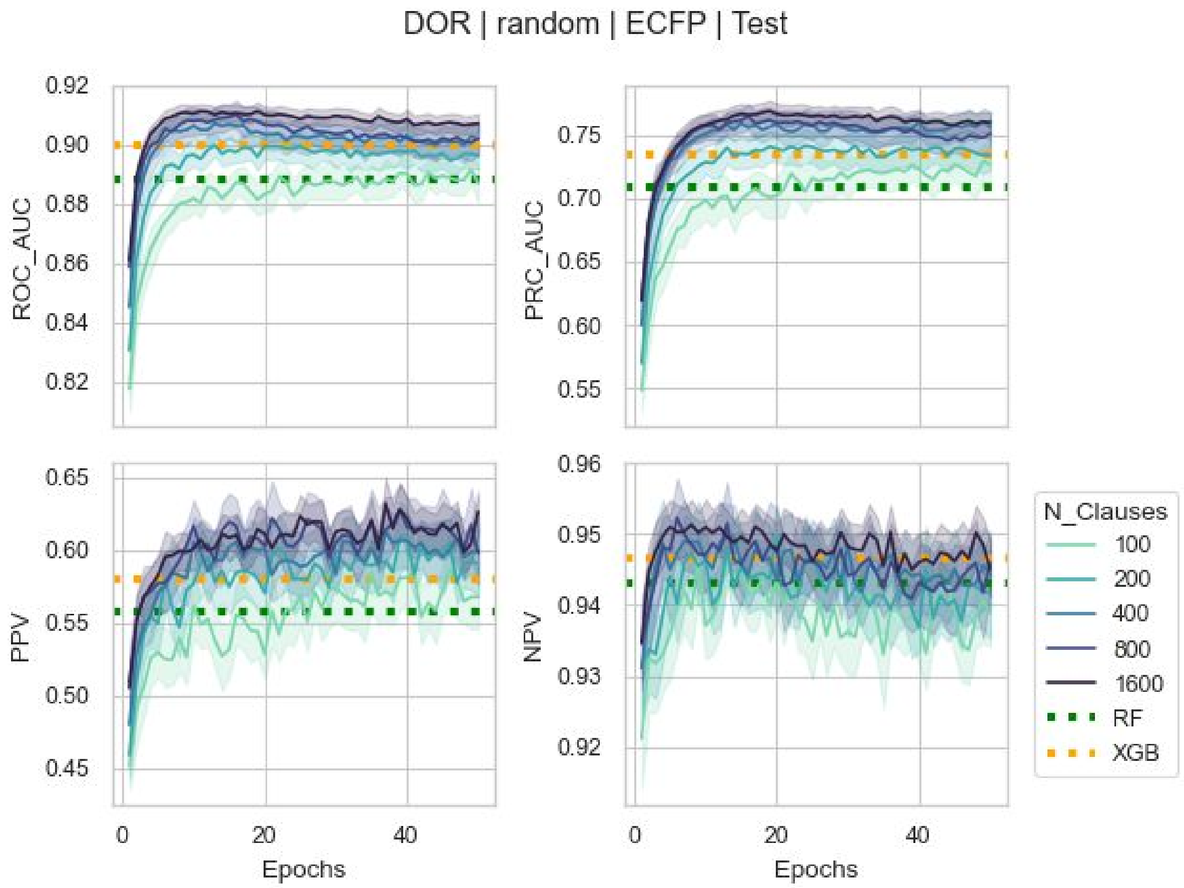

Figure S28: TM metric scores over 50 epochs on DOR test sets for random split-group and ECFP descriptors. Annotated by dotted lines are the mean test set scores of RF and XGBoost.

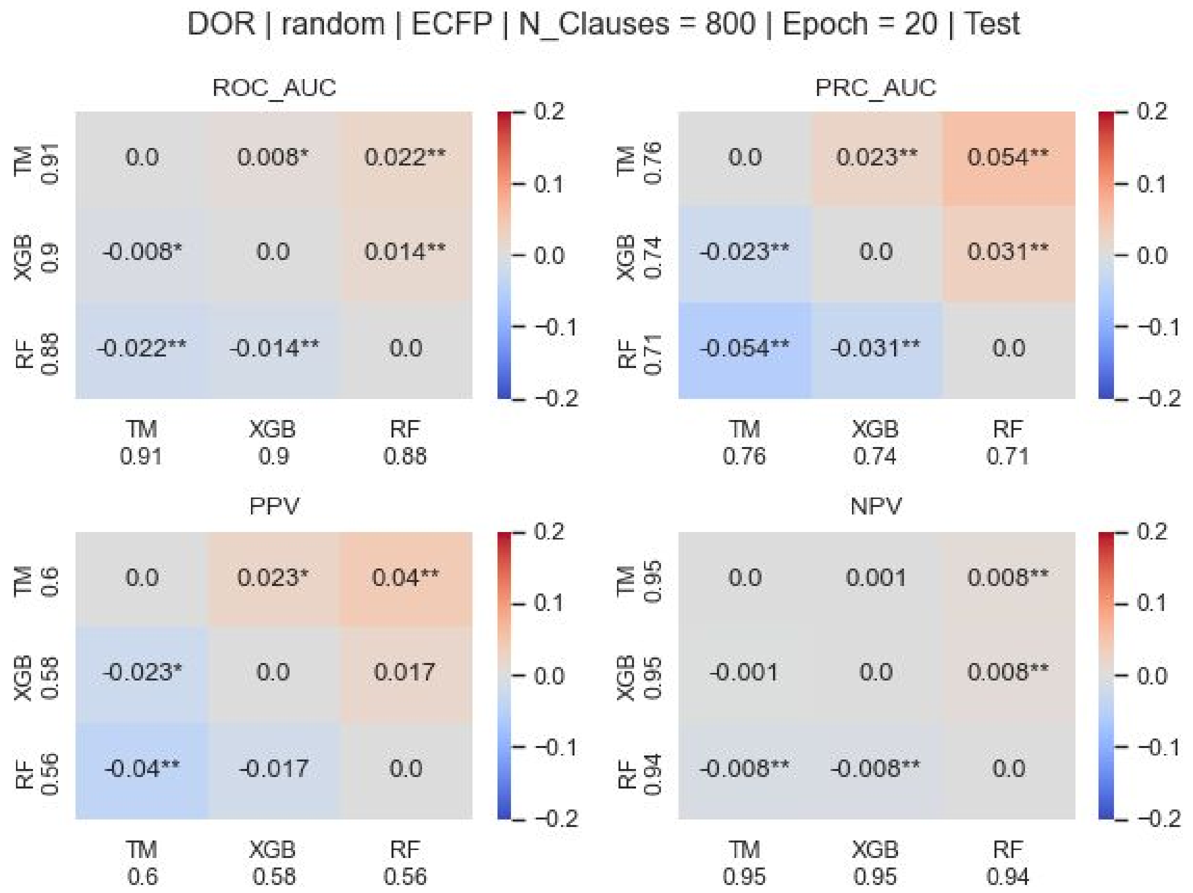

Figure S29: Cohen's D difference of means, pair-wise comparison of models for the DOR dataset with random group-split, ECFP descriptors and TM models of 800 clauses at 20 epochs. Complete with annotated statistical tests via Tukey's HSD where the number of asterix represents a different statistical significance level.

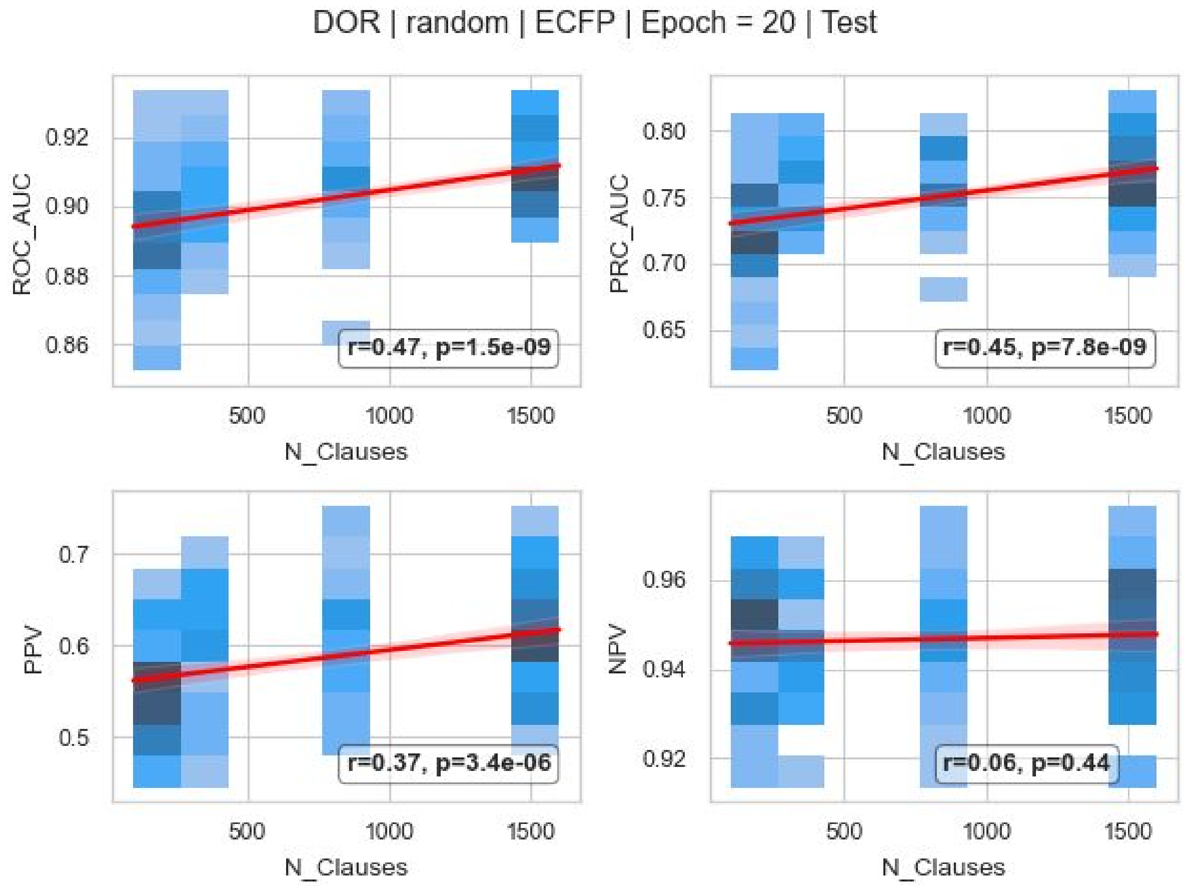

Figure S30: Number of clauses and metric-score histograms of DOR test sets with fitted line for random split-group, ECFP descriptors and TM-models of 800 clauses at 20 epochs. Pearson's R and p-value are annotated for said line.

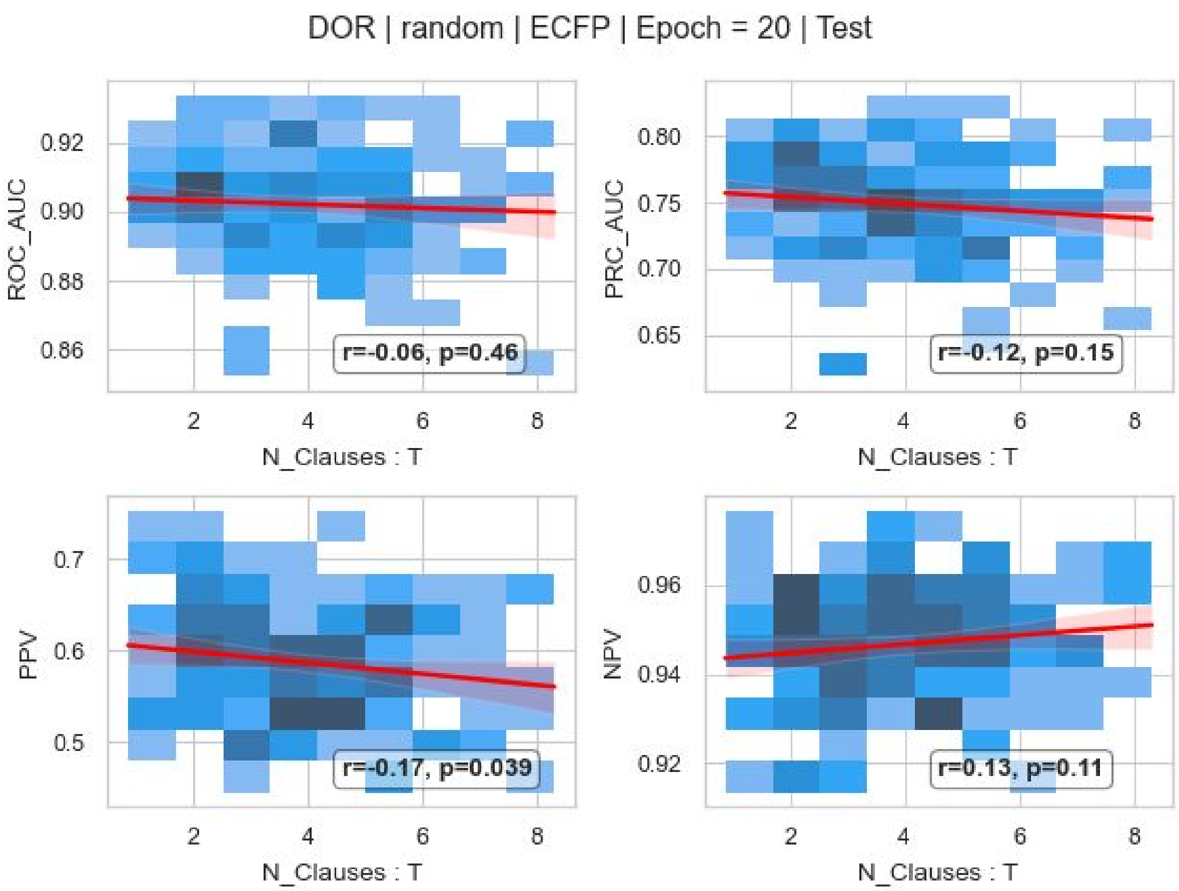

Figure S31: *N_Clauses : T* ratio and test-set metric scores histogram for DOR dataset with fitted line for random split-group, ECFP descriptors and TM-models of 800 clauses at 20 epochs. Pearson's R and p-value are annotated for said line.

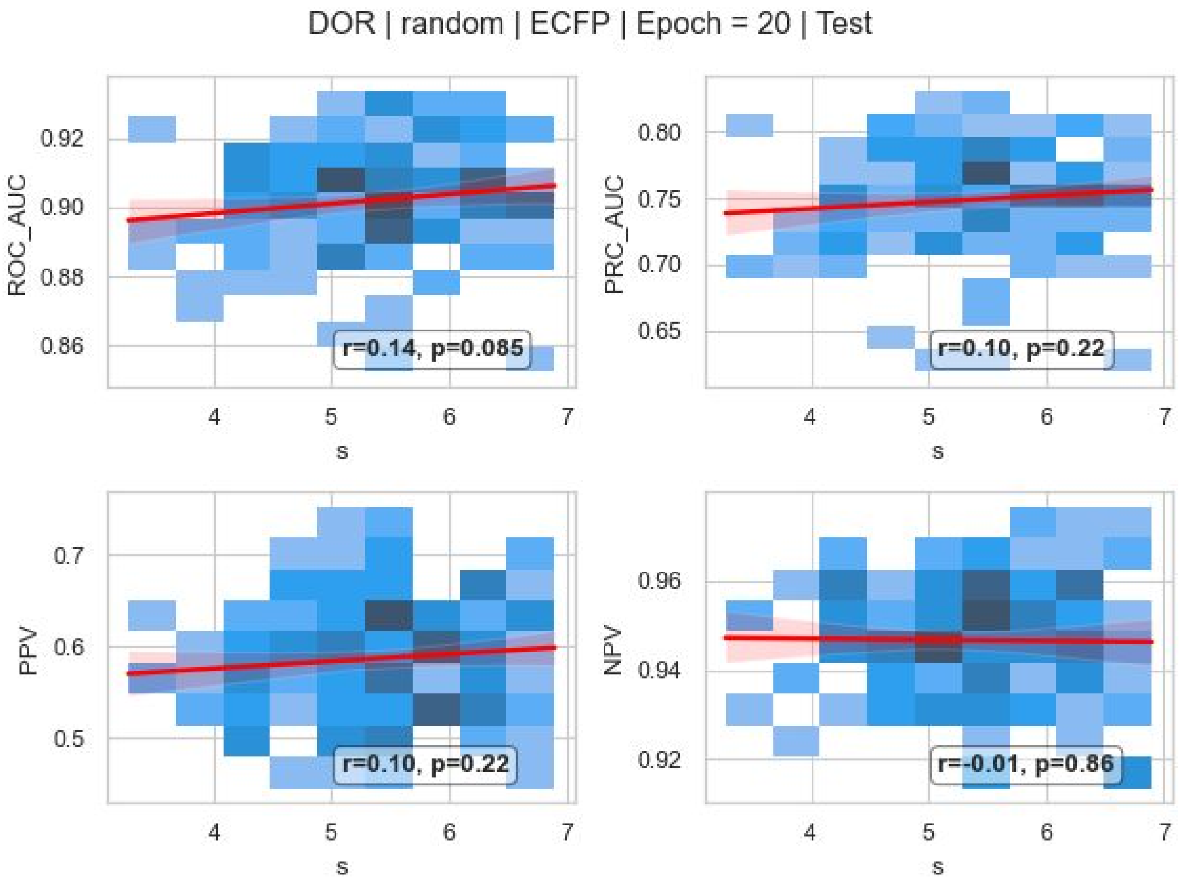

Figure S32: Hyper-parameter *s* and test-set metric scores histogram for DOR dataset with fitted line for random split-group, ECFP descriptors and TM-models of 800 clauses at 20 epochs. Pearson's R and p-value are annotated for said line.

scaffold split-group | ECFP Descriptors

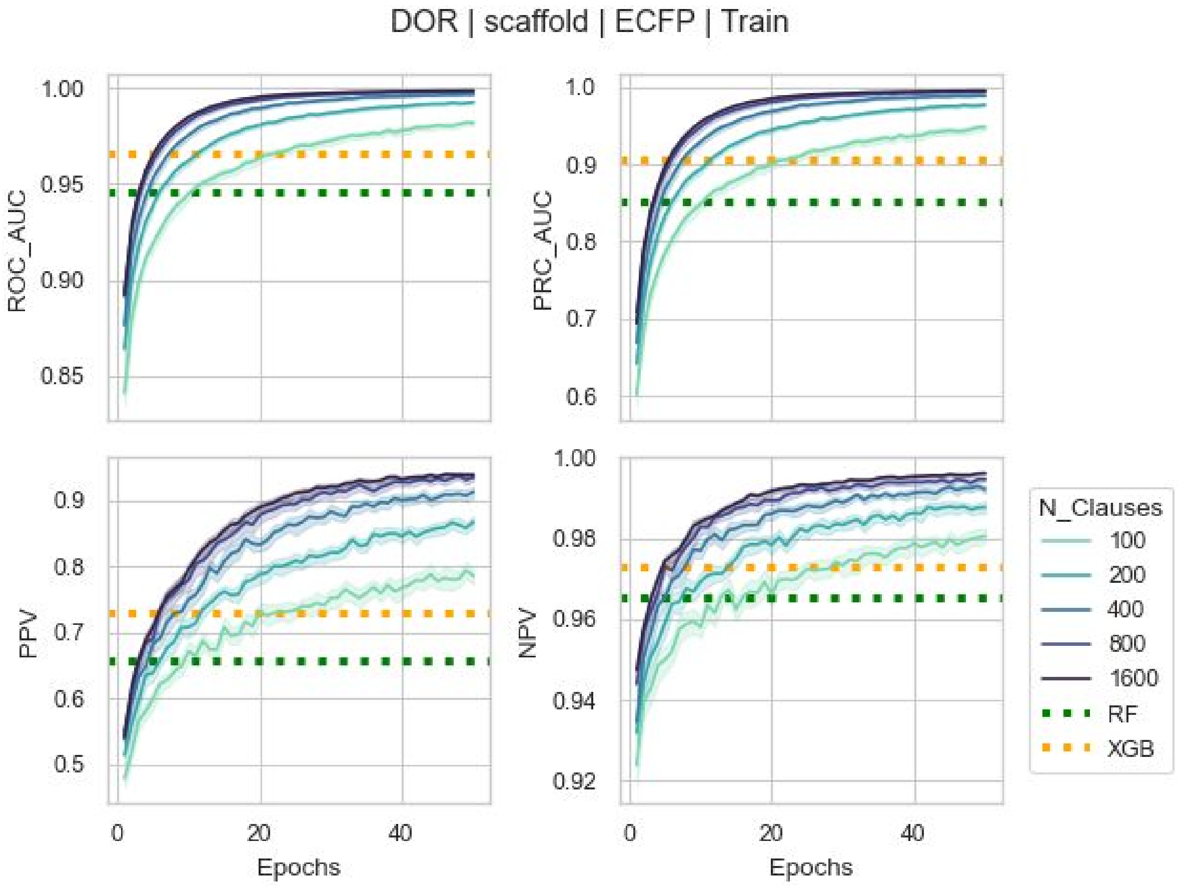

Figure S33: TM metric scores over 50 epochs on DOR training sets for scaffold split-group and ECFP descriptors. Annotated by dotted lines are the mean training set scores of RF and XGBoost.

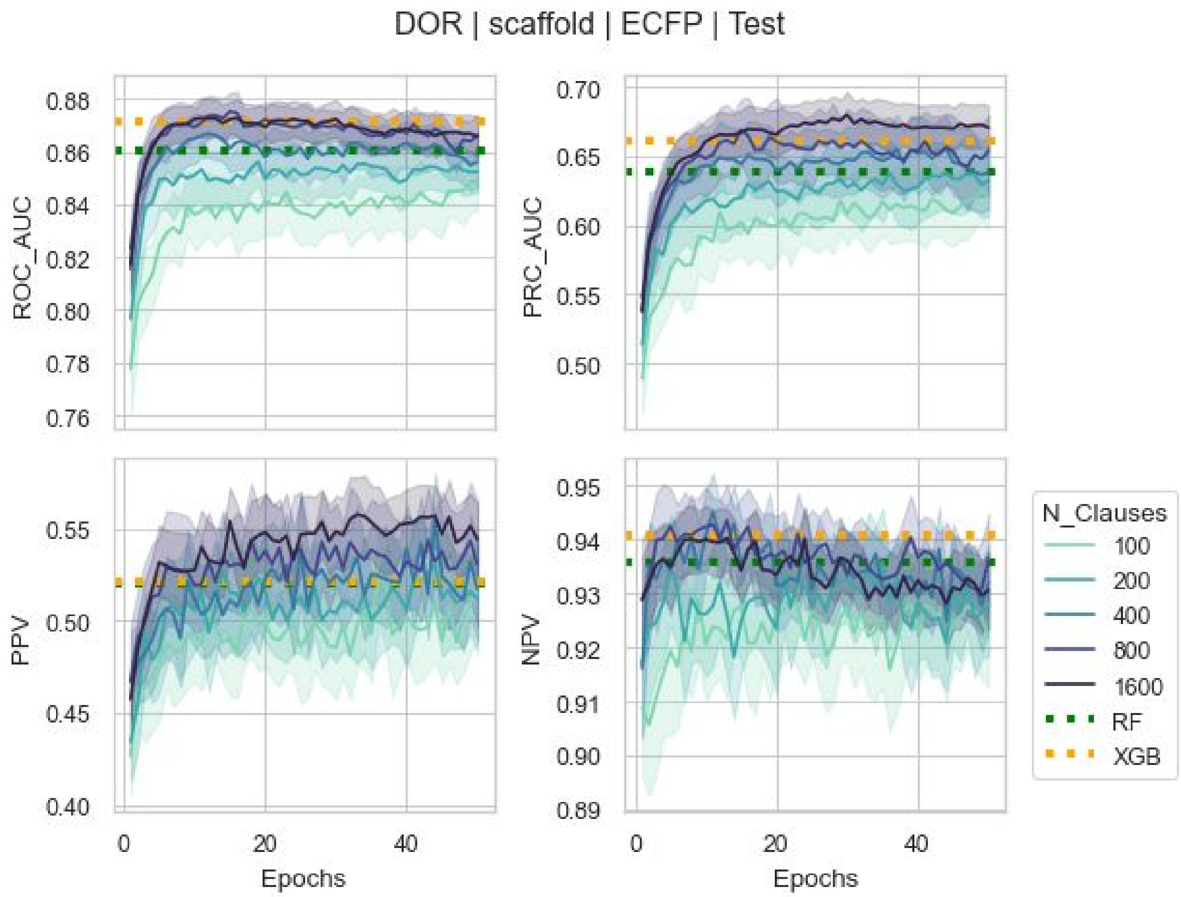

Figure S34: TM metric scores over 50 epochs on DOR test sets for scaffold split-group and ECFP descriptors. Annotated by dotted lines are the mean training set scores of RF and XGBoost.

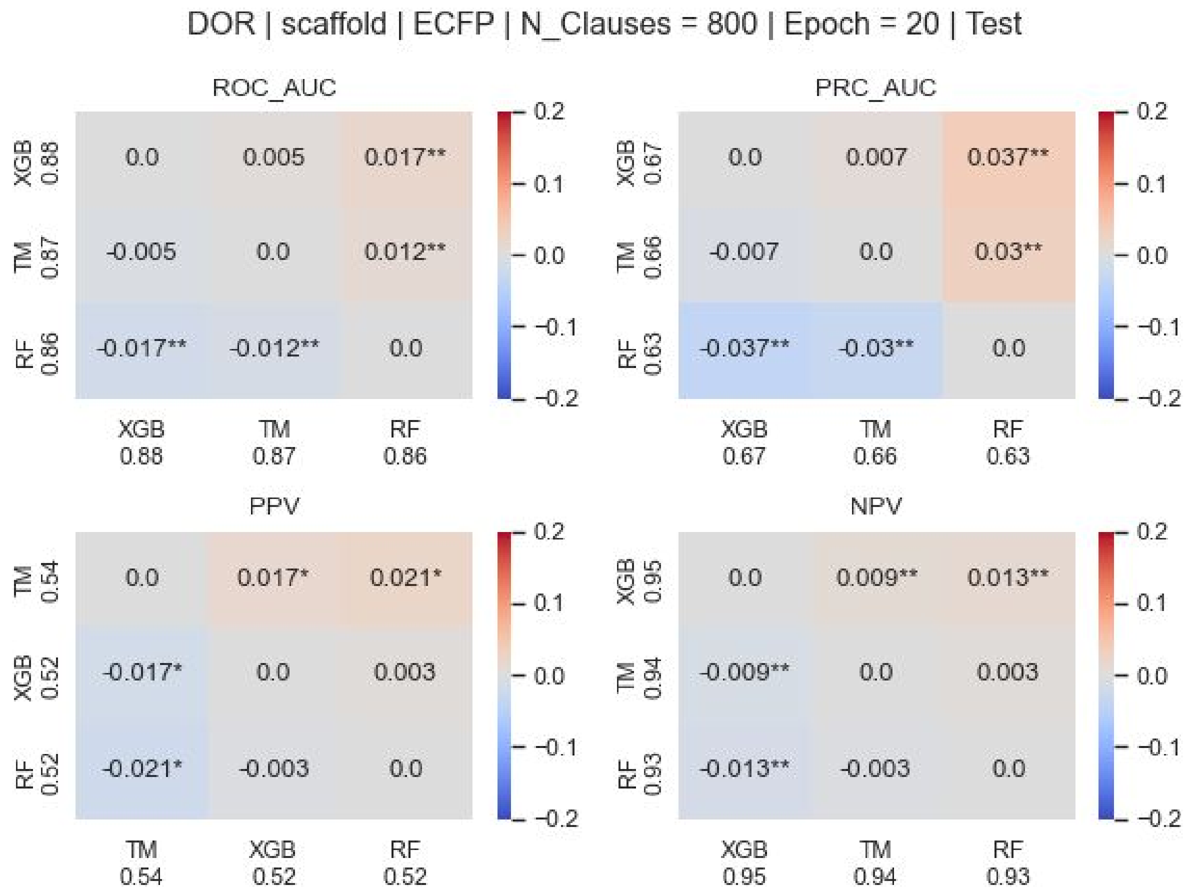

Figure S35: Cohen's D difference of means, pair-wise comparison of models for the DOR dataset with scaffold group-split, ECFP descriptors and TM models of 800 clauses at 20 epochs. Complete with annotated statistical tests via Tukey's HSD where the number of asterix represents a different statistical significance level.

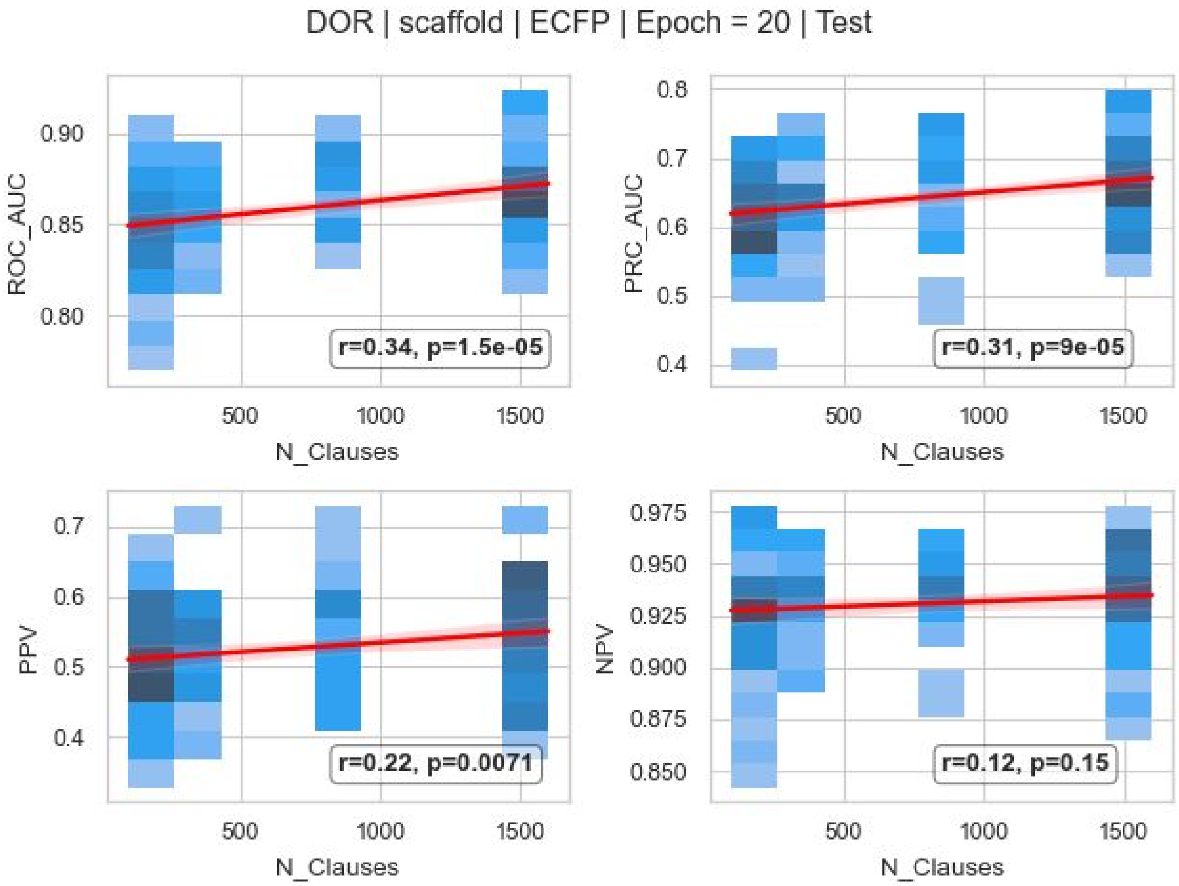

Figure S36: Number of clauses and metric-score histograms of DOR test sets with fitted line for scaffold split-group, ECFP descriptors and TM-models of 800 clauses at 20 epochs. Pearson's R and p-value are annotated for said line.

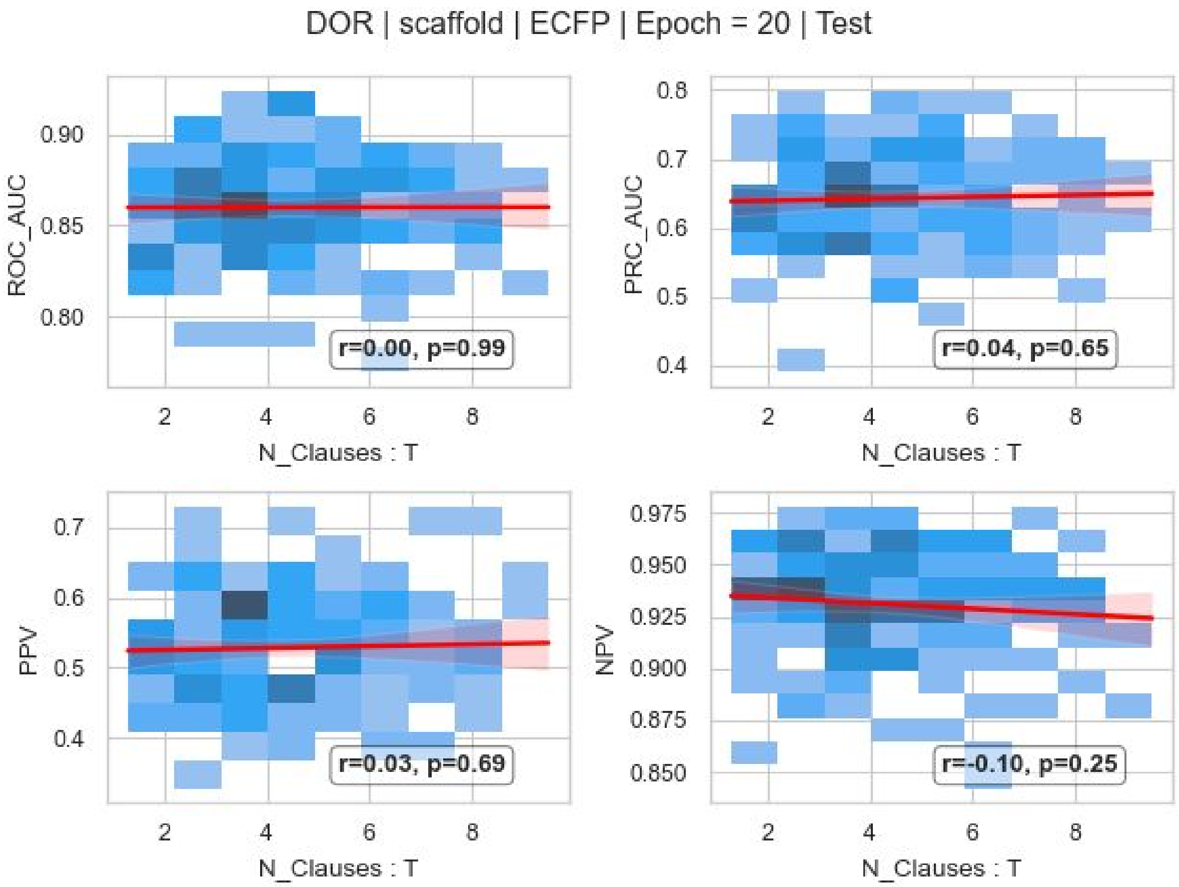

Figure S37: *N_Clauses : T* ratio and test-set metric scores histogram of DOR test sets with fitted line for scaffold split-group, ECFP descriptors and TM-models of 800 clauses at 20 epochs. Pearson's R and p-value are annotated for said line.

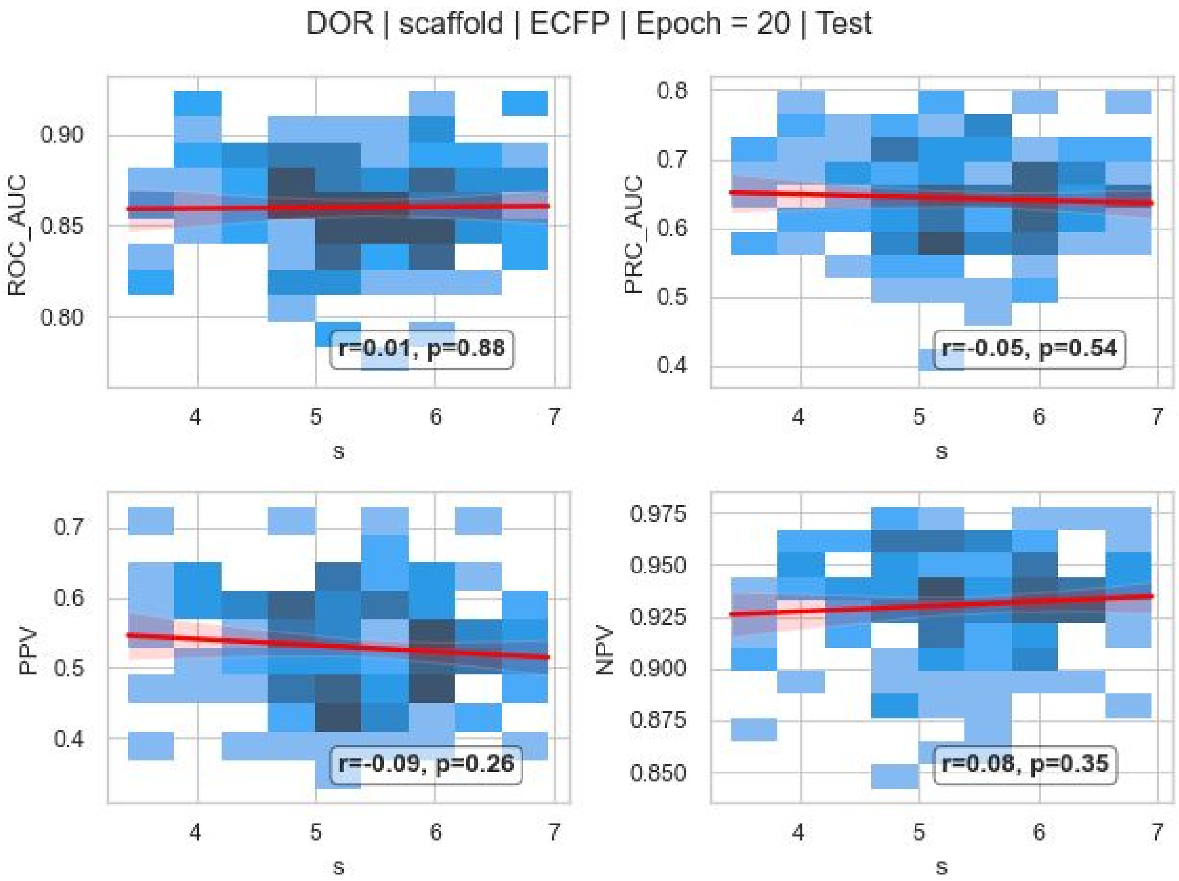

Figure S38: Hyper-parameter *s* and test-set metric scores histogram of DOR test sets with fitted line for scaffold split-group, ECFP descriptors and TM-models of 800 clauses at 20 epochs. Pearson's R and p-value are annotated for said line.

random split-group | RDKit2D Descriptors

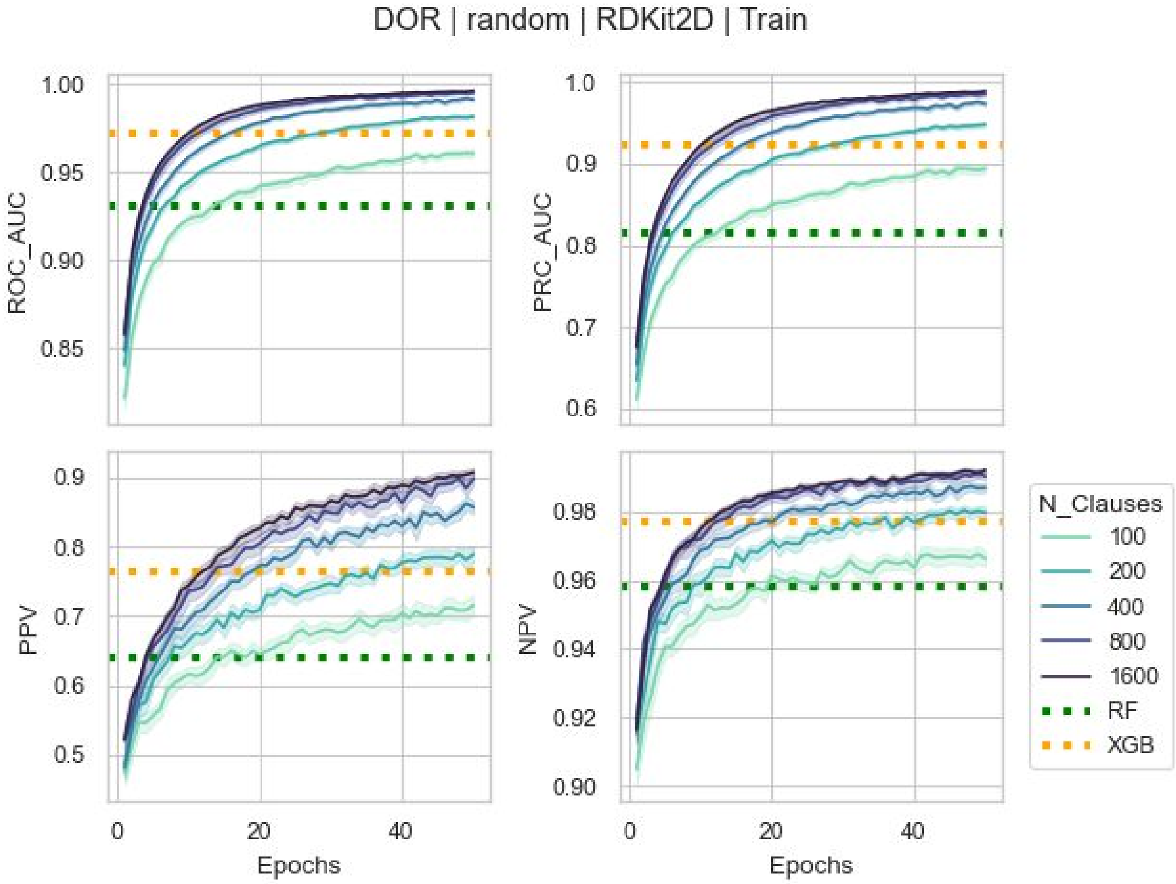

Figure S39: TM metric scores over 50 epochs on DOR training sets for random split-group and RDKit2D descriptors. Annotated by dotted lines are the mean training set scores of RF and XGBoost.

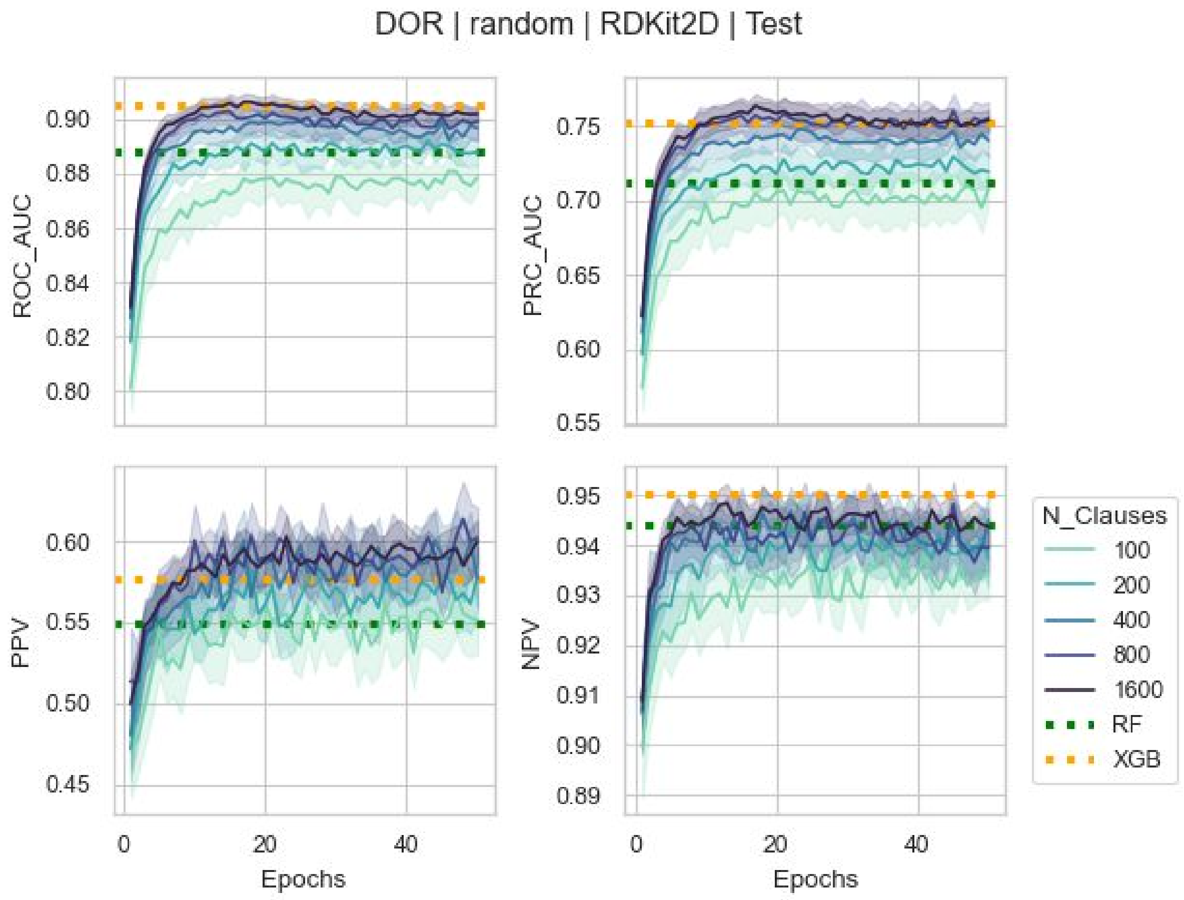

Figure S40: TM metric scores over 50 epochs on MOR test sets for random split-group and RDKit2D descriptors. Annotated by dotted lines are the mean training set scores of RF and XGBoost.

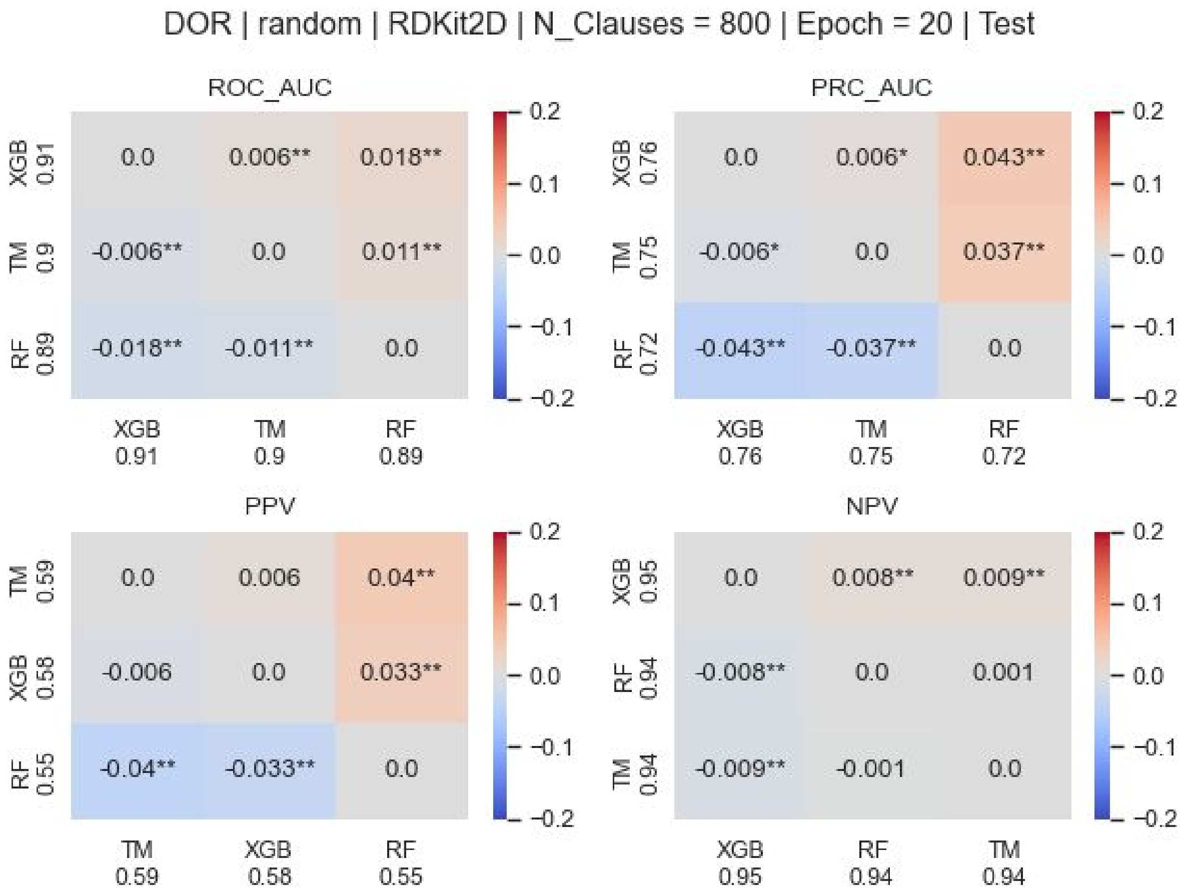

Figure S41: Cohen's D difference of means, pair-wise comparison of models for the DOR dataset with random group-split, RDKit2D descriptors and TM models of 800 clauses at 20 epochs. Complete with annotated statistical tests via Tukey's HSD where the number of asterix represents a different statistical significance level.

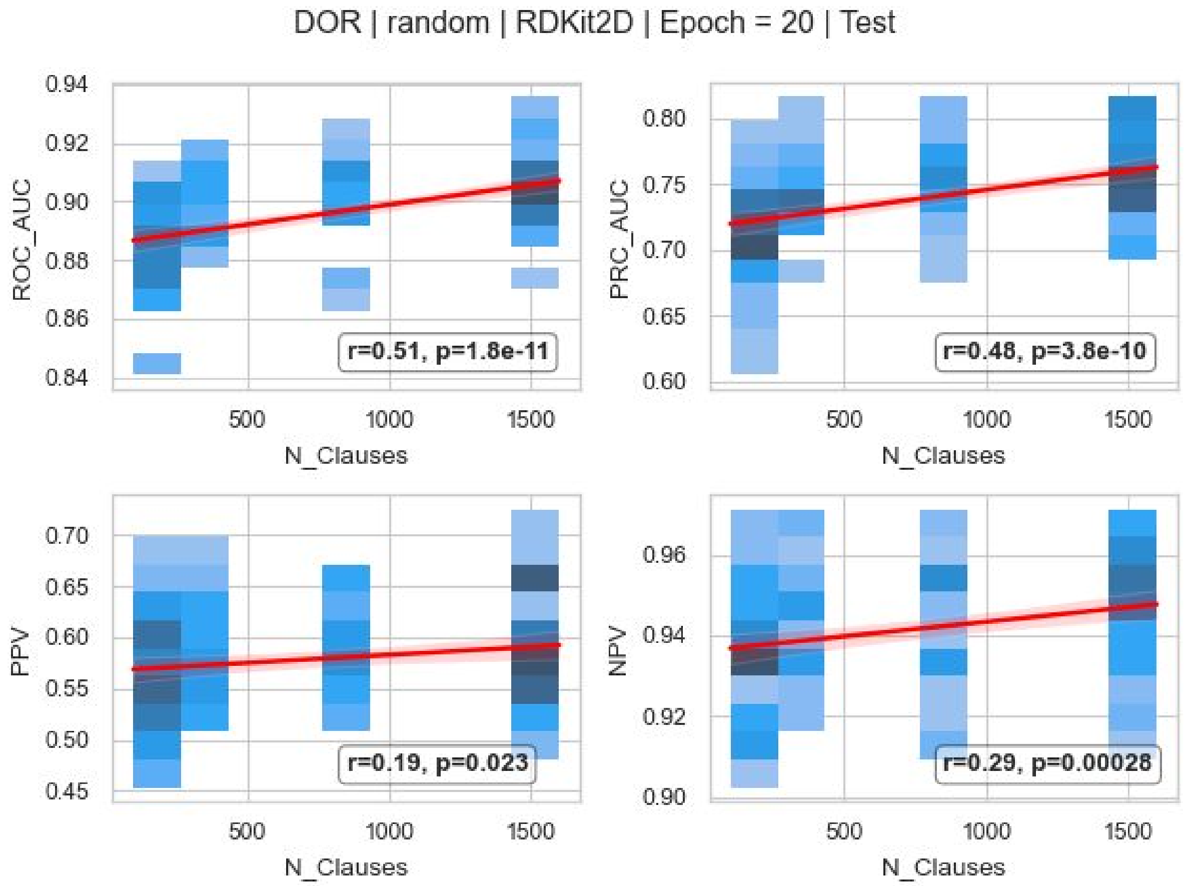

Figure S42: Number of clauses and metric-score histograms of DOR test sets with fitted line for random split-group, RDKit2D descriptors and TM-models of 800 clauses at 20 epochs. Pearson's R and p-value are annotated for said line.

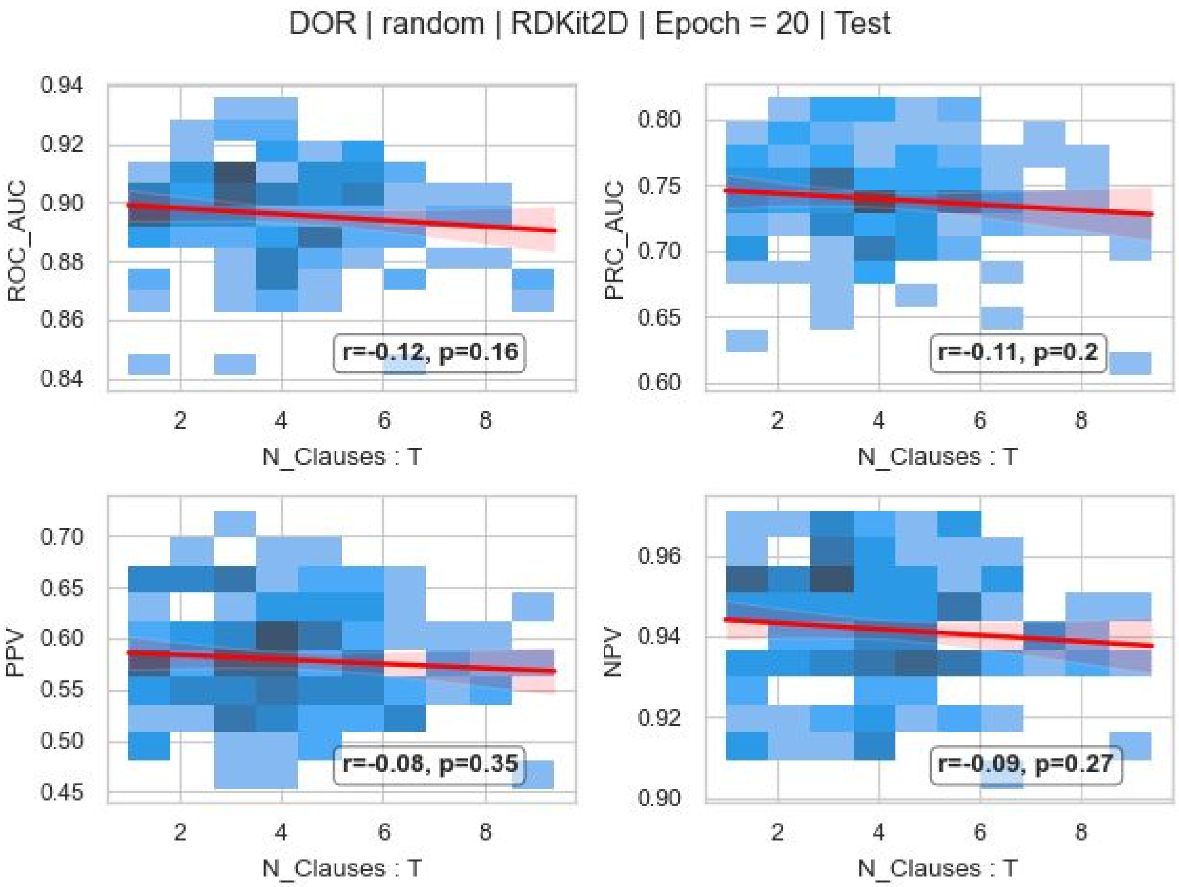

Figure S43: *N_Clauses : T* ratio and test-set metric scores histogram of DOR test sets with fitted line for random split-group, RDKit2D descriptors and TM-models of 800 clauses at 20 epochs. Pearson's R and p-value are annotated for said line.

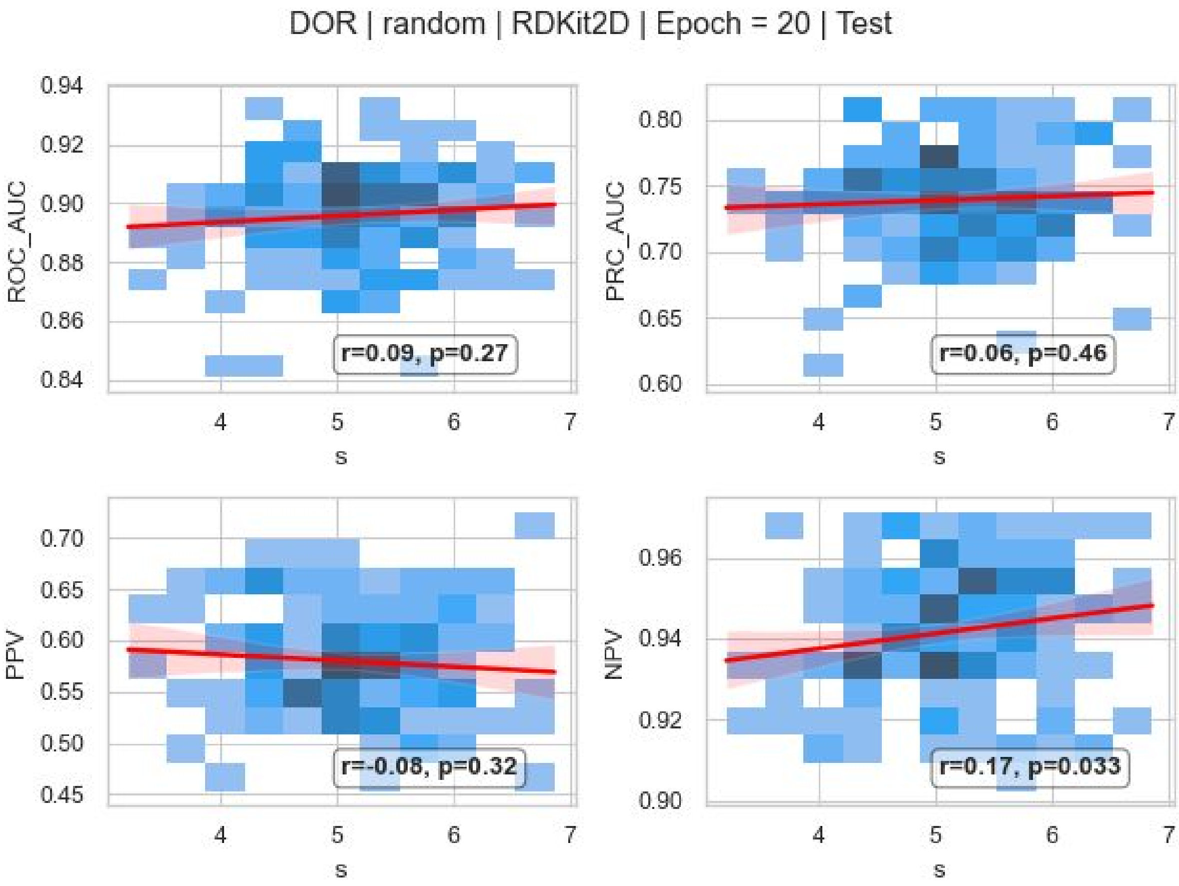

Figure S44: Hyper-parameter *s* and test-set metric scores histogram of DOR test sets with fitted line for random split-group, RDKit2D descriptors and TM-models of 800 clauses at 20 epochs. Pearson's R and p-value are annotated for said line.

scaffold group-split | RDKit2D Descriptors

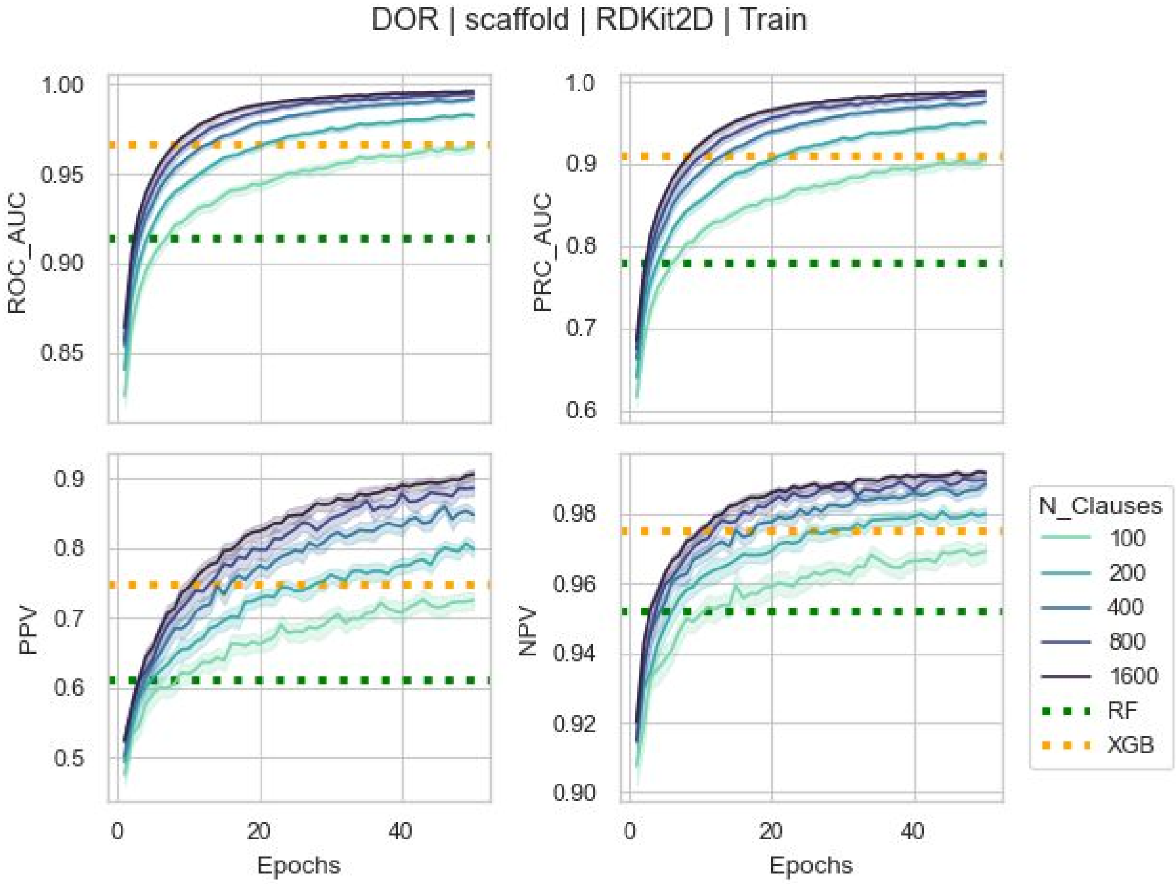

Figure S45: TM metric scores over 50 epochs on DOR training sets for scaffold split-group and RDKit2D descriptors. Annotated by dotted lines are the mean training set scores of RF and XGBoost.

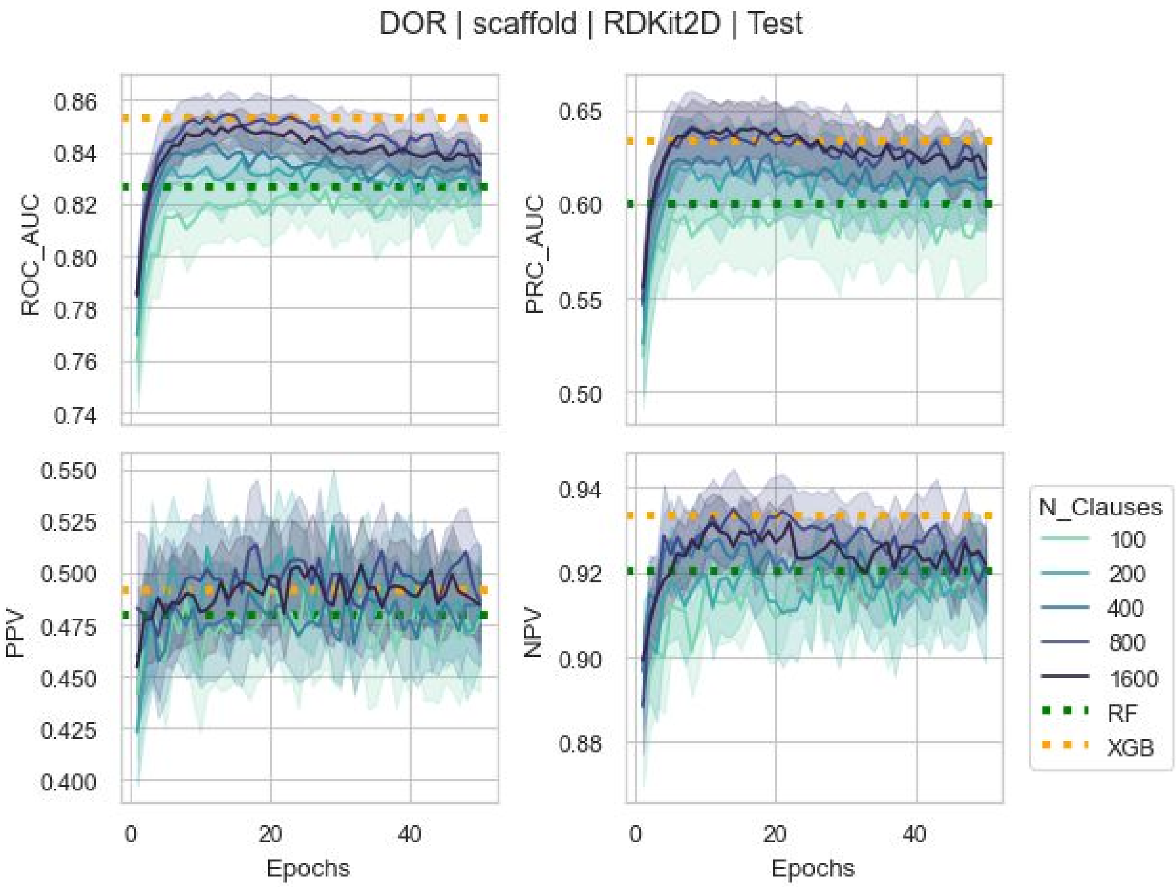

Figure S46: TM metric scores over 50 epochs on DOR test sets for scaffold split-group and RDKit2D descriptors. Annotated by dotted lines are the mean training set scores of RF and XGBoost.

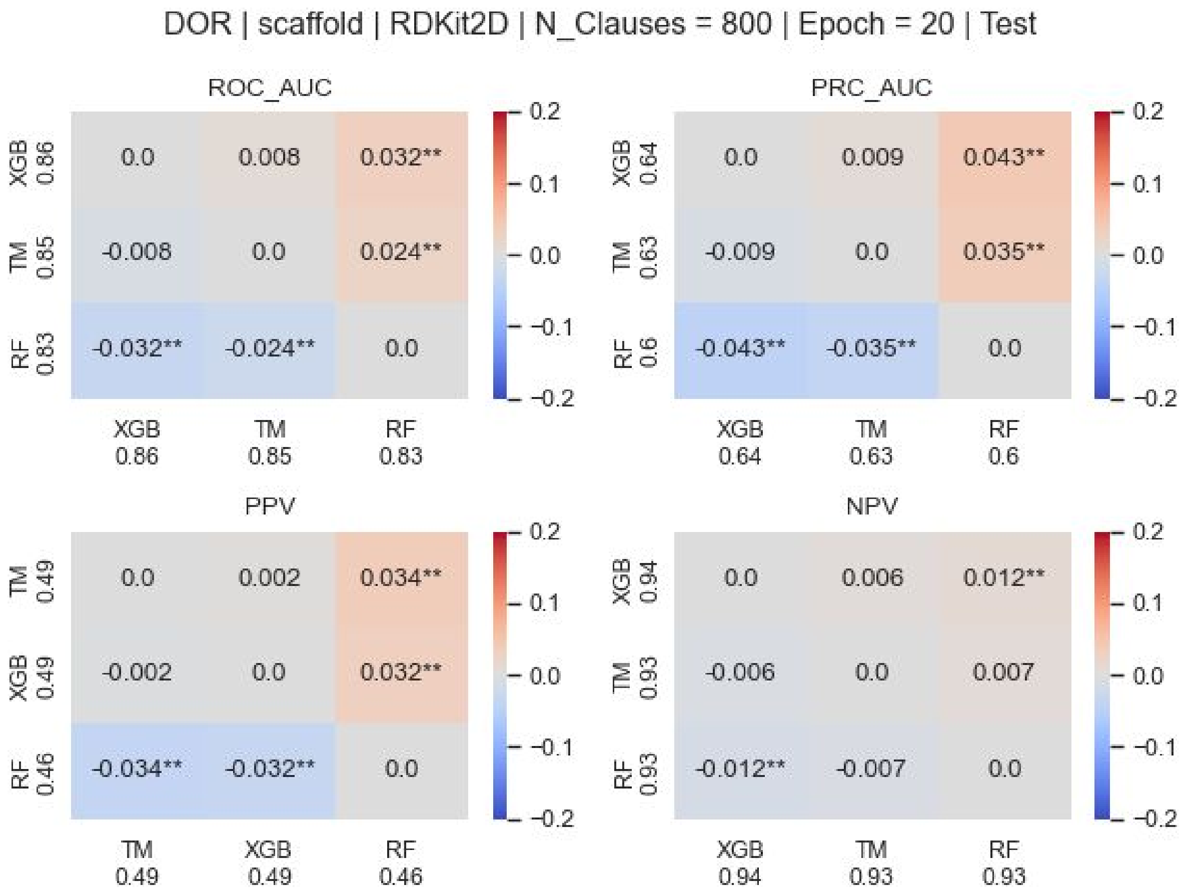

Figure S47: Cohen's D difference of means, pair-wise comparison of models for the DOR dataset with scaffold group-split, RDKit2D descriptors and TM models of 800 clauses at 20 epochs. Complete with annotated statistical tests via Tukey's HSD where the number of asterix represents a different statistical significance level.

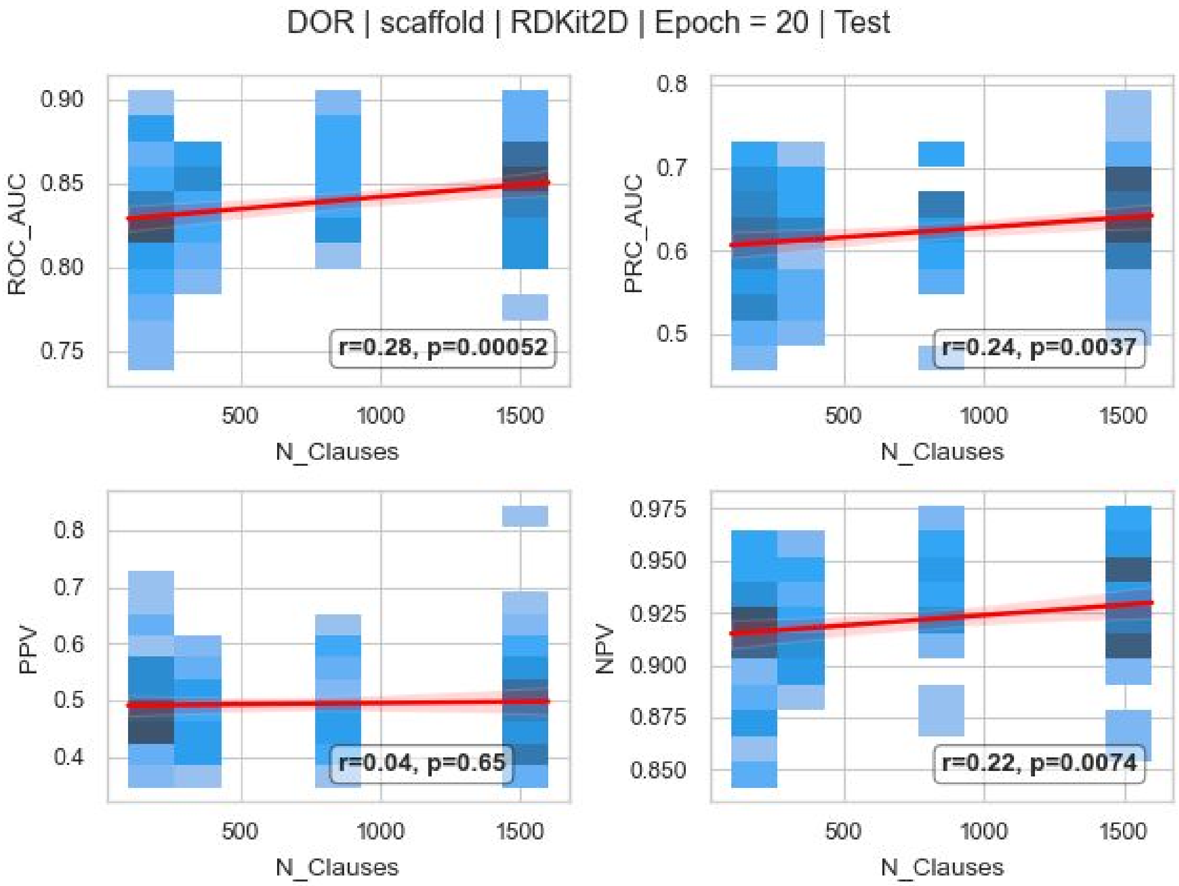

Figure S48: Number of clauses and metric-score histograms of MOR test sets with fitted line for scaffold split-group, RDKit2D descriptors and TM-models of 800 clauses at 20 epochs. Pearson's R and p-value are annotated for said line.

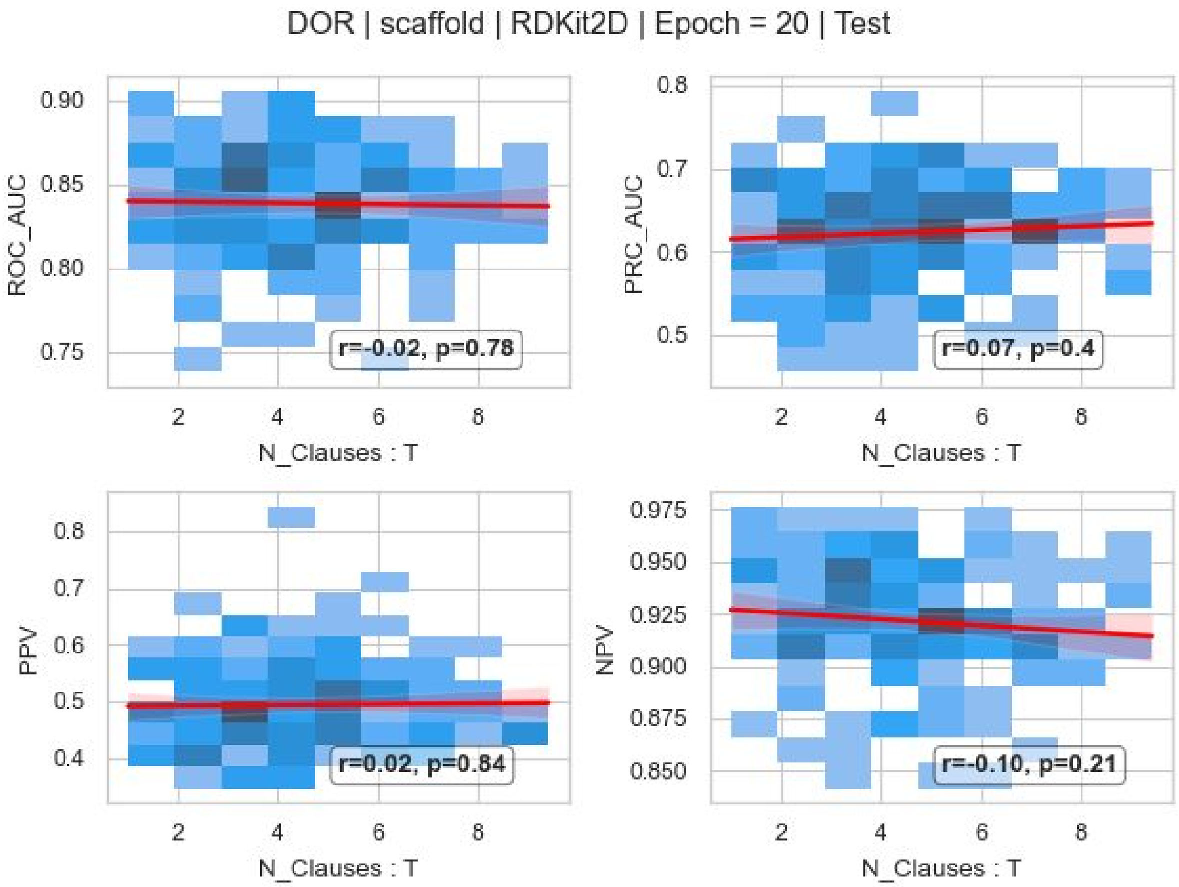

Figure S49: *N_Clauses : T* ratio and test-set metric scores histogram of MOR test sets with fitted line for scaffold split-group, RDKit2D descriptors and TM-models of 800 clauses at 20 epochs. Pearson's R and p-value are annotated for said line.

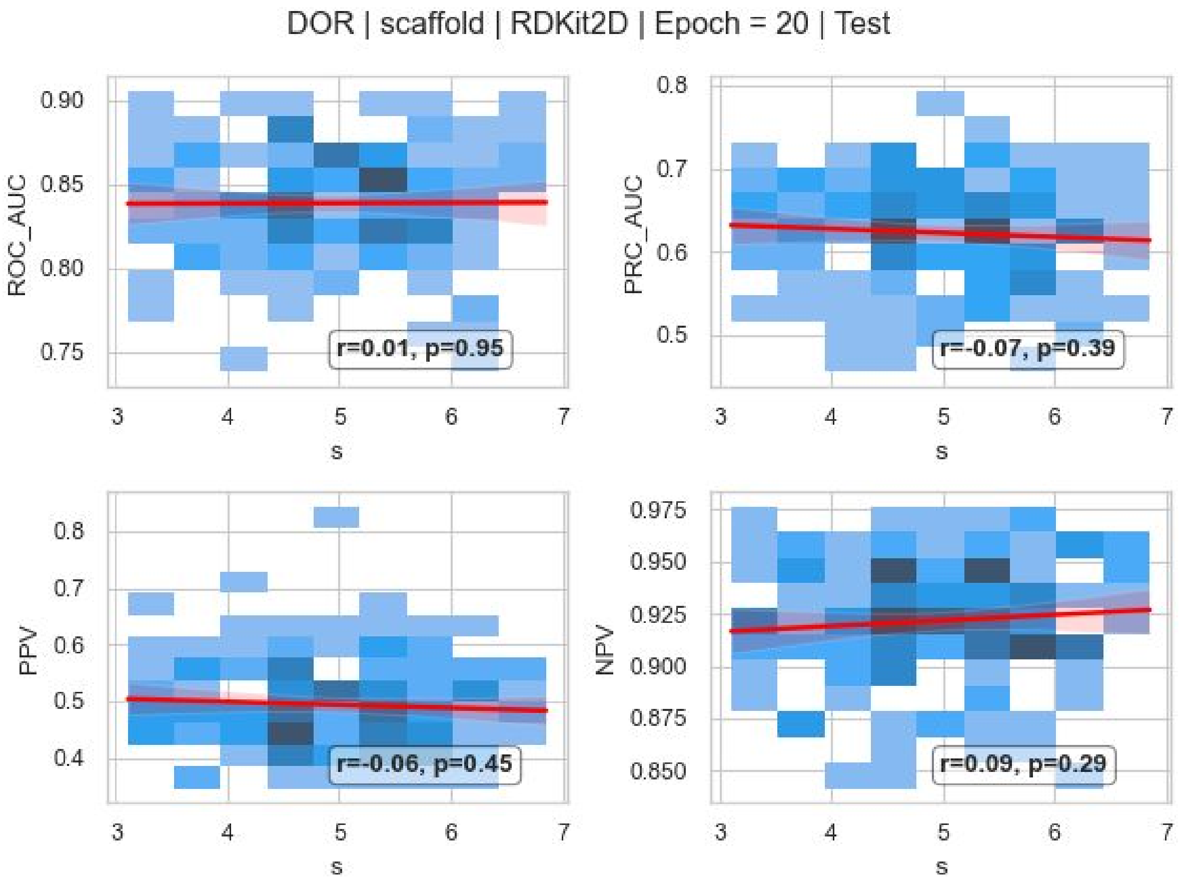

Figure S50: Hyper-parameter *s* and test-set metric scores histogram of DOR test sets with fitted line for scaffold split-group, RDKit2D descriptors and TM-models of 800 clauses at 20 epochs. Pearson's R and p-value are annotated for said line.

κ - Opioid Receptor

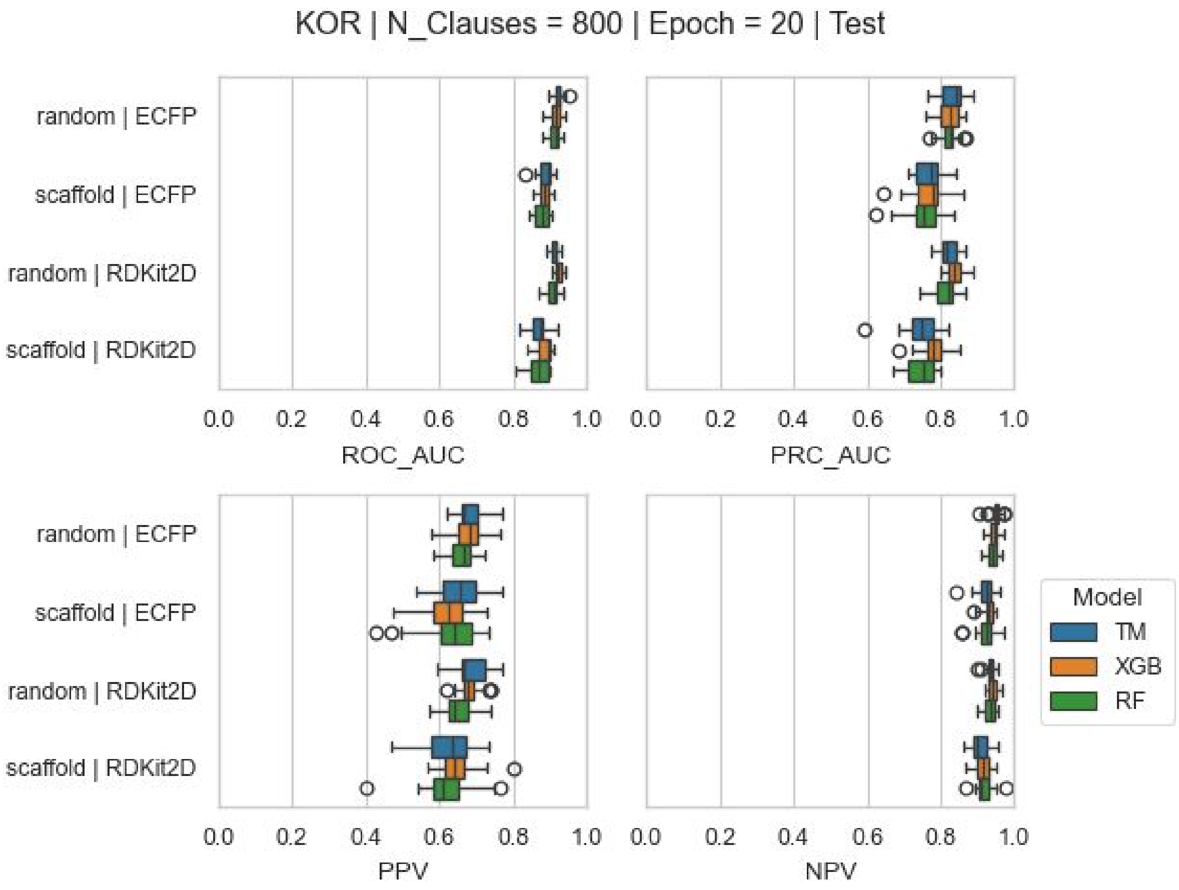

Figure S51: Box plot of model metric scores on a test set across split-group | descriptor pairs for KOR dataset. The TM uses 800 clauses and learning is stopped after 20 epochs.

random split-group I ECFP Descriptors

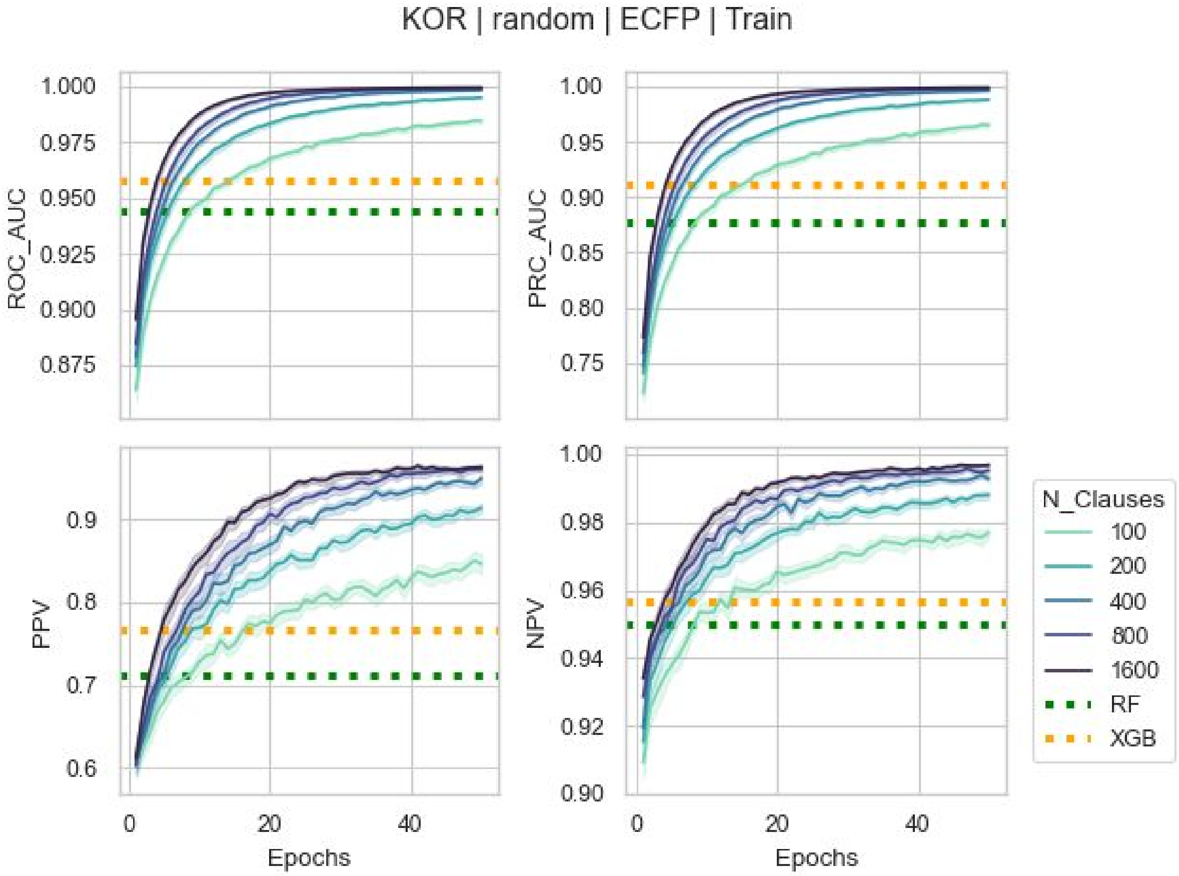

Figure S52: TM metric scores over 50 epochs on KOR training sets for random split-group and ECFP descriptors. Annotated by dotted lines are the mean training set scores of RF and XGBoost.

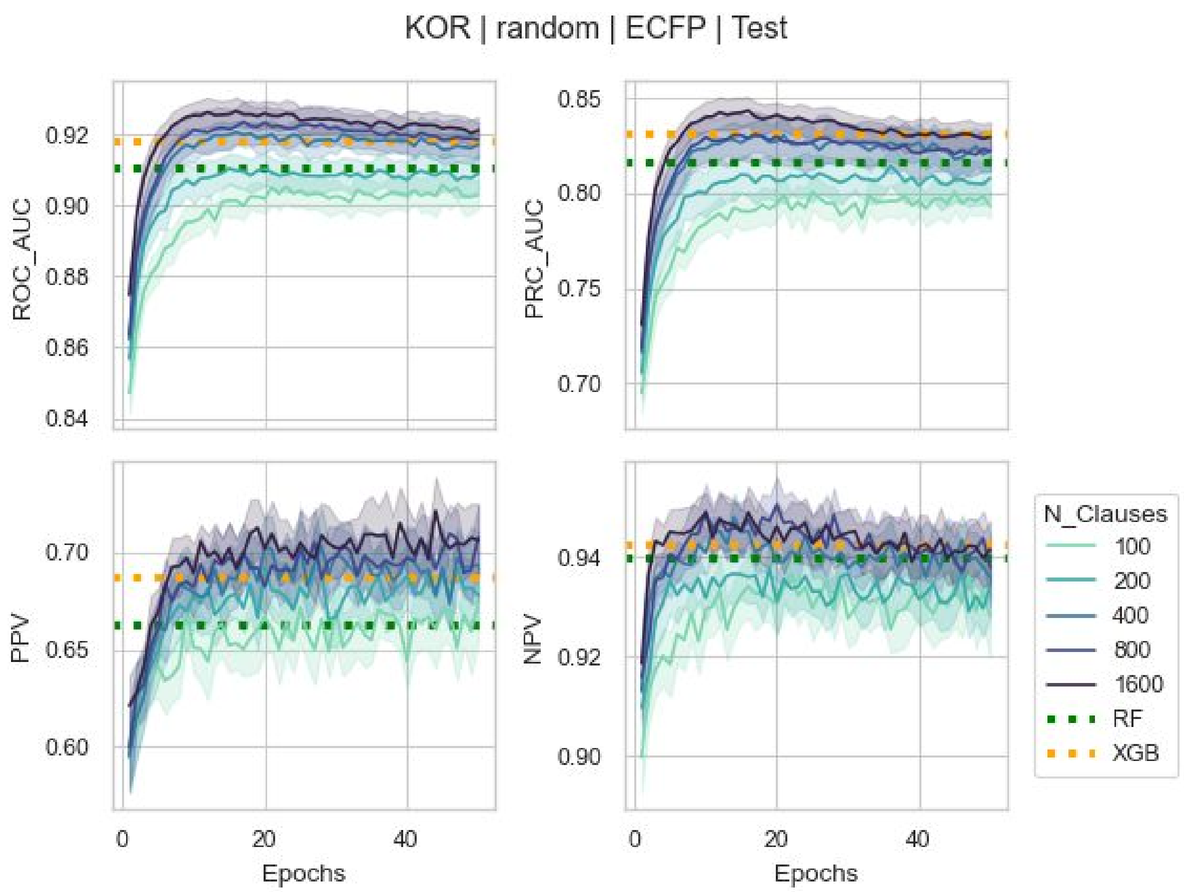

Figure S53: TM metric scores over 50 epochs on KOR test sets for random split-group and ECFP descriptors. Annotated by dotted lines are the mean test set scores of RF and XGBoost.

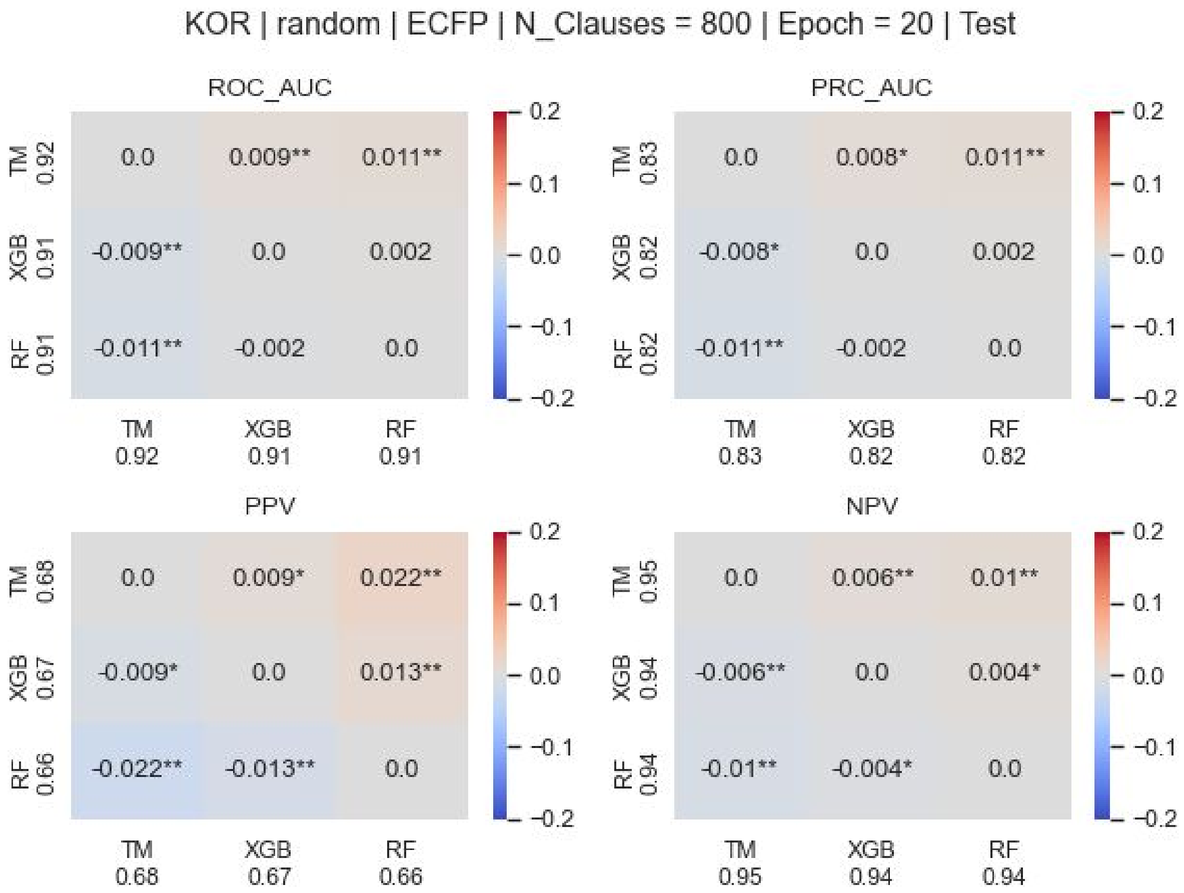

Figure S54: Cohen's D difference of means, pair-wise comparison of models for the KOR dataset with random group-split, ECFP descriptors and TM models of 800 clauses at 20 epochs. Complete with annotated statistical tests via Tukey's HSD where the number of asterix represents a different statistical significance level.

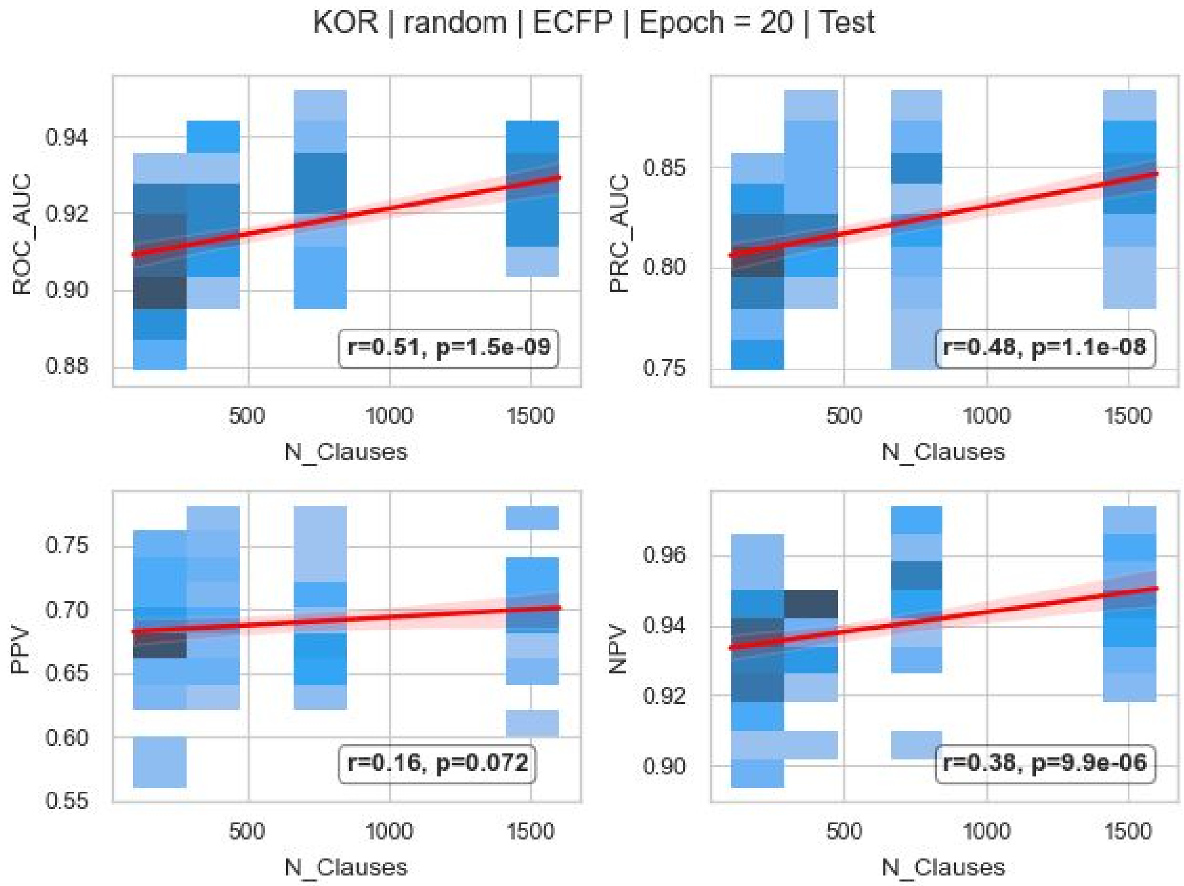

Figure S55: Number of clauses and metric-score histograms of KOR test sets with fitted line for random split-group, ECFP descriptors and TM-models of 800 clauses at 20 epochs. Pearson's R and p-value are annotated for said line.

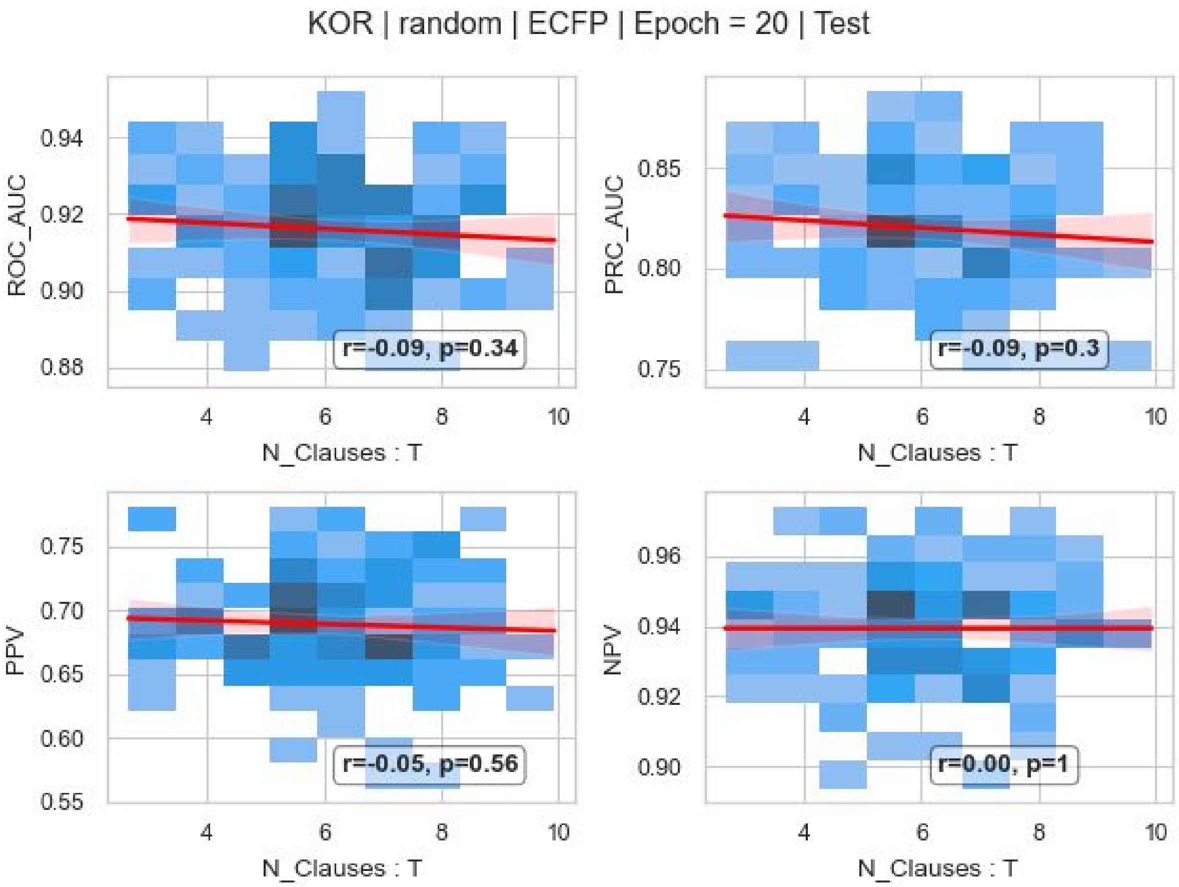

Figure S56: *N_Clauses : T* ratio and test-set metric scores histogram for KOR dataset with fitted line for random split-group, ECFP descriptors and TM-models of 800 clauses at 20 epochs. Pearson's R and p-value are annotated for said line.

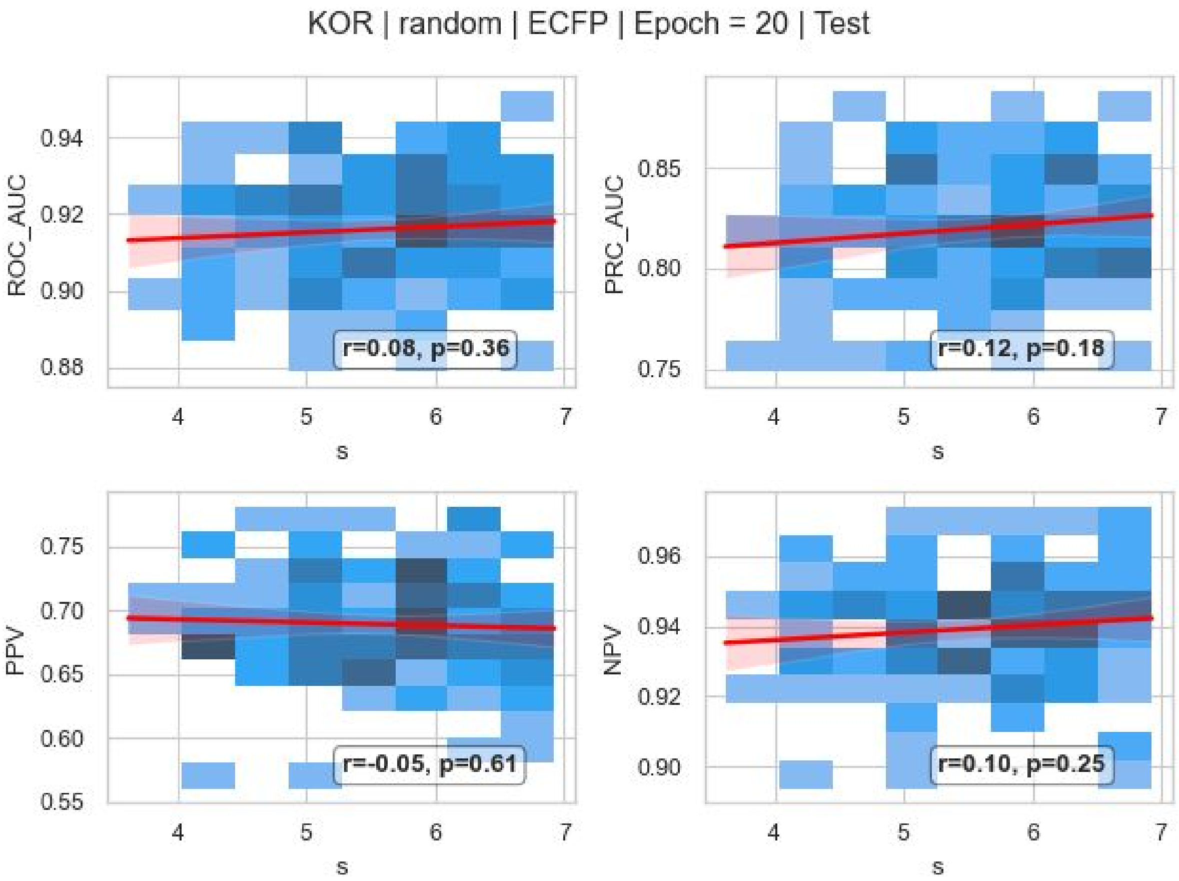

Figure S57: Hyper-parameter *s* and test-set metric scores histogram for KOR dataset with fitted line for random split-group, ECFP descriptors and TM-models of 800 clauses at 20 epochs. Pearson's R and p-value are annotated for said line.

scaffold split-group | ECFP Descriptors

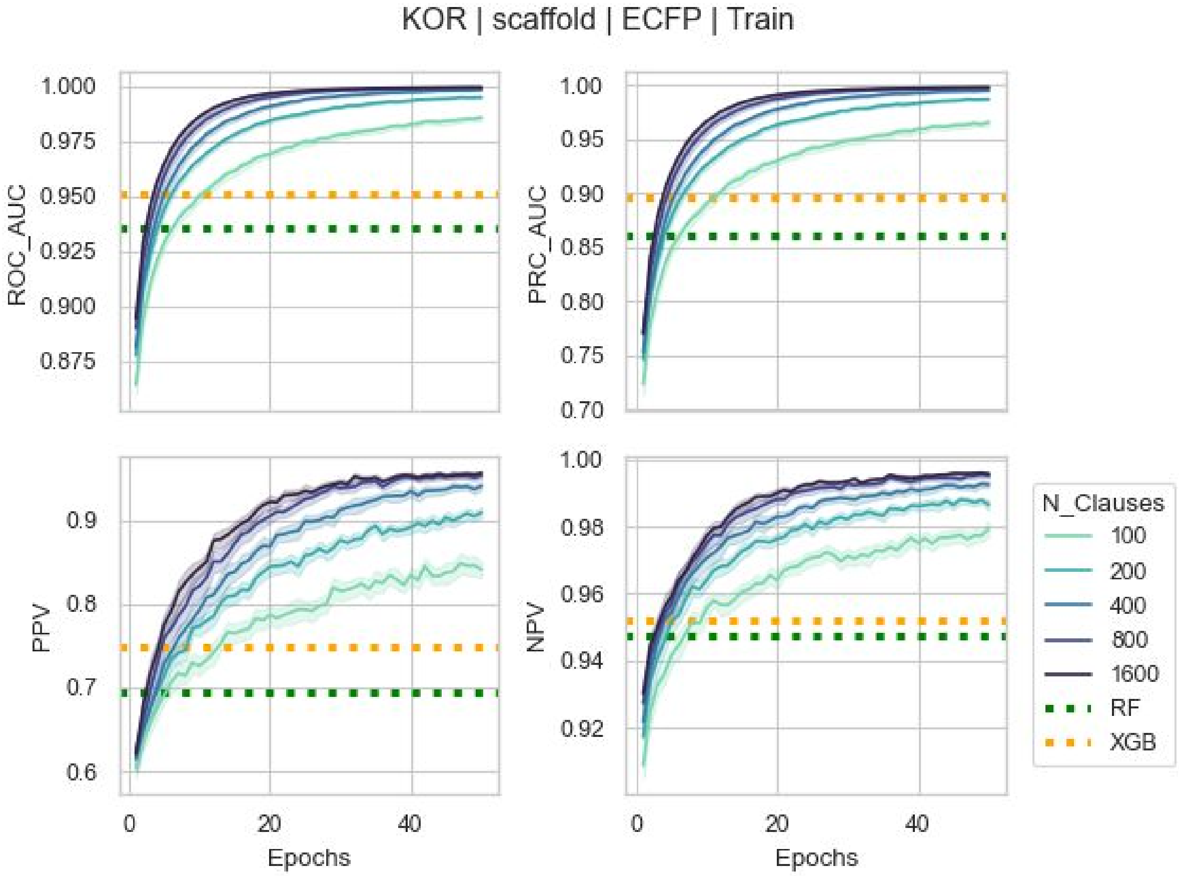

Figure S58: TM metric scores over 50 epochs on KOR training sets for scaffold split-group and ECFP descriptors. Annotated by dotted lines are the mean training set scores of RF and XGBoost.

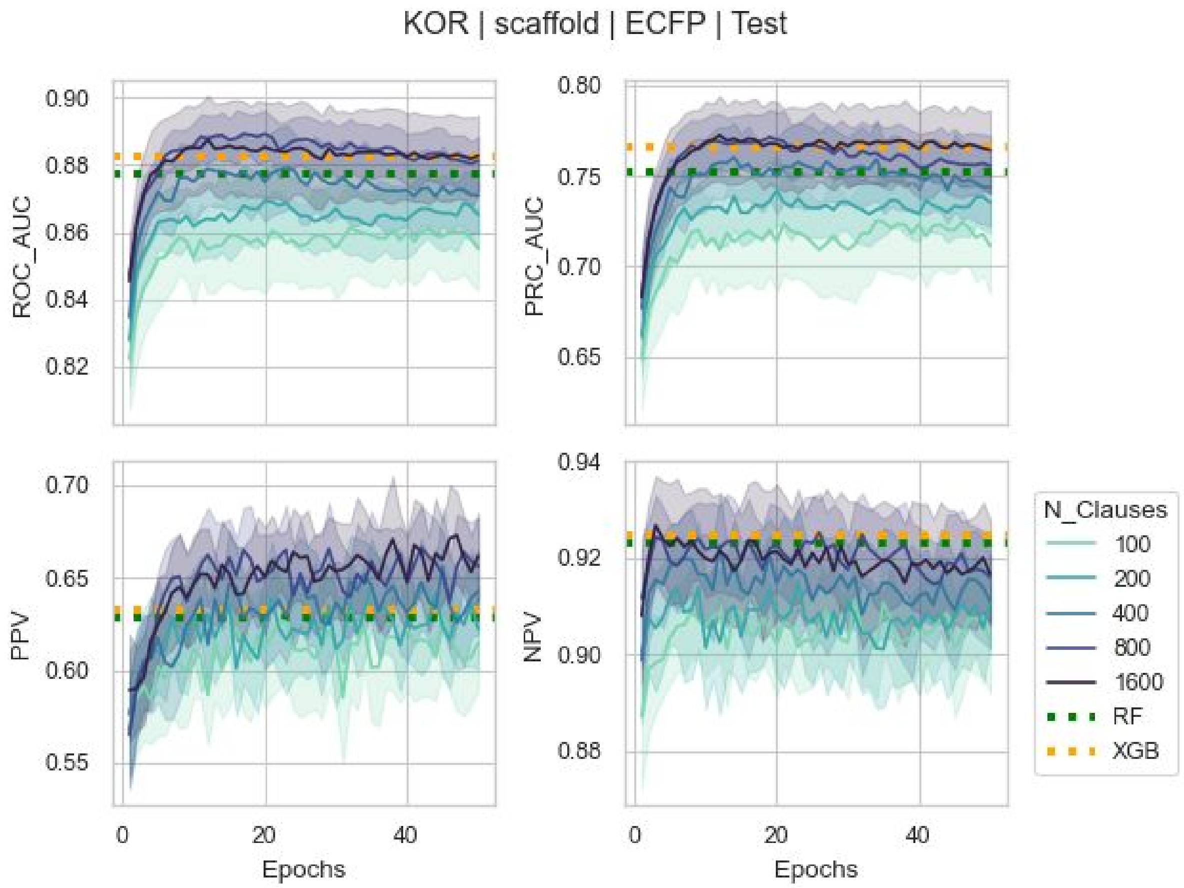

Figure S59: TM metric scores over 50 epochs on KOR test sets for scaffold split-group and ECFP descriptors. Annotated by dotted lines are the mean training set scores of RF and XGBoost.

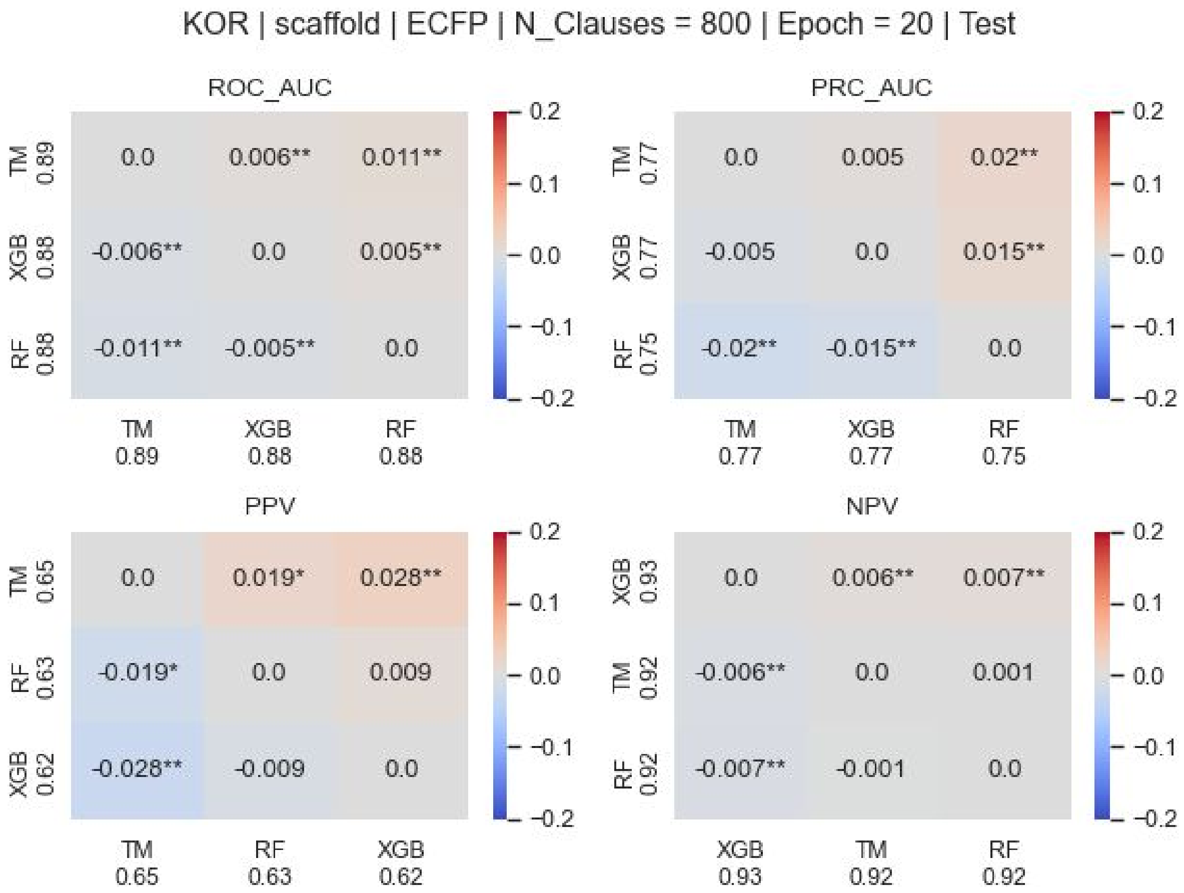

Figure S60: Cohen’s D difference of means, pair-wise comparison of models for the KOR dataset with scaffold group-split, ECFP descriptors and TM models of 800 clauses at 20 epochs. Complete with annotated statistical tests via Tukey’s HSD where the number of asterix represents a different statistical significance level.

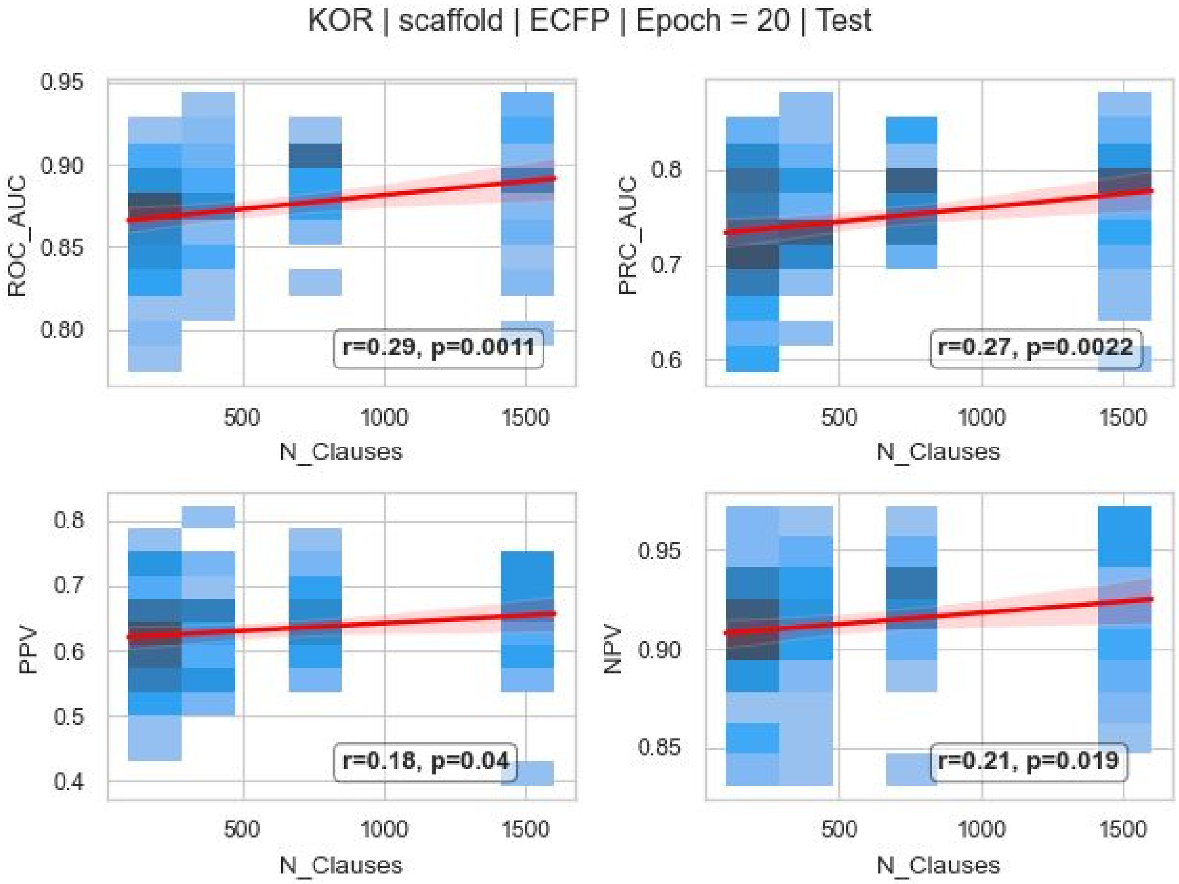

Figure S61: Number of clauses and metric-score histograms of KOR test sets with fitted line for scaffold split-group, ECFP descriptors and TM-models of 800 clauses at 20 epochs. Pearson's R and p-value are annotated for said line.

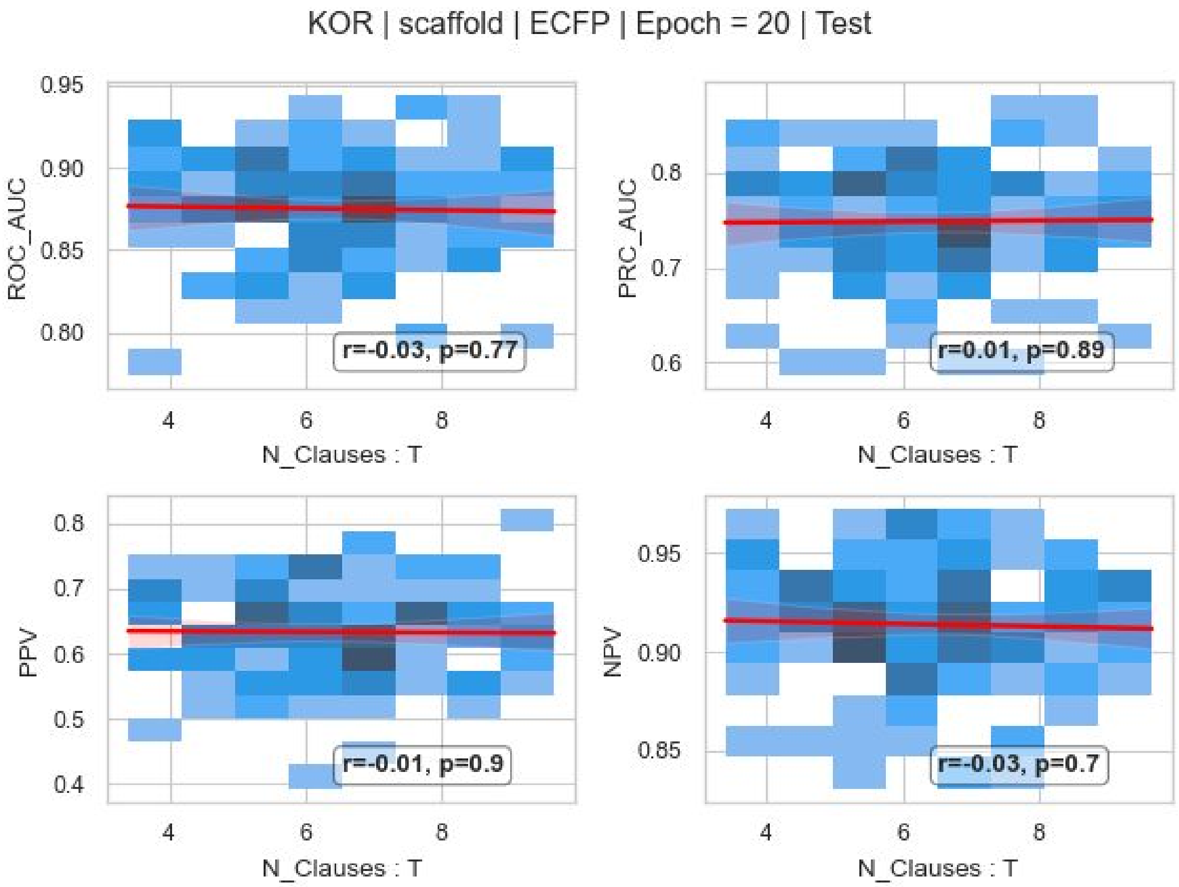

Figure S62: *N_Clauses : T* ratio and test-set metric scores histogram of KOR test sets with fitted line for scaffold split-group, ECFP descriptors and TM-models of 800 clauses at 20 epochs. Pearson's R and p-value are annotated for said line.

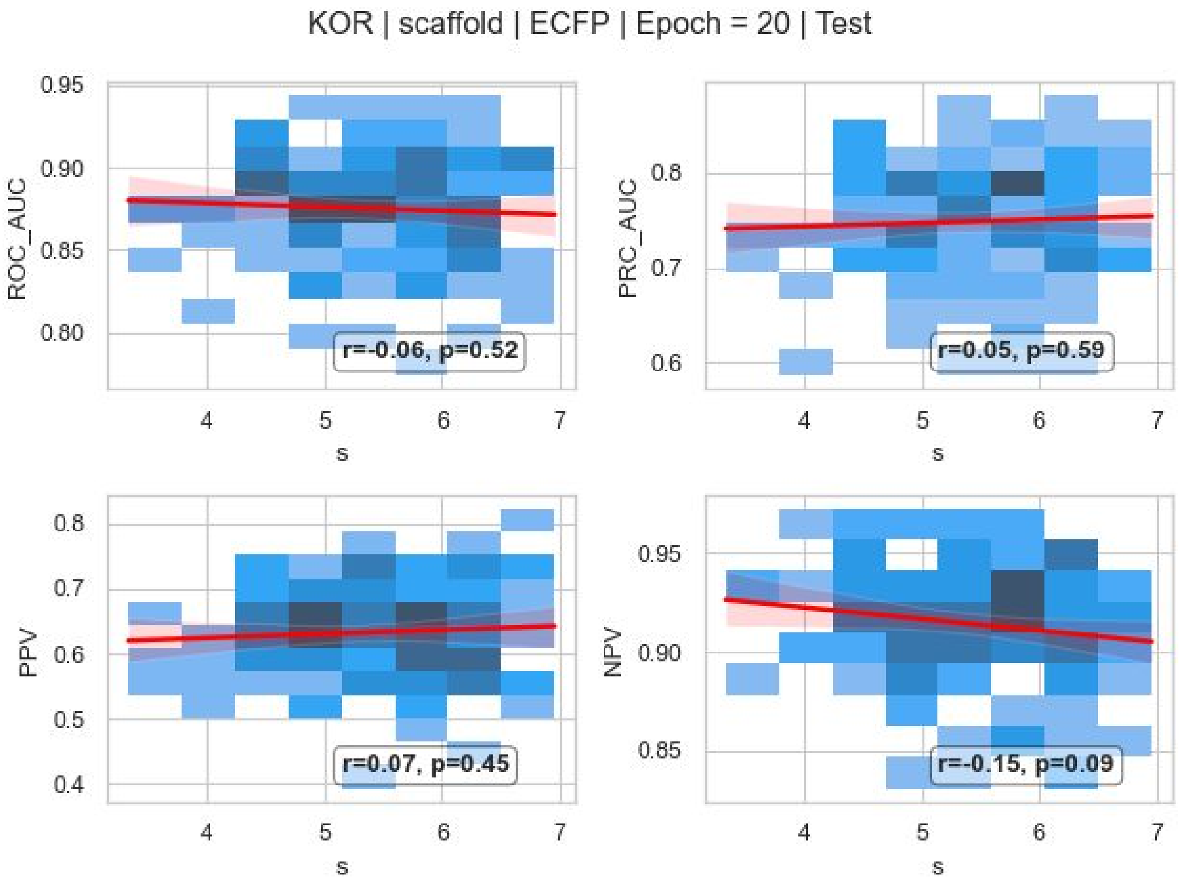

Figure S63: Hyper-parameter *s* and test-set metric scores histogram of KOR test sets with fitted line for scaffold split-group, ECFP descriptors and TM-models of 800 clauses at 20 epochs. Pearson's R and p-value are annotated for said line.

random split-group | RDKit2D Descriptors

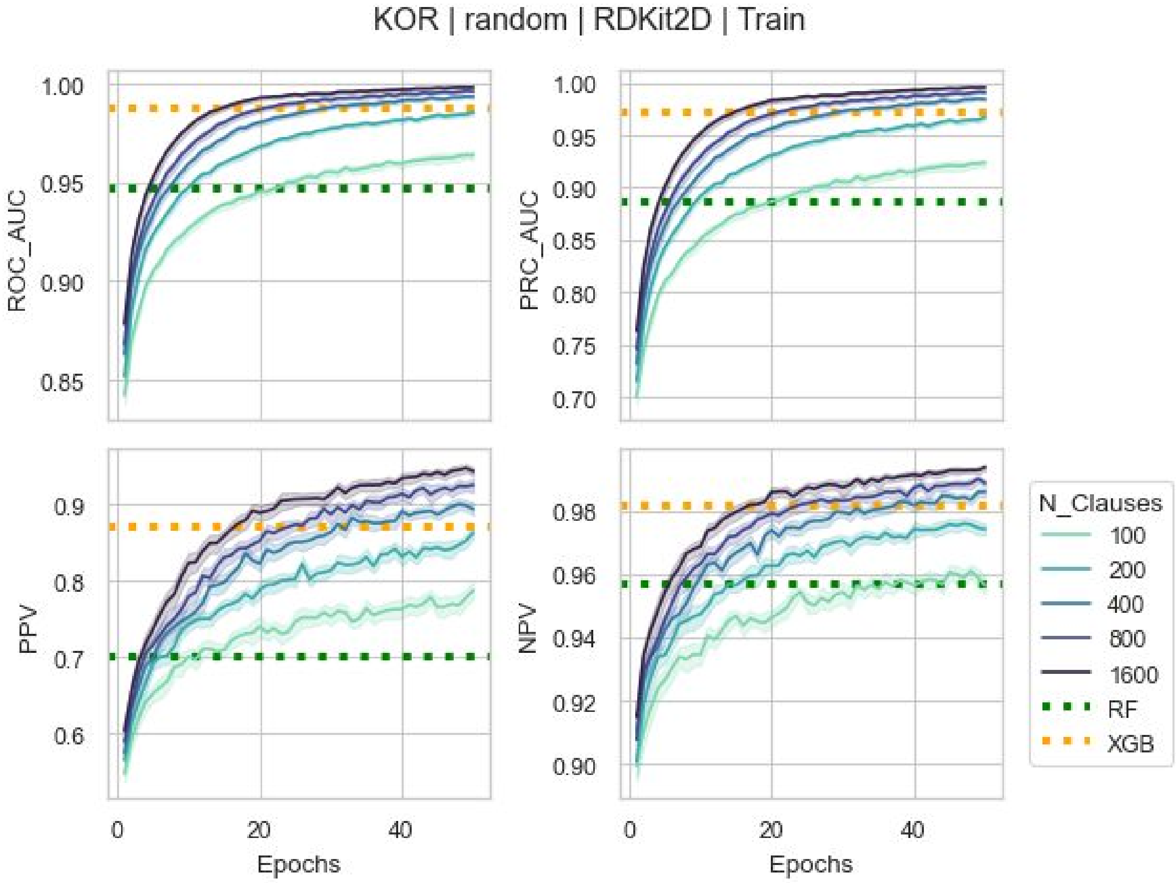

Figure S64: TM metric scores over 50 epochs on KOR training sets for random split-group and RDKit2D descriptors. Annotated by dotted lines are the mean training set scores of RF and XGBoost.

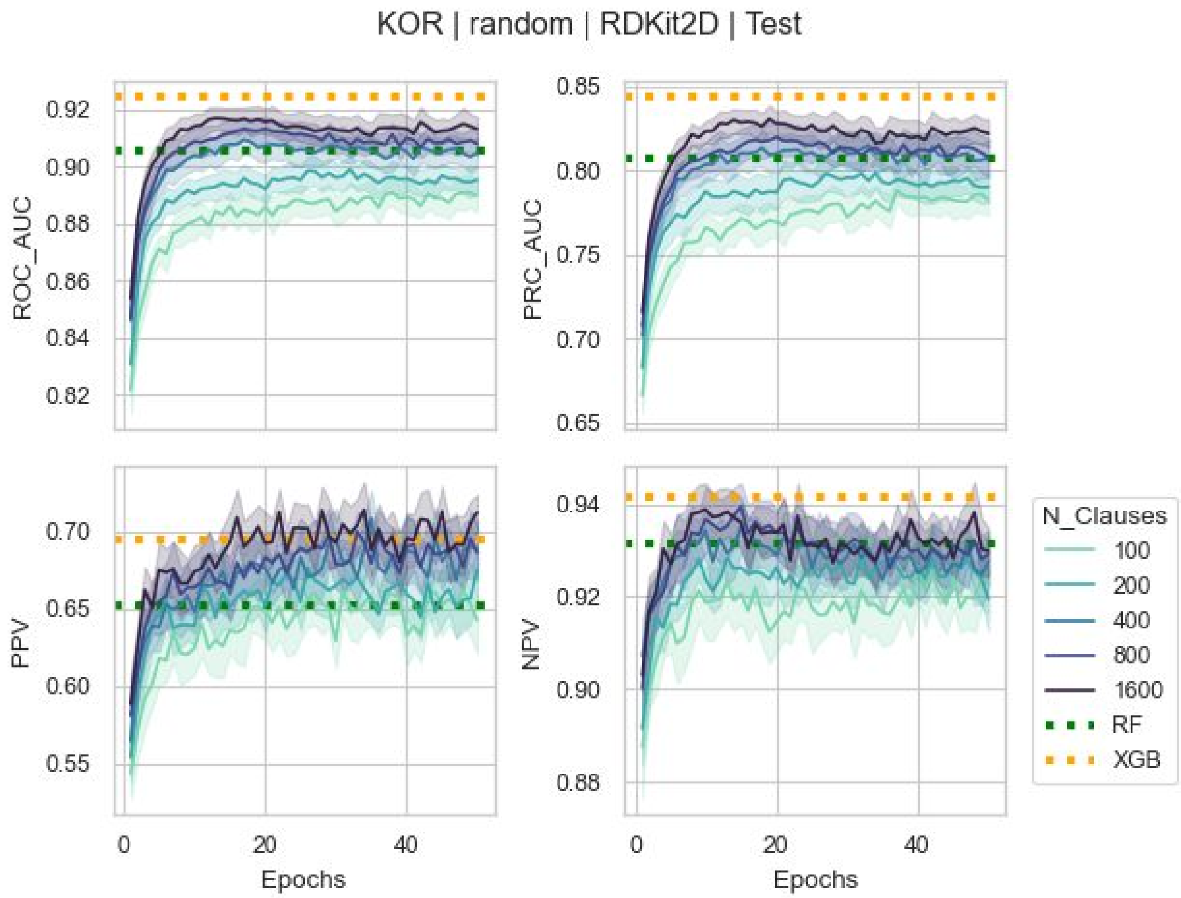

Figure S65: TM metric scores over 50 epochs on KOR test sets for random split-group and RDKit2D descriptors. Annotated by dotted lines are the mean training set scores of RF and XGBoost.

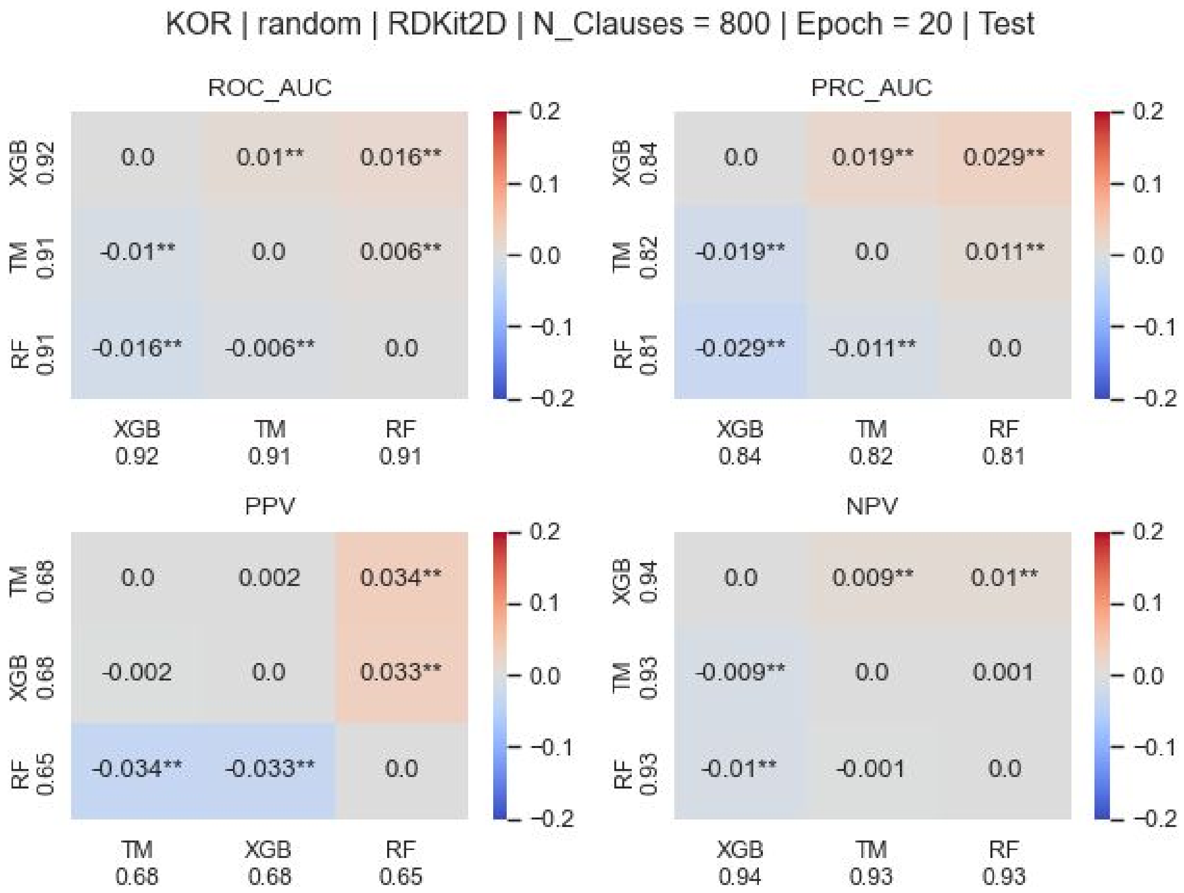

Figure S66: Cohen's D difference of means, pair-wise comparison of models for the KOR dataset with random group-split, RDKit2D descriptors and TM models of 800 clauses at 20 epochs. Complete with annotated statistical tests via Tukey's HSD where the number of asterix represents a different statistical significance level.

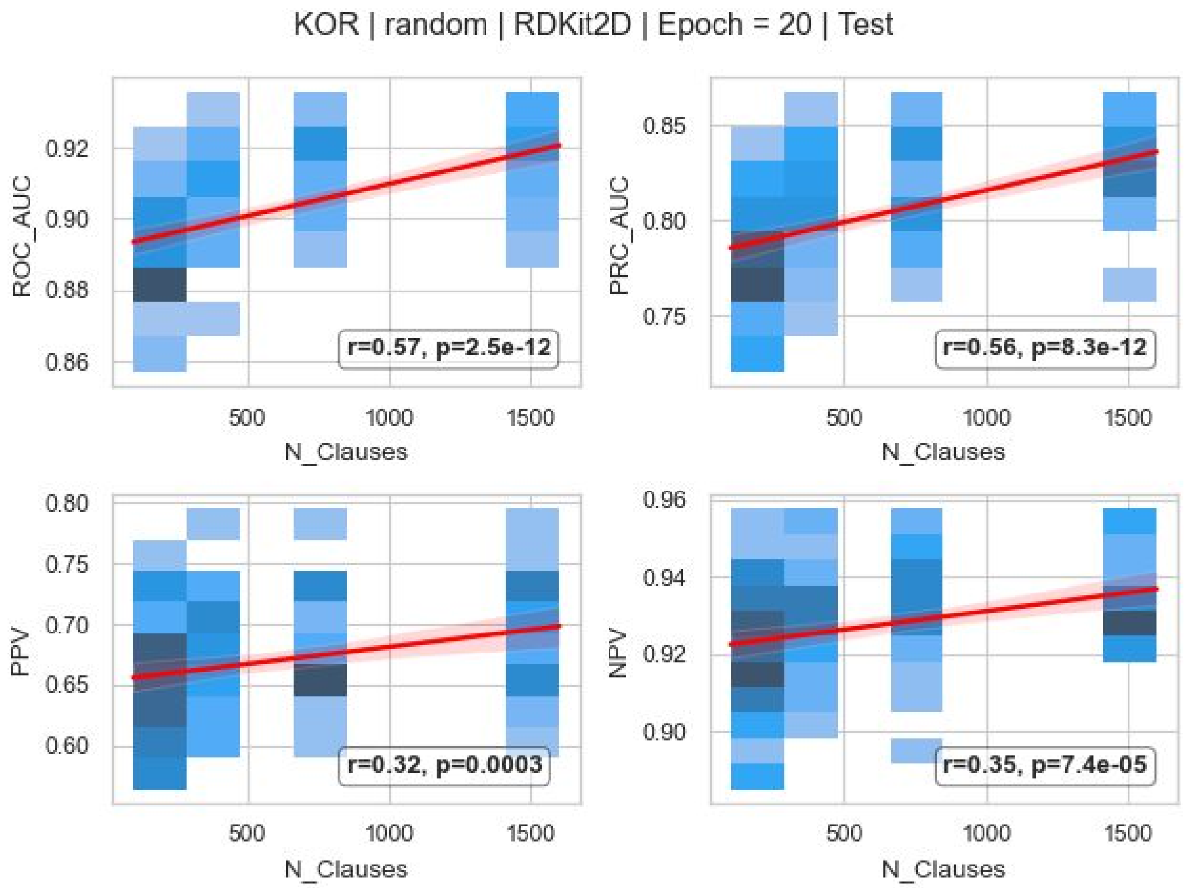

Figure S67: Number of clauses and metric-score histograms of KOR test sets with fitted line for random split-group, RDKit2D descriptors and TM-models of 800 clauses at 20 epochs. Pearson's R and p-value are annotated for said line.

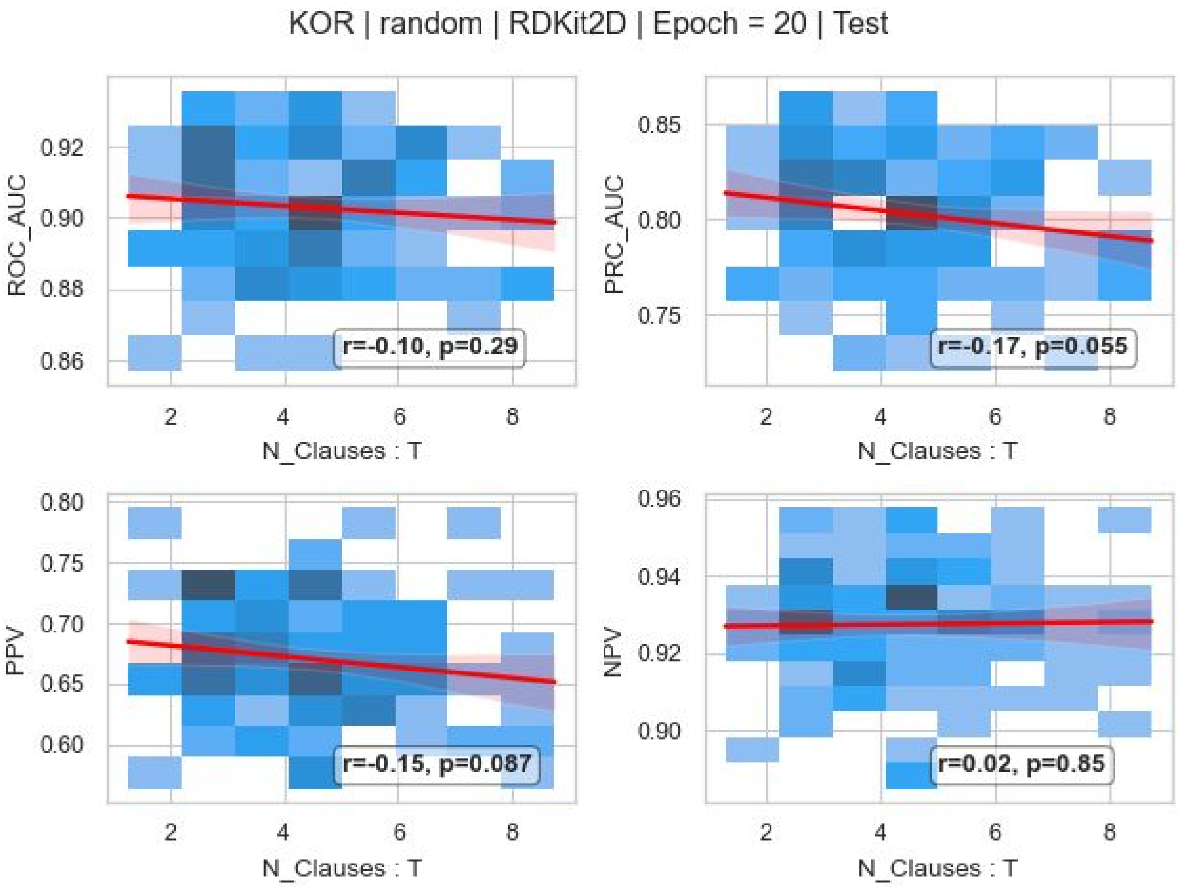

Figure S68: *N_Clauses : T* ratio and test-set metric scores histogram of KOR test sets with fitted line for random split-group, RDKit2D descriptors and TM-models of 800 clauses at 20 epochs. Pearson's R and p-value are annotated for said line.

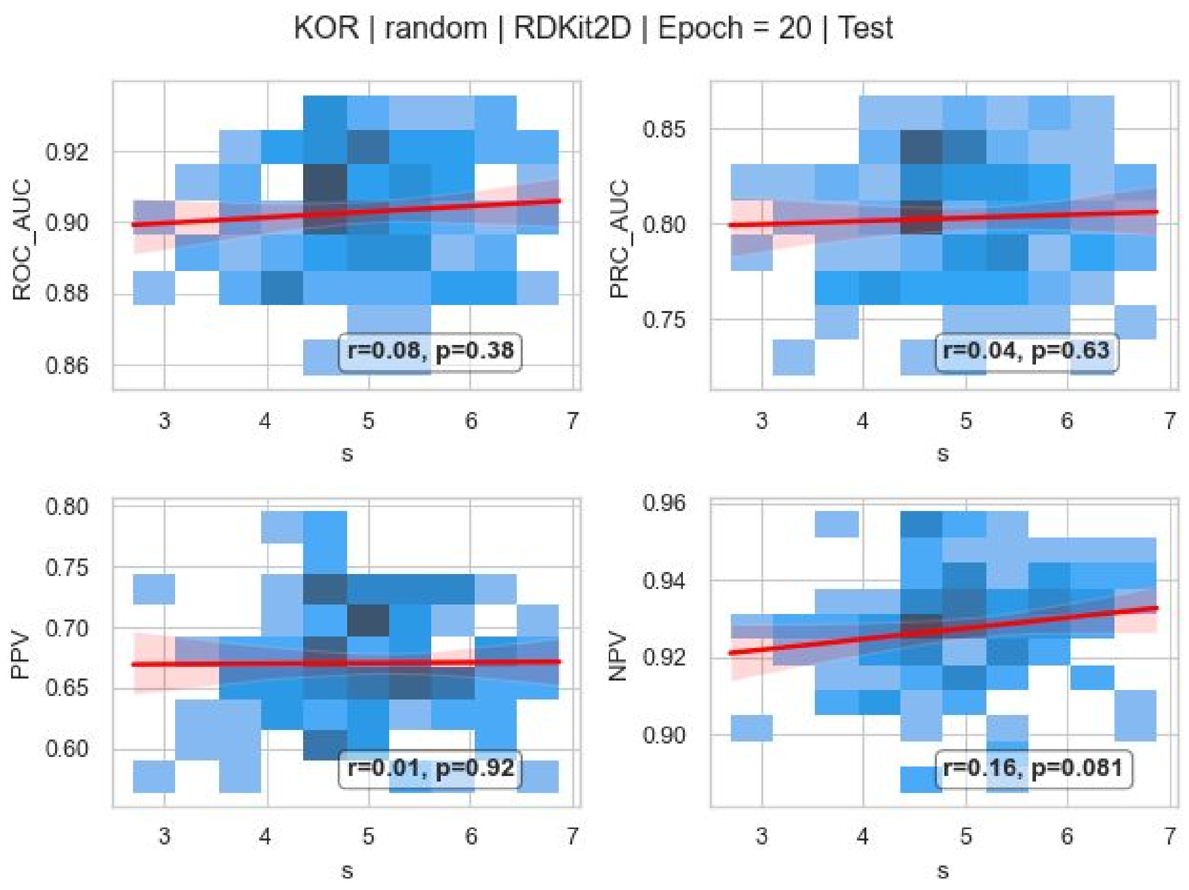

Figure S69: Hyper-parameter *s* and test-set metric scores histogram of KOR test sets with fitted line for random split-group, RDKit2D descriptors and TM-models of 800 clauses at 20 epochs. Pearson's R and p-value are annotated for said line.

scaffold group-split | RDKit2D Descriptors

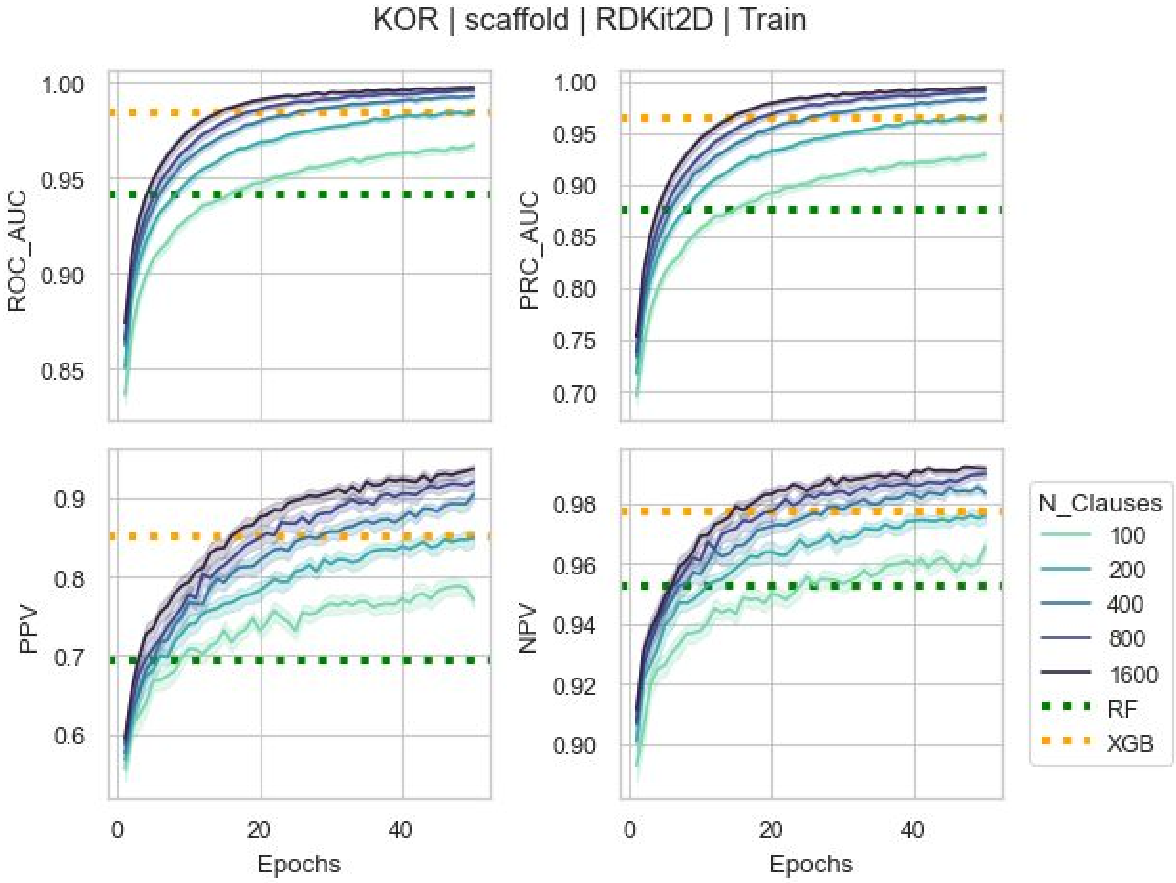

Figure S70: TM metric scores over 50 epochs on KOR training sets for scaffold split-group and RDKit2D descriptors. Annotated by dotted lines are the mean training set scores of RF and XGBoost.

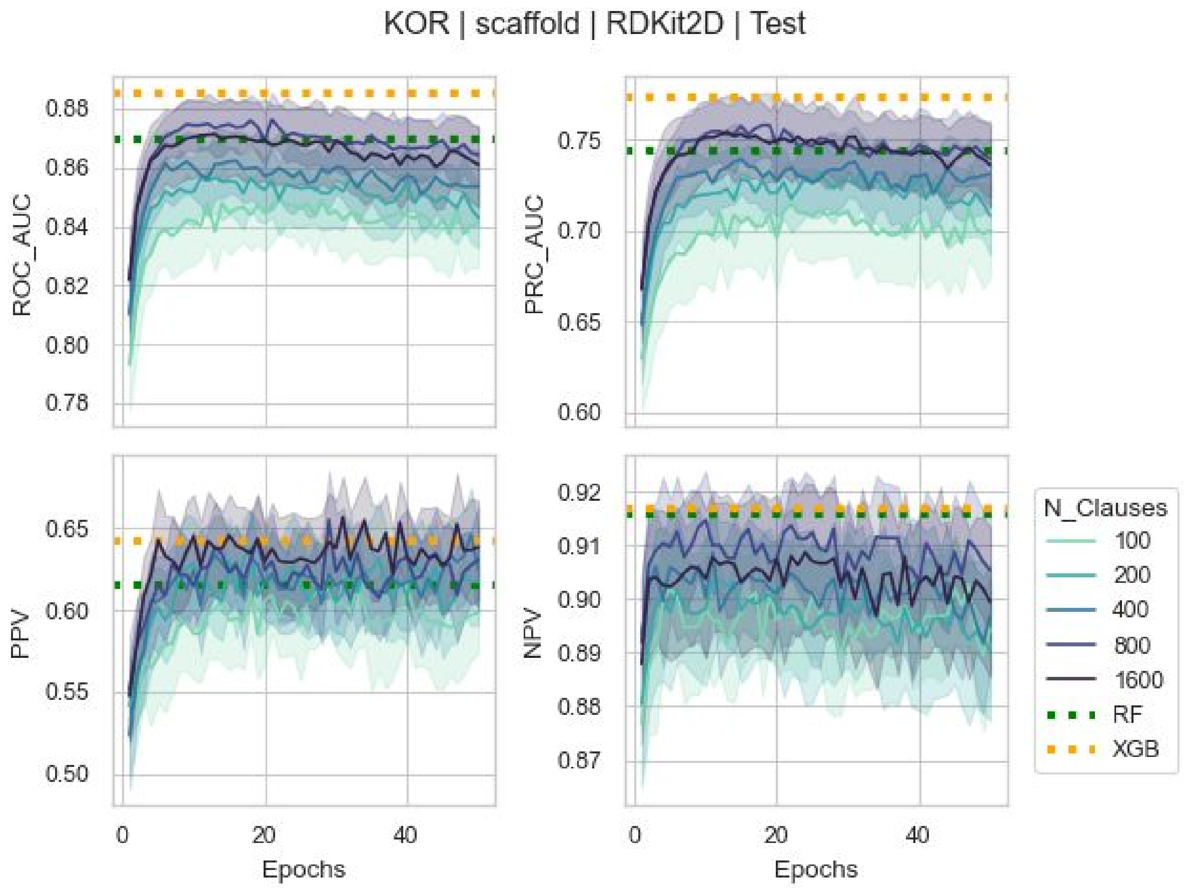

Figure S71: TM metric scores over 50 epochs on KOR test sets for scaffold split-group and RDKit2D descriptors. Annotated by dotted lines are the mean training set scores of RF and XGBoost.

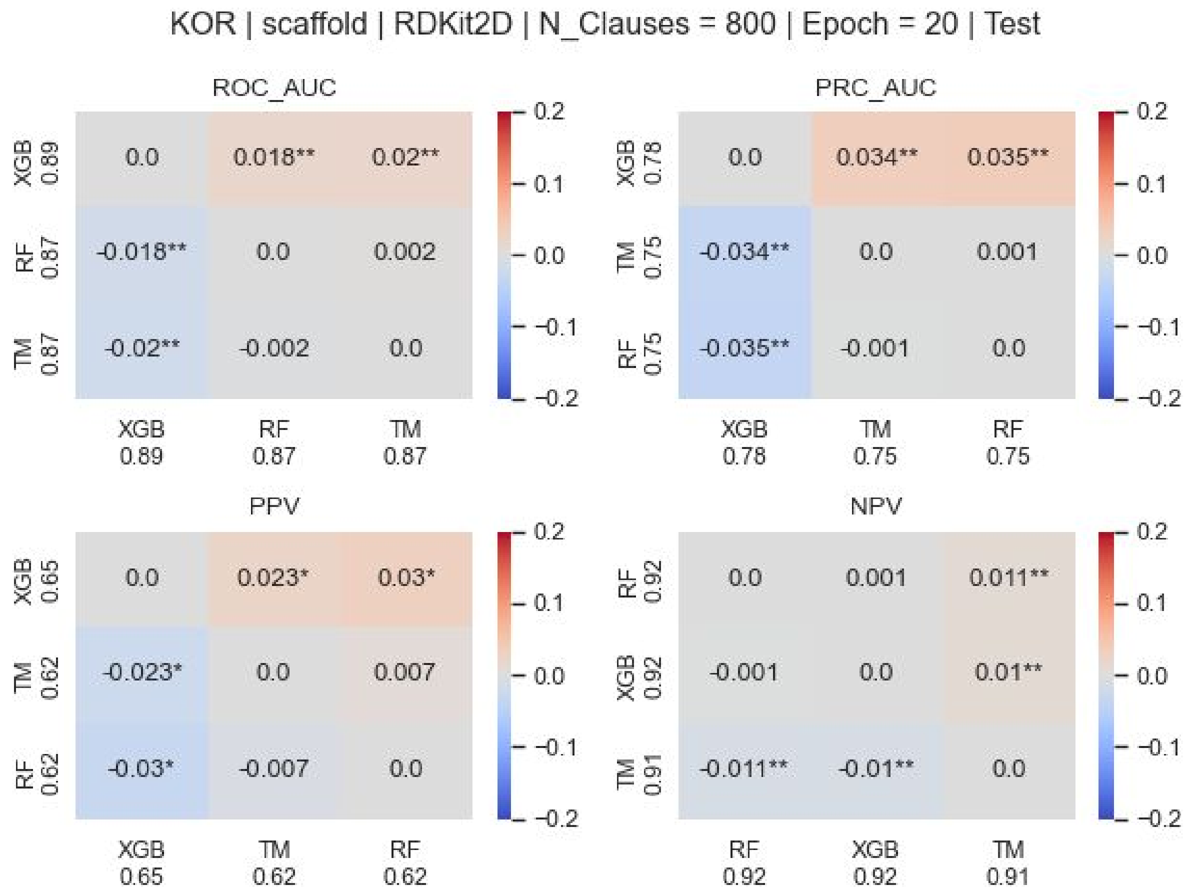

Figure S72: Cohen's D difference of means, pair-wise comparison of models for the KOR dataset with scaffold group-split, RDKit2D descriptors and TM models of 800 clauses at 20 epochs. Complete with annotated statistical tests via Tukey's HSD where the number of asterix represents a different statistical significance level.

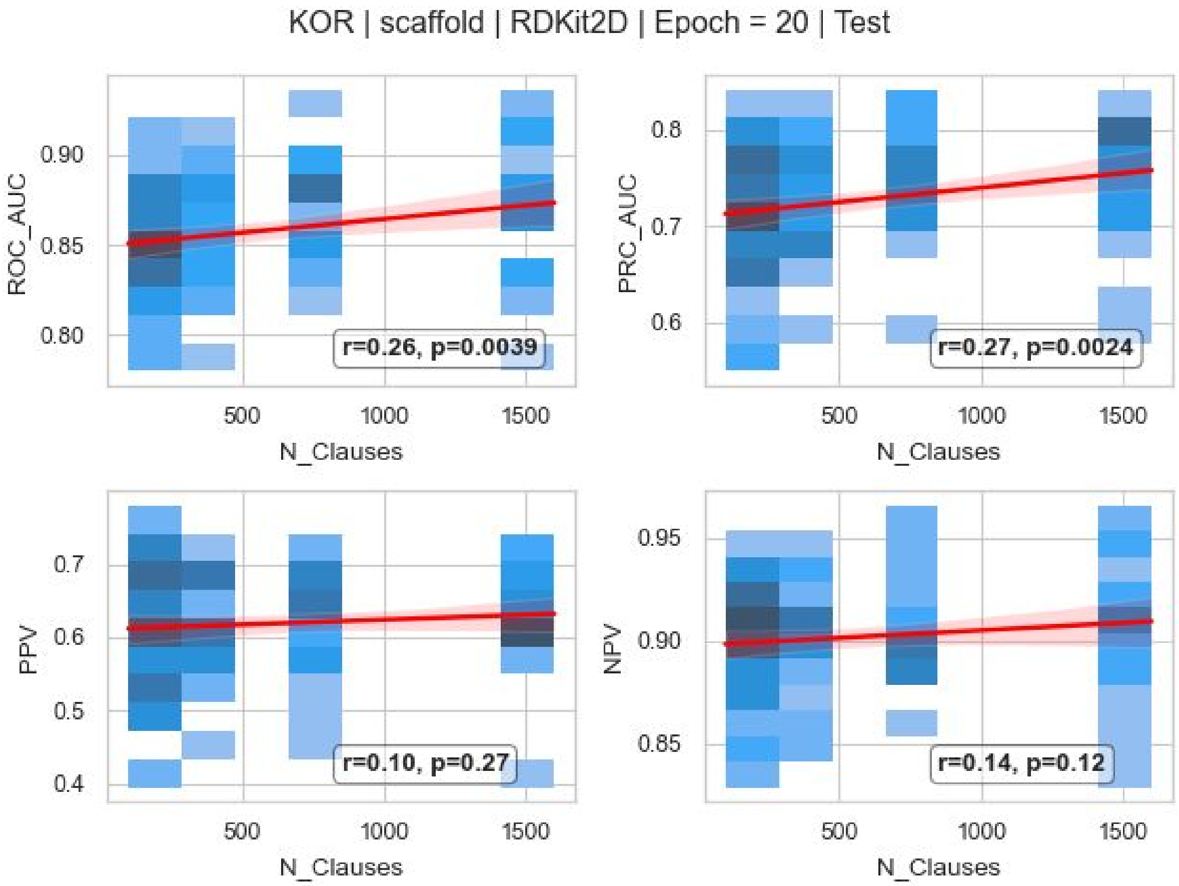

Figure S73: Number of clauses and metric-score histograms of KOR test sets with fitted line for scaffold split-group, RDKit2D descriptors and TM-models of 800 clauses at 20 epochs. Pearson's R and p-value are annotated for said line.

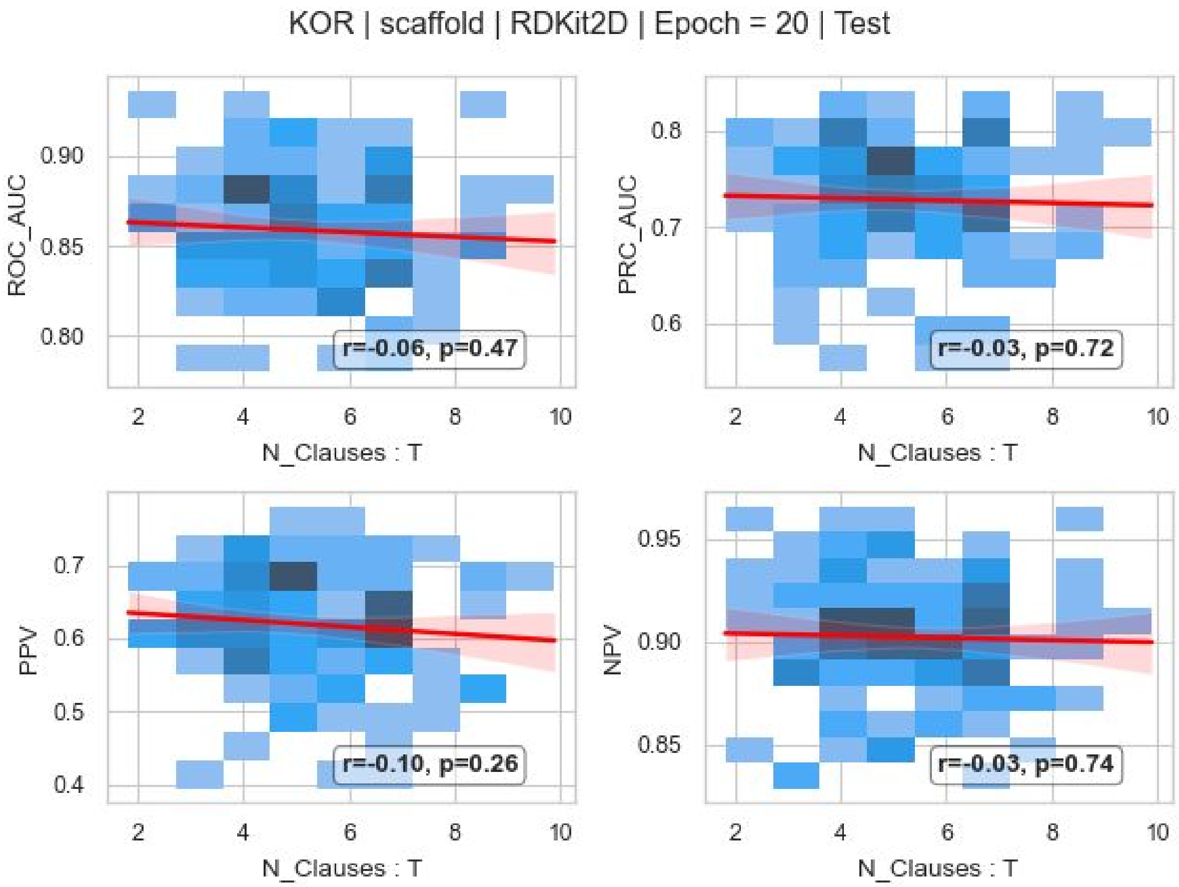

Figure S74: *N_Clauses : T* ratio and test-set metric scores histogram of KOR test sets with fitted line for scaffold split-group, RDKit2D descriptors and TM-models of 800 clauses at 20 epochs. Pearson's R and p-value are annotated for said line.

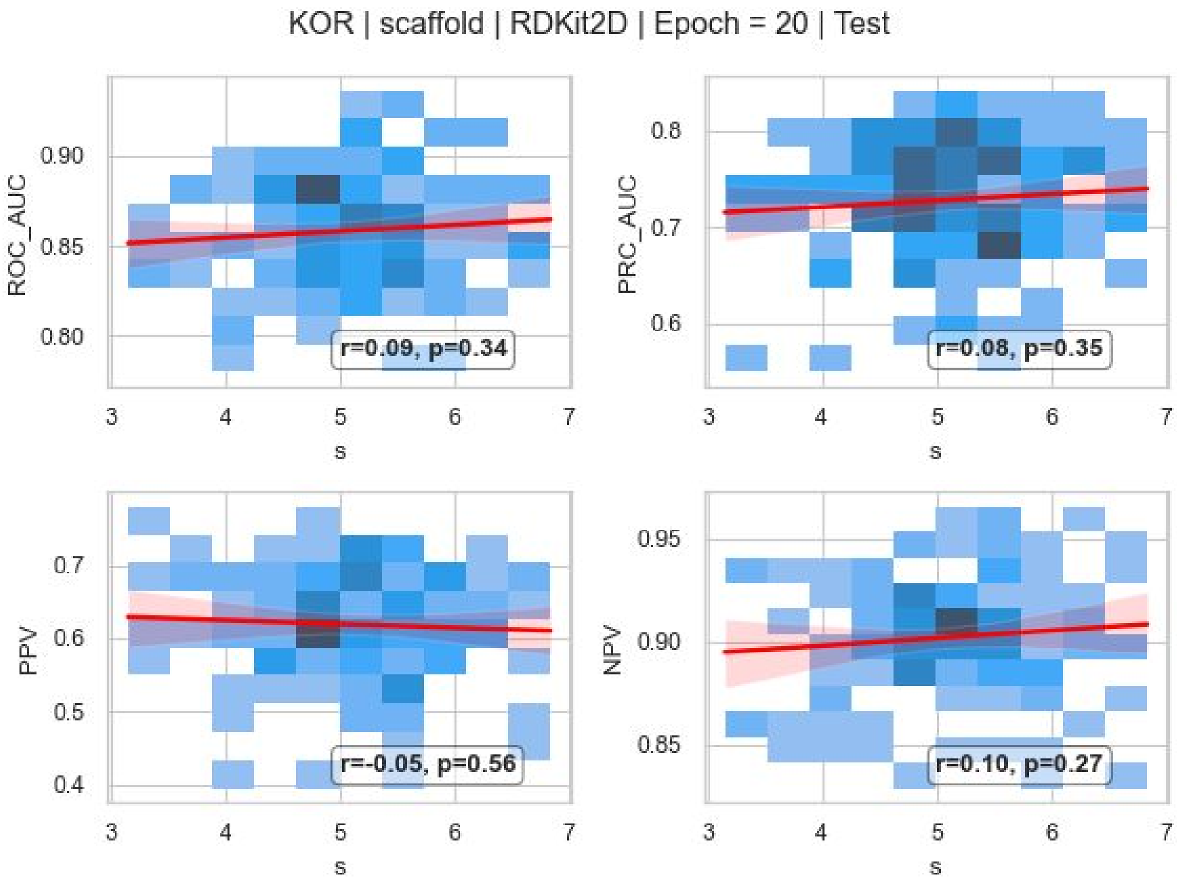

Figure S75: Hyper-parameter *s* and test-set metric scores histogram of KOR test sets with fitted line for scaffold split-group, RDKit2D descriptors and TM-models of 800 clauses at 20 epochs. Pearson's R and p-value are annotated for said line.

CYP3A4

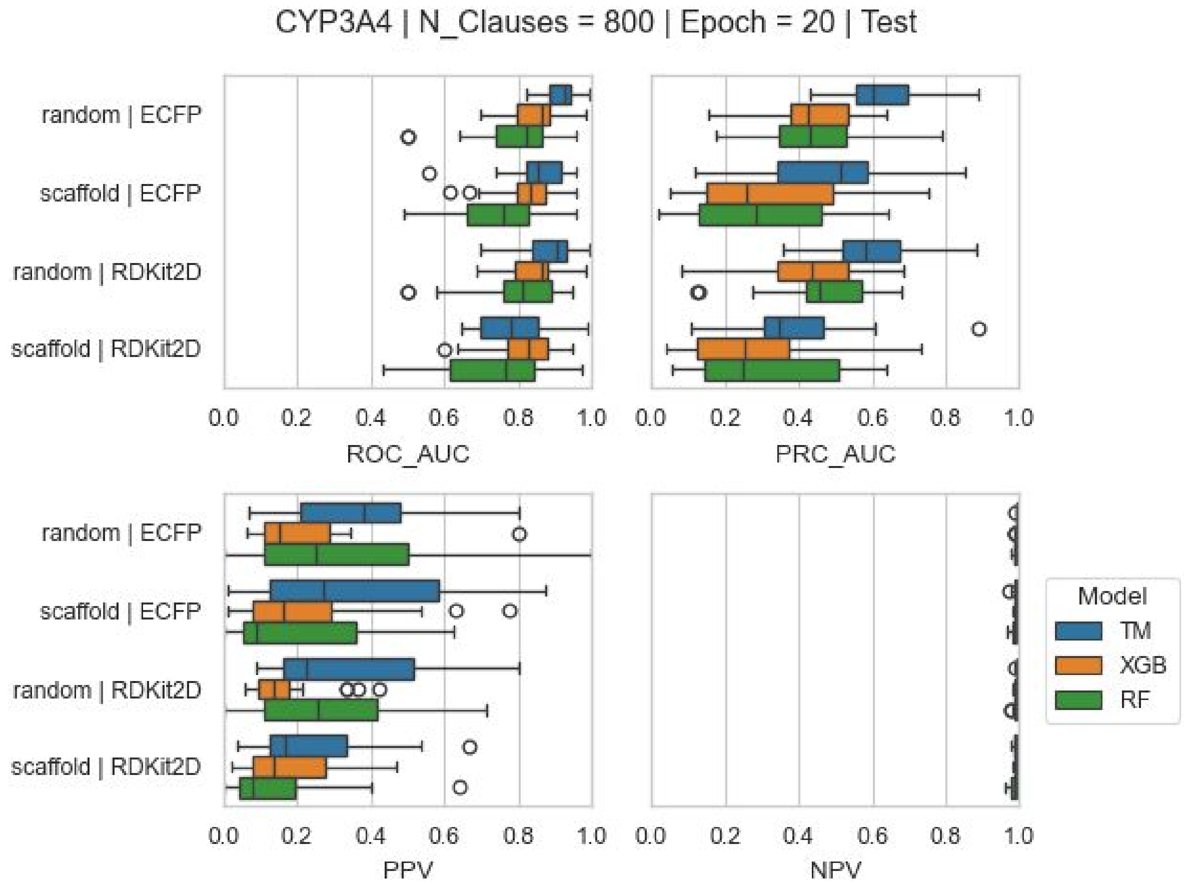

Figure S76: Box plot of model metric scores on a test set across split-group | descriptor pairs for CYP3A4 dataset. The TM uses 800 clauses and learning is stopped after 20 epochs.

random split-group I ECFP Descriptors

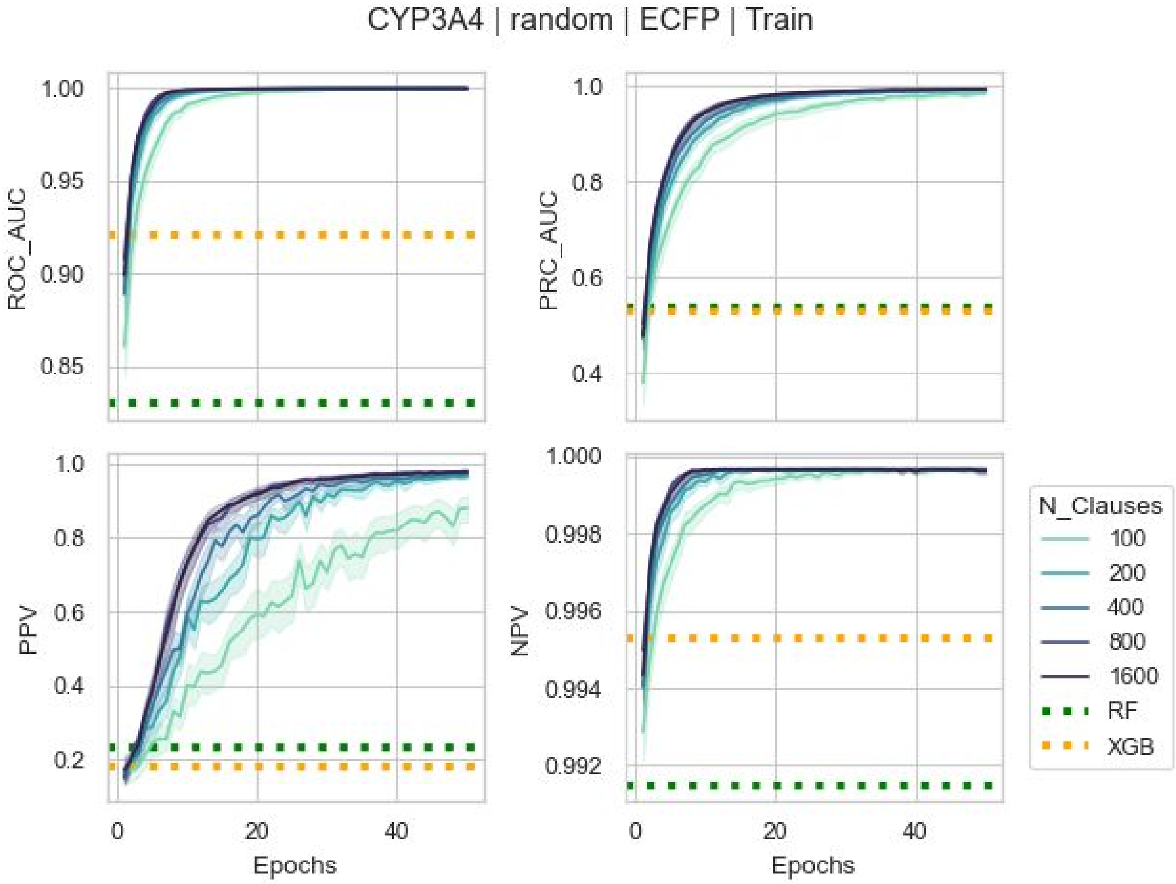

Figure S77: TM metric scores over 50 epochs on CYP3A4 training sets for random split-group and ECFP descriptors. Annotated by dotted lines are the mean training set scores of RF and XGBoost.

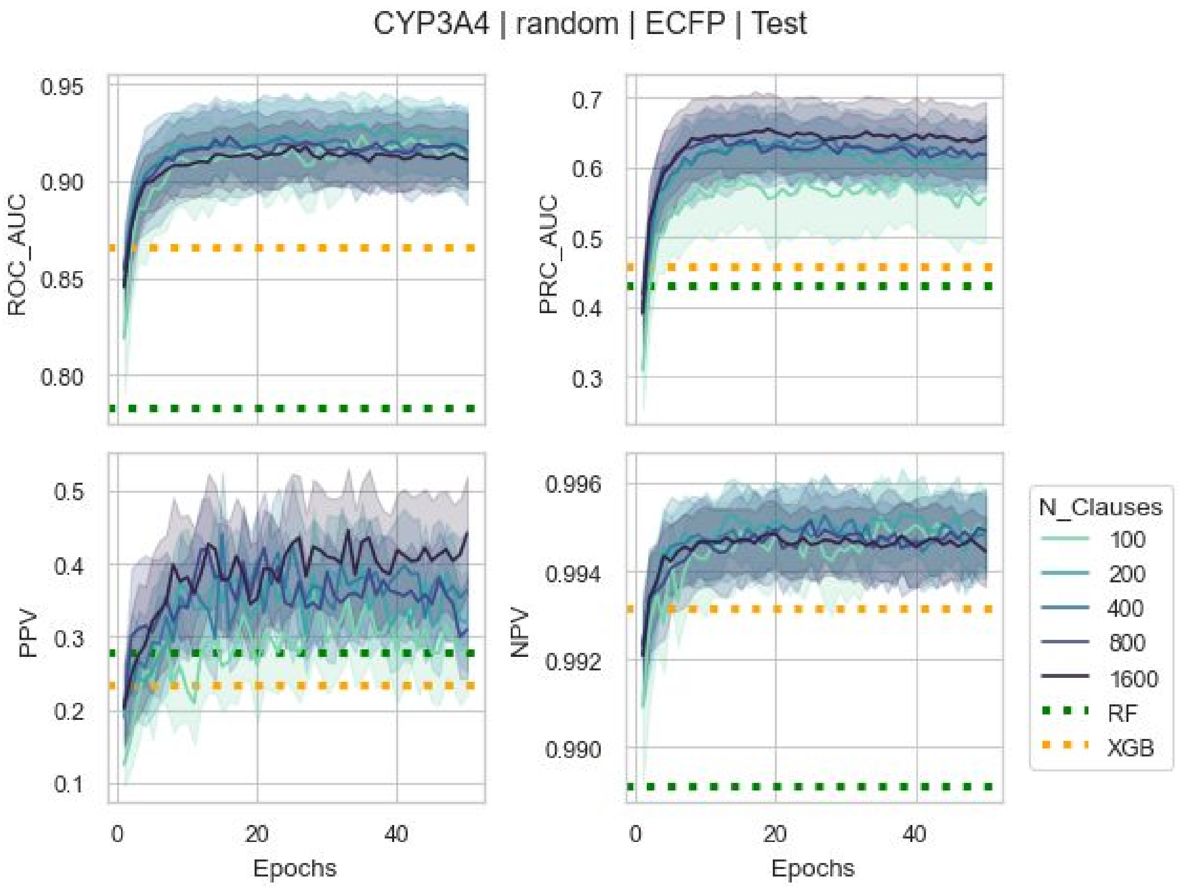

Figure S78: TM metric scores over 50 epochs on CYP3A4 test sets for random split-group and ECFP descriptors. Annotated by dotted lines are the mean test set scores of RF and XGBoost.

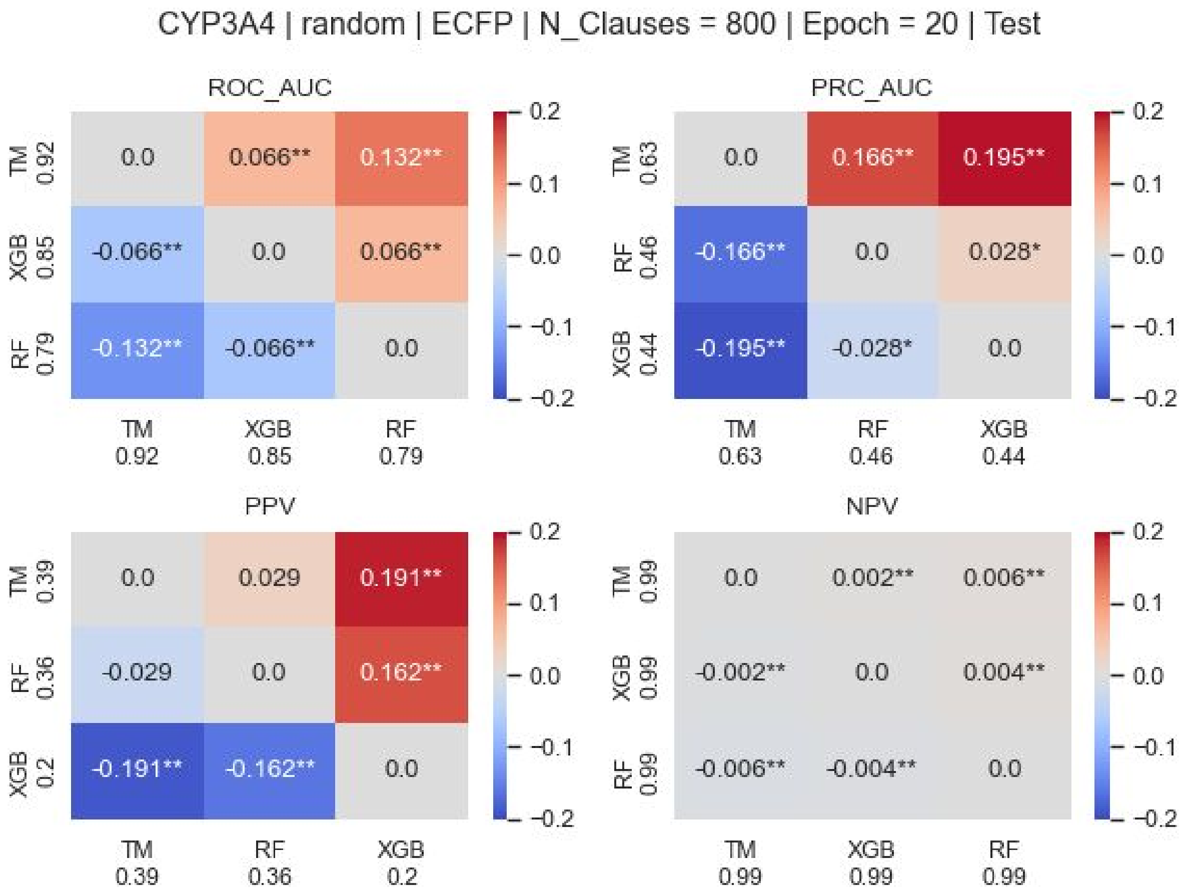

Figure S79: Cohen's D difference of means, pair-wise comparison of models for the CYP3A4 dataset with random group-split, ECFP descriptors and TM models of 800 clauses at 20 epochs. Complete with annotated statistical tests via Tukey's HSD where the number of asterix represents a different statistical significance level.

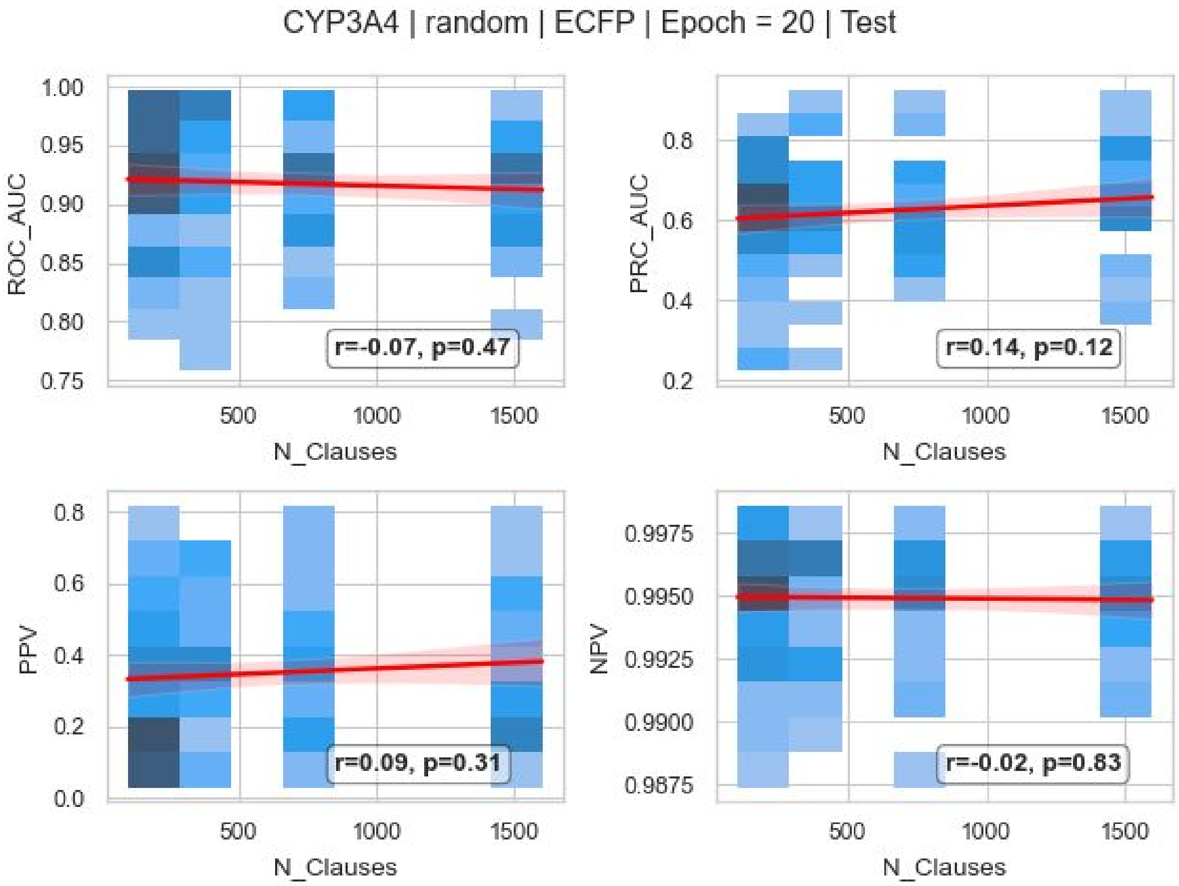

Figure S80: Number of clauses and metric-score histograms of CYP3A4 test sets with fitted line for random split-group, ECFP descriptors and TM-models of 800 clauses at 20 epochs. Pearson's R and p-value are annotated for said line.

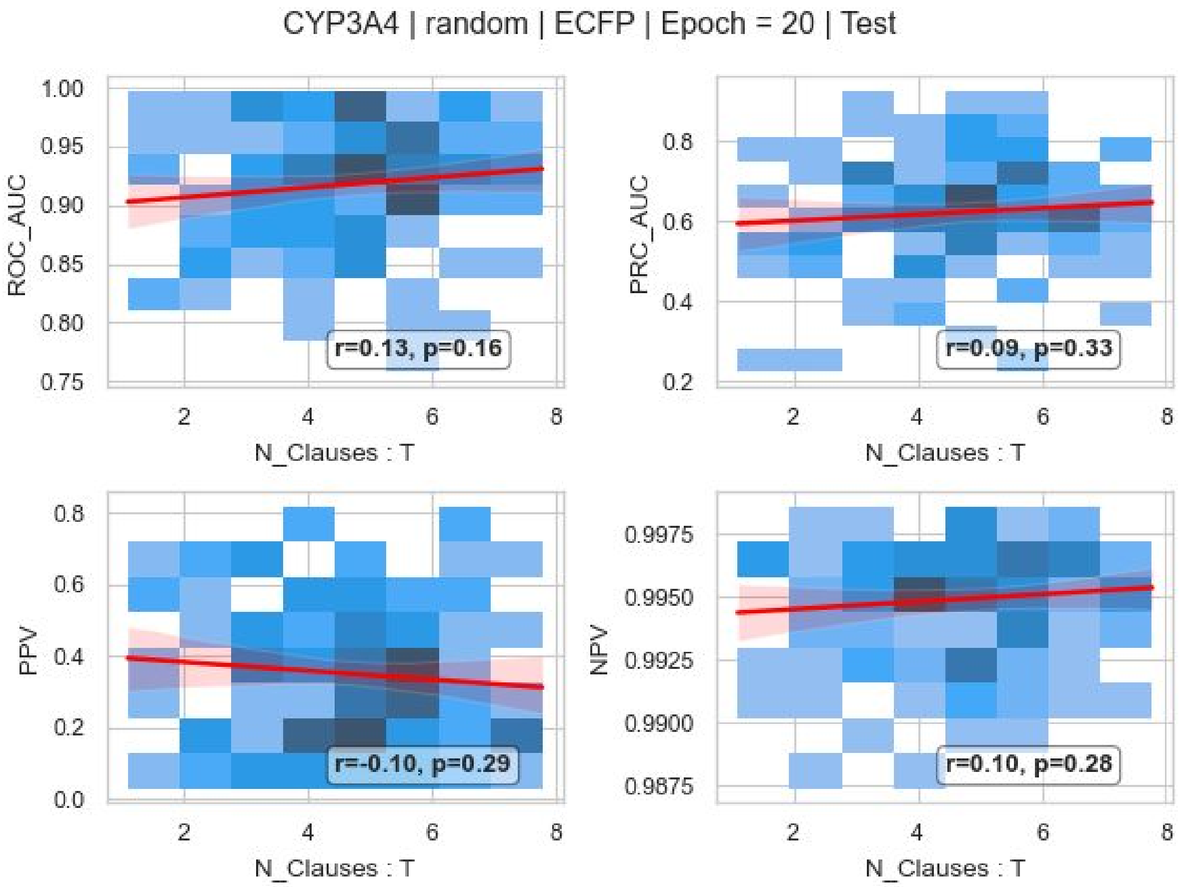

Figure S81: *N_Clauses : T* ratio and test-set metric scores histogram for CYP3A4 dataset with fitted line for random split-group, ECFP descriptors and TM-models of 800 clauses at 20 epochs. Pearson's R and p-value are annotated for said line.

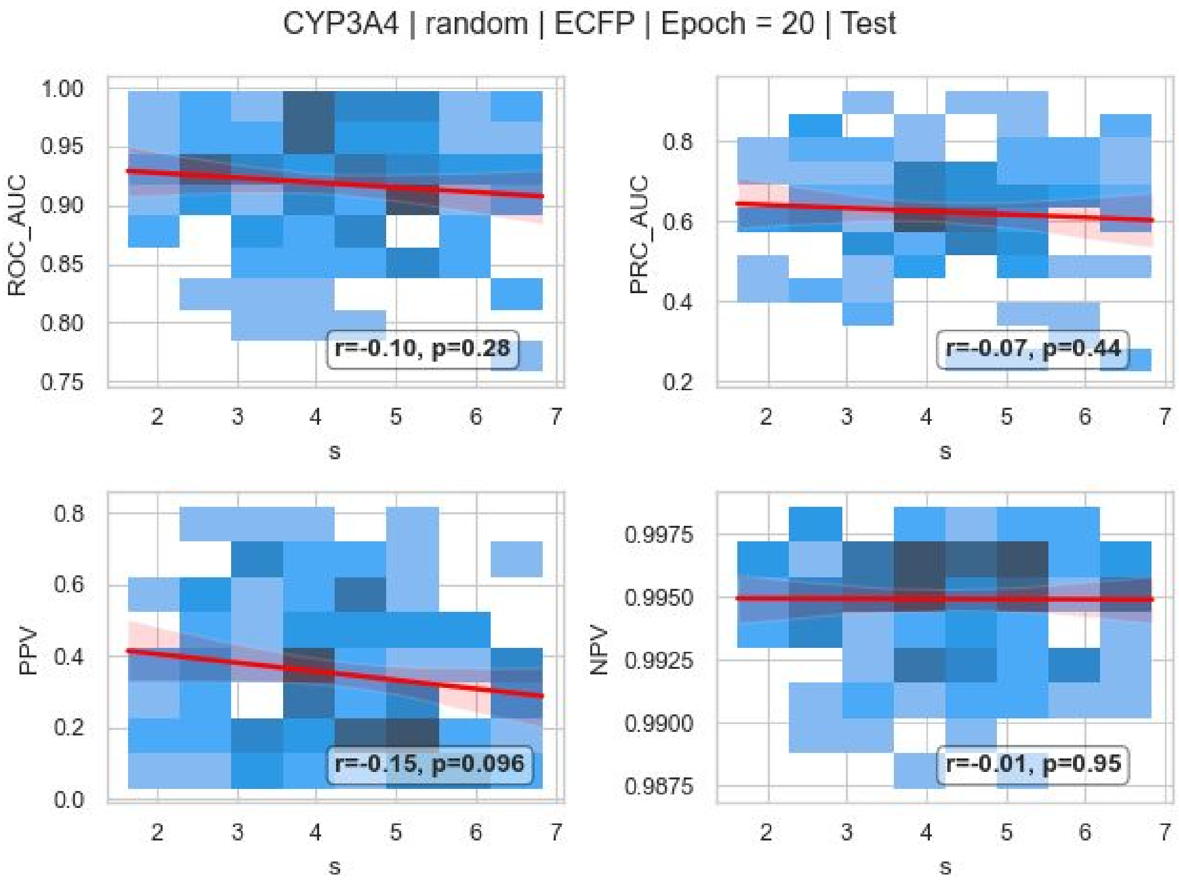

Figure S82: Hyper-parameter *s* and test-set metric scores histogram for CYP3A4 dataset with fitted line for random split-group, ECFP descriptors and TM-models of 800 clauses at 20 epochs. Pearson's R and p-value are annotated for said line.

scaffold split-group | ECFP Descriptors

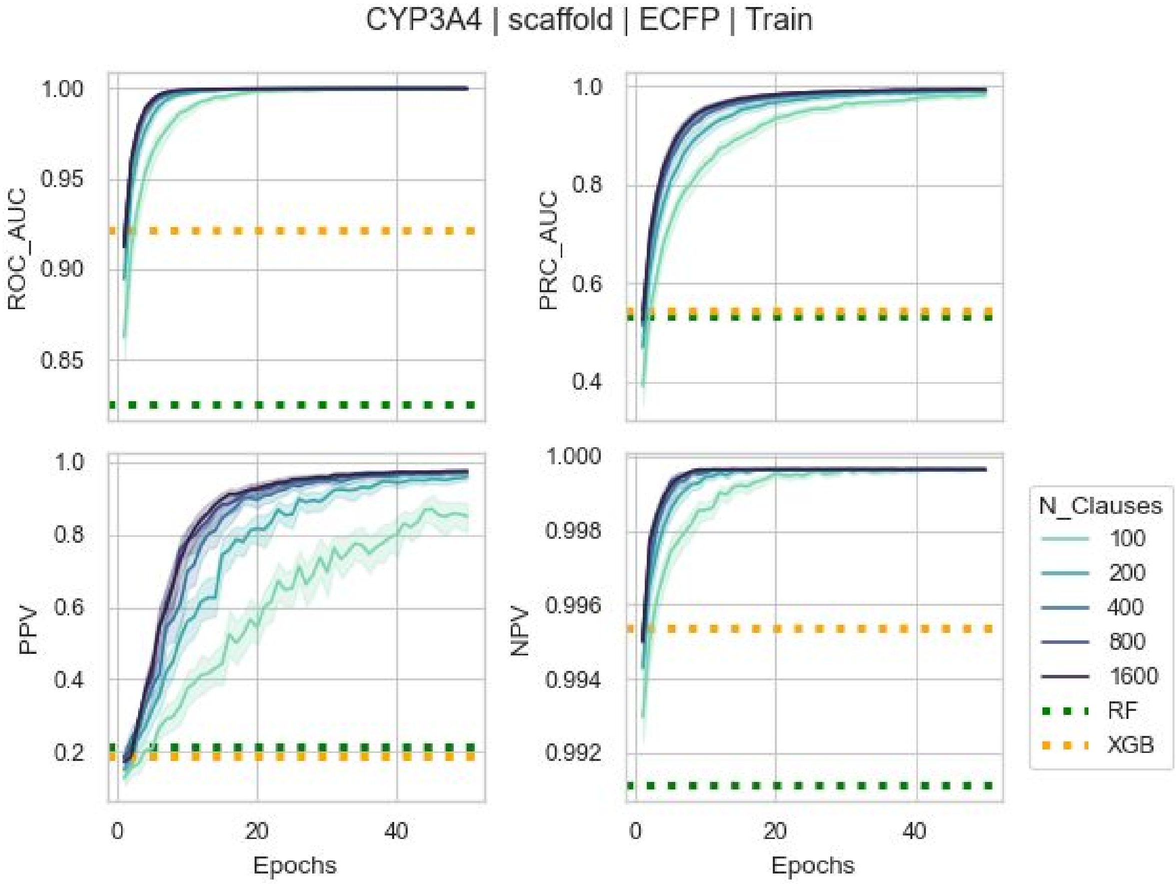

Figure S83: TM metric scores over 50 epochs on CYP3A4 training sets for scaffold split-group and ECFP descriptors. Annotated by dotted lines are the mean training set scores of RF and XGBoost.

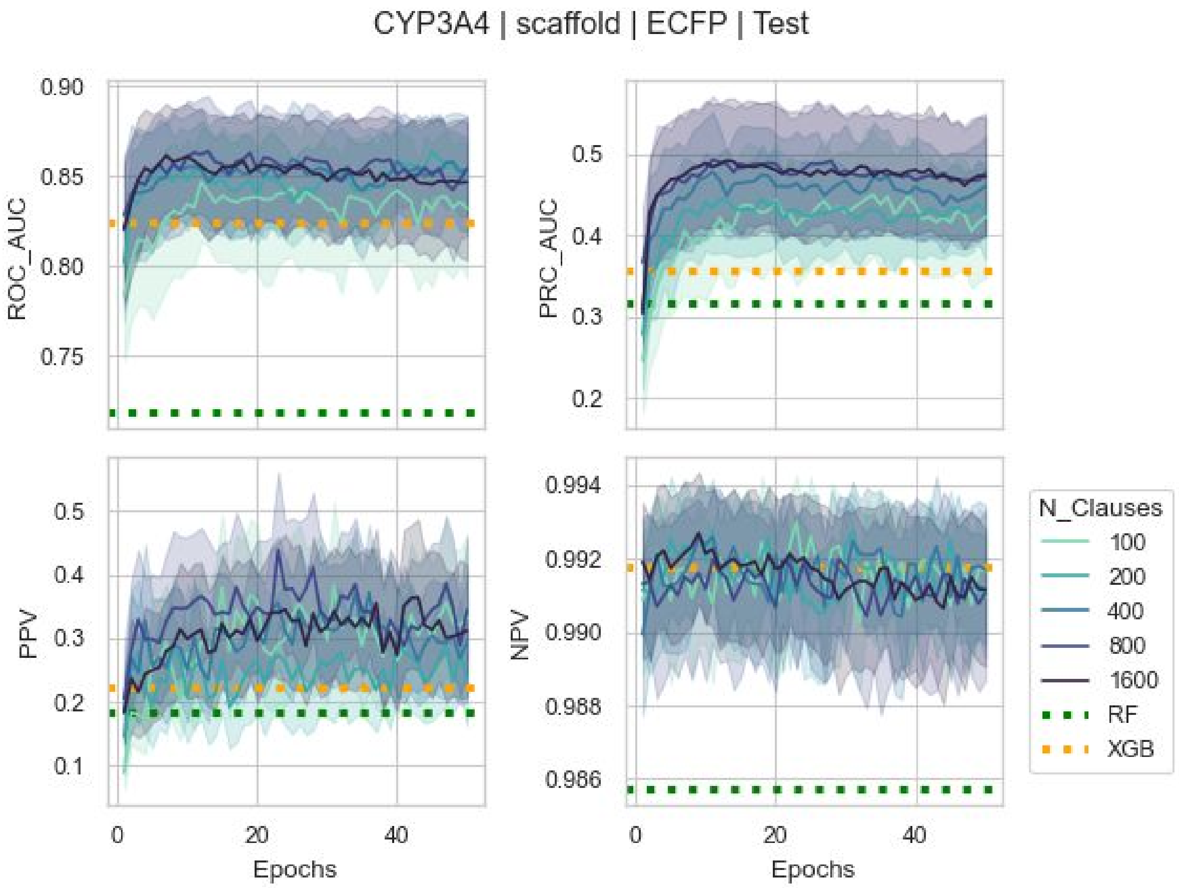

Figure S84: TM metric scores over 50 epochs on CYP3A4 test sets for scaffold split-group and ECFP descriptors. Annotated by dotted lines are the mean training set scores of RF and XGBoost.

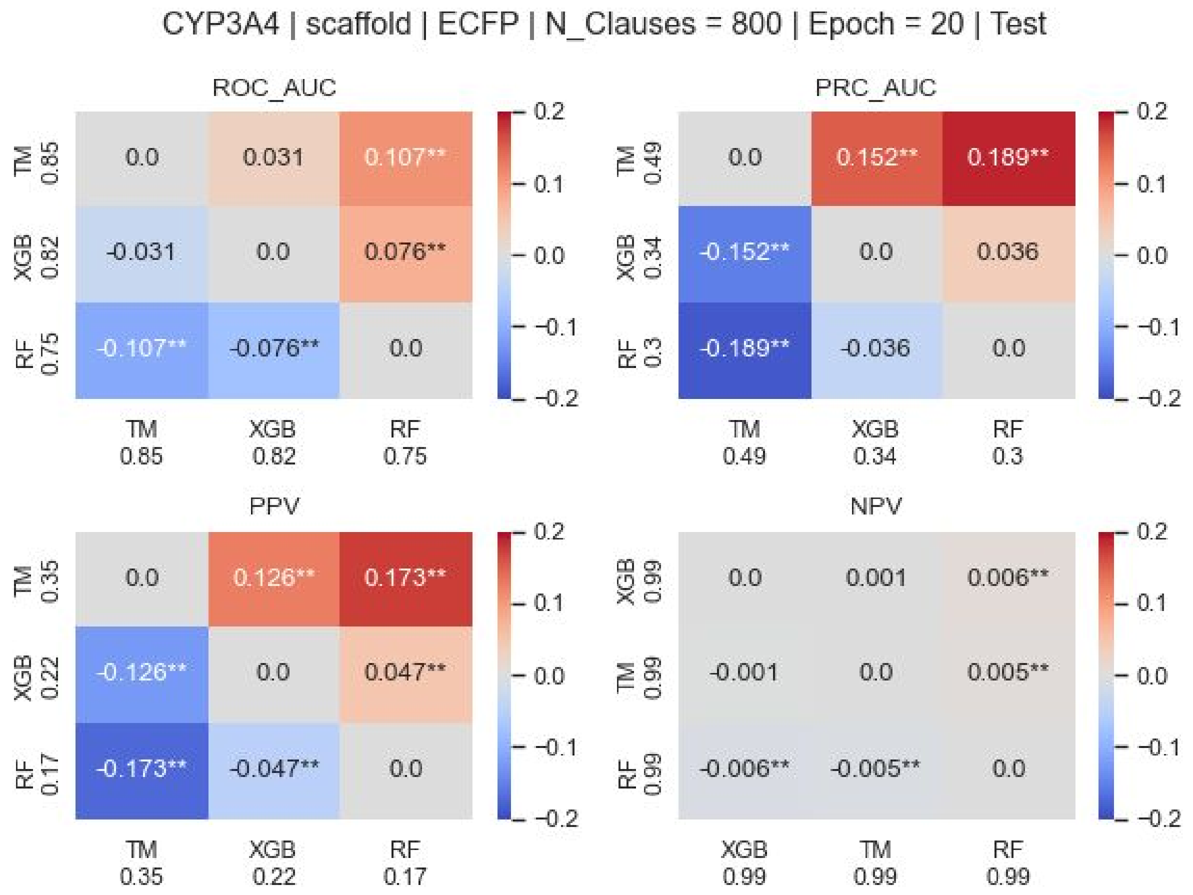

Figure S85: Cohen's D difference of means, pair-wise comparison of models for the CYP3A4 dataset with scaffold group-split, ECFP descriptors and TM models of 800 clauses at 20 epochs. Complete with annotated statistical tests via Tukey's HSD where the number of asterix represents a different statistical significance level.

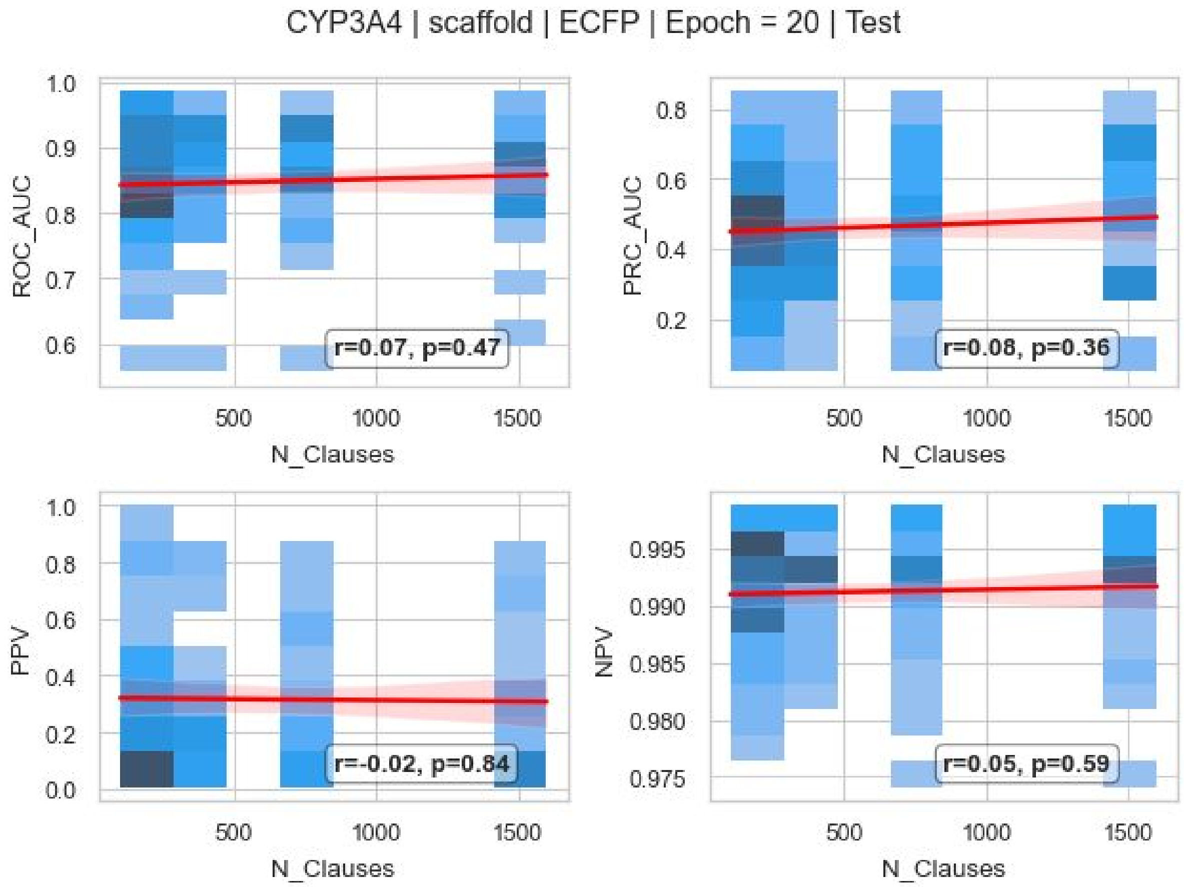

Figure S86: Number of clauses and metric-score histograms of KOR test sets with fitted line for scaffold split-group, ECFP descriptors and TM-models of 800 clauses at 20 epochs. Pearson’s R and p-value are annotated for said line.

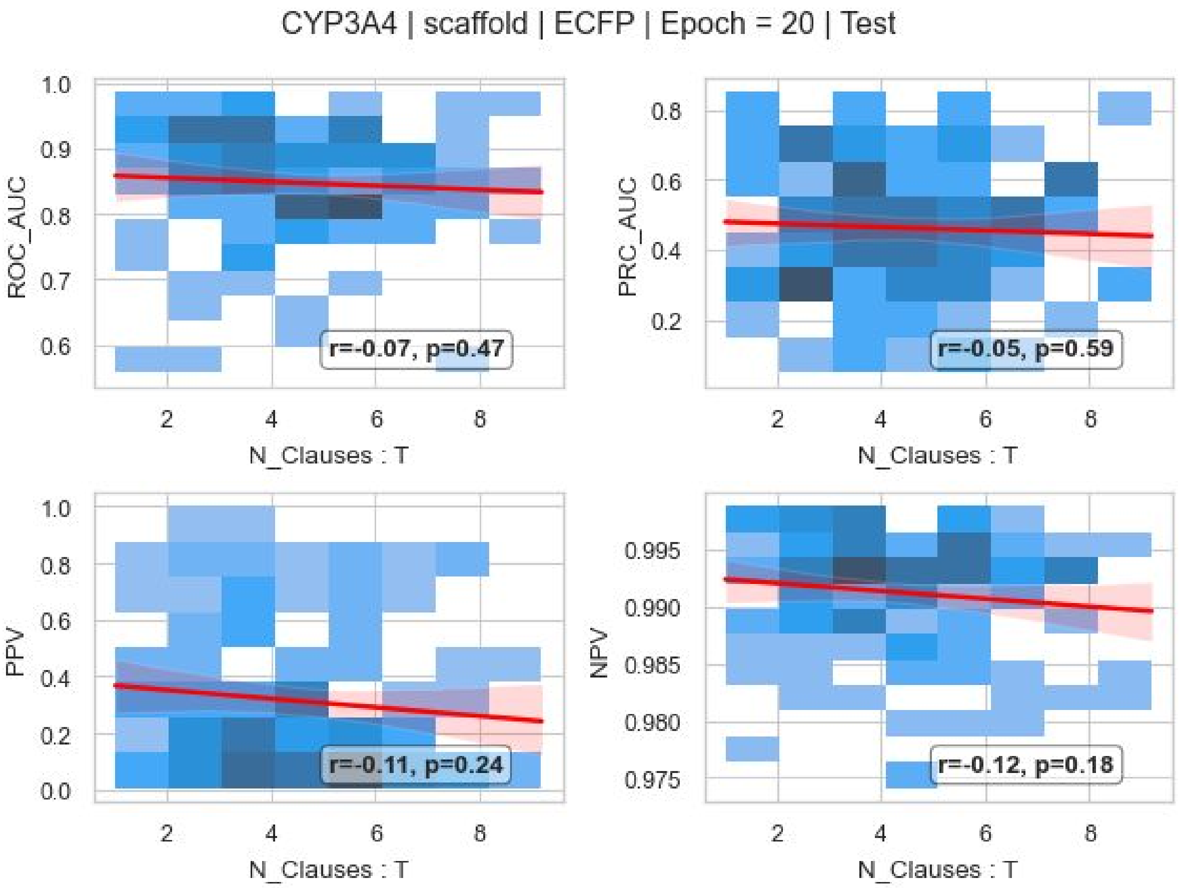

Figure S87: *N_Clauses : T* ratio and test-set metric scores histogram of CYP3A4 test sets with fitted line for scaffold split-group, ECFP descriptors and TM-models of 800 clauses at 20 epochs. Pearson's R and p-value are annotated for said line.

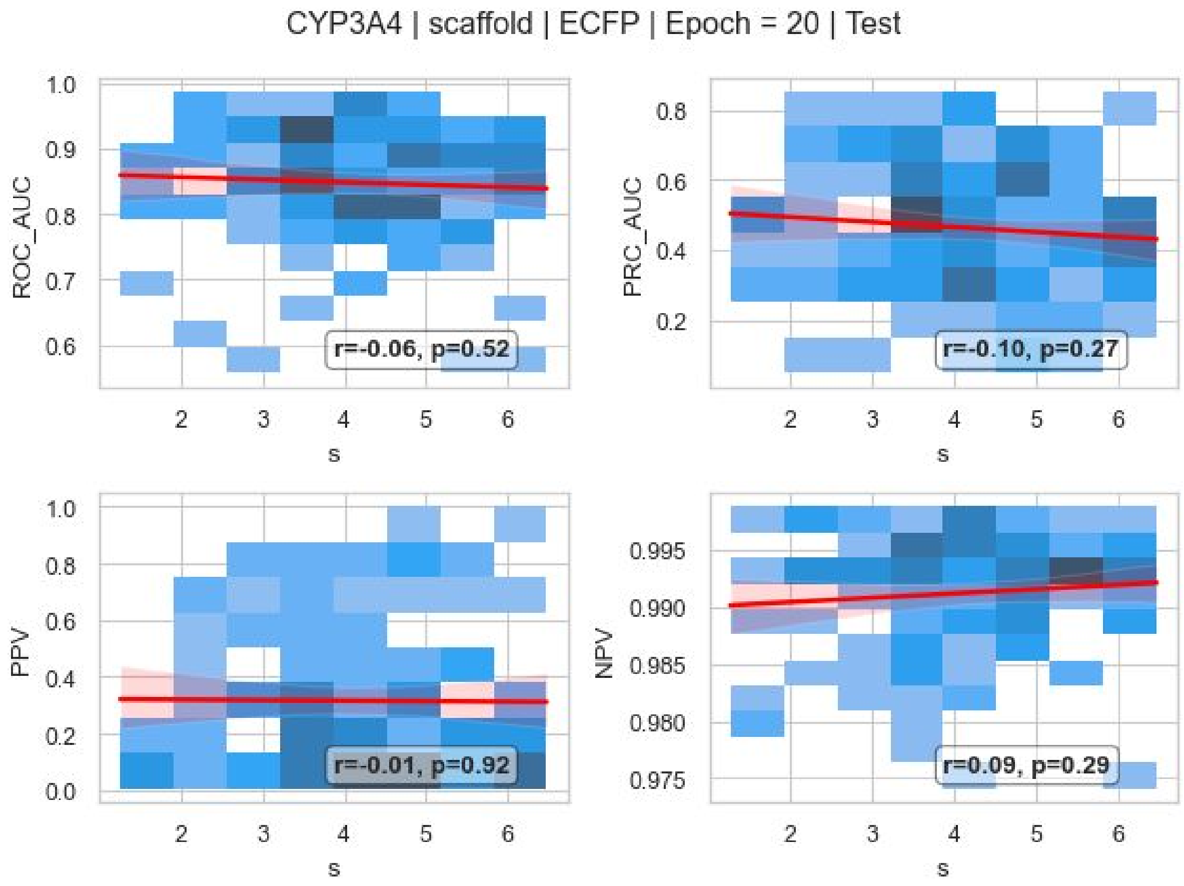

Figure S88: Hyper-parameter *s* and test-set metric scores histogram of CYP3A4 test sets with fitted line for scaffold split-group, ECFP descriptors and TM-models of 800 clauses at 20 epochs. Pearson's R and p-value are annotated for said line.

random split-group | RDKit2D Descriptors

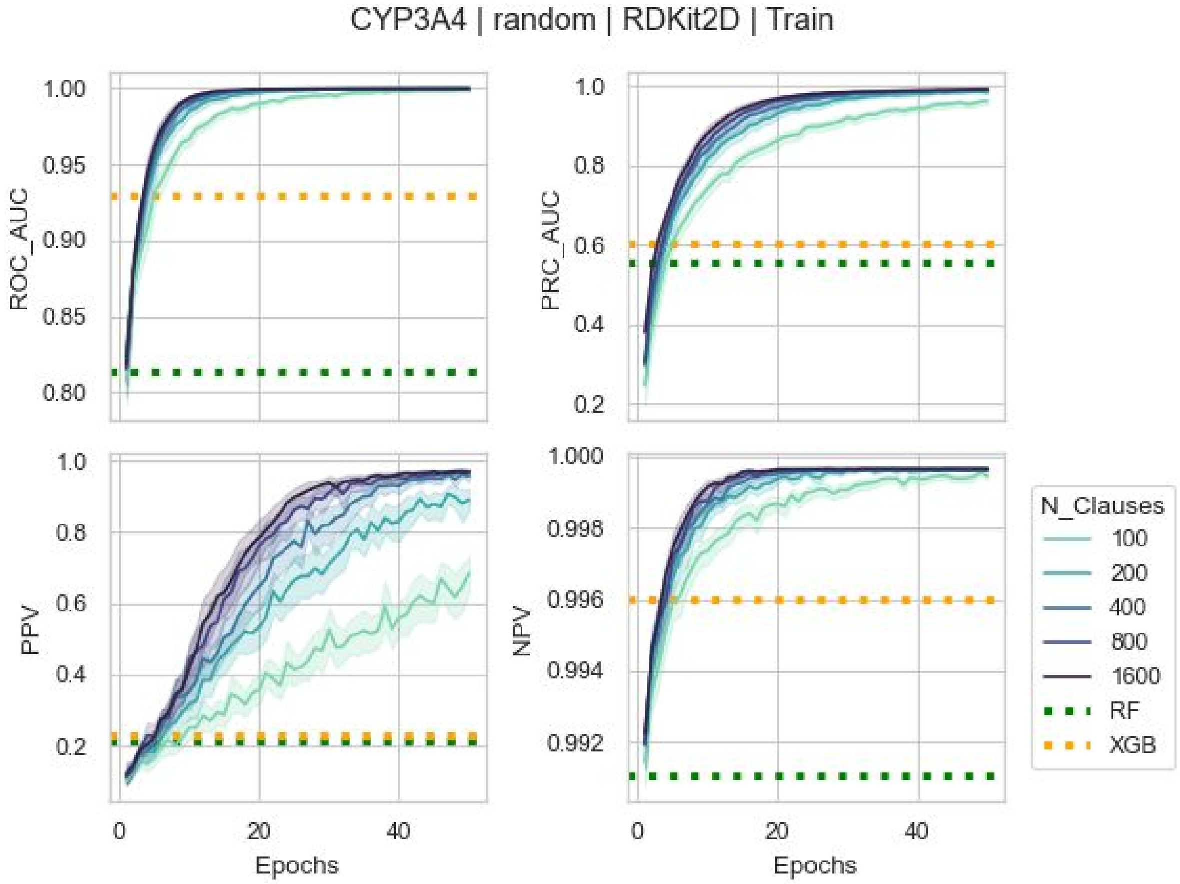

Figure S89: TM metric scores over 50 epochs on CYP3A4 training sets for random split-group and RDKit2D descriptors. Annotated by dotted lines are the mean training set scores of RF and XGBoost.

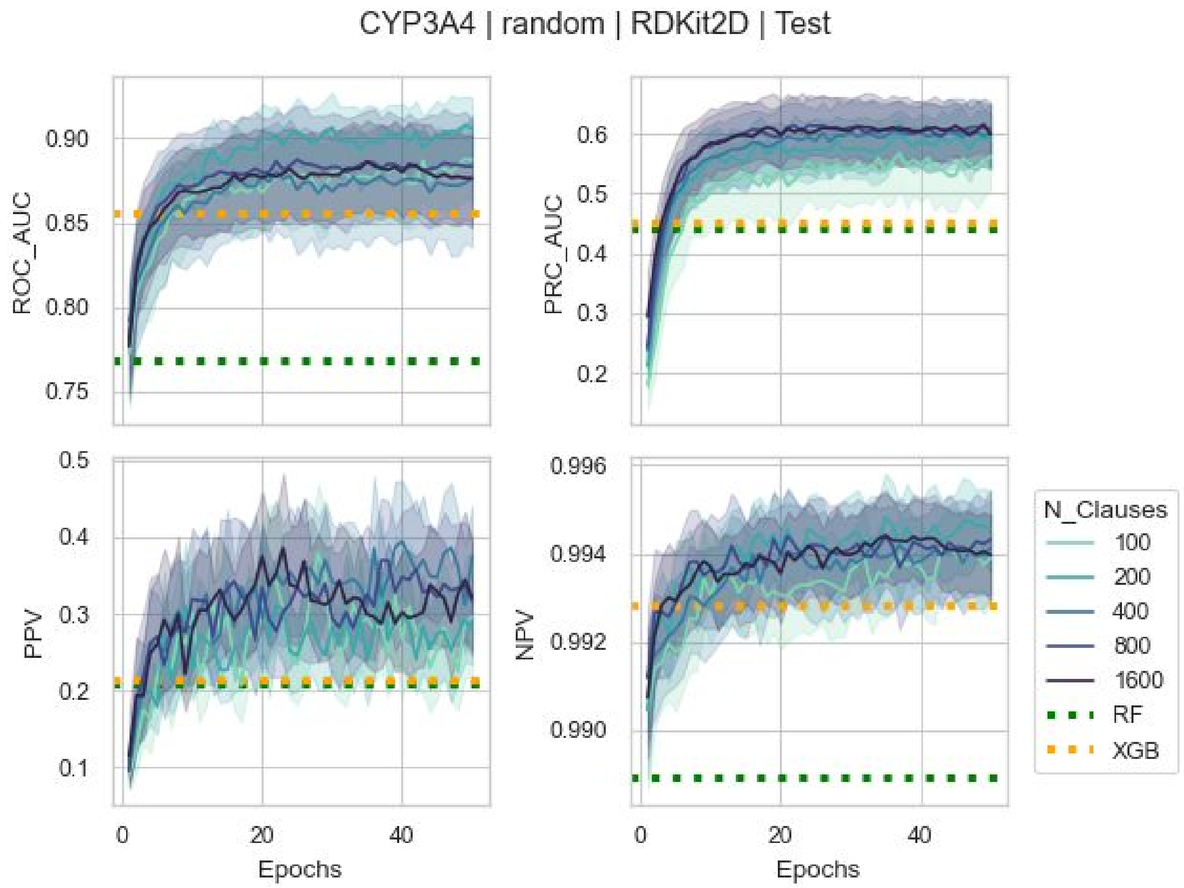

Figure S90: TM metric scores over 50 epochs on CYP3A4 test sets for random split-group and RDKit2D descriptors. Annotated by dotted lines are the mean training set scores of RF and XGBoost.

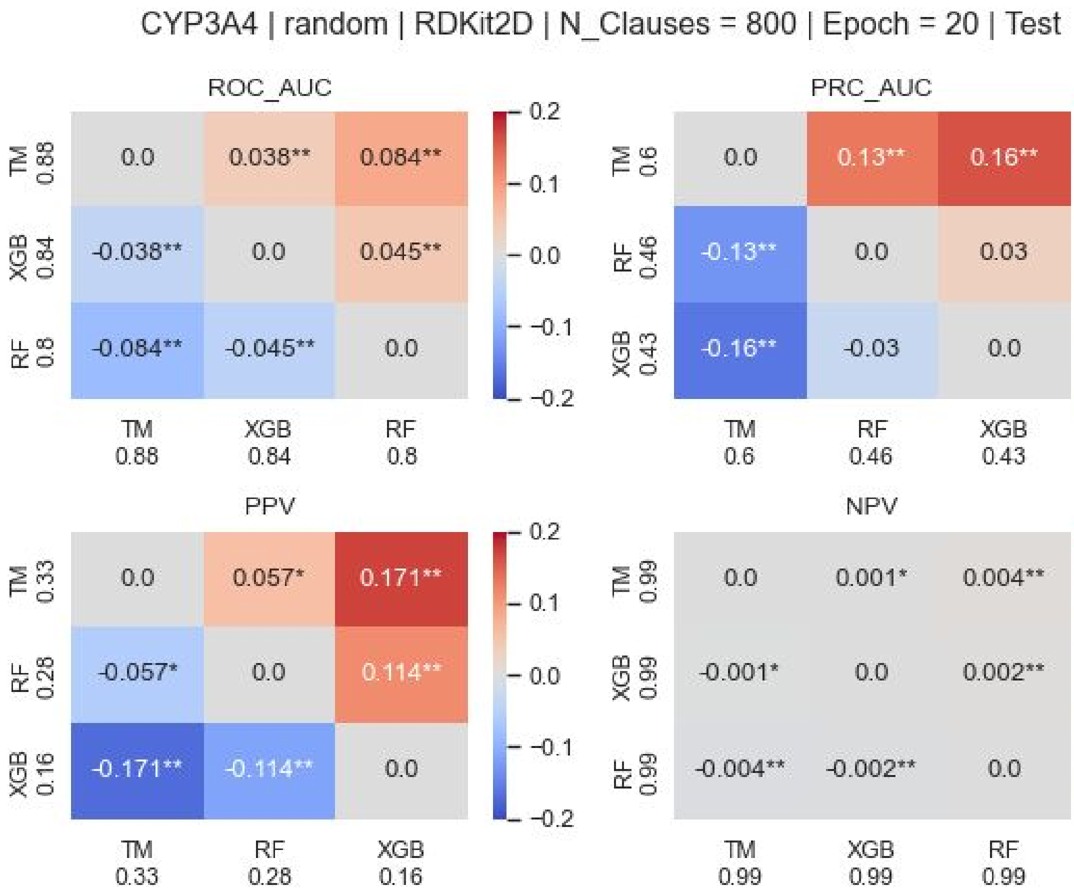

Figure S91: Cohen's D difference of means, pair-wise comparison of models for the CYP3A4 dataset with random group-split, RDKit2D descriptors and TM models of 800 clauses at 20 epochs. Complete with annotated statistical tests via Tukey's HSD where the number of asterix represents a different statistical significance level.

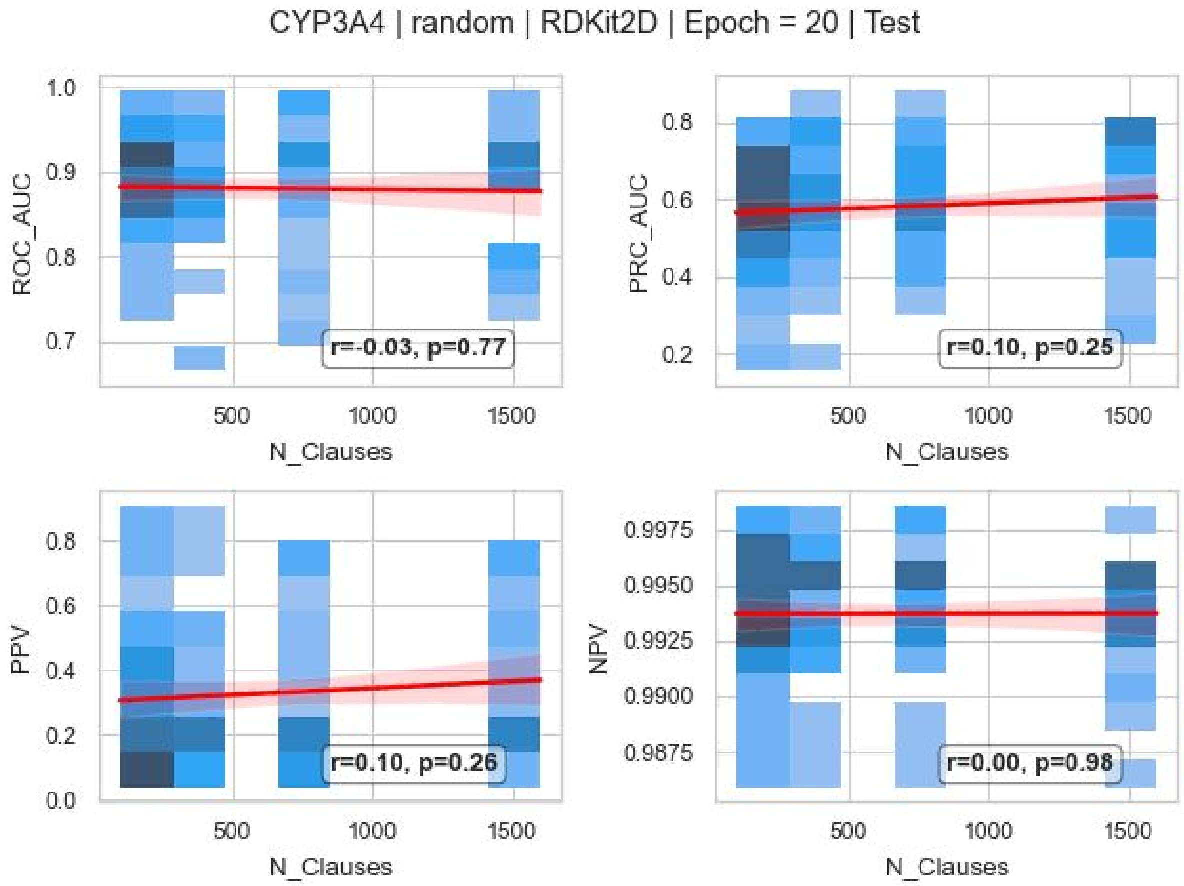

Figure S92: Number of clauses and metric-score histograms of CYP3A4 test sets with fitted line for random split-group, RDKit2D descriptors and TM-models of 800 clauses at 20 epochs. Pearson's R and p-value are annotated for said line.

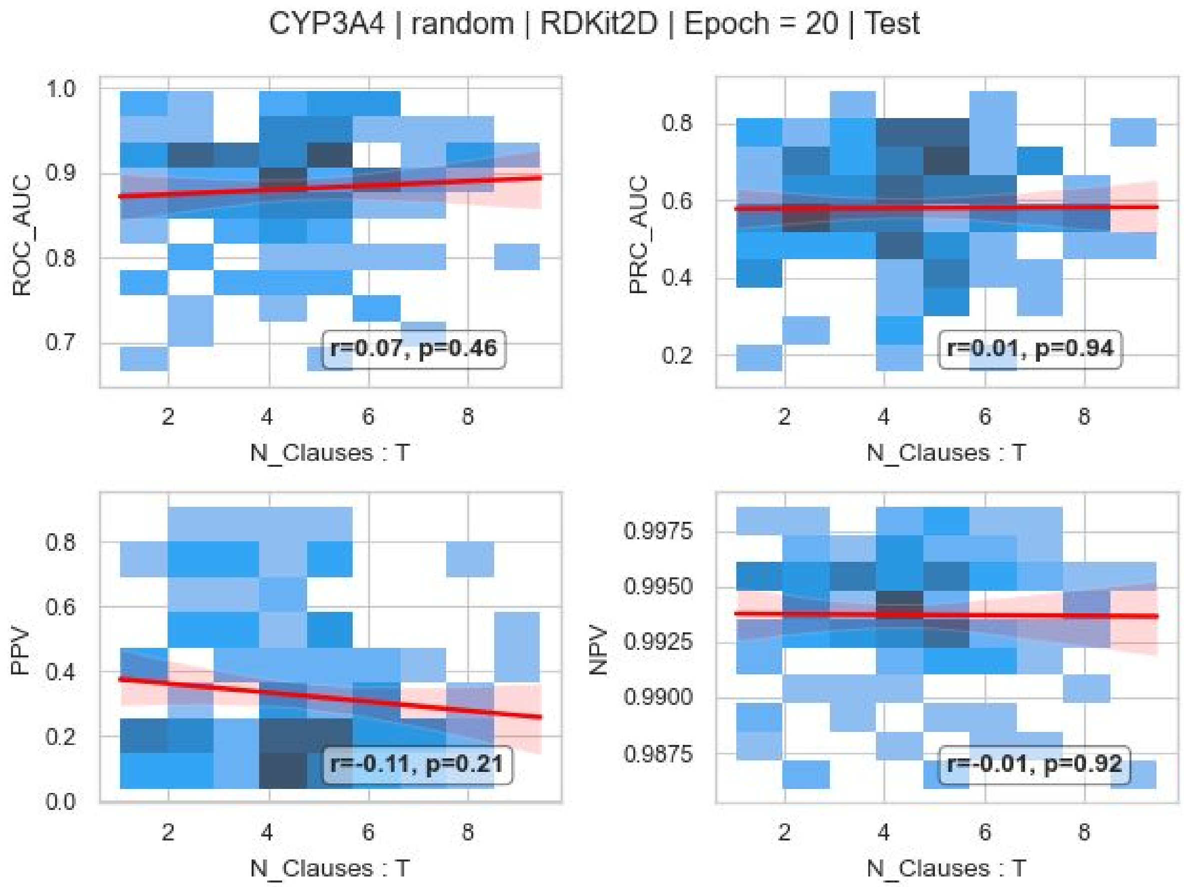

Figure S93: *N_Clauses : T* ratio and test-set metric scores histogram of CYP3A4 test sets with fitted line for random split-group, RDKit2D descriptors and TM-models of 800 clauses at 20 epochs. Pearson's R and p-value are annotated for said line.

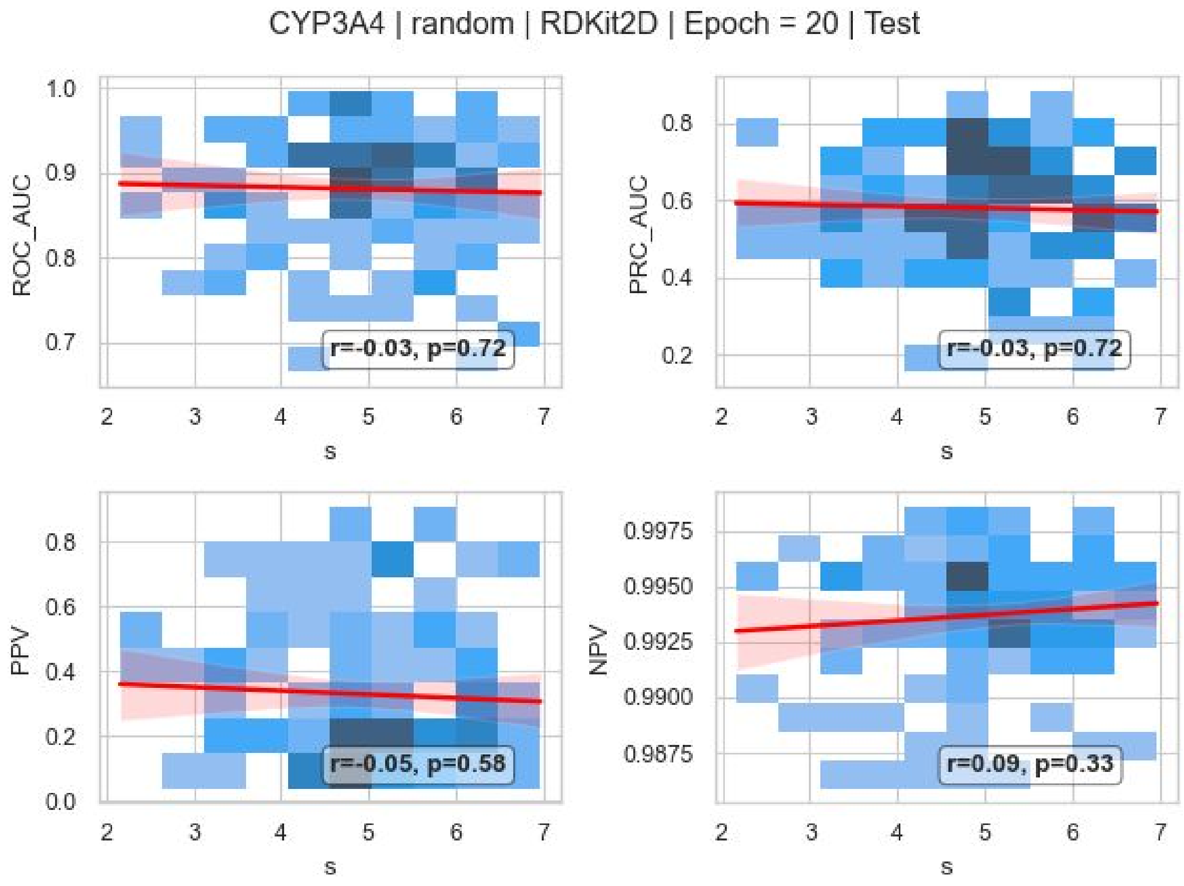

Figure S94: Hyper-parameter *s* and test-set metric scores histogram of CYP3A4 test sets with fitted line for random split-group, RDKit2D descriptors and TM-models of 800 clauses at 20 epochs. Pearson's R and p-value are annotated for said line.

scaffold group-split | RDKit2D Descriptors

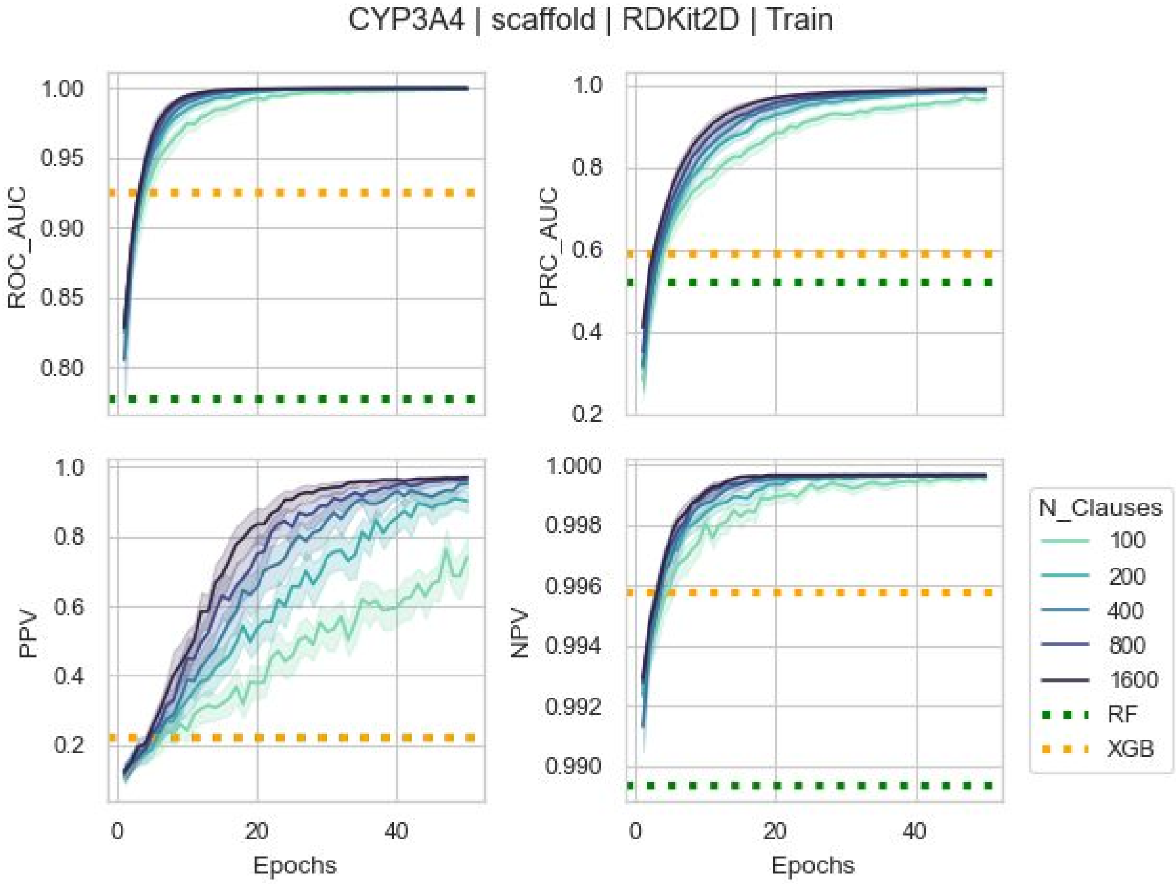

Figure S95: TM metric scores over 50 epochs on CYP3A4 training sets for scaffold split-group and RDKit2D descriptors. Annotated by dotted lines are the mean training set scores of RF and XGBoost.

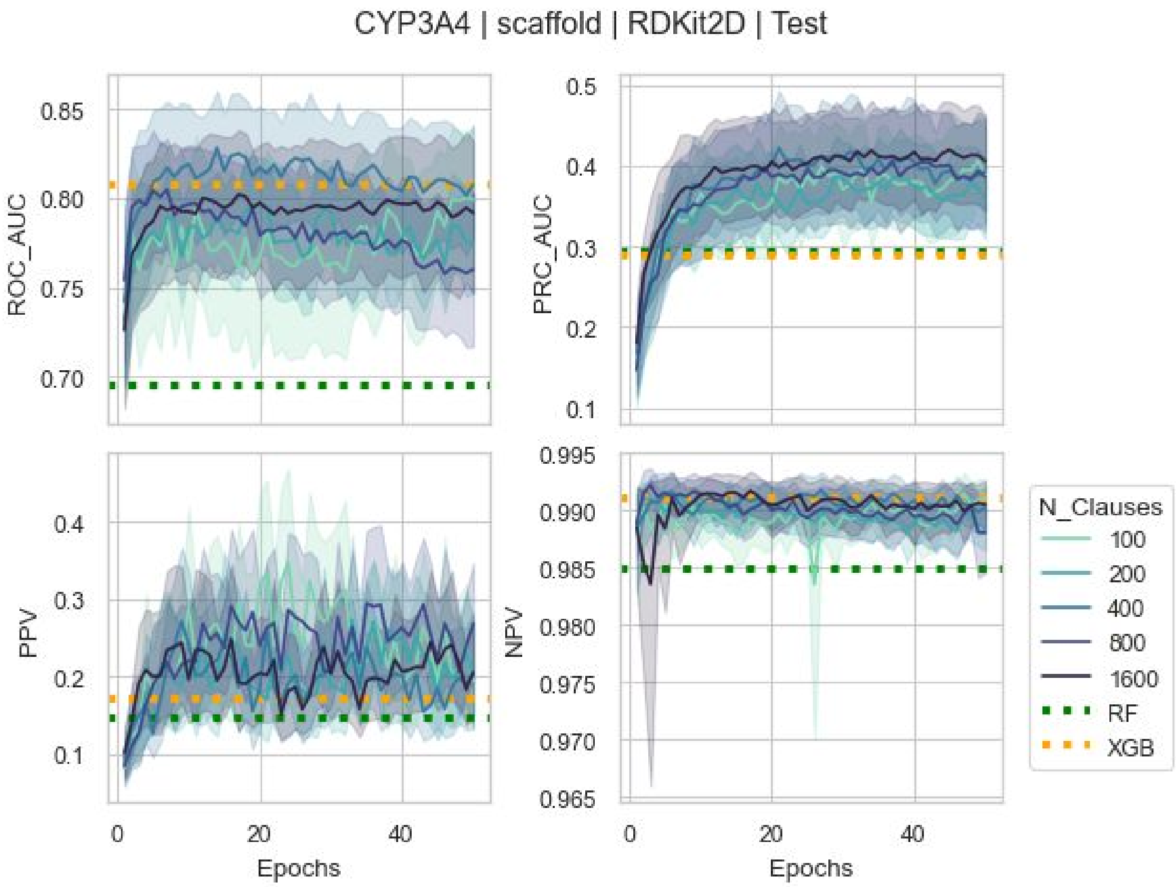

Figure S96: TM metric scores over 50 epochs on CYP3A4 test sets for scaffold split-group and RDKit2D descriptors. Annotated by dotted lines are the mean training set scores of RF and XGBoost.

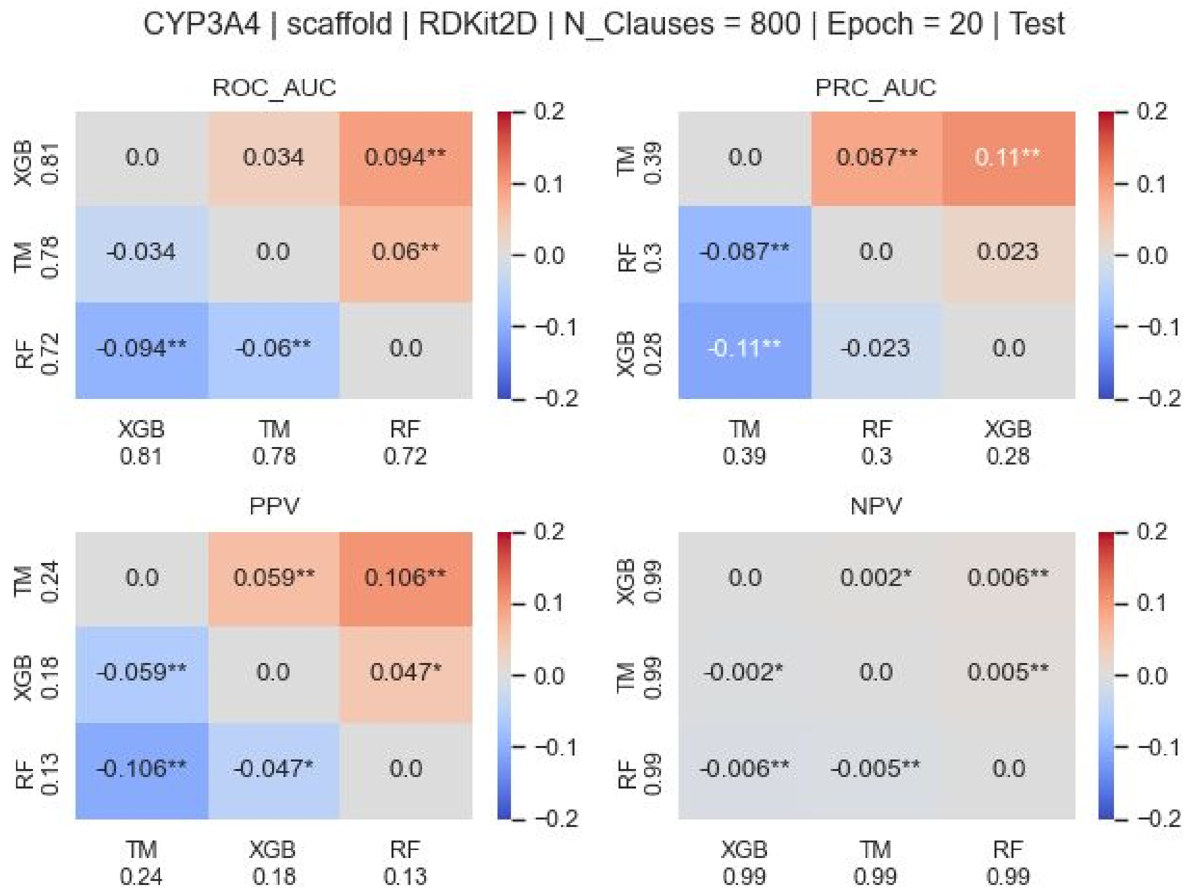

Figure S97: Cohen's D difference of means, pair-wise comparison of models for the CYP3A4 dataset with scaffold group-split, RDKit2D descriptors and TM models of 800 clauses at 20 epochs. Complete with annotated statistical tests via Tukey's HSD where the number of asterix represents a different statistical significance level.

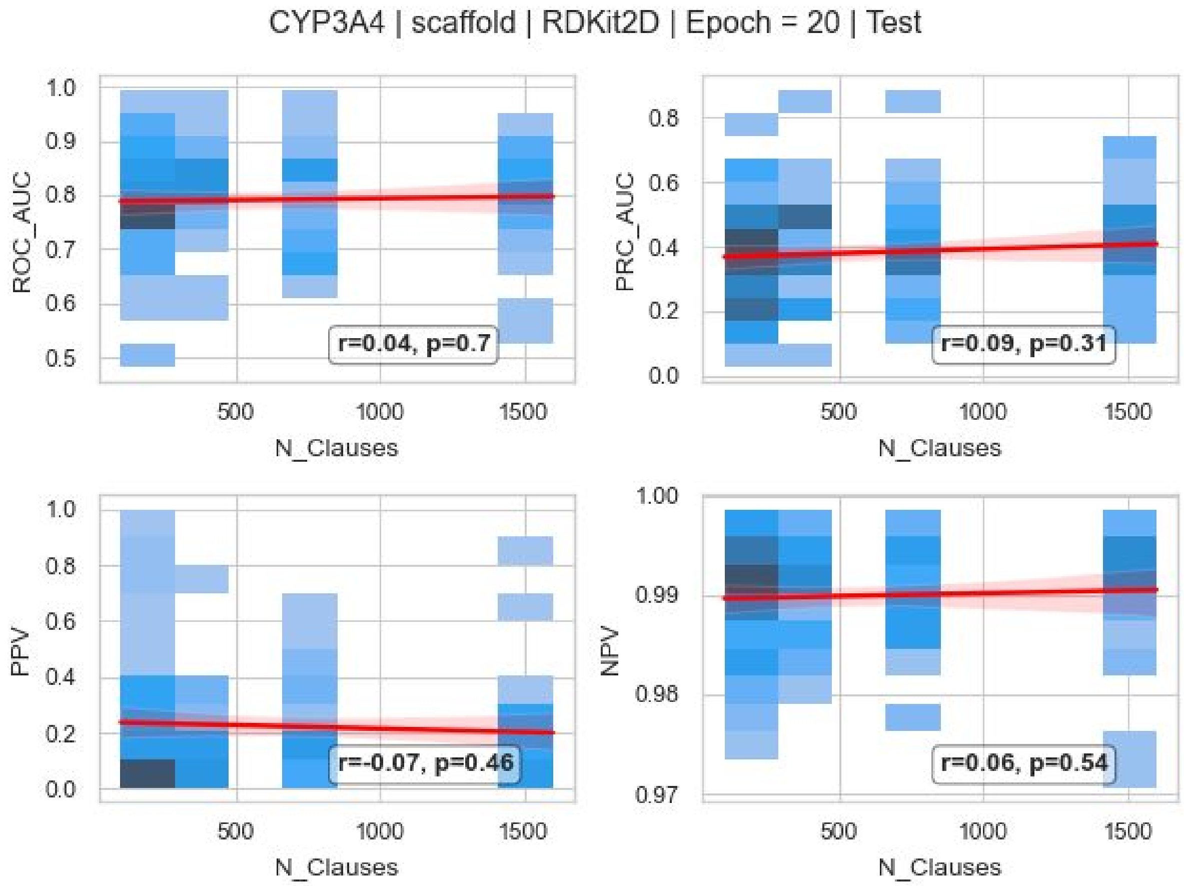

Figure S98: Number of clauses and metric-score histograms of CYP3A4 test sets with fitted line for scaffold split-group, RDKit2D descriptors and TM-models of 800 clauses at 20 epochs. Pearson's R and p-value are annotated for said line.

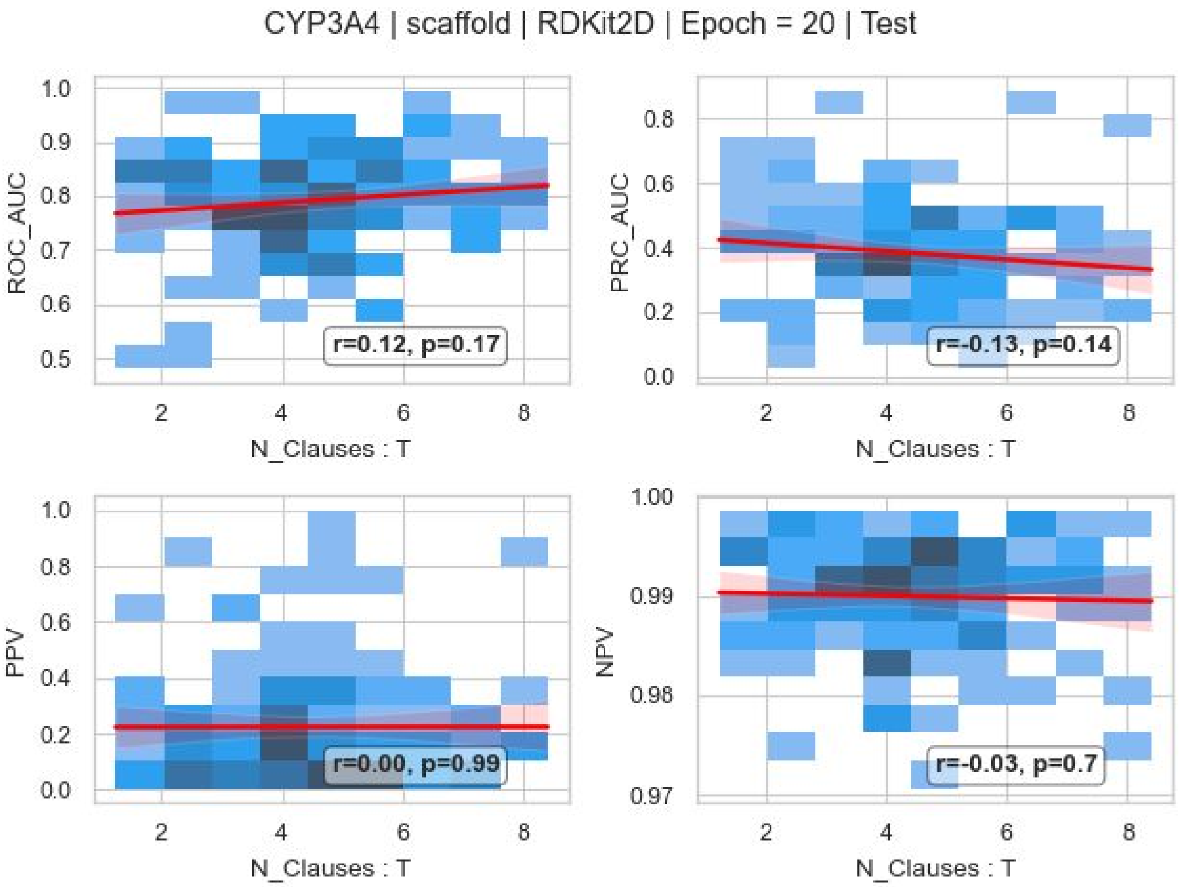

Figure S99: *N_Clauses : T* ratio and test-set metric scores histogram of CYP3A4 test sets with fitted line for scaffold split-group, RDKit2D descriptors and TM-models of 800 clauses at 20 epochs. Pearson's R and p-value are annotated for said line.

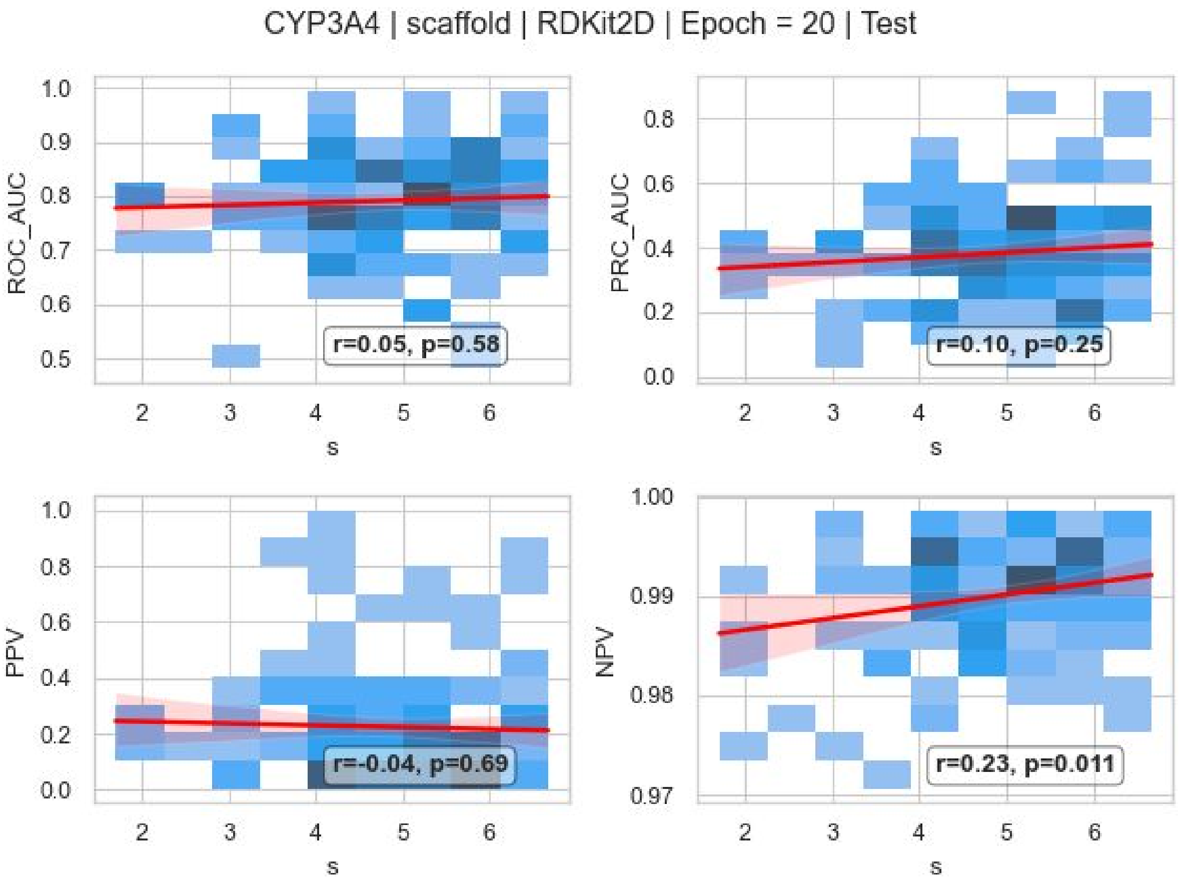

Figure S100: Hyper-parameter *s* and test-set metric scores histogram of CYP3A4 test sets with fitted line for scaffold split-group, RDKit2D descriptors and TM-models of 800 clauses at 20 epochs. Pearson's R and p-value are annotated for said line.

CYP2D6

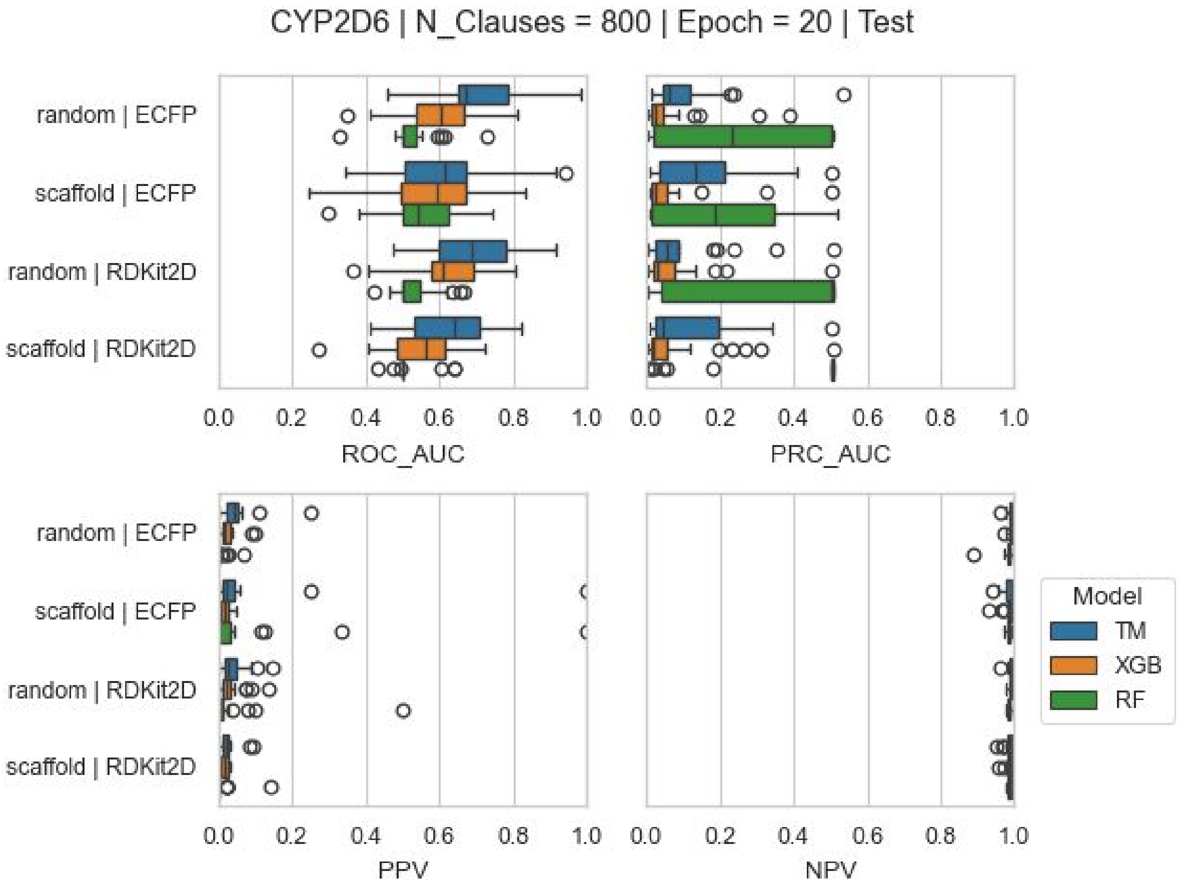

Figure S101: Box plot of model metric scores on a test set across split-group | descriptor pairs for CYP2D6 dataset. The TM uses 800 clauses and learning is stopped after 20 epochs.

random split-group I ECFP Descriptors

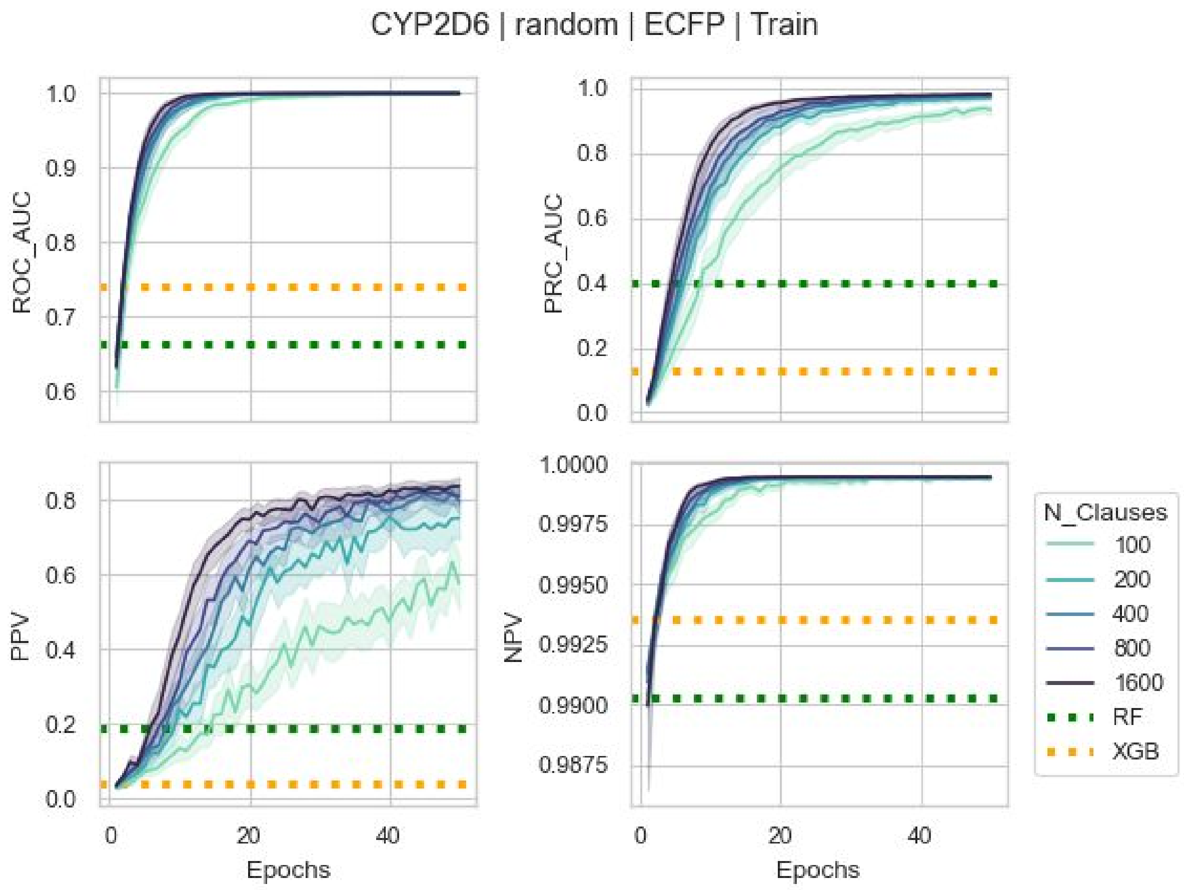

Figure S102: TM metric scores over 50 epochs on CYP2D6 training sets for random split-group and ECFP descriptors. Annotated by dotted lines are the mean training set scores of RF and XGBoost.

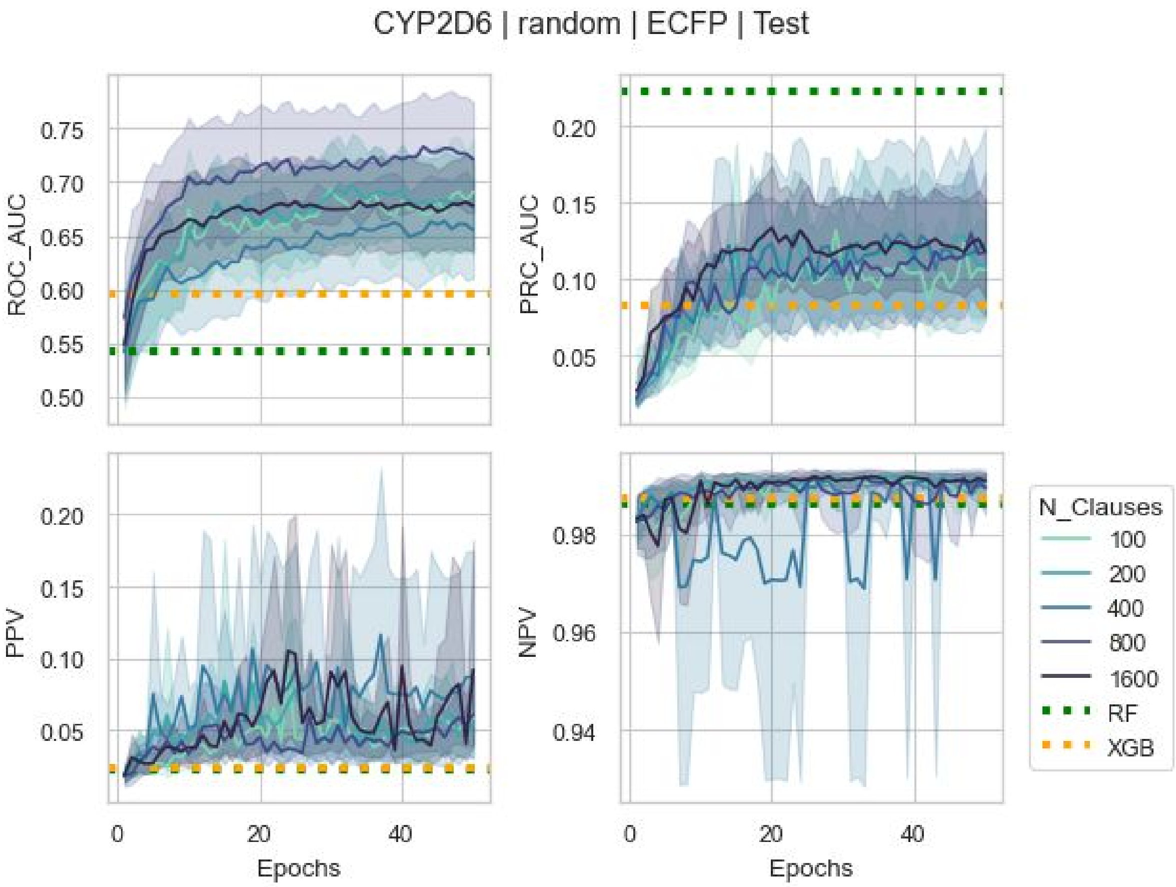

Figure S103: TM metric scores over 50 epochs on CYP2D6 test sets for random split-group and ECFP descriptors. Annotated by dotted lines are the mean test set scores of RF and XGBoost.

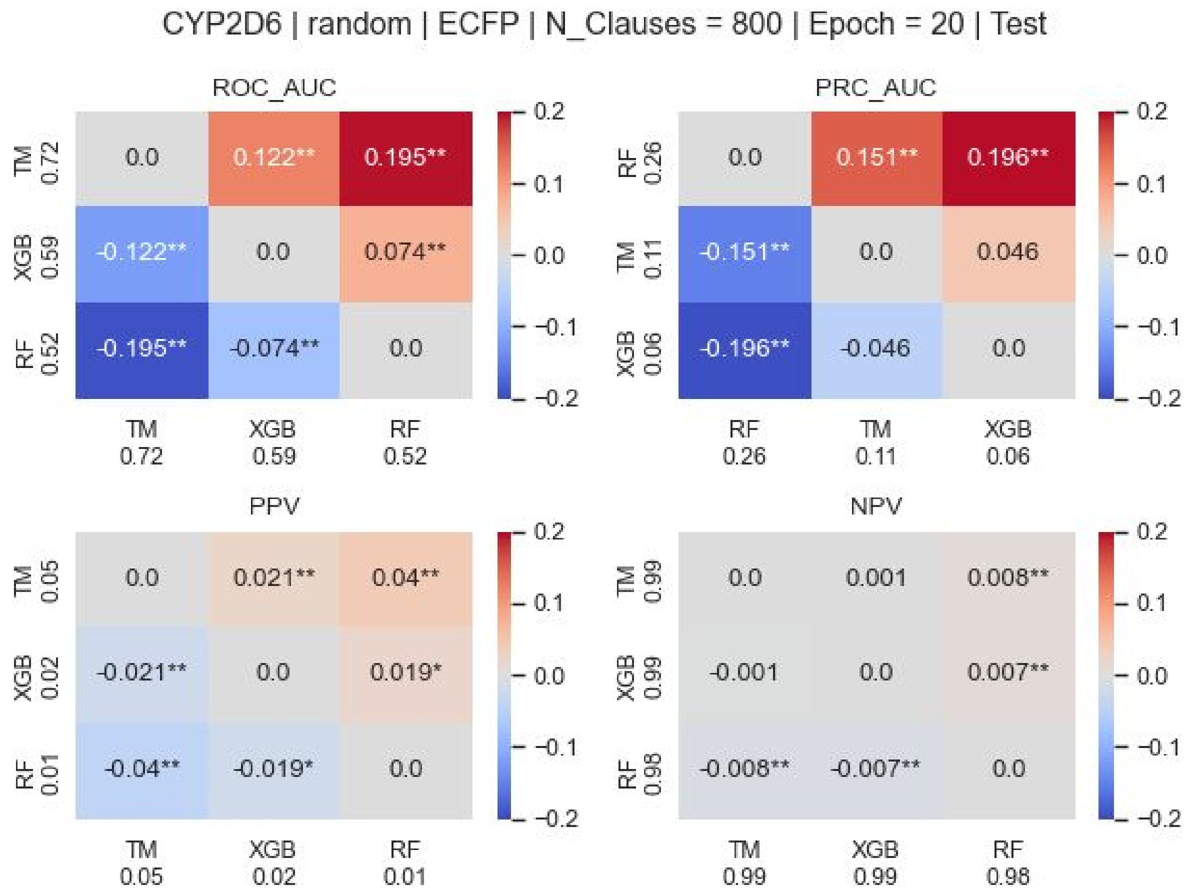

Figure S104: Cohen's D difference of means, pair-wise comparison of models for the CYP2D6 dataset with random group-split, ECFP descriptors and TM models of 800 clauses at 20 epochs. Complete with annotated statistical tests via Tukey's HSD where the number of asterix represents a different statistical significance level.

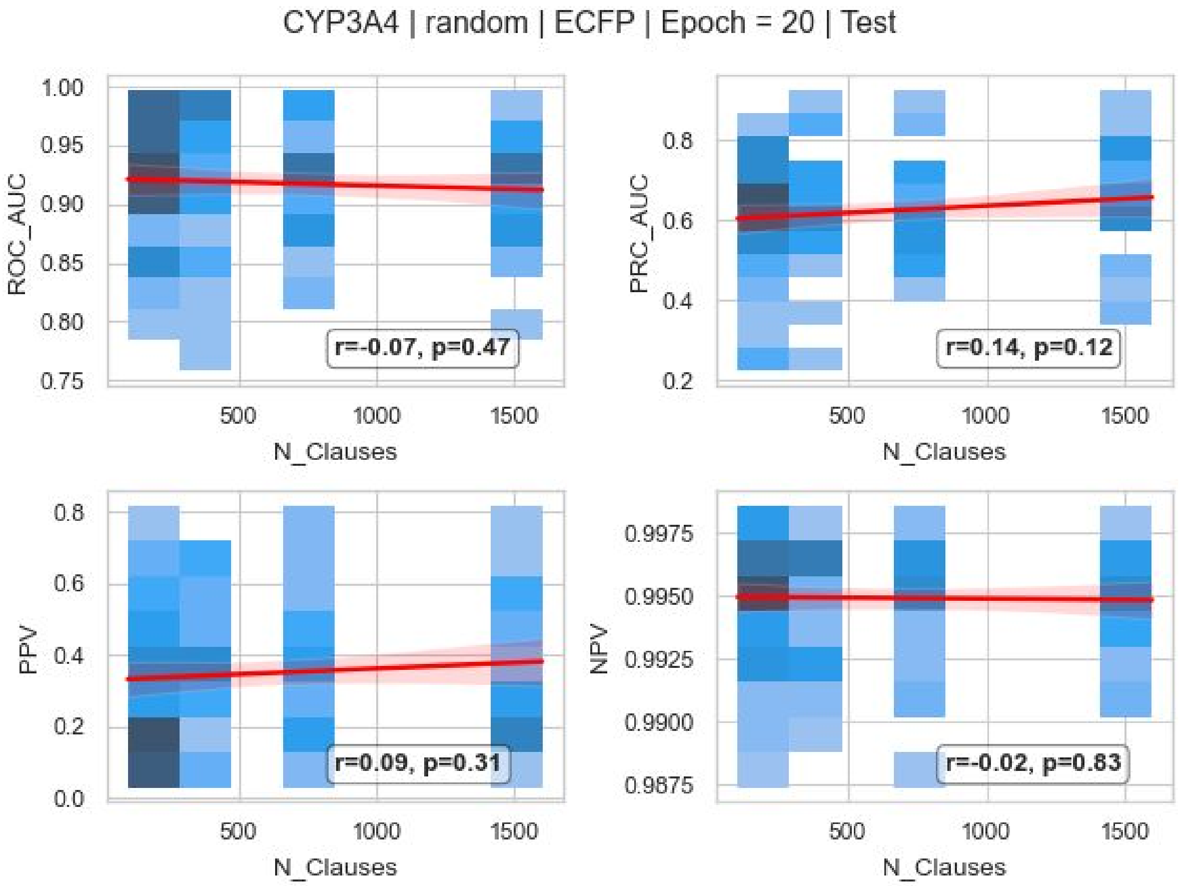

Figure S105: Number of clauses and metric-score histograms of CYP2D6 test sets with fitted line for random split-group, ECFP descriptors and TM-models of 800 clauses at 20 epochs. Pearson's R and p-value are annotated for said line.

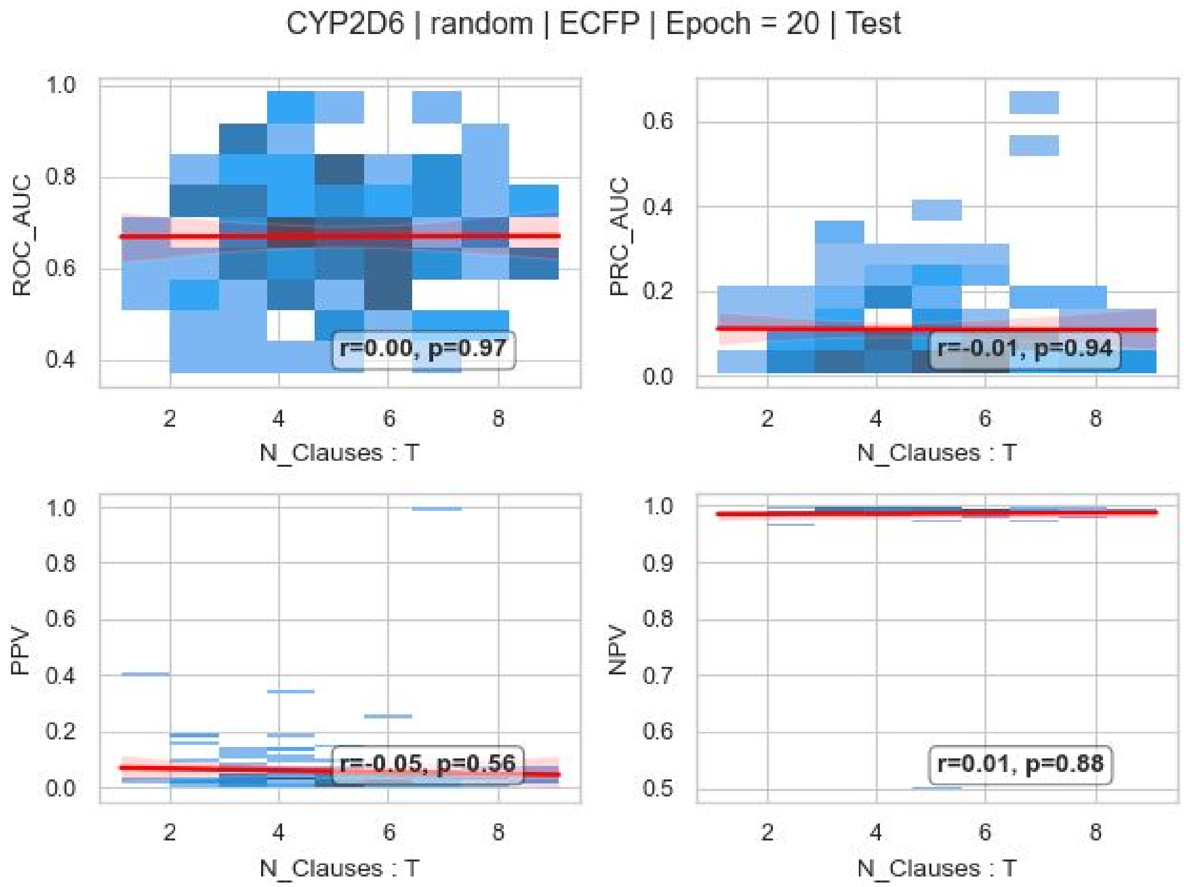

Figure S106: *N_Clauses : T* ratio and test-set metric scores histogram for CYP2D6 dataset with fitted line for random split-group, ECFP descriptors and TM-models of 800 clauses at 20 epochs. Pearson's R and p-value are annotated for said line.

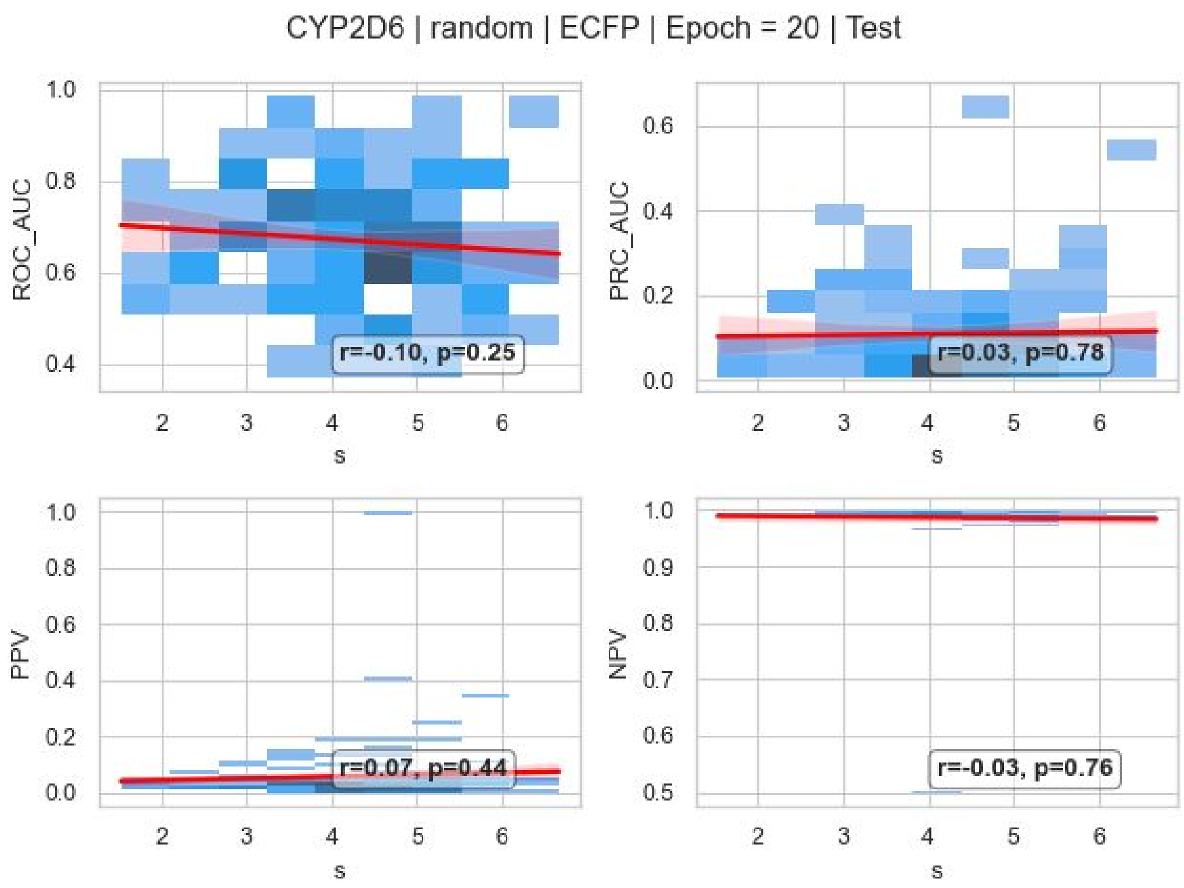

Figure S107: Hyper-parameter *s* and test-set metric scores histogram for CYP2D6 dataset with fitted line for random split-group, ECFP descriptors and TM-models of 800 clauses at 20 epochs. Pearson's R and p-value are annotated for said line.

scaffold split-group | ECFP Descriptors

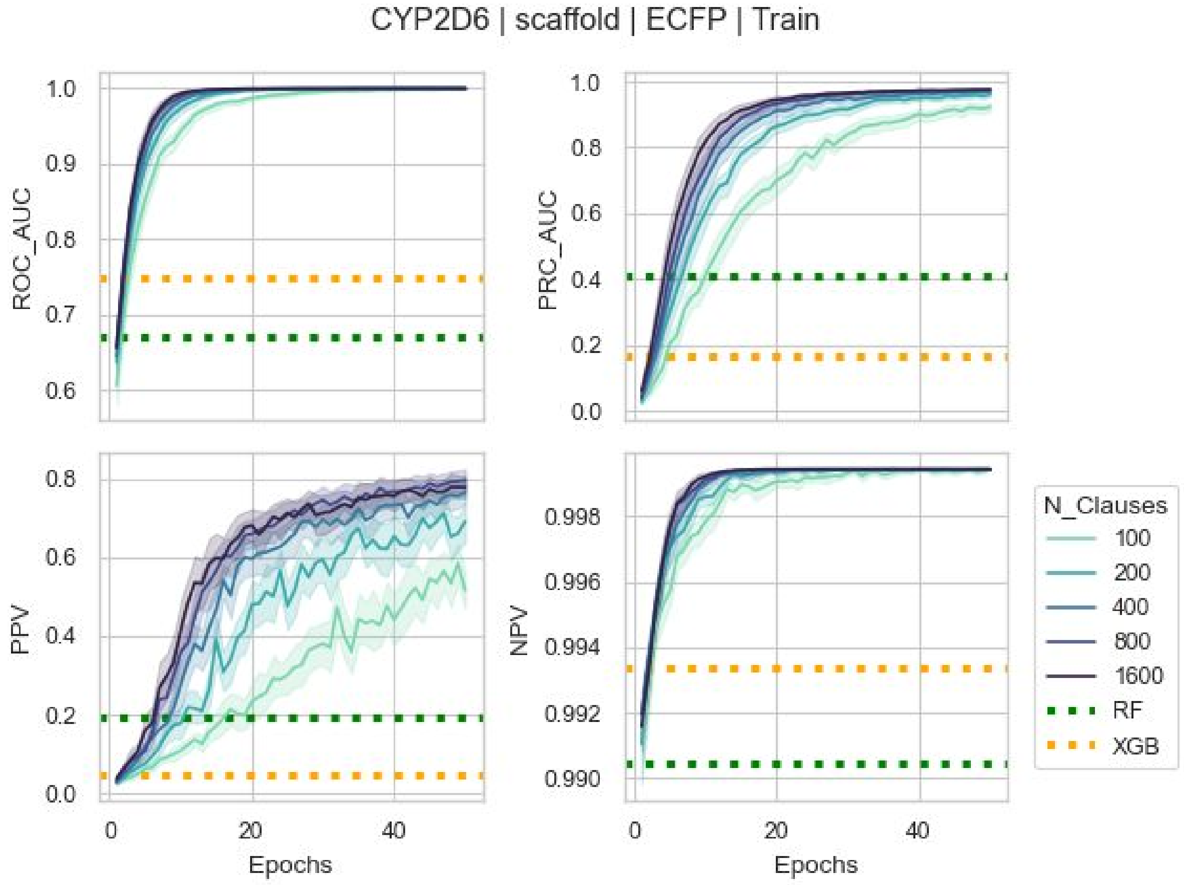

Figure S108: TM metric scores over 50 epochs on CYP2D6 training sets for scaffold split-group and ECFP descriptors. Annotated by dotted lines are the mean training set scores of RF and XGBoost.

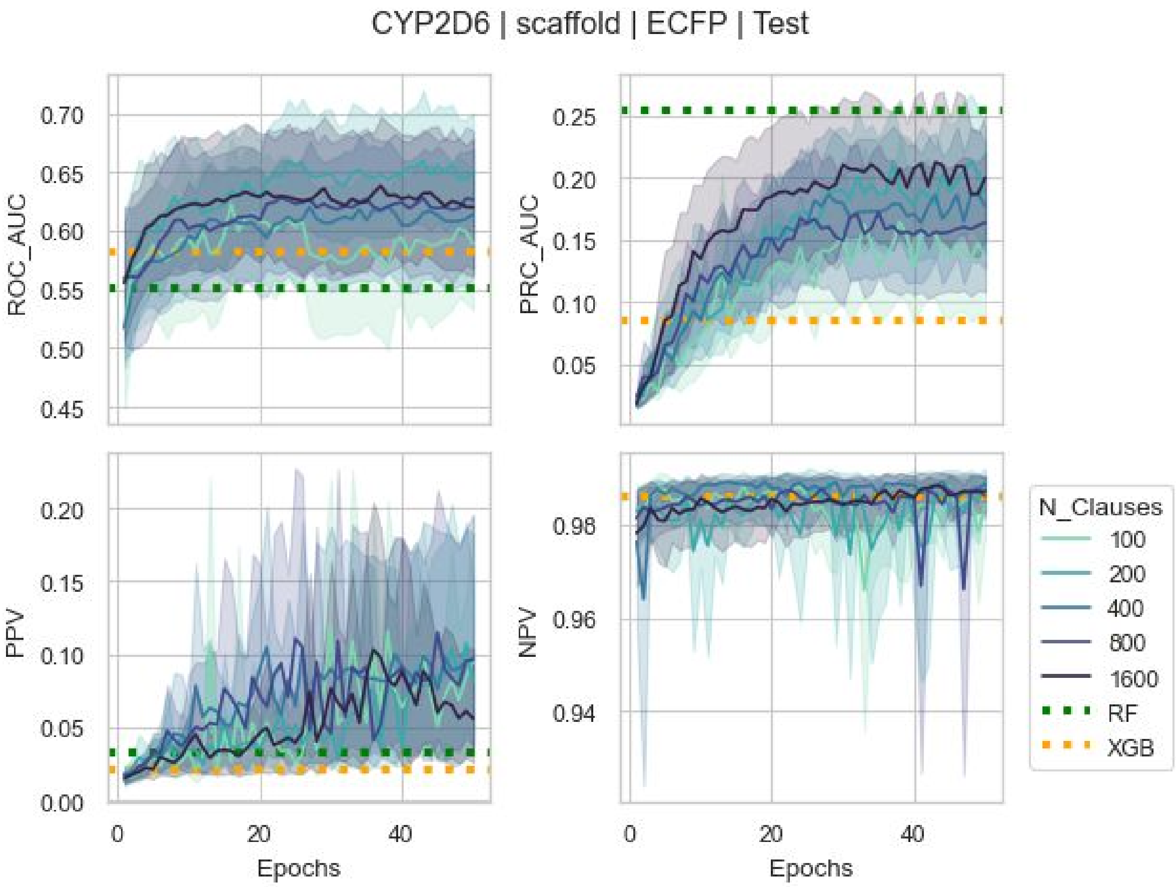

Figure S109: TM metric scores over 50 epochs on CYP2D6 test sets for scaffold split-group and ECFP descriptors. Annotated by dotted lines are the mean training set scores of RF and XGBoost.

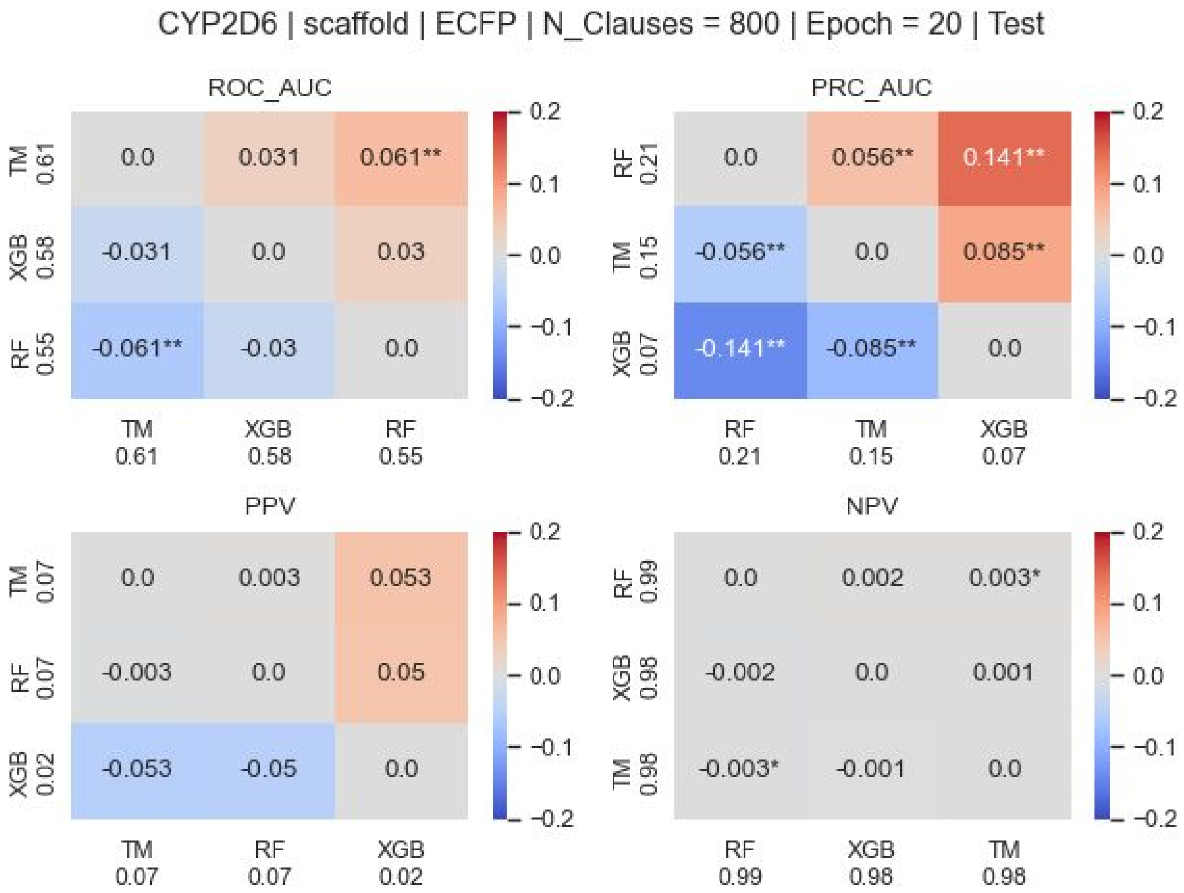

Figure S110: Cohen's D difference of means, pair-wise comparison of models for the CYP2D6 dataset with scaffold group-split, ECFP descriptors and TM models of 800 clauses at 20 epochs. Complete with annotated statistical tests via Tukey's HSD where the number of asterix represents a different statistical significance level.

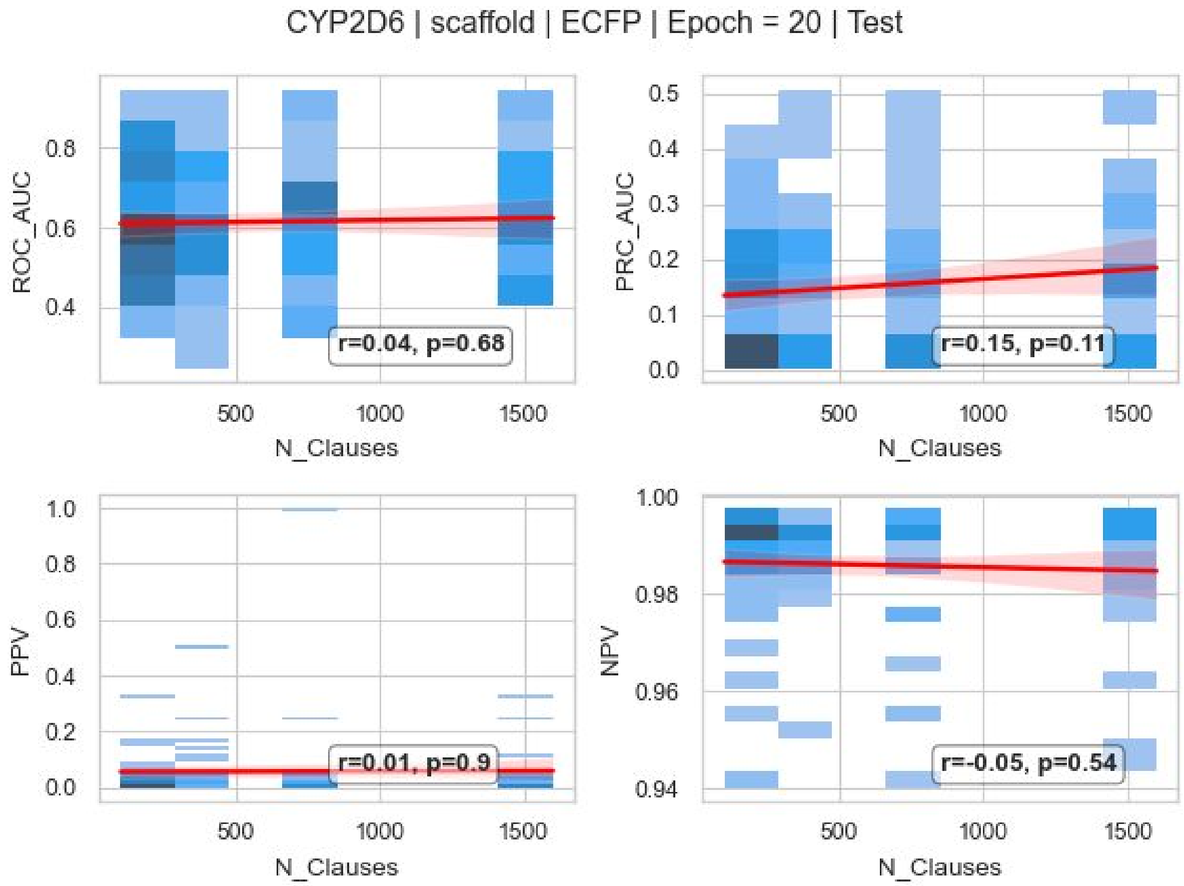

Figure S111: Number of clauses and metric-score histograms of CYP2D6 test sets with fitted line for scaffold split-group, ECFP descriptors and TM-models of 800 clauses at 20 epochs. Pearson's R and p-value are annotated for said line.

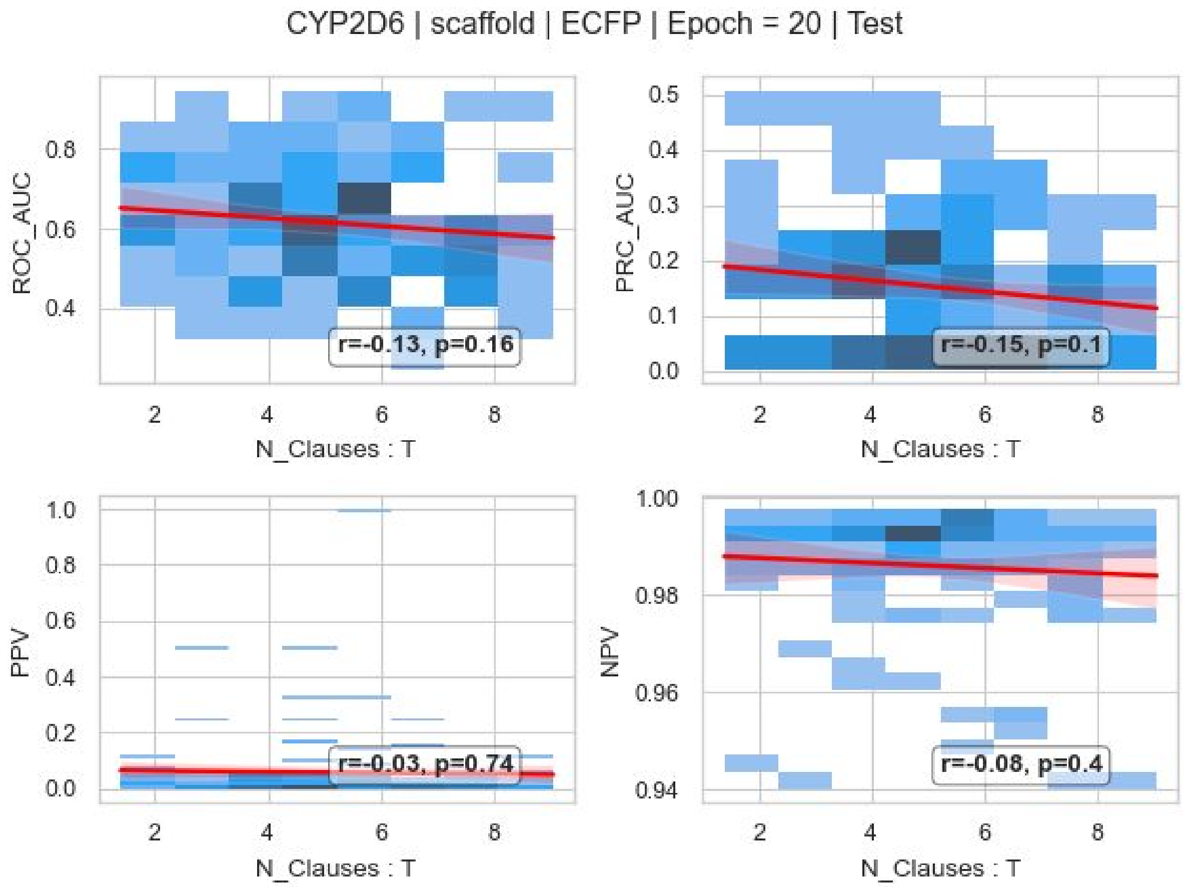

Figure S112: *N_Clauses : T* ratio and test-set metric scores histogram of CYP2D6 test sets with fitted line for scaffold split-group, ECFP descriptors and TM-models of 800 clauses at 20 epochs. Pearson's R and p-value are annotated for said line.

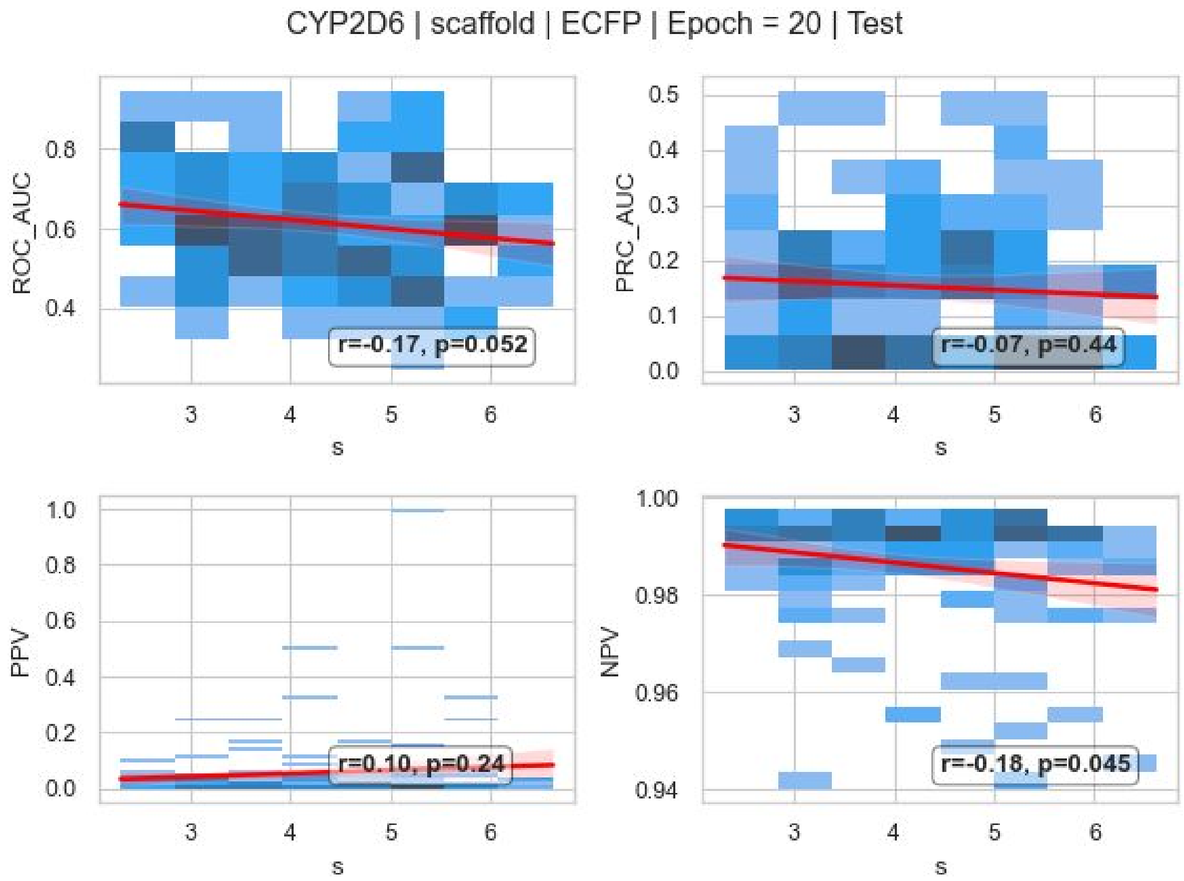

Figure S113: Hyper-parameter s and test-set metric scores histogram of CYP2D6 test sets with fitted line for scaffold split-group, ECFP descriptors and TM-models of 800 clauses at 20 epochs. Pearson's R and p-value are annotated for said line.

random split-group | RDKit2D Descriptors

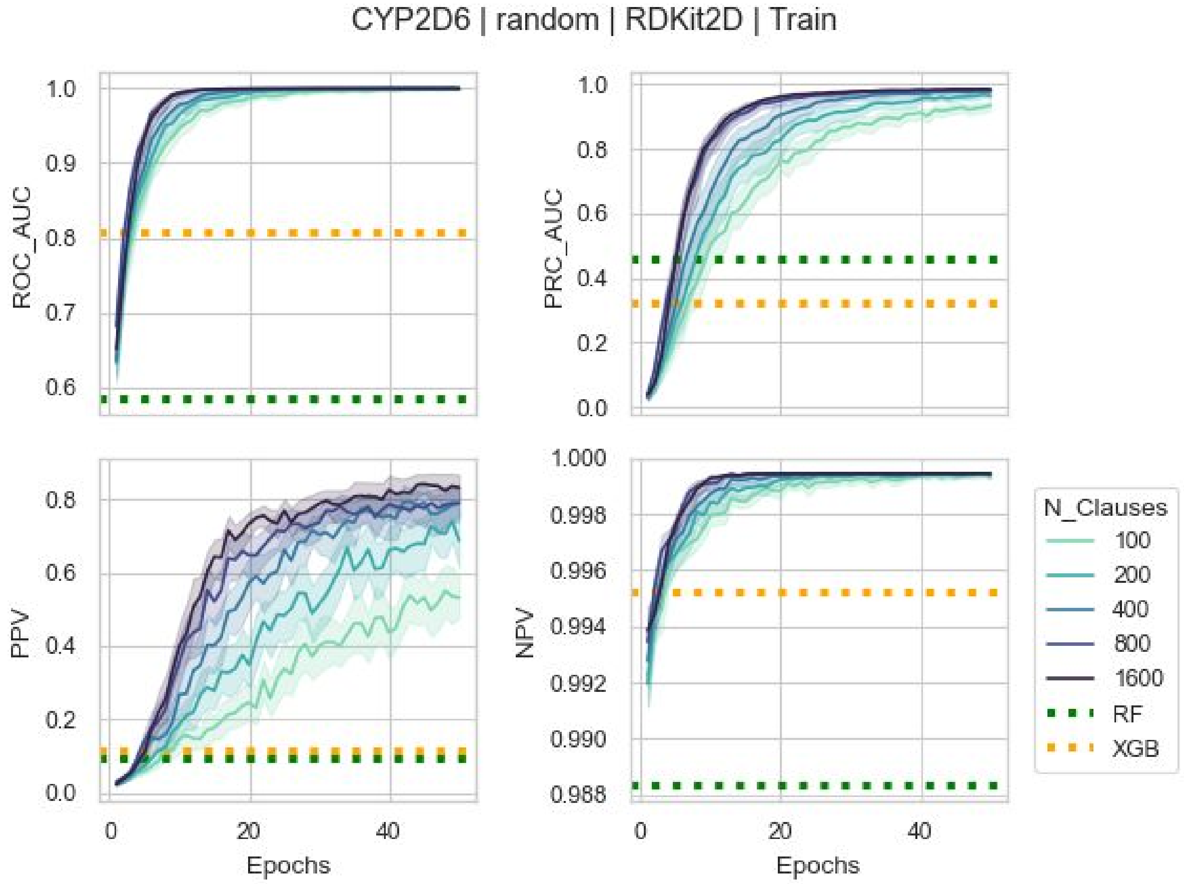

Figure S114: TM metric scores over 50 epochs on CYP2D6 training sets for random split-group and RDKit2D descriptors. Annotated by dotted lines are the mean training set scores of RF and XGBoost.

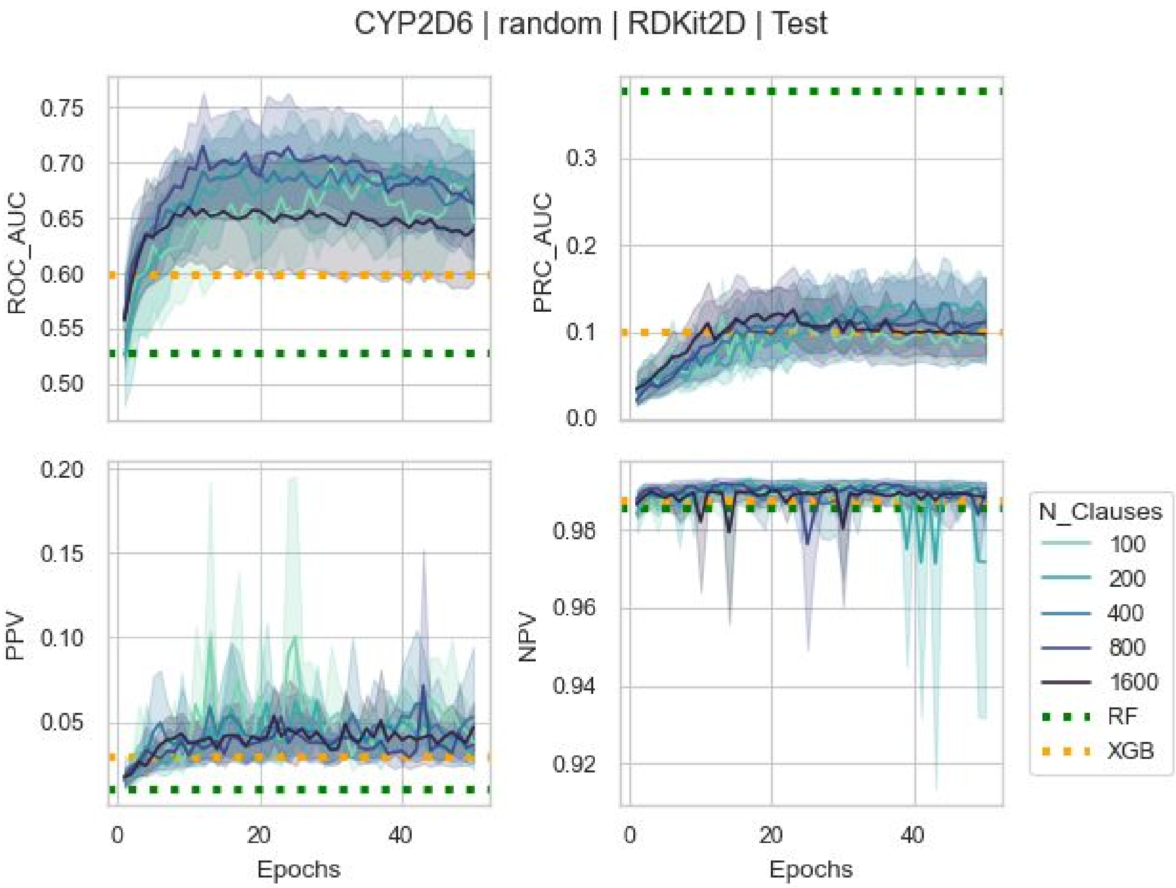

Figure S115: TM metric scores over 50 epochs on CYP2D6 test sets for random split-group and RDKit2D descriptors. Annotated by dotted lines are the mean training set scores of RF and XGBoost.

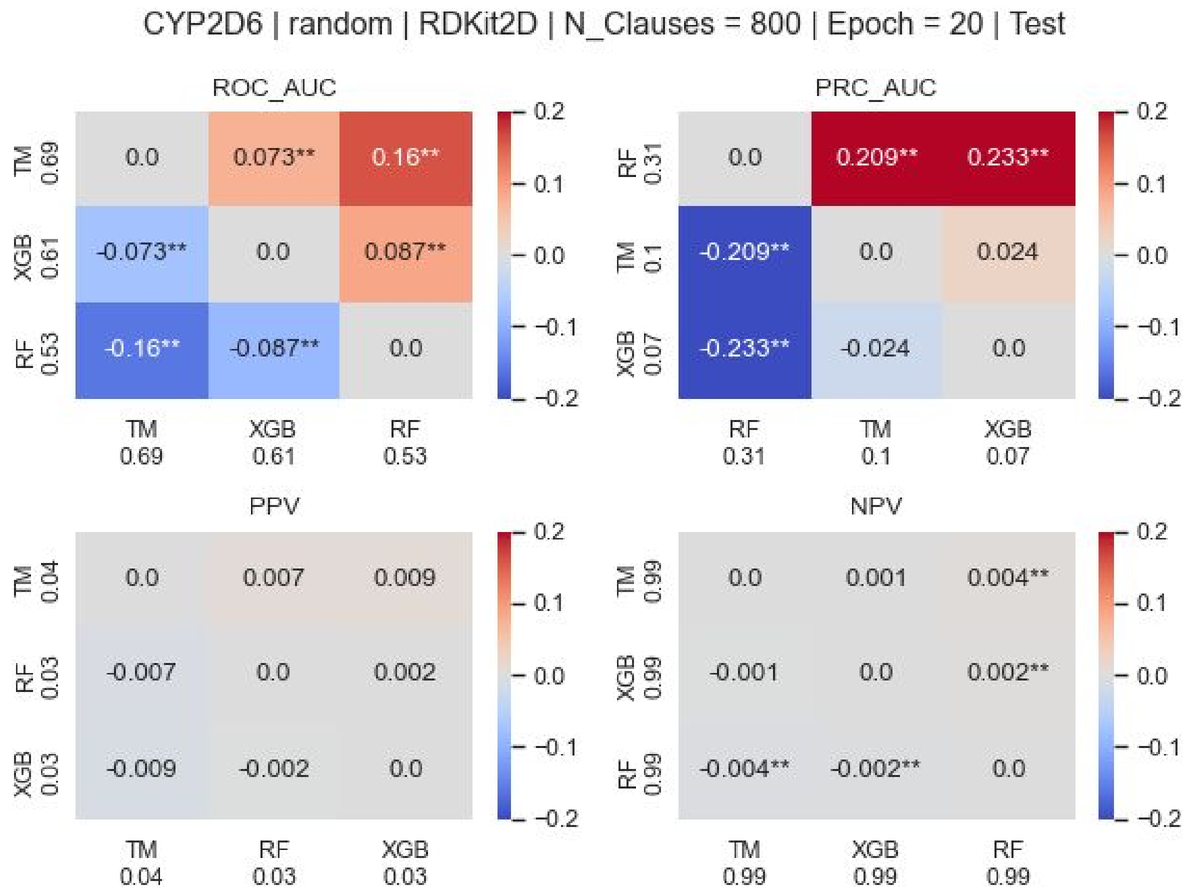

Figure S116: Cohen's D difference of means, pair-wise comparison of models for the CYP2D6 dataset with random group-split, RDKit2D descriptors and TM models of 800 clauses at 20 epochs. Complete with annotated statistical tests via Tukey's HSD where the number of asterix represents a different statistical significance level.

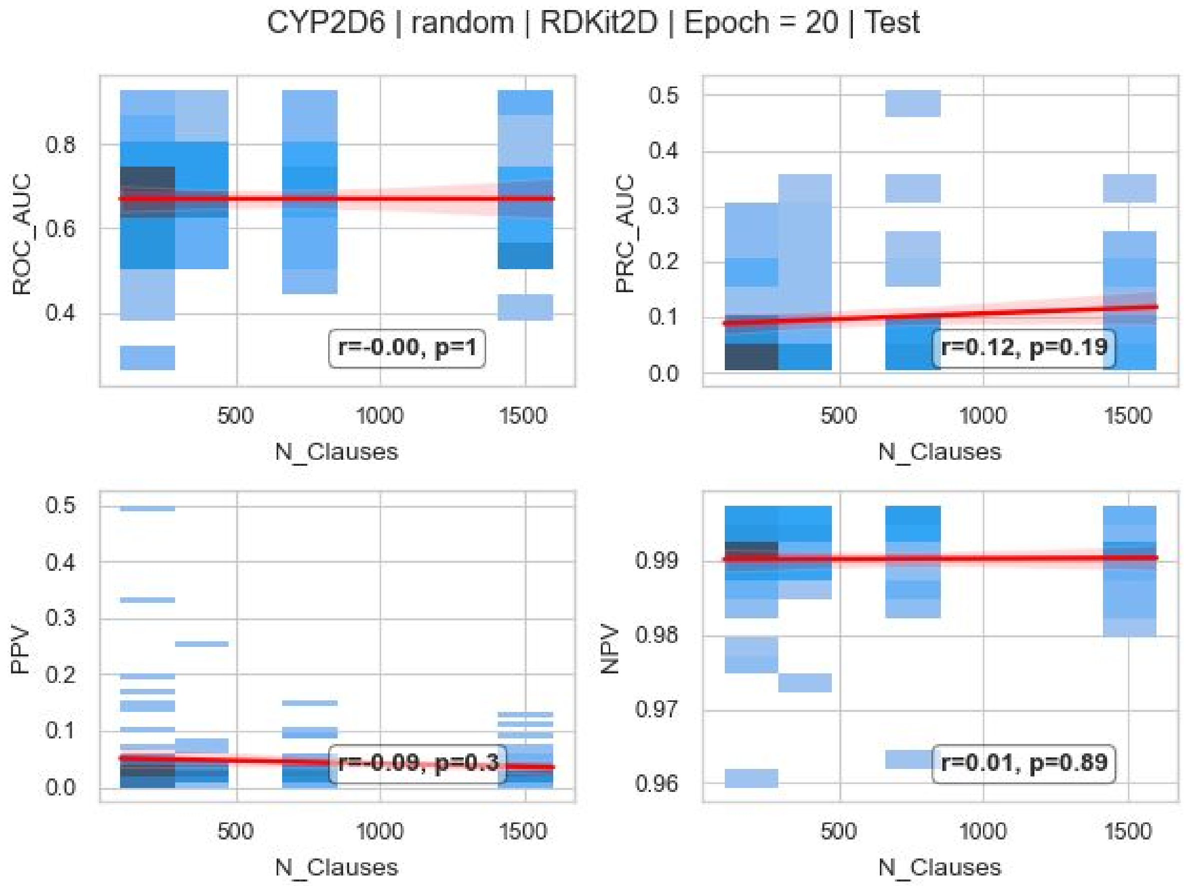

Figure S117: Number of clauses and metric-score histograms of CYP2D6 test sets with fitted line for random split-group, RDKit2D descriptors and TM-models of 800 clauses at 20 epochs. Pearson's R and p-value are annotated for said line.

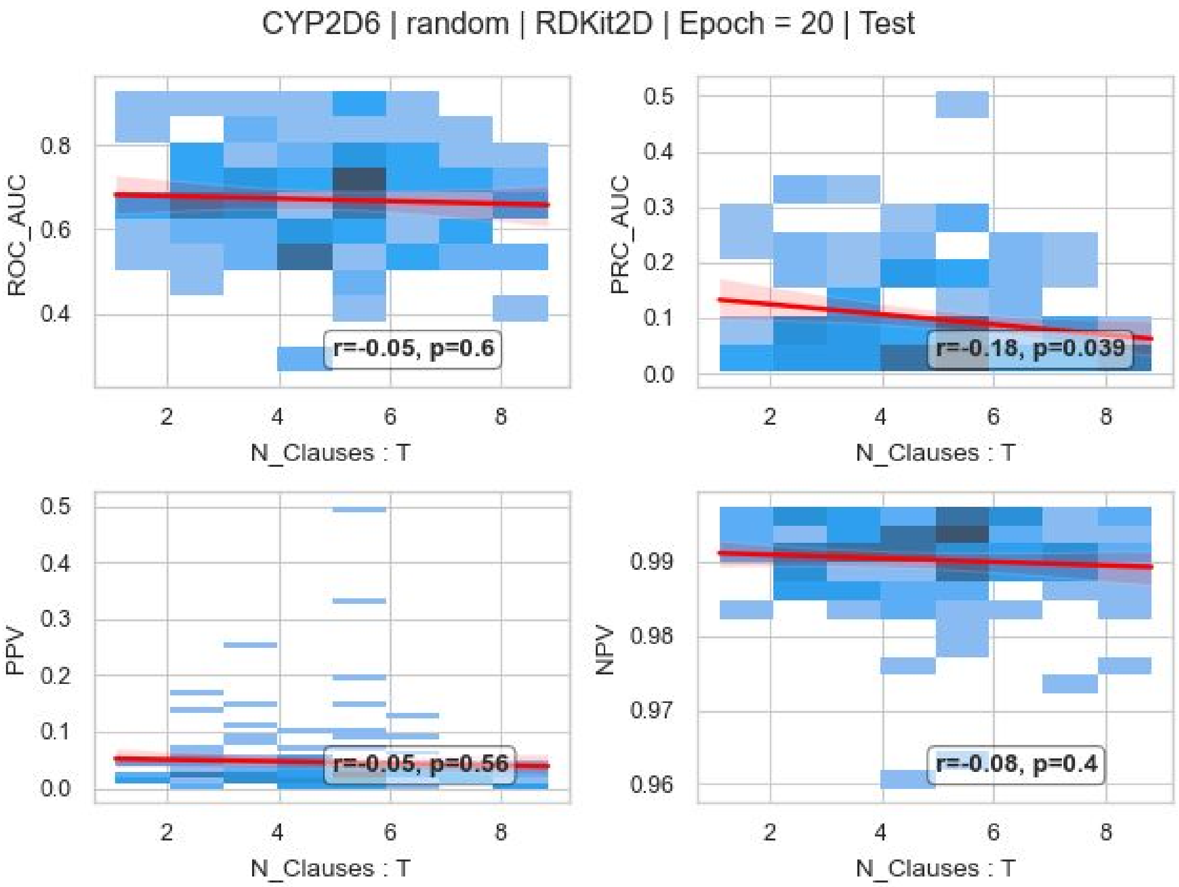

Figure S118: *N_Clauses : T* ratio and test-set metric scores histogram of CYP2D6 test sets with fitted line for random split-group, RDKit2D descriptors and TM-models of 800 clauses at 20 epochs. Pearson's R and p-value are annotated for said line.

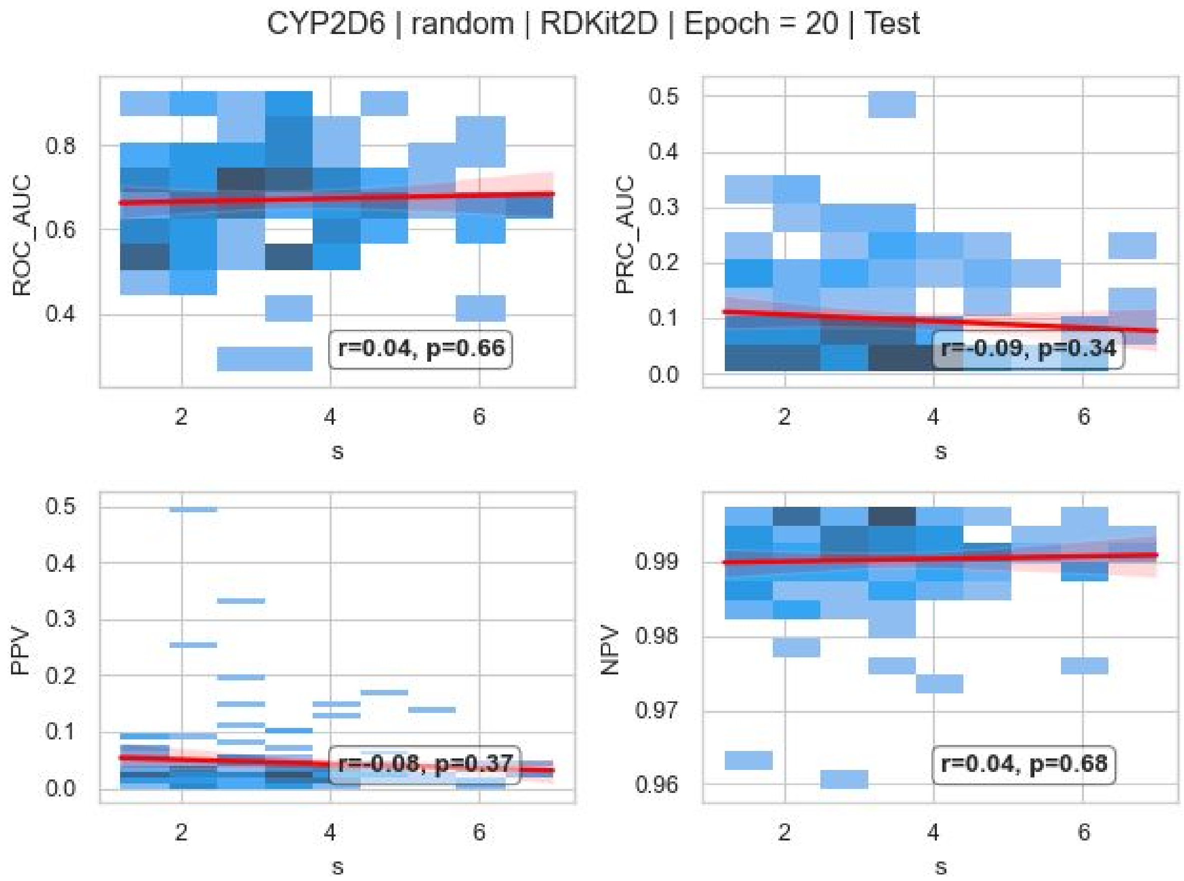

Figure S119: Hyper-parameter *s* and test-set metric scores histogram of CYP2D6 test sets with fitted line for random split-group, RDKit2D descriptors and TM-models of 800 clauses at 20 epochs. Pearson's R and p-value are annotated for said line.

scaffold group-split | RDKit2D Descriptors

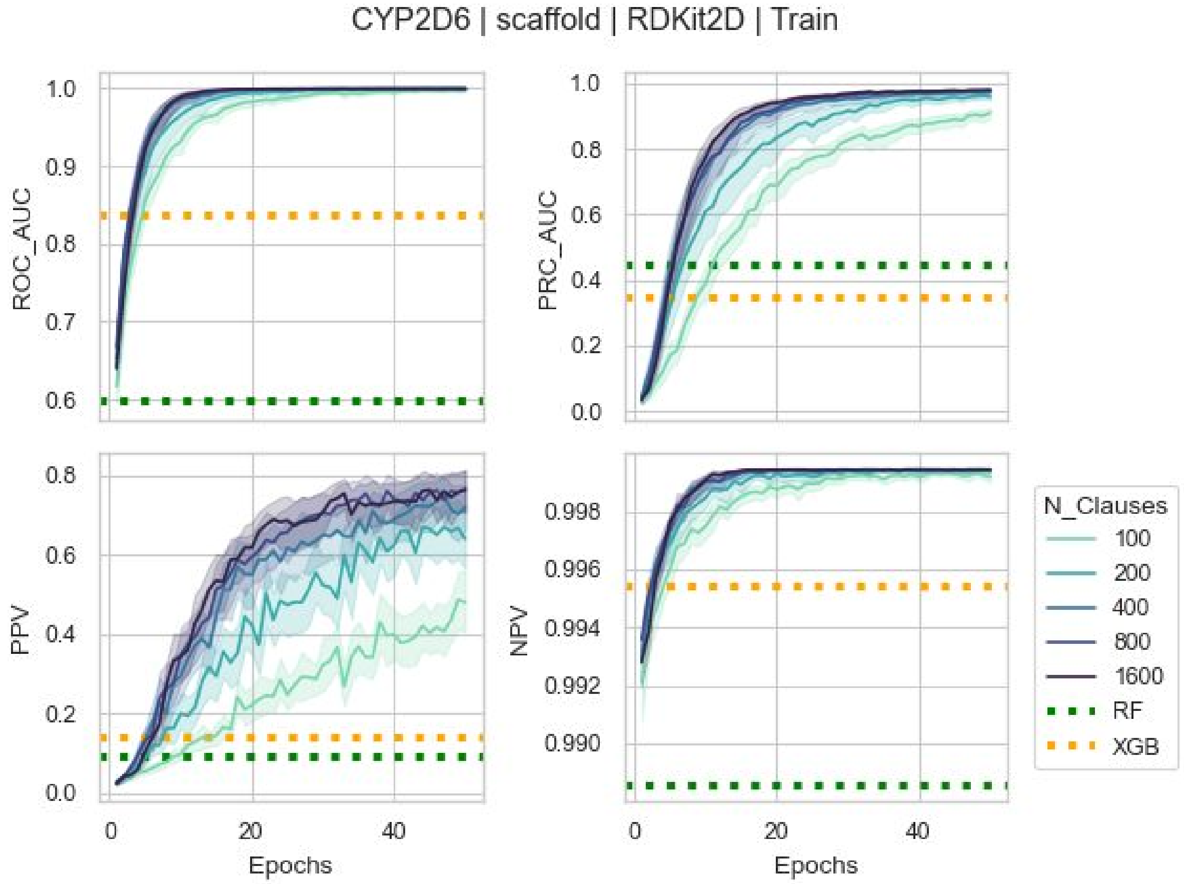

Figure S120: TM metric scores over 50 epochs on CYP2D6 training sets for scaffold split-group and RDKit2D descriptors. Annotated by dotted lines are the mean training set scores of RF and XGBoost.

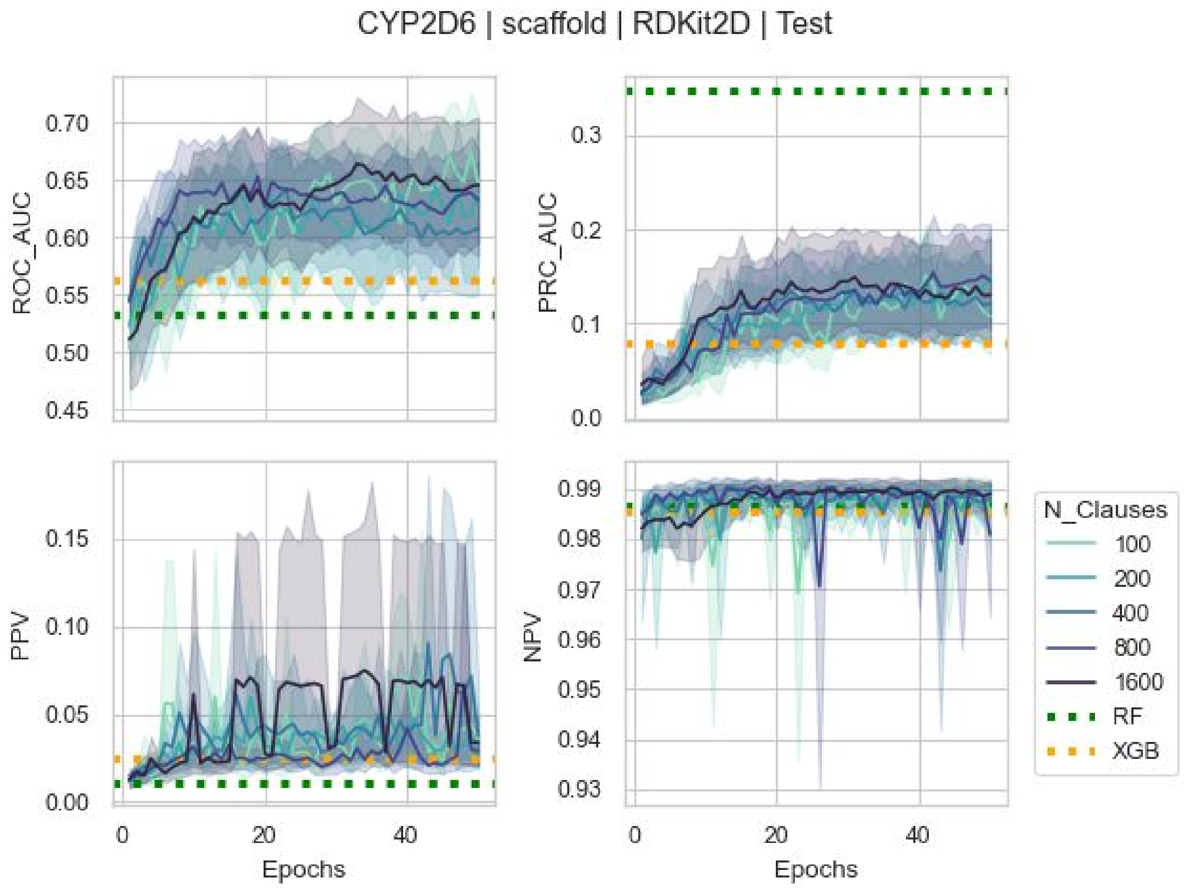

Figure S121: TM metric scores over 50 epochs on CYP2D6 test sets for scaffold split-group and RDKit2D descriptors. Annotated by dotted lines are the mean training set scores of RF and XGBoost.

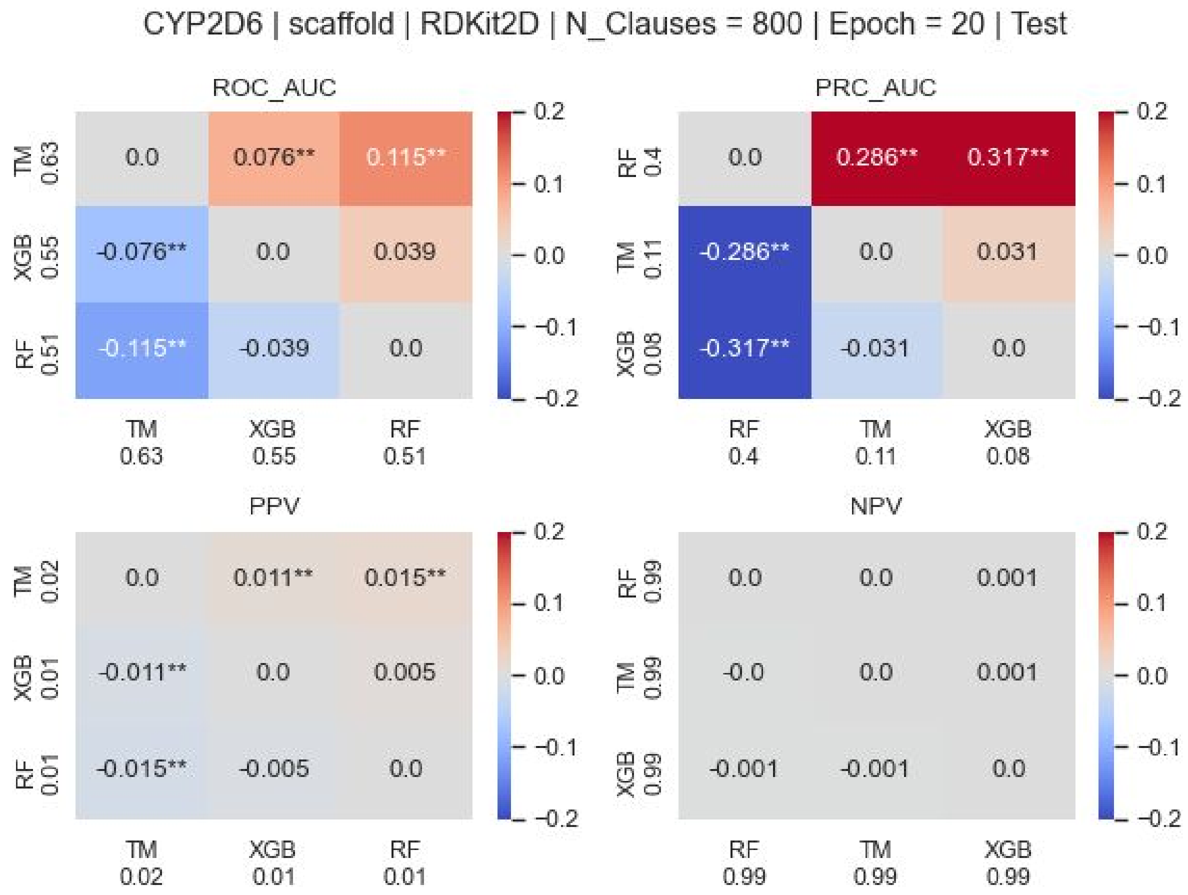

Figure S122: Cohen's D difference of means, pair-wise comparison of models for the CYP2D6 dataset with scaffold group-split, RDKit2D descriptors and TM models of 800 clauses at 20 epochs. Complete with annotated statistical tests via Tukey's HSD where the number of asterix represents a different statistical significance level.

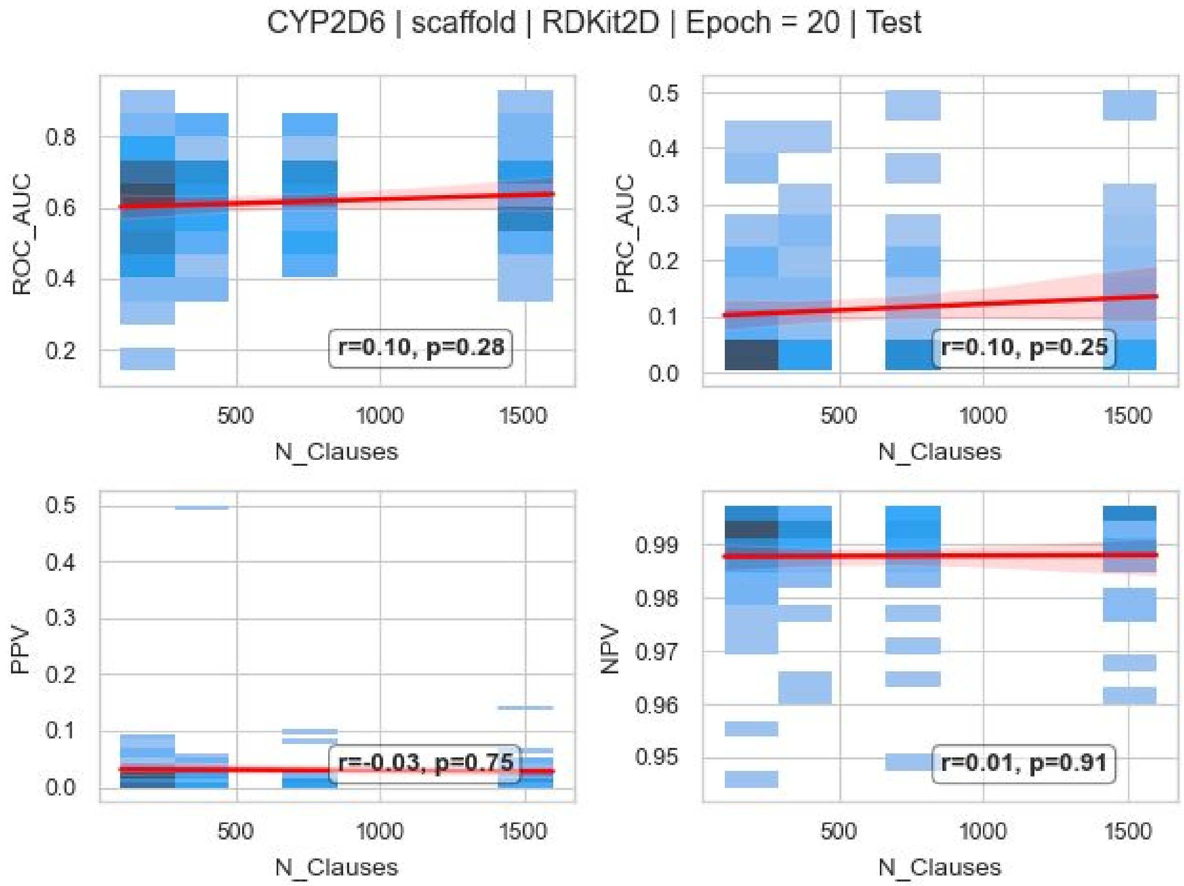

Figure S123: Number of clauses and metric-score histograms of CYP2D6 test sets with fitted line for scaffold split-group, RDKit2D descriptors and TM-models of 800 clauses at 20 epochs. Pearson's R and p-value are annotated for said line.

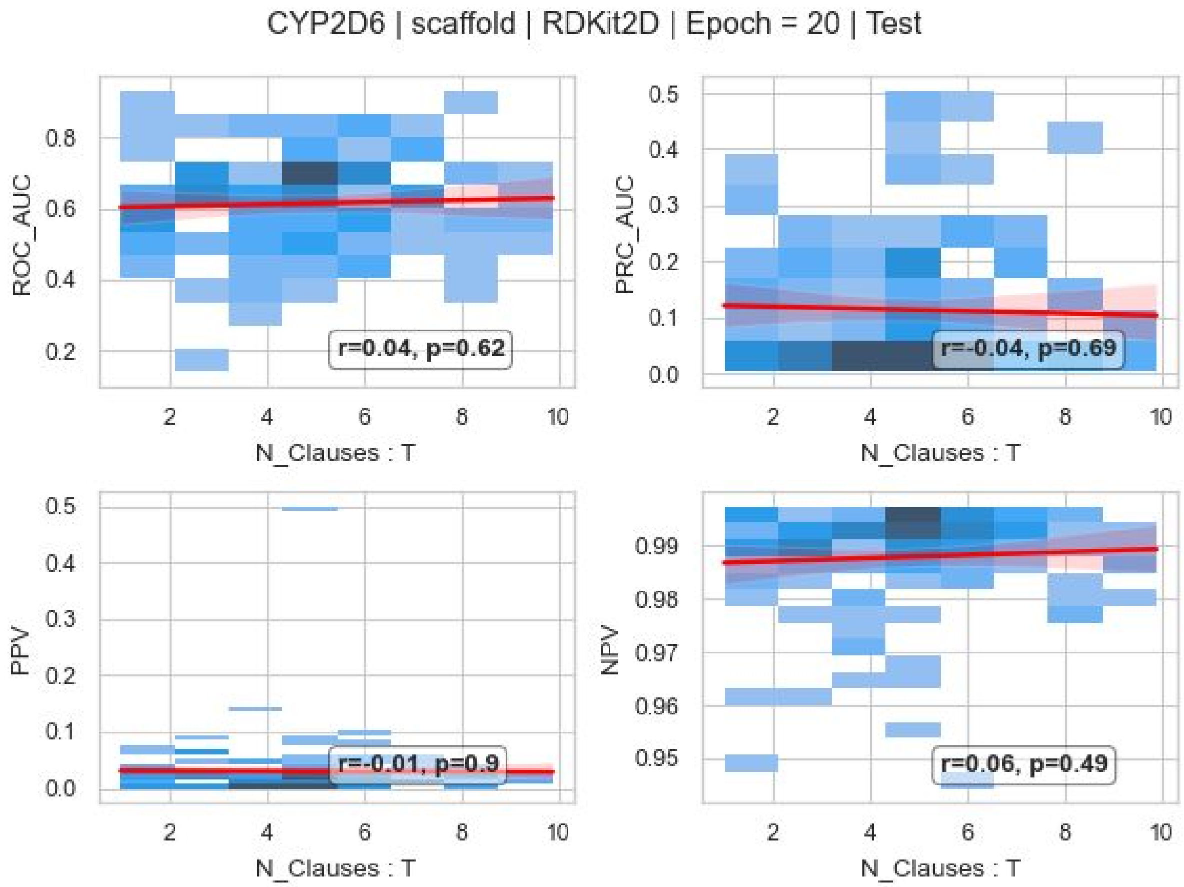

Figure S124: *N_Clauses : T* ratio and test-set metric scores histogram of CYP2D6 test sets with fitted line for scaffold split-group, RDKit2D descriptors and TM-models of 800 clauses at 20 epochs. Pearson's R and p-value are annotated for said line.

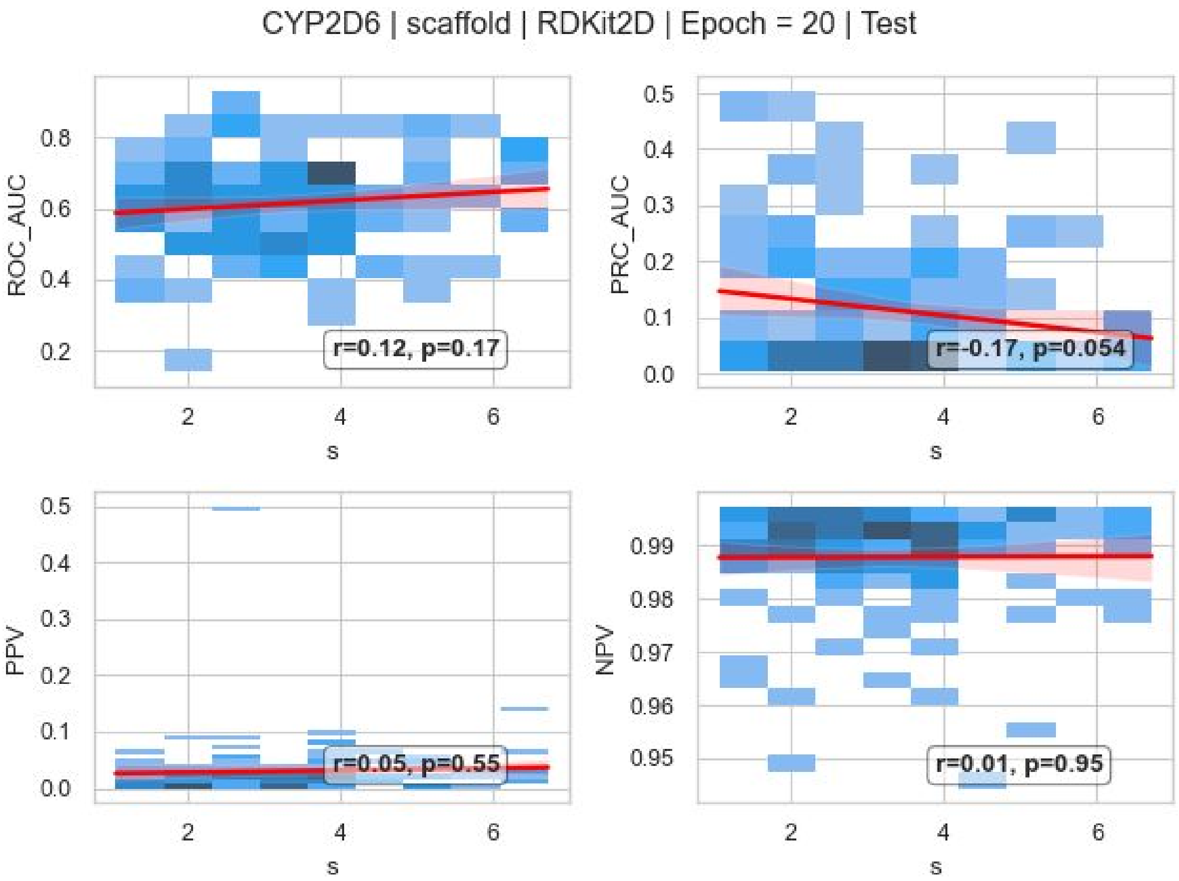

Figure S125: Hyper-parameter *s* and test-set metric scores histogram of CYP2D6 test sets with fitted line for scaffold split-group, RDKit2D descriptors and TM-models of 800 clauses at 20 epochs. Pearson's R and p-value are annotated for said line.
